# Wood–Ljungdahl pathway found in novel marine Korarchaeota groups illuminates their evolutionary history

**DOI:** 10.1128/msystems.00305-23

**Published:** 2023-07-17

**Authors:** Jie Pan, Xinxu Zhang, Wei Xu, Yang Liu, Lirui Liu, Zhuhua Luo, Meng Li

**Affiliations:** 1 Archaeal Biology Center, Institute for Advanced Study, Shenzhen University, Shenzhen, Guangdong, China; 2 Shenzhen Key Laboratory of Marine Microbiome Engineering, Institute for Advanced Study, Shenzhen University, Shenzhen, Guangdong, China; 3 Shenzhen Xbiome Biotech Co. Ltd., Shenzhen, Guangdong, China; 4 Key Laboratory of Marine Biogenetic Resources, Third Institute of Oceanography, Ministry of Natural Resources, Xiamen, Fujian, China; Ocean University of China, Qingdao, Shandong Province, China

**Keywords:** Korarchaeota, Wood–Ljungdahl pathway, marine groups, antioxidation, evolutionary factor, moderate-temperature environment

## Abstract

Korarchaeota, due to its rarity in common environments, is one of the archaeal phyla that has received the least attention from researchers. It was previously thought to consist solely of strict thermophiles. However, our study provides genetic evidence for the presence of korarchaeal members in temperate subsurface seawater. Furthermore, a systematic reclassification of the Korarchaeota based on 16S rRNA genes and genomes has revealed three novel marine groups (Kor-6 to Kor-8) at the root of the Korarchaeota branch. Kor-6 contains microbes that are present in moderate temperatures. All three novel marine phyla possess genes for the Wood–Ljungdahl pathway, and Kor-7 and Kor-8 possess fewer genes encoding oxygen resistance traits than other korarchaeal groups, suggesting a distinct lifestyle for these novel phyla. Our results, together with estimations of Korarchaeota divergence times, suggest that oxygen availability may be one of the important factors that have influenced the evolution of Korarchaeota.

**IMPORTANCE:**

Korarchaeota were previously thought to inhabit exclusively high-temperature environments. However, our study provides genetic evidence for their unexpected presence in temperate marine waters. Through analysis of publicly available korarchaeal reference data, we have systematically reclassified Korarchaeota and identified the existence of three previously unknown marine groups (Kor-6, Kor-7, and Kor-8) at the root of the Korarchaeota branch. Comparative analysis of their gene content revealed that these novel groups exhibit a lifestyle distinct from other Korarchaeota. Specifically, they have the ability to fix carbon exclusively via the Wood–Ljungdahl (WL) pathway, and the genomes within Kor-7 and Kor-8 contain few genes encoding antioxidant enzymes, indicating their strictly anaerobic lifestyle. Further studies suggest that the genes related to methane metabolism and the WL pathway may have been inherited from a common ancestor of the Korarchaeota and that oxygen availability may be one of the important evolutionary factors that shaped the diversification of this archaeal phylum.

## INTRODUCTION

In 1996, phylogenetic analysis of environmental rRNA sequences from a hot spring in Yellowstone National Park (Wyoming, USA) revealed two archaeal sequences that formed a novel cluster distant from Euryarchaeota and Crenarchaeota. This cluster was named Korarchaeota (1). Following the advancements of DNA sequencing technology, additional korarchaeal 16S rRNA gene sequences were detected in various deep-sea hydrothermal vents and terrestrial hot springs, ranging from mildly acidic to neutral pH environments, and from anoxic to oxic sediments (2 - 6). These observations indicated that Korarchaeota are typically thermophilic archaea. In 2006, based on the phylogeny of the 16S rRNA genes, the Korarchaeota cluster was divided into five distinct groups, which corresponded to their distinct geographic distribution (2). In later studies, these phylogenetic clusters were named after their clearly separated geographic locations rather than using the above classification (3, 5), which led to ambiguous korarchaeal classification.

As in the case of most archaeal phyla, no pure culture of Korarchaeota has been obtained to date, and the knowledge of korarchaeal metabolism is mainly inferred from the available genomes. The first obtained korarchaeal genome, *Candidatus* (*Ca*.) Korarchaeum (K.) cryptofilum, was sampled from a strictly anaerobic enrichment of a Yellowstone National Park sample (7). Subsequent gene annotations suggested a heterotrophic lifestyle, with peptides predicted to be the principal carbon and energy sources (7). However, the genome lacks complete biosynthetic pathways for purine and some cofactors, suggesting that the archaeon is a hot spring symbiont or a scavenger (7). The second genomic study of Korarchaeota was conducted 10 yr later. This study reported on the low Korarchaeota diversity in a hot spring in New Zealand and the potential metabolism of metagenome-assembled genome (MAG) NZ13-K, whose metabolic potential was similar to that of *Ca*. K. cryptofilum (8). Since then, several studies have highlighted that some korarchaeal members might lead a mixotrophic lifestyle, potentially utilizing energy from methane and sulfite metabolism (9 - 11). This has expanded the knowledge of the metabolic diversity of Korarchaeota and provided clues on early archaeal evolution.

With the development of next-generation sequencing technology in recent years, some large-scale projects have surveyed microbial diversity in various environments and released dozens of korarchaeal MAGs (12 - 16). Nonetheless, no studies have included these korarchaeal genomes for comparative analysis up to date, and the marine Korarchaeota have been rarely studied in particular. In the current study, we reconstructed a korarchaeal MAG from the metagenome of a seawater column in the Yap Trench in the western Pacific Ocean. Considering its unique habitat (as a member of Korarchaeota), we proposed that the MAG has a distinct taxonomy and metabolism. Therefore, we performed classification and genomic comparison analyses based on available 16S rRNA gene and genome data collected from public databases. Our study highlighted the taxonomic diversity and metabolic characteristics of three novel marine groups of Korarchaeota. Finally, we estimated species divergence times to give some clues about their evolution and possible influencing factors.

## RESULTS AND DISCUSSION

### A novel korarchaeal MAG from subsurface seawater and its classification

**Fig 1:**
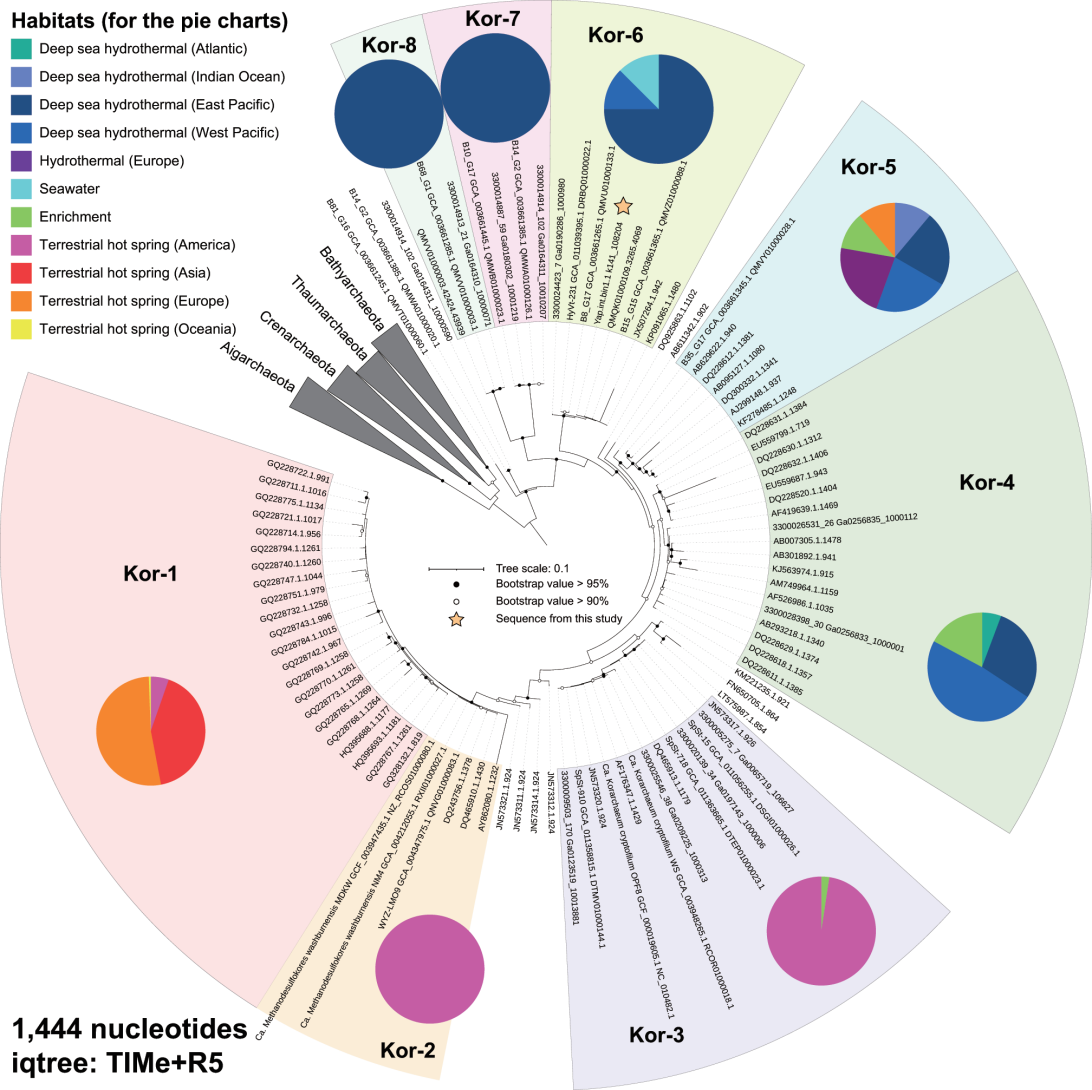
Classification of Korarchaeota according to maximum likelihood tree constructed based on the 16S rRNA gene, and the locations they inhabit. The scale bar indicates the average number of nucleotide substitutions per site. The best-fit model was selected by ModelFinder function in IQ-TREE software.

We sampled 10 layers of seawater in the Yap Trench (Table S1), as previously described (17, 18). The DNA was extracted from the water samples and sequenced (see Materials and Methods). In total, we obtained 446.69 Gbp raw reads from the samples (28.39–65.20 Gbp per sample) (Table S1). Assembly and binning resulted in over 500 bins, including one korarchaeal genomic bin. After bin refinement (see Materials and Methods), the resulting korarchaeal bin, Yap.int.bin1.1, was 2,012,112 bp in length, with an estimated completeness of 88.71% and contamination of 1.87% (evaluated using CheckM v1.0.1162; the results using miComplete v1.1.163 are presented in Table S2). In this MAG, we identified a partial 16S rRNA gene sequence (640 bp) in a 6,571 bp scaffold. Phylogenetic analyses of the 16S rRNA gene (Fig. 1) and the genomes [based on 16 ribosomal proteins (19), 122 single-copy genes (20), and 57 single-copy genes (21); Fig. S1a through f] confirmed that MAG Yap.int.bin1.1 belongs to Korarchaeota.

The first comprehensive classification of Korarchaeota was proposed in 2006 (2). It has not been updated since then. Therefore, we collected all available korarchaeal 16S rRNA gene sequences (266 sequences; >700 bp) and MAGs (42 from published papers and 1 from the current study) to update the classification of Korarchaeota and analyze the taxonomy of MAG Yap.int.bin1.1 (Tables S2 and S3). Phylogenetic analysis of 135 representative 16S rRNA gene sequences (at 99% similarity cutoff) revealed three distinct clusters (intra-cluster similarity >97%, inter-cluster similarity <91%) at the root of the korarchaeal clade, in addition to the five previously described korarchaeal groups (Kor-1 to Kor-5) (2) (Fig. 1). We named these clusters as Kor-6 to Kor-8, accordingly. To note, the 16S rRNA gene sequence of MAG Yap.int.bin1.1 is located within Kor-6 (Fig. 1).

**Fig 2:**
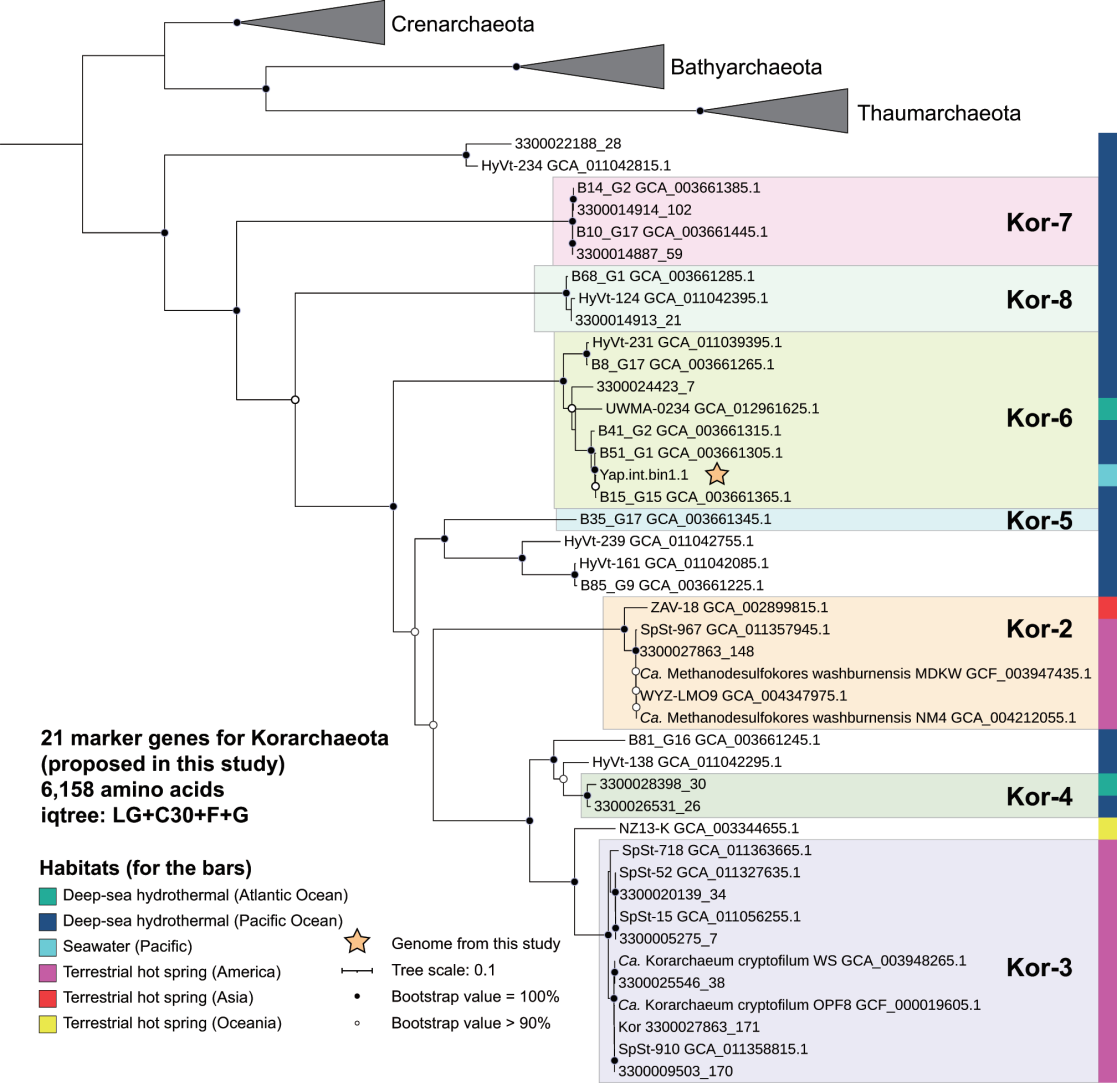
Maximum likelihood tree and group assignment of Korarchaeota MAGs, and the locations they inhabit. Twenty-one single-copy genes were selected and concatenated to construct the phylogeny (see “Determination of marker genes for phylogeny construction” in Materials and Methods). The best-fit model was selected by adding mixture models (C10-C60 models) to ModelFinder function in IQ-TREE software.

We constructed the genomic phylogeny of korarchaeal MAGs using three sets of widely accepted marker genes [16 ribosomal proteins (19), 122 single-copy genes (20), and 57 single-copy genes (21); Fig. S2]. Because some of these marker genes are absent in Korarchaeota and other genes may have undergone phylum-level transfer during the evolutionary history (21), we analyzed and manually checked homologous genes of the respective marker genes in all the available korarchaeal MAGs (Fig. S3 and Table S4). We finally selected 21 homologous genes as a set of marker genes for determining korarchaeal phylogeny (Table S5; the details are described in “Determination of marker genes for phylogeny construction” in Materials and Methods). The genomic phylogenies constructed using these four sets of marker genes were similar to that based on the 16S rRNA gene. In particular, we found at least eight groups within Korarchaeota, with three novel groups (Kor-6 to Kor-8) at the root of the Korarchaeota branch, and MAG Yap.int.bin1.1 is a member of Kor-6 (Fig. 2; Fig. S2 and 4). Thus, the phylogenies of both 16S rRNA genes and genomes support the classification of three novel groups (Kor-6 to Kor-8) and the phylogenetic placement of MAG Yap.int.bin1.1 in Kor-6.

### Korarchaeota in subsurface seawater

To calculate the abundance of Korarchaeota in the water columns, we mapped the raw reads from different water layers to the scaffolds of MAG Yap.int.bin1.1. Surprisingly, Korarchaeota were the most abundant in the seawater layers 10 and 30 m below sea level, accounting for 10.00%–13.99% of all reads (Fig. S5 and Table S1).

Initially, we considered the possibility that the microbial communities in our samples were influenced by the surrounding volcanic activity. However, after reviewing the western pacific hydrothermal sites recorded in the InterRidge Vents Database (https://vents-data.interridge.org/), we found that the closest site to our sample location is the hydrothermal group around Guam (Fig. S6), which is almost 600 km away. This suggests that our samples were unlikely to be affected by volcanic activity.

To further confirm that the microbial communities analyzed in our study were not affected by those from hydrothermal environments, we extracted the taxonomic profiles from all MAGs and merged them with the taxonomic data of marine environments from the Earth Microbiome Project (EMP) database (22) (Table S6), and then performed a metric multidimensional scaling analysis to evaluate the similarities of taxonomic compositions among different environments. The results indicated that the microbial communities in most of our trench seawater samples were more similar to those in marine sediment samples than those in hydrothermal samples (except for Yap-CTD02-BC; Fig. S7). Therefore, the communities in our trench seawater samples were mainly influenced by microbes from moderate-temperature marine sediments rather than those from hydrothermal environments.

We then searched the EMP database (22) and the Integrated Microbial Next-Generation Sequencing (IMNGS) platform data (23) for korarchaeal 16S rRNA gene sequences and found some sequences from subsurface seawater in Norway and the Northwest Antarctic Peninsula (Table 1). Subsequent phylogenetic analysis of 16S rRNA genes showed that these subsurface seawater sequences from the EMP and the IMNGS platform and that from MAG Yap.int.bin1.1 clustered within Kor-6 (Fig. S8). These results imply that the presence of Korarchaeota in the subsurface seawater may not be rare, and these mesophilic/normophilic Korarchaeota belong to Kor-6.

**TABLE 1:** Distribution of Korarchaeota in moderate-temperature environments, according to the information retrieved from the indicated public databases in September 2020

Sample ID	Sample category	Korarchaeal percentage (%)	Location	Latitude degree	Longitude degree	Depth (m)	Projects/sources
1627.DRBC	Lake sediment	0.0046	China: Tibet Darebu Co lake	32.47	83.22	0.025	EMP^*a*^
1039.L.Jacarepia.SA	Marine sediment	0.0010	Brazil: Rio de Janeiro coastline	−23.14	−44.17	0.08	EMP^*a*^
1235.sp1163	Seawater	0.0003	Norway: Svalbard	78.933	11.883	6	EMP^*a*^
1235.sp1304	Seawater	0.0002	Norway: Svalbard	78.933	11.883	6	EMP^*a*^
SRR6829007	Groundwater	0.1202	China:Jianhan Plain	30.37	113.37	–^*c*^	IMNGS platform^*b*^
SRR6829059	Groundwater	0.0020	China:Jianhan Plain	30.36	113.35	–^*c*^	IMNGS platform^*b*^
SRR6829061	Groundwater	0.0113	China:Jianhan Plain	30.39	113.27	–^*c*^	IMNGS platform^*b*^
SRR769613	Lake sediment	0.0323	Kenya: Lake Bogoria	0.3333	36.25	0–1	IMNGS platform^*b*^
SRR769616	Lake sediment	0.1179	Kenya: Lake Bogoria	0.3333	36.25	0–1	IMNGS platform^*b*^
SRR769617	Lake sediment	0.2139	Kenya: Lake Bogoria	0.3333	36.25	0–1	IMNGS platform^*b*^
SRR769626	Lake sediment	0.1222	Kenya: Lake Bogoria	0.3333	36.25	0–1	IMNGS platform^*b*^
SRR5313248	Lake water	0.0010	China: Qinghai	36.56	100.53	–^*c*^	IMNGS platform^*b*^
SRR5465783	Marine sediments	0.0012	Indian Ocean	−34.819	54.684	3523	IMNGS platform^*b*^
SRR5465785	Marine sediments	0.0014	Indian Ocean	−38.953	47.407	1880	IMNGS platform^*b*^
SRR3821671	Seawater	0.0006	Southern Ocean: Northwest Antarctic Peninsula	−61.9985	−55.76	75.14	IMNGS platform^*b*^
SRR5465778	Seawater	0.0047	Indian Ocean	−37.833	49.617	2800	IMNGS platform^*b*^
SAMN13684226	Marine sediment	–^*c*^	India: Gulf of Kutch	22.6	69.5	5–48	(16)
SAMN13684329	Marine sediment	–^*c*^	India: Gulf of Kutch	22.6	69.5	5–48	(16)

^*a*^ Earth Microbiome Project (https://earthmicrobiome.org/).

^*b*^ Integrated Microbial Next-Generation Sequencing platform (https://www.imngs.org/).

^*c*^ –[Table-fn T1_FN3], data not available.

If Korarchaeota are capable of living in the subsurface seawater, some environmental factors may influence their distribution. Therefore, we performed a correlation analysis between chemical parameters and korarchaeal abundance to identify potential environmental preferences of this korarchaeal community. However, we did not observe any significant correlation between the chemical parameters we measured and the abundance of Korarchaeota (Fig. S9). One possibility is that there are still unknown environmental factors in the subsurface seawater that affect the distribution of Korarchaeota, and the subsurface seawater (a moderate environment) is not a preferred habitat for Korarchaeota. To this end, further research is needed to uncover the real reasons underlying this phenomenon.

### Biogeographic separation of Korarchaeota groups

We mapped the geographic information for 266 16S rRNA gene sequences and 43 MAGs onto the phylogeny. In the 16S rRNA gene aspect, all Kor-4, Kor-6, Kor-7, and Kor-8 sequences were from marine environments, whereas all Kor-1, Kor-2, and Kor-3 sequences were from terrestrial springs. This suggests an apparent biogeographic separation of the marine and terrestrial Korarchaeota groups (Fig. 1). In addition, within the terrestrial groups, the Kor-2 and Kor-3 sequences were exclusively derived from North America (Fig. 1), and the Kor-1 sequences formed two clusters originated from the Kamchatka Peninsula and Iceland, respectively (Fig. S10). However, within the marine Kor-4 and Kor-6 groups, members from different oceanic regions clustered together (Fig. 1), suggesting no biogeographic separation within the marine Korarchaeota community.

Similarly, we observed an obvious biogeographic separation between the marine (the Kor-4 to Kor-8 MAGs) and terrestrial MAG groups (the Kor-1, Kor-2, and Kor-3 MAGs). Within the terrestrial groups, all Kor-3 MAGs were from North America, and the Asia-originated MAG in Kor-2 was phylogenetically distant from America-originated MAGs in Kor-2 (Fig. 2). These observations support the notion of biogeographic separation of the Korarchaeota reported in previous studies (3, 4).

In particular, both phylogenies based on the 16S rRNA gene and MAGs indicate that the marine-originated groups Kor-6 to Kor-8 are phylogenetically located at the root of the Korarchaeota branch (Fig. 1 and 2). This strongly supports the marine origin of Korarchaeota, as proposed previously (3, 5).

On the other hand, some clusters present in one tree did not appear in the other tree, such as clusters near Kor-4 and Kor-5 in the genomic tree and sequences near Kor-2 in the 16S rRNA gene tree. This observation suggests the existence of other korarchaeal groups. More genomic data from different geographic locations are needed, as insufficient korarchaeal 16S rRNA gene sequences and MAGs hamper our understanding about the diversity of Korarchaeota.

However, obtaining high-quality genomes of archaea can be challenging due to their low abundance in the environments. To address this issue, several strategies have been employed, such as increasing the sequencing depth of metagenomes and introducing third-generation sequencing technologies to obtain long-read sequences (24). These advancements have resulted in the generation of more high-quality genomes for low-abundance archaea, revealing the remarkable diversity of archaeal communities across various environments.

### Metabolic distinction of the novel Korarchaeota groups

**Fig 3:**
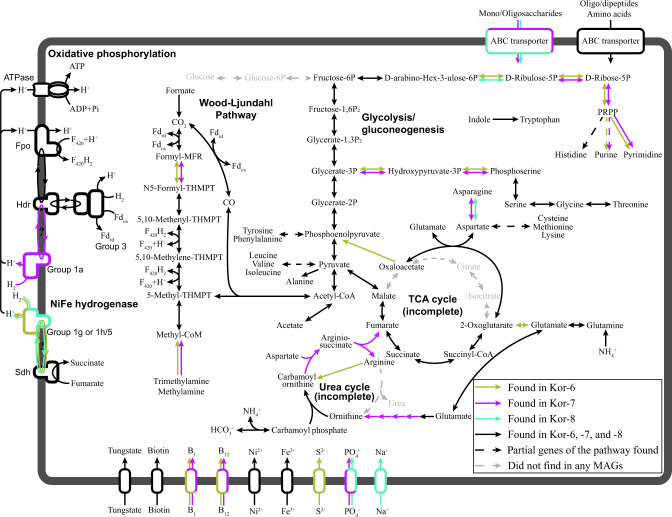
Carbon and energy metabolism of Kor-6, Kor-7, and Kor-8 members. Metabolic construction is based on the presence of protein-coding genes in the relevant MAGs.

**Fig 4:**
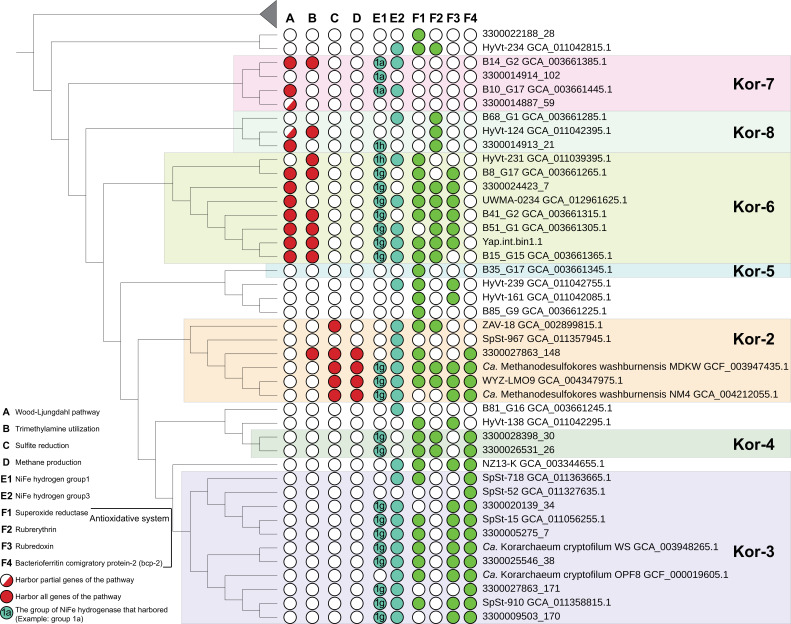
The presence and absence of selected functional genes and pathways in korarchaeal MAGs. The genes and copy numbers are detailed in Table S7. The tree is an edited version of that presented in Fig. 2, generated by ignoring the branch lengths. The presence of the Wood–Ljungdahl pathway was considered if more than half of the associated genes were present. The presence of a partial Wood–Ljungdahl pathway was considered if genes for more than one subunit of the archaeal-type CODH/ACS (*cdhABCDE*) were present.

To explore and compare the potential metabolism of korarchaeal groups, we annotated the genes in all korarchaeal MAGs and identified the homologous genes among the MAGs. Multidimensional scaling analysis of the number of homologous gene in each MAG showed that the MAGs of the same group were significantly clustered together, while those of different groups were significantly separated (Fig. S11). This result suggests that different korarchaeal groups may exhibit different metabolic potentials or lifestyles. Previous studies have suggested a symbiotic lifestyle of *Ca*. K. cryptofilum (Kor-3) (7, 25) and a mixotrophic lifestyle of *Ca*. Methanodesulfokores washburnensis (Kor-2) with energy derived from methanogenesis and dissimilatory sulfite reduction (10), indicating a major difference between their survival strategies. Therefore, we hypothesize that the potential metabolism of three novel marine Korarchaeota groups (Kor-6, Kor-7, and Kor-8) is distinct from other Korarchaeota groups according to the multidimensional scaling analysis result (Fig. S11). According to our annotations, most Kor-6, Kor-7, and Kor-8 MAGs exclusively harbor the entire set of genes related to the WL pathway, and none of them harbor the genes of glucokinase and glucose-6-phosphate isomerase for the utilization of glucose via glycolysis (Fig. 3 and 4). Therefore, the three novel marine Korarchaeota groups have the genomic potential to utilize carbon dioxide via the WL pathway for carbon assimilation. Notably, our gene prediction results revealed that no WL pathway genes were found in the genomes of other korarchaeal groups. Even some lineages within Kor-2 that are considered as the potential methanogens (10) do not harbor the genes encoding the key enzyme of the WL pathway, acetyl-CoA synthase/CO dehydrogenase. Therefore, the lifestyle of Kor-6, Kor-7, and Kor-8 is likely to be significantly different from that of other korarchaeal groups.

Furthermore, according to a previous study (10), the heterodisulfide reductase complex and the NiFe hydrogenase, together with the sulfite reductase or methane metabolism, are the major energy-generating pathway in Kor-2. In the Kor-6, Kor-7, and Kor-8 MAGs, we also found genes for the heterodisulfide reductase complex and various NiFe hydrogenases. However, instead of genes related to methane and sulfate metabolism, we only found genes for a potential F420 dehydrogenase. These observations suggest that members of these three groups may adopt a potential energy-generating strategy similar to those reported in some methanogens, where the heterodisulfide reductase and the NiFe hydrogenase catalyze the reduction of F420 and the produced F420H_2_ can be utilized in the WL pathway to generate F420 and drive its reduction [Fig. 3, reviewed by Diender et al. (26)].

Given the above evidence for these energy-generating genes and the WL pathways in their genomes, it is highly possible that Kor-6, Kor-7, and Kor-8 utilize the WL pathway for both carbon fixation and energy generation. Therefore, these groups may lead an autotrophic lifestyle in the marine environment.

In addition, we identified a large number of genes for amino acid or mono-/oligosaccharide ABC transporters, as well as some genes for incomplete purine and some amino acid biosynthetic pathways in the Kor-6, Kor-7, and Kor-8 MAGs (Fig. 3), similar to those in the Kor-3 members (7). Thus, the three marine Korarchaeota groups may also be able to directly take up and utilize extracellular amino acids. They also harbor genes for an incomplete tricarboxylic acid cycle, and a gene for aspartate aminotransferase, which converts oxaloacetate and glutamate to 2-oxoglutarate and aspartate (Fig. 3). The coexistence of these genes has also been noted in Kor-3 (10) and other archaeal phyla (27, 28), and these genes are thought to control the generation of biosynthetic precursors in these microorganisms (10). Taken together, the three novel korarchaeal groups may possess another carbon utilization pathway by scavenging amino acids in the environment to synthesize organic compounds. The genomic capability of utilizing both carbon dioxide and organic carbon in the environment provides the metabolic flexibility for better environmental adaptation of these microbes.

In addition, we evaluated the oxygen sensitivity of all korarchaeal MAGs by searching for the presence of genes encoding antioxidant enzymes. The results showed that such genes are absent in Kor-7 MAGs, and members of Kor-8 harbor only the rubrerythrin gene. Kor-6 MAGs encode superoxide reductase, rubrerythrin, and rubredoxin, while Kor-2 MAGs harbor all four genes related to the antioxidant system as shown in Fig. 4. In the majority of hyperthermophilic aerobic/microaerophilic and anaerobic archaea, superoxide reductase is the key enzyme of the oxidative stress defense system. It first reduces superoxide to hydrogen peroxide with reduced rubredoxin (29, 30), and the generated hydrogen peroxide is then reduced to water by rubrerythrin (31). Bacterioferritin comigratory protein-2 (Bcp-2) has been reported to be involved in the detoxification of hydrogen peroxide (32). Therefore, the absence of essential antioxidant genes in the Kor-7 and Kor-8 MAGs suggests that these microbes may be highly oxygen sensitive and strictly anaerobic. On the contrary, members within Kor-6 may tolerate low levels of oxygen. Furthermore, within these novel marine Korarchaeota groups, the NiFe hydrogenase group 1 genes harbored by the Kor-6 MAGs belong to group 1g (same as those harbored by Kor-2, Kor-3, and Kor-4), while those harbored by the Kor-7 and Kor-8 MAGs belong to groups 1a and 1h/5, respectively (Fig. 4). NiFe hydrogenase group 1a is oxygen sensitive and participates in methanogenic heterodisulfide respiration in Euryarchaeota (33, 34); NiFe hydrogenase group 1h/5 scavenges electrons to maintain aerobic respiration during starvation (33, 35); and the NiFe hydrogenase group 1g is considered to be an oxygen-tolerant hydrogenase as it is harbored by facultatively aerobic Crenarchaeota (36, 37). Thus, the different oxygen sensitivity of the korarchaeal groups may be adapted to their genes encoding antioxidation-related enzymes and hydrogenases with different levels of oxygen tolerance.

### Evolution of Korarchaeota

#### Archaea tree marker gene selection

The genomic comparisons of Korarchaeota revealed pronounced differences in the metabolic potential of the three novel marine Korarchaeota groups, especially in the WL pathway and the antioxidant genes discussed above. The results raise the question of how these groups obtained or evolved these metabolic potentials, and whether the genes involved were inherited from a common ancestor or transferred from other microbes. In this section, we used gene-gain and -loss analysis and Bayesian estimation of species divergence times to explore the evolution of the WL pathway and the antioxidant genes within Korarchaeota.

Since both analysis methods depend on the availability of a reliable phylogenetic tree of Archaea, we first constructed phylogenetic trees based on three accepted sets of marker genes for Archaea [16 ribosomal proteins (19), 122 single-copy genes (20), and 57 single-copy genes (21)] using selected archaeal genomes. Referring to previous studies (21, 38), the roots of these archaeal trees were placed between the DPANN members and other archaea, as the DPANN members form a rapidly evolving branch distinct from other archaea members (39). The results show that the phylogenetic placement of Korarchaeota, Asgard archaea, and other TACK archaea in the tree based on 57 single-copy genes (Fig. S1e and f) was different from those constructed using the other two sets of marker genes (Fig. S1a through d). Furthermore, a recent study showed the unstable evolutionary position of Korarchaeota (40). All these results suggest that the marker genes should be re-evaluated to allow the construction of a more reliable archaeal tree. The set of 57 single-copy genes was refined from the set of 122 archaeal single-copy genes in a previous study (21) to minimize the effect of host–symbiont horizontal gene transfer (HGT) between Euryarchaeota and DPANN archaea. In the current study, we individually analyzed the 57 single-copy genes (Fig. S13) and selected 37 genes for subsequent phylogenetic analysis (as described in “Determination of marker genes for phylogeny construction” in Materials and Methods; Tables S8 and S9). We then constructed an archaeal phylogenetic tree based on the concatenated sequence alignment of 37 marker genes extracted from 283 high-quality archaeal MAGs (completeness >90%; for Korarchaeota and DPANN archaea, completeness >85%; the genes are listed in Table S10, and the trees are shown in Fig. S1g and h). This tree was used for the gene-gain and -loss analysis and the estimation of species divergence times.

#### Evolution of the WL pathway and methane metabolism

The WL pathway is considered a metabolic pathway of ancient origin, which involves a series of biochemical reactions to reduce carbon dioxide and produce acetyl-CoA. To predict the gene duplication, transfer, and loss events in the korarchaeal evolution, all genes related to the WL pathway were selected to perform the gene-gain and -loss analysis using the amalgamated likelihood estimation (ALE) algorithm. This algorithm first sets a reconciled gene tree from a sample of gene trees and then estimates gene copy numbers and event rates at the nodes of the species tree (41). The species tree is provided in Fig. S14, and the results of gene copy number and event rate estimates are listed in Table S11. In addition, the branch nodes are considered the common ancestor of the branch members (e.g., Node 6 is the common ancestor of Korarchaeota).

The ALE results indicated that most of the genes related to the WL pathway were present in the common ancestor of Korarchaeota (Node 6 in Fig. S14 and Table S11), with estimated copy numbers of 0.53–1.92. These results suggest that the common ancestor of Korarchaeota harbors the genes related to the WL pathway, and the three novel marine groups may have inherited the WL pathway from their common ancestor. To further confirm this finding, we constructed a phylogenetic tree using the key gene cluster of the WL pathway (*cdhABCDE*, encoding CO-methylating acetyl-CoA synthase). In this tree, sequences from TACK and Asgard archaea clustered together (Fig. S15a), which was consistent with the topology of the genomic tree (Fig. S1g and h). Therefore, the results suggest that the evolution of *cdhABCDE* may be accompanied by the archaeal evolution, and this gene cluster is more likely to have been passed from the common ancestor of TACK to the common ancestor of Korarchaeota, then to the three novel Korarchaeota groups found in this study. Therefore, both ALE and phylogenetic results support that the korarchaeal WL pathway was inherited from the common ancestor of Korarchaeota.

However, in the ALE results, we observed high estimated values for transfer events at the nodes within the Korarchaeota branch, such as a value of 0.62 for the *cdhA* gene at Node 9, and 0.39 and 0.41 for the *cdhB* gene at Node 9 and MAG B14_G2 GCA_003661385.1 (Table S11). Since Node 9 is the common ancestor of Kor-6 (Fig. S14), the high transfer value at Node 9 suggests that the *cdhA* and *cdhB* genes in Kor-6 were transferred from the other microbes. Phylogenetic analyses of individual genes within the *cdhABCDE* gene cluster were also performed. The results showed that the korarchaeal sequences of *cdhA* and *cdhB* genes did not cluster together, and the sequences of these genes within Kor-6 and Kor-8 did not cluster with the sequences of Bathyarchaeota (Fig. S15b and c), which was different from the patterns revealed by the *cdhC* and *cdhD* phylogenies (Fig. S14d and e). These observations suggest possible HGT events of the WL pathway genes (such as *cdhA* and *cdhB*) between Korarchaeota and other microbes. Taken together, all the results suggest that the WL pathway harbored by the novel korarchaeal groups was inherited from the common ancestor of Korarchaeota, except for some possible HGT events that may have occurred between Kor-6 and Kor-8 and other microbes.

It is known that the WL pathway is coupled to methane metabolism to generate energy and fix carbon in hydrogenotrophic methanogens (42). Considering this, we also performed ALE analysis of the genes related to methane metabolism. The estimated copy numbers of genes related to methane metabolism (*mcrACDG*; key gene cluster encoding methyl-coenzyme M reductase) for Node 5 (common ancestor of TACK superphylum) ranged from 0.41 to 0.83, and the estimated transfer values of these genes in Node 12 (common ancestor of methane-metabolizing Korarchaeota) ranged from 0.15 to 0.47 (Fig. S14 and Table S11). These numbers were insufficient to infer whether these genes were inherited from the common ancestor of Korarchaeota. Therefore, the phylogenies were constructed based on the *mcrA* gene and the concatenated sequence alignment of the *mcrABCDG* genes. The results show that the korarchaeal sequences were located at the root of the TACK branch (Kor-2, Thaumarchaeota, Nezhaarchaeota, and Vestraetearchaeota in Fig. S16), which was consistent with the relative position of Korarchaeota in the genomic tree of Archaea (Fig. S1g and h). Thus, the Kor-2 *mcrABCDG* genes may have been inherited from the common ancestor of Korarchaeota, which supports the finding of a previous study (10).

Furthermore, the common ancestor of Korarchaeota may have contained the genes related to both the WL pathway and methane metabolism, which are two important components of the hydrogenotrophic methanogenic pathway. The tetrahydromethanopterin *S*-methyltransferase complex (encoded by *mtrABCDEFGH*), which is believed to have evolved later than the WL pathway and methane metabolism, links the WL pathway and methane metabolism in carbon dioxide-reducing methanogenesis (43). However, most of the korarchaeal MAGs analyzed in the current study encode for only one subunit (*mtrH*), and its gene product does not have the full activity of the tetrahydromethanopterin *S*-methyltransferase complex (44). This leads to an inference that the common ancestor of Korarchaeota may have been incapable of hydrogenotrophic methanogenesis. Altogether, the common ancestor of Korarchaeota may have had a flexible lifestyle by fixing carbon via the WL pathway and producing methane from methylated compounds.

#### Evolution of antioxidant genes

As mentioned above, the absence of antioxidant genes in the Kor-7 members may suggest a strictly anaerobic lifestyle, which is very different from those of the other Korarchaeota groups. Therefore, a gene-gain and -loss analysis of the antioxidant genes was performed, including the genes encoding superoxide reductase, rubredoxin, rubrerythrin, and bcp-2. The results revealed low estimated gene copies of the antioxidant genes, except for the ones of superoxide reductase, in the common ancestor of Korarchaeota (Node 5 in Table S11; 0.01–0.18). Also, high estimated transfer values were observed for the common ancestors of the other Korarchaeota groups (Table S11; 0.62 for the rubredoxin gene in Node 9, 0.6 for the rubrerythrin gene in Node 9, and 0.47 for the bcp-2 gene in Node 10). These results suggest that these antioxidant genes may have been acquired by HGT from other microbes.

**Fig 5:**
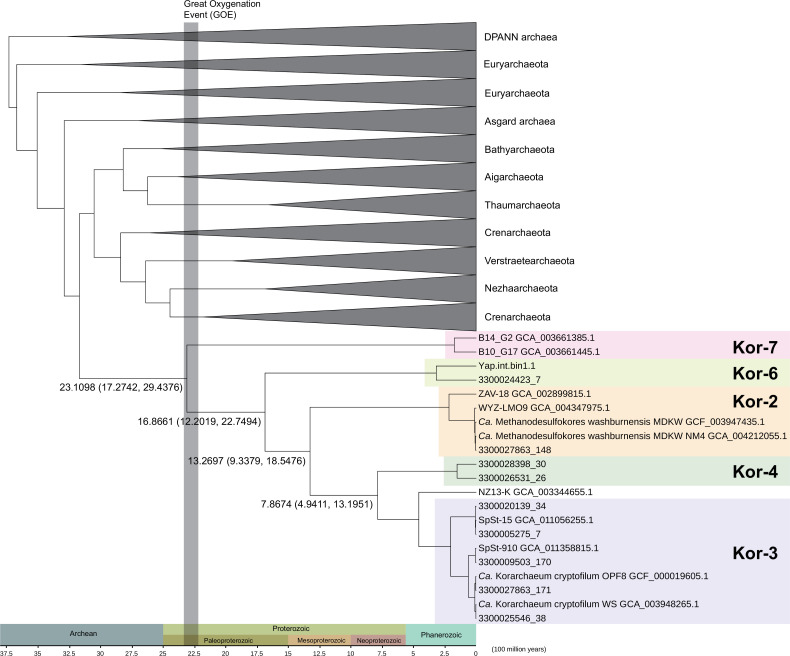
Evolutionary timeline of Korarchaeota groups estimated by using MCMCTree and a phylogenetic MAGs tree across Archaea. The numbers at the branches are the estimated times (Mya; posterior 95% equal-tailed confidence intervals in brackets) of the nodes. The Bayesian tree was constructed using concatenated sequences of 37 marker genes (see “Determination of marker genes for phylogeny construction” in Materials and Methods). The three vertical gray bars represent the timing of the great oxygenation event (2,330 Mya), the predicted breakup of the supercontinent at the beginning of the Mesoproterozoic Era, and the predicted breakup of the Rodinia supercontinent, accordingly. The clades of other phyla have been collapsed in the tree to highlight the divergence time of the Korarchaeota groups. A full-tree version is shown in Fig. S17.

Furthermore, we calculated the divergence times of archaeal species by performing Bayesian estimation under a molecular clock model with the constraints of Thermoproteales and Sulfolobales (after the GOE; the Great Oxygenation Event) and the most recent common ancestor of *Sulfolobus solfataricus* and *Sulfolobus islandicus* (475 million years ago; Mya) (45). The analysis estimated the first divergence of Korarchaeota at 2,311 Mya [posterior 95% equal-tailed confidence interval (CI): 1,724–2,944 Mya], i.e., during the GOE (2,220–2,320 Mya) (46, 47) (Fig. 5). This coincidence led us to hypothesize that the divergence of Kor-7 and the other korarchaeal groups may be related to the GOE. The GOE increased the oxygen content of the ocean and the atmosphere and changed the life and environments on Earth, as reviewed by Sessions et al. (48). Many studies have reported the impact of the GOE on the evolution of genes (49), pathways (50 - 52), and species (45, 53, 54). In the current study, combined results of the possible transfer of antioxidant genes and the divergent time estimation suggest that the gene pools of different korarchaeal groups seem to be strongly affected by environmental oxygen levels, which may be a consequence of the GOE. Therefore, the emergence of oxygen may contribute to the evolution of Korarchaeota, and oxygen availability may be one of the evolutionary drivers of Korarchaeota.

In summary, Korarchaeota is one of the least studied archaeal phyla and was previously known to include strict thermophiles. The current study provides genetic evidence for the presence of korarchaeal members in moderate-temperature subsurface seawater. Furthermore, our systematic reclassification of Korarchaeota reveals the presence of three novel marine groups (Kor-6, Kor-7, and Kor-8) at the root of the Korarchaeota tree. These three novel groups have the genomic potential to fix carbon via the WL pathway. The absence of antioxidant genes in Kor-7 and Kor-8 suggests that they may be strictly anaerobic. Further analyses show that the korarchaeal genes related to methanogenesis and the WL pathway were inherited from their common ancestor, suggesting that the common ancestor may have lived a flexible lifestyle by fixing carbon via the WL pathway and producing methane from methylated compounds. Oxygen availability may affect the gene pools of individual korarchaeal groups and can be one of the important factors that have shaped the evolution of Korarchaeota. Taken together, our study expanded the taxonomic and metabolic diversity of Korarchaeota and inferred a possible evolutionary path of Korarchaeota.

## MATERIALS AND METHODS

### Sample collection and metagenomic sequencing

Sample collection, DNA extraction, and metagenomic sequencing have been described in detail elsewhere (17). Briefly, samples from 10 layers of seawater (0, 10, 30, 50, 100, 200, 500, 1,000, 3,000, and 6,000 m below sea level) in water column Yap-CTD02 (9.90° N, 138.51° E) were collected from the Yap Trench in the western Pacific Ocean during the 37th Dayang cruise in 2016. Chemical parameters of the samples were determined in triplicate as previously described and are presented in Table S1 (17, 18). All samples were filtered through a 0.22-µm membrane filter. The membranes were then stored at –80°C until DNA extraction. DNA extraction was performed using Mo Bio Laboratories PowerWater DNA Isolation Kit according to its protocol. The extracted DNA was examined by gel electrophoresis and quantified using a NanoDrop Microvolume Spectrophotometer. The qualified samples were sent to Novogene Co., Ltd. (Tianjin, China) for metagenomic sequencing using Hiseq X Ten PE150 (Illumina, USA) following Illumina’s protocol.

### Genome construction

Raw metagenomic reads were dereplicated (100% identity over 100% length) and trimmed using Sickle v1.33 (55). Then, the remaining reads were *de novo* assembled into scaffolds using IDBA_UD v1.1.1 and the parameters “-mink 65 -maxk 145 -steps 10” (56). Subsequently, the scaffolds were binned using MetaBAT v2.12.1 with default parameters (57). More than 500 bins were obtained, which included one korarchaeal bin [1.97 Mbp long, 87.56% completeness, and 2.8% contamination, as evaluated by CheckM v1.0.11 (58)]. To improve the quality of this bin, the bin and all korarchaeal genomes available in the NCBI Assembly database (last accessed in September 2020) and Nayfach et al. (15) study were collected to form a study-specific database; then, the raw reads from Yap Trench seawater sequenced in the current study were mapped to this database using BWA v0.7.14 with default parameters (59). All mapped reads were extracted, and the assembly and binning steps repeated, as described above. Finally, one korarchaeal MAG was obtained and further manually cleaned using mmgenome tool (60). The completeness and contamination of this MAG were calculated using CheckM v1.0.11 (58) and miComplete v1.1.1 (61). The MAG taxonomy was first assigned using the GTDB-Tk package v1.3 (20). Its classification was further analyzed phylogenetically based on the 16S rRNA gene (Fig. 1), three accepted sets of marker genes [16 ribosomal proteins (19), 122 single-copy genes (20), and 57 single-copy genes (21)], and the marker genes refined in the current study (see “Determination of marker genes for phylogeny construction”). The phylogenetic methods are described in “Phylogenetic analysis.”

### Gene annotation

The 16S rRNA genes were predicted and taxonomically assigned by using BLASTn against the SILVA NR99 database v138 (62). Prodigal v2.6.3 was used for gene calling in korarchaeal MAGs (63). Genes were annotated by combining the results of the EggNOG-mapper annotated pipeline v5.0 (default parameters) (64) and BLASTp searches (e-value cutoff is 1e−5) against the Non-Redundant Protein Sequence Database (NR database; retrieved in April 2020). To further classify the functional groups of NiFe hydrogenase of Korarchaeota, the hydrogenase-encoding genes were aligned with the reference sequences from Greening et al. (33). The annotated results of all genes mentioned in this study are listed in Table S12.

### Genomic abundance calculation and comparison

To calculate the relative abundance of Korarchaeota and other taxa in the different layers of the seawater column, raw sequencing reads were mapped to the MAG scaffolds using BWA v0.7.14 with default parameters (59). The relative abundances were calculated using the Reads Per Kilobase per Million mapped reads method, with the formula: (number of mapped reads)/(MAG length × metagenomic size) (65). To analyze the correlations between the korarchaeal abundance and environmental parameters, a Spearman test was performed, and the results are visualized using corrplot package (66). To compare the microbial compositions of Yap Trench samples in our study with those in marine environments within EMP (22), we first redirected the taxonomic information used in EMP to the correspondent taxonomy in Genome Taxonomy Database (20). Then the taxonomic table of our samples was constructed by calculating the genomic abundance. Finally, two taxonomic tables were merged together for conducting multidimensional scaling analysis by using vegan package (67).

### Determination of marker genes for phylogeny construction

Twenty-one marker genes for Korarchaeota phylogenetic analysis used in the current study were selected from among homologous genes in korarchaeal MAGs. First, homologous genes in all korarchaeal MAGs were analyzed by using OrthorFinder v2.5.2 (68) with parameters “-S mmseqs -M msa.” This yielded 6,034 homologous genes within Korarchaeota. Of these, 115 genes are only present in a single copy in each korarchaeal MAG with completeness >85%. The individual phylogenies of the 115 genes were then analyzed by building maximum-likelihood trees, accordingly. All the trees are presented in Fig. S3. Among the 115 genes, korarchaeal sequences of 21 genes were monophyletic in the trees (Table S4). Therefore, the 21 genes were selected as the marker genes for korarchaeal phylogenetic analysis (Table S5).

Archaeal phylogeny of 283 high-quality genomes was first constructed by using three accepted marker gene sets [16 ribosomal proteins (19), 122 single-copy genes (20), and 57 single-copy genes (21); see “Phylogenetic analysis” section below]. However, the relative positions of Asgard archaea, Korarchaeota, and other TACK archaea in the 57-marker-gene trees were different from those in the 16-ribosomal-protein and 122-marker-gene trees (Fig. S1). Because the 57-marker gene set has been refined from the 122-marker gene set by considering the effects of the host–symbiont horizontal gene transfer between Euryarchaeota and DPANN archaea (21), the marker genes for Archaea were refined from the set of the 57 marker genes. Individual phylogenies of 51 genes (six genes were discarded because they are harbored by less than half of the analyzed MAGs) were analyzed by building maximum-likelihood trees, accordingly. All the trees are presented in Fig. S13. Since the Korarchaeota genomes did not cluster with the Euryarchaeota and DPANN archaea genomes in the phylogenies of the three accepted marker gene sets (21, 69, 70) (Fig. S1), a gene was discarded if the corresponding korarchaeal sequences clustered with the sequences of Euryarchaeota and DPANN archaea (Table S8). Ultimately, 37 genes were selected as the marker genes for archaeal phylogeny construction (Table S9).

### Phylogenetic analysis

For the analysis, 16S rRNA gene sequences of Korarchaeota were obtained from the SILVA SSUParc database v138 (62) and extracted from all the Korarchaeota MAGs available in the NCBI Assembly database (last accessed in September 2020) and Nayfach et al. (15) study. Most of the sequences from the SILVA database were obtained through PCR-mediated sequencing (Table S3). However, some of these sequences are short (due to the specific primers used in the referenced studies) so the sequences less than 700 bp were excluded from our study. The remaining sequences were clustered at 99% similarity using USEARCH v10.0 (71); 135 representative sequences were thus obtained. For genomic phylogenetic analysis, three accepted sets of marker genes were selected: 122 archaeal single-copy genes, predicted using GTDB-Tk package v1.3 (20), and genes of 16 ribosomal proteins proposed by Hug et al. (19) and 57 single-copy genes proposed by Dombrowski et al. (21), identified based on the gene annotation performed previously (see “Gene annotation” part). The marker genes are listed in Tables S13–15. In addition, 21 single-copy genes were selected for the phylogenetic analysis of Korarchaeota, and 37 marker genes were refined for the phylogenetic analysis of Archaea (see “Determination of marker genes for phylogeny construction” section). To analyze the evolution of carbon monoxide dehydrogenase/acetyl-CoA synthase, methyl-coenzyme M reductase, and the selected marker genes mentioned in the “Determination of marker genes for phylogeny construction” section, the amino acid sequences for the corresponding genes extracted from the korarchaeal MAGs and reference genomes [including those from Adam et al. (72)] were analyzed.

The 16S rRNA gene sequences were aligned using SINA v1.2.11 against the SILVA SSU database v138 (73). The amino acid sequences corresponding to the marker genes and the functional genes mentioned above were aligned using muscle v3.8.31 (74). Columns with more than 5% gaps were trimmed using trimAl v1.4 (75). Then, for multiple-sequence phylogeny, the aligned sequences were concatenated. All maximum-likelihood trees were built using IQ-TREE v2.1.3 (76) with the best-fit models selected by ModelFinder function. Specifically, for the amino acid sequences, mixture models (C10-C60 models) were involved in model selection process by applying “-madd” flag. The number of bootstraps was 100. All Bayesian tree inferences were analyzed by using Phylobayes-mpi v1.8 (77) with GTR + CAT model, and all runs were calculated until the maxdiff value was <0.1 or more than 10,000 rounds have been calculated. All the phylogenetic trees were visualized using the iTOL web server (78).

### Genomic comparisons and species divergence time estimation

Multidimensional scaling analysis of Korarchaeota homologous genes was performed using phylogenetic hierarchical orthogroup table (obtained as described in the “Determination of marker genes for phylogeny construction” section) and vegan package (67).

Gene-level events, such as duplication, transfer, and loss, were estimated using 283 high-quality MAGs (completeness >90%; for Korarchaeota and DPANN archaea, completeness >85%; listed in Table S10) by using ALE algorithm, as recommended by Szöllõsi et al. (41). Briefly, samples of 10,000 trees of the selected gene families (listed in Table S11) and the species tree were obtained by using phylobayes-mpi v1.8 (77), as described in the “Phylogenetic analysis” section. They were then reconciled by performing the maximum-likelihood implementation of the undated ALE algorithm (79) to estimate the evolutionary events for the genes.

Species divergence time estimation was based on the phylogenetic tree of 283 high-quality MAGs described above. The analysis was conducted by using MCMCTree v4.9j (80) with several temporal calibrations, as described previously (45): the root of Archaea of approximately 4,380–3,460 Mya (81); Thermoproteales and Sulfolobales both originating after the GOE (approximately 2,330 Mya) (82, 83); and most recent common ancestor of *S. solfataricus* and *S. islandicus* originating later than 475 Mya (84). The general method in MCMCTree v4.9j (80) was implemented, as follows. First, CodeML module was used to calculate the overall substitution rate for each gene family. Then, mcmctree module (with usedata = 3) was used to calculate the MLE of branch length, gradient, and Hessian (85). Finally, the mcmctree module (with usedata = 2) was repeated to estimate the divergence time. The parameters of mcmctree module were “burnin = 20000, sampfreq = 10, nsample = 200000.” The full results are shown in Fig. S17. The convergence of results was checked by plotting the posterior mean times from two independent analysis runs, as shown in Fig. S18.

## Data Availability

The raw sequence data are available at the National Center for Biotechnology Information (NCBI) under BioProject ID PRJNA479337. The sequences of Korarchaeota MAG Yap.int.bin1.1 have been deposited in NCBI under Genome ID JAMKQM000000000 and in eLMSG (an eLibrary of Microbial Systematics and Genomics) under the accession numbers LMSG_G000001524.1.

## SUPPLEMENTAL MATERIAL

Supplemental figures msystems.00305-23-s0001.pdf Figures S1, S2, S4 to S12, S14, and S16 to S18.

Figure S3 msystems.00305-23-s0002.pdf Phylogenetic trees constructed using 115 single-copy genes selected from among homologous genes in korarchaeal MAGs.

Figure S13 msystems.00305-23-s0003.pdf Phylogenetic trees based on 51 archaeal marker genes.

Figure S15 msystems.00305-23-s0004.pdf Phylogenetic trees constructed using concatenated sequences of the *cdhABCDE* genes and those based on single genes.

Legends msystems.00305-23-s0005.docx Legends to the supplemental material.

Supplemental tables msystems.00305-23-s0006.xlsx Tables S1 to S15.

ASM does not own the copyrights to Supplemental Material that may be linked to, or accessed through, an article. The authors have granted ASM a non-exclusive, world-wide license to publish the Supplemental Material files. Please contact the corresponding author directly for reuse.
