# Supplementary material for: Wood–Ljungdahl pathway found in novel marine Korarchaeota groups illuminates their evolutionary history
Source: mSystems. 2023 Jul 17;8(4):e00305-23. doi: 10.1128/msystems.00305-23 (PMC10469681; doi:10.1128/msystems.00305-23)
Supplement: Supplemental figures — Figures S1, S2, S4 to S12, S14, and S16 to S18. [file msystems.00305-23-s0001.pdf]

Figure S1

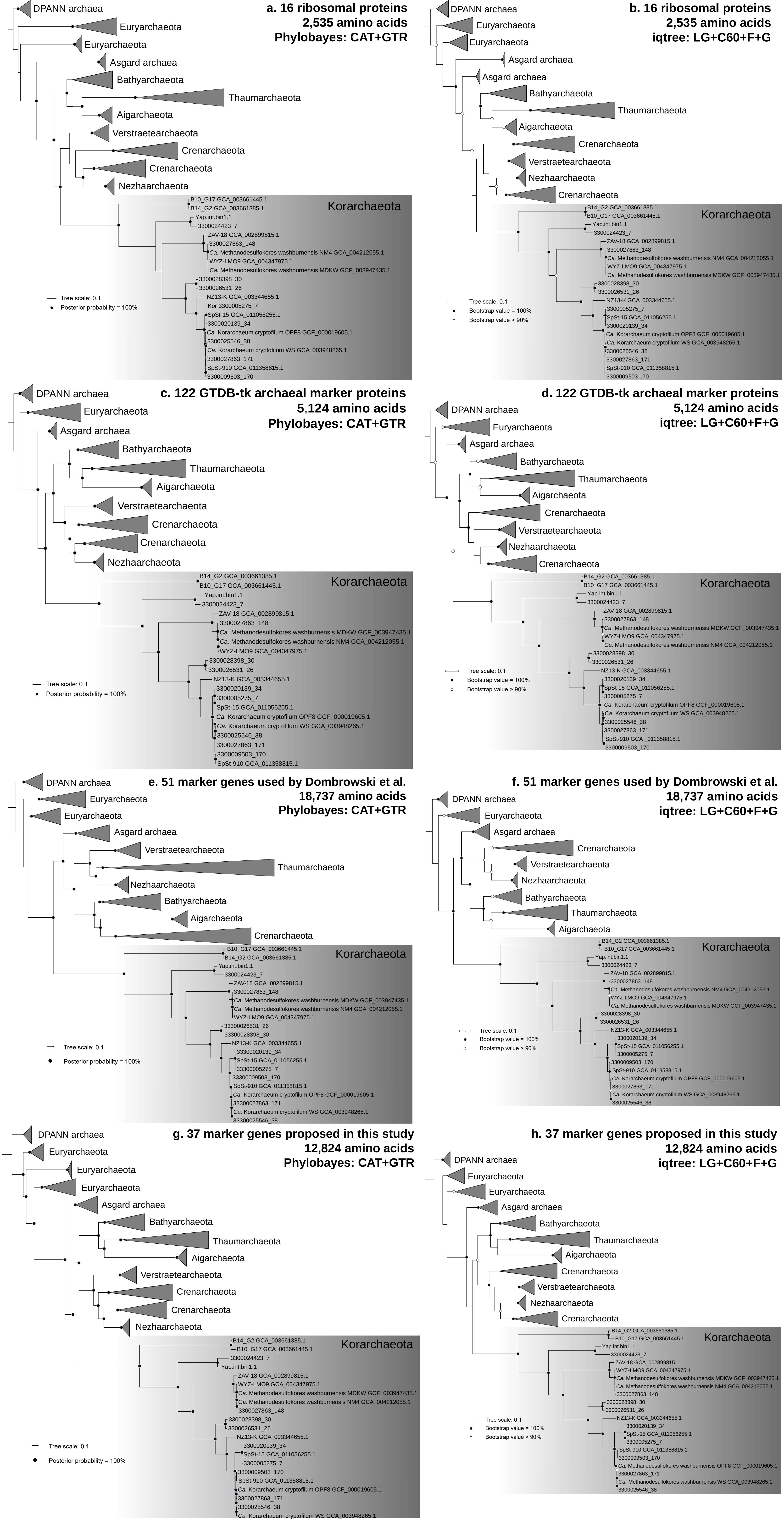

a. 16 ribosomal proteins
2,535 amino acids
Phylobayes: CAT+GTR
DPANN archaea
Euryarchaeota
Euryarchaeota
Asgard archaea
Bathyarchaeota
Thaumarchaeota
Aigarchaeota
Verstraetearchaeota
Crenarchaeota
Crenarchaeota
Nezhaarchaeota
Korarchaeota
B10_G17 GCA_003661445.1
B14_G2 GCA_003661385.1
Yap.int.bin1.1
3300024423_7
ZAV-18 GCA_002899815.1
3300027863_148
Ca. Methanodesulfokores washburnensis NM4 GCA_004212055.1
WYZ-LMO9 GCA_004347975.1
Ca. Methanodesulfokores washburnensis MDKW GCF_003947435.1
3300028398_30
3300026531_26
NZ13-K GCA_003344655.1
Kor 3300005275_7
SpSt-15 GCA_011056255.1
3300020139_34
Ca. Korarchaeum cryptofilum OPF8 GCF_000019605.1
3300025546_38
Ca. Korarchaeum cryptofilum WS GCA_003948265.1
3300027863_171
SpSt-910 GCA_011358815.1
3300009503_170
Tree scale: 0.1
Posterior probability = 100%
b. 16 ribosomal proteins
2,535 amino acids
iqtree: LG+C60+F+G
DPANN archaea
Euryarchaeota
Euryarchaeota
Asgard archaea
Asgard archaea
Bathyarchaeota
Thaumarchaeota
Aigarchaeota
Crenarchaeota
Verstraetearchaeota
Nezhaarchaeota
Crenarchaeota
Korarchaeota
B14_G2 GCA_003661385.1
B10_G17 GCA_003661445.1
Yap.int.bin1.1
3300024423_7
ZAV-18 GCA_002899815.1
3300027863_148
Ca. Methanodesulfokores washburnensis NM4 GCA_004212055.1
WYZ-LMO9 GCA_004347975.1
Ca. Methanodesulfokores washburnensis MDKW GCF_003947435.1
3300028398_30
3300026531_26
NZ13-K GCA_003344655.1
3300005275_7
SpSt-15 GCA_011056255.1
3300020139_34
Ca. Korarchaeum cryptofilum OPF8 GCF_000019605.1
Ca. Korarchaeum cryptofilum WS GCA_003948265.1
3300025546_38
3300027863_171
SpSt-910 GCA_011358815.1
3300009503_170
Tree scale: 0.1
Bootstrap value = 100%
Bootstrap value > 90%
c. 122 GTDB-tk archaeal marker proteins
5,124 amino acids
Phylobayes: CAT+GTR
DPANN archaea
Euryarchaeota
Asgard archaea
Bathyarchaeota
Thaumarchaeota
Aigarchaeota
Verstraetearchaeota
Crenarchaeota
Crenarchaeota
Nezhaarchaeota
Korarchaeota
B14_G2 GCA_003661385.1
B10_G17 GCA_003661445.1
Yap.int.bin1.1
3300024423_7
ZAV-18 GCA_002899815.1
3300027863_148
Ca. Methanodesulfokores washburnensis MDKW GCF_003947435.1
Ca. Methanodesulfokores washburnensis NM4 GCA_004212055.1
WYZ-LMO9 GCA_004347975.1
3300028398_30
3300026531_26
NZ13-K GCA_003344655.1
3300020139_34
3300005275_7
SpSt-15 GCA_011056255.1
Ca. Korarchaeum cryptofilum OPF8 GCF_000019605.1
Ca. Korarchaeum cryptofilum WS GCA_003948265.1
3300025546_38
3300027863_171
3300009503_170
SpSt-910 GCA_011358815.1
Tree scale: 0.1
Posterior probability = 100%
d. 122 GTDB-tk archaeal marker proteins
5,124 amino acids
iqtree: LG+C60+F+G
DPANN archaea
Euryarchaeota
Asgard archaea
Bathyarchaeota
Thaumarchaeota
Aigarchaeota
Crenarchaeota
Verstraetearchaeota
Nezhaarchaeota
Crenarchaeota
Korarchaeota
B14_G2 GCA_003661385.1
B10_G17 GCA_003661445.1
Yap.int.bin1.1
3300024423_7
ZAV-18 GCA_002899815.1
3300027863_148
Ca. Methanodesulfokores washburnensis MDKW GCF_003947435.1
WYZ-LMO9 GCA_004347975.1
Ca. Methanodesulfokores washburnensis NM4 GCA_004212055.1
3300028398_30
3300026531_26
NZ13-K GCA_003344655.1
3300020139_34
SpSt-15 GCA_011056255.1
3300005275_7
Ca. Korarchaeum cryptofilum OPF8 GCF_000019605.1
Ca. Korarchaeum cryptofilum WS GCA_003948265.1
3300025546_38
3300027863_171
SpSt-910 GCA_011358815.1
3300009503_170
Tree scale: 0.1
Bootstrap value = 100%
Bootstrap value > 90%
e. 51 marker genes used by Dombrowski et al.
18,737 amino acids
Phylobayes: CAT+GTR
DPANN archaea
Euryarchaeota
Euryarchaeota
Asgard archaea
Verstraetearchaeota
Thaumarchaeota
Nezhaarchaeota
Bathyarchaeota
Aigarchaeota
Crenarchaeota
Korarchaeota
B10_G17 GCA_003661445.1
B14_G2 GCA_003661385.1
Yap.int.bin1.1
3300024423_7
ZAV-18 GCA_002899815.1
3300027863_148
Ca. Methanodesulfokores washburnensis MDKW GCF_003947435.1
Ca. Methanodesulfokores washburnensis NM4 GCA_004212055.1
WYZ-LMO9 GCA_004347975.1
33300026531_26
33300028398_30
NZ13-K GCA_003344655.1
33300020139_34
SpSt-15 GCA_011056255.1
3300005275_7
33300009503_170
SpSt-910 GCA_011358815.1
Ca. Korarchaeum cryptofilum OPF8 GCF_000019605.1
33300027863_171
Ca. Korarchaeum cryptofilum WS GCA_003948265.1
33300025546_38
Tree scale: 0.1
Posterior probability = 100%
f. 51 marker genes used by Dombrowski et al.
18,737 amino acids
iqtree: LG+C60+F+G
DPANN archaea
Euryarchaeota
Asgard archaea
Crenarchaeota
Verstraetearchaeota
Nezhaarchaeota
Bathyarchaeota
Thaumarchaeota
Aigarchaeota
Korarchaeota
B14_G2 GCA_003661385.1
B10_G17 GCA_003661445.1
Yap.int.bin1.1
3300024423_7
ZAV-18 GCA_002899815.1
3300027863_148
Ca. Methanodesulfokores washburnensis NM4 GCA_004212055.1
WYZ-LMO9 GCA_004347975.1
Ca. Methanodesulfokores washburnensis MDKW GCF_003947435.1
3300028398_30
3300026531_26
NZ13-K GCA_003344655.1
3300020139_34
SpSt-15 GCA_011056255.1
3300005275_7
3300009503_170
SpSt-910 GCA_011358815.1
Ca. Korarchaeum cryptofilum OPF8 GCF_000019605.1
3300027863_171
Ca. Korarchaeum cryptofilum WS GCA_003948265.1
3300025546_38
Tree scale: 0.1
Bootstrap value = 100%
Bootstrap value > 90%
g. 37 marker genes proposed in this study
12,824 amino acids
Phylobayes: CAT+GTR
DPANN archaea
Euryarchaeota
Euryarchaeota
Euryarchaeota
Asgard archaea
Bathyarchaeota
Thaumarchaeota
Aigarchaeota
Verstraetearchaeota
Crenarchaeota
Crenarchaeota
Nezhaarchaeota
Korarchaeota
B14_G2 GCA_003661385.1
B10_G17 GCA_003661445.1
3300024423_7
Yap.int.bin1.1
ZAV-18 GCA_002899815.1
WYZ-LMO9 GCA_004347975.1
Ca. Methanodesulfokores washburnensis MDKW GCF_003947435.1
Ca. Methanodesulfokores washburnensis NM4 GCA_004212055.1
3300027863_148
3300028398_30
3300026531_26
NZ13-K GCA_003344655.1
3300020139_34
SpSt-15 GCA_011056255.1
3300005275_7
3300009503_170
SpSt-910 GCA_011358815.1
Ca. Korarchaeum cryptofilum OPF8 GCF_000019605.1
3300027863_171
3300025546_38
Ca. Korarchaeum cryptofilum WS GCA_003948265.1
Tree scale: 0.1
Posterior probability = 100%
h. 37 marker genes proposed in this study
12,824 amino acids
iqtree: LG+C60+F+G
DPANN archaea
Euryarchaeota
Euryarchaeota
Asgard archaea
Bathyarchaeota
Thaumarchaeota
Aigarchaeota
Crenarchaeota
Verstraetearchaeota
Nezhaarchaeota
Crenarchaeota
Korarchaeota
B14_G2 GCA_003661385.1
B10_G17 GCA_003661445.1
Yap.int.bin1.1
3300024423_7
ZAV-18 GCA_002899815.1
WYZ-LMO9 GCA_004347975.1
Ca. Methanodesulfokores washburnensis MDKW GCF_003947435.1
Ca. Methanodesulfokores washburnensis NM4 GCA_004212055.1
3300027863_148
3300028398_30
3300026531_26
NZ13-K GCA_003344655.1
3300020139_34
SpSt-15 GCA_011056255.1
3300005275_7
SpSt-910 GCA_011358815.1
3300009503_170
Ca. Methanodesulfokores washburnensis OPF8 GCF_000019605.1
3300027863_171
Ca. Methanodesulfokores washburnensis WS GCA_003948265.1
3300025546_38
Tree scale: 0.1
Bootstrap value = 100%
Bootstrap value > 90%

Figure S2

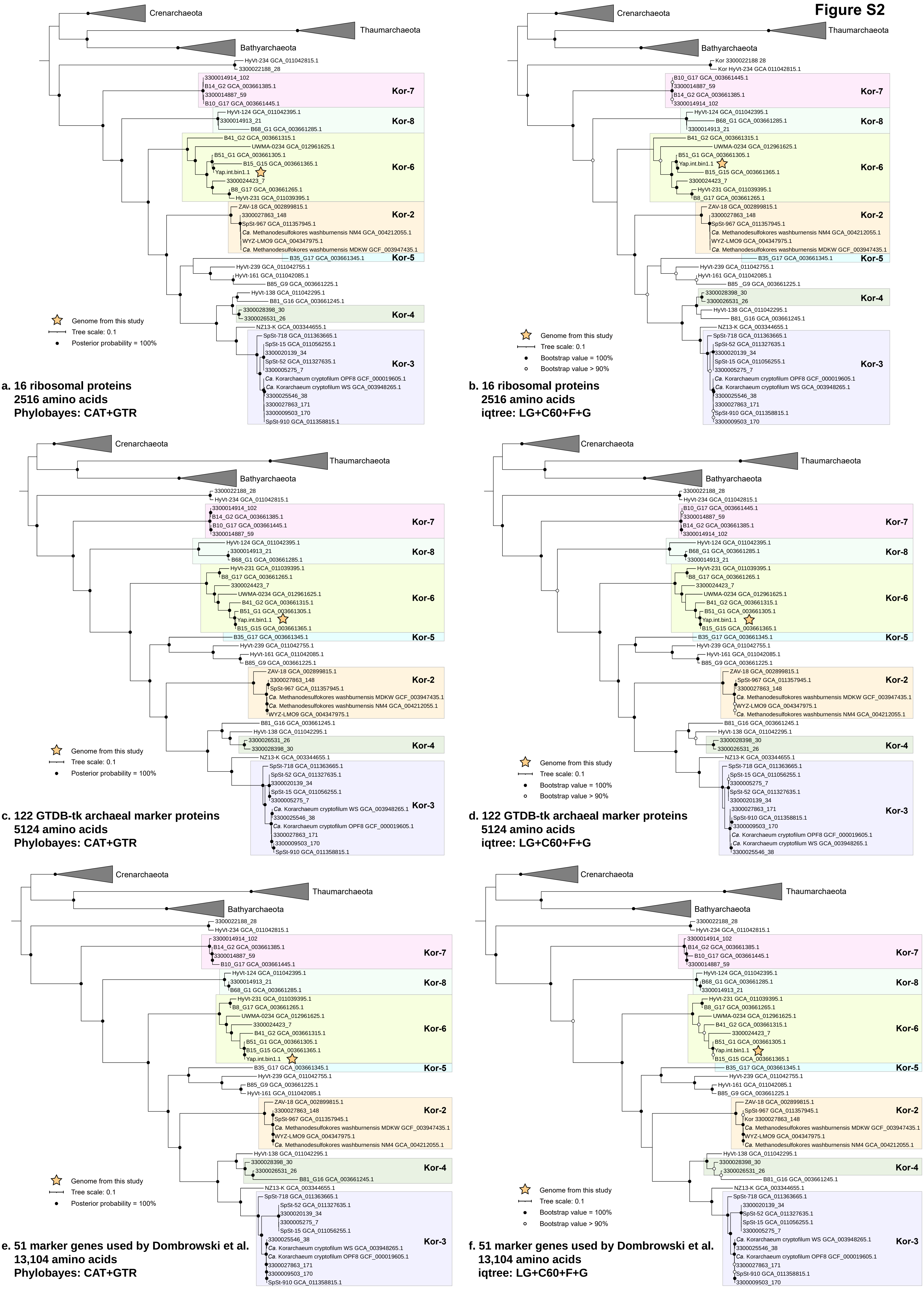
Crenarchaeota
Thaumarchaeota
Bathyarchaeota
HyVt-234 GCA_011042815.1
3300022188_28
3300014914_102
B14_G2 GCA_003661385.1
3300014887_59
B10_G17 GCA_003661445.1
Kor-7
HyVt-124 GCA_011042395.1
3300014913_21
B68_G1 GCA_003661285.1
Kor-8
B41_G2 GCA_003661315.1
UWMA-0234 GCA_012961625.1
B51_G1 GCA_003661305.1
B15_G15 GCA_003661365.1
Yap.int.bin1.1
3300024423_7
B8_G17 GCA_003661265.1
HyVt-231 GCA_011039395.1
Kor-6
ZAV-18 GCA_002899815.1
3300027863_148
SpSt-967 GCA_011357945.1
Ca. Methanodesulfokores washburnensis NM4 GCA_004212055.1
WYZ-LMO9 GCA_004347975.1
Ca. Methanodesulfokores washburnensis MDKW GCF_003947435.1
Kor-2
B35_G17 GCA_003661345.1
Kor-5
HyVt-239 GCA_011042755.1
HyVt-161 GCA_011042085.1
B85_G9 GCA_003661225.1
HyVt-138 GCA_011042295.1
B81_G16 GCA_003661245.1
3300028398_30
3300026531_26
Kor-4
NZ13-K GCA_003344655.1
SpSt-718 GCA_011363665.1
SpSt-15 GCA_011056255.1
3300020139_34
SpSt-52 GCA_011327635.1
3300005275_7
Ca. Korarchaeum cryptofilum OPF8 GCF_000019605.1
Ca. Korarchaeum cryptofilum WS GCA_003948265.1
3300025546_38
3300027863_171
3300009503_170
SpSt-910 GCA_011358815.1
Kor-3
Genome from this study
Tree scale: 0.1
Posterior probability = 100%
a. 16 ribosomal proteins
2516 amino acids
Phylobayes: CAT+GTR
Kor 3300022188 28
Kor HyVt-234 GCA_011042815.1
Kor 3300027863_148
Bootstrap value = 100%
Bootstrap value > 90%
b. 16 ribosomal proteins
2516 amino acids
iqtree: LG+C60+F+G
c. 122 GTDB-tk archaeal marker proteins
5124 amino acids
Phylobayes: CAT+GTR
d. 122 GTDB-tk archaeal marker proteins
5124 amino acids
iqtree: LG+C60+F+G
e. 51 marker genes used by Dombrowski et al.
13,104 amino acids
Phylobayes: CAT+GTR
f. 51 marker genes used by Dombrowski et al.
13,104 amino acids
iqtree: LG+C60+F+G

Figure S4

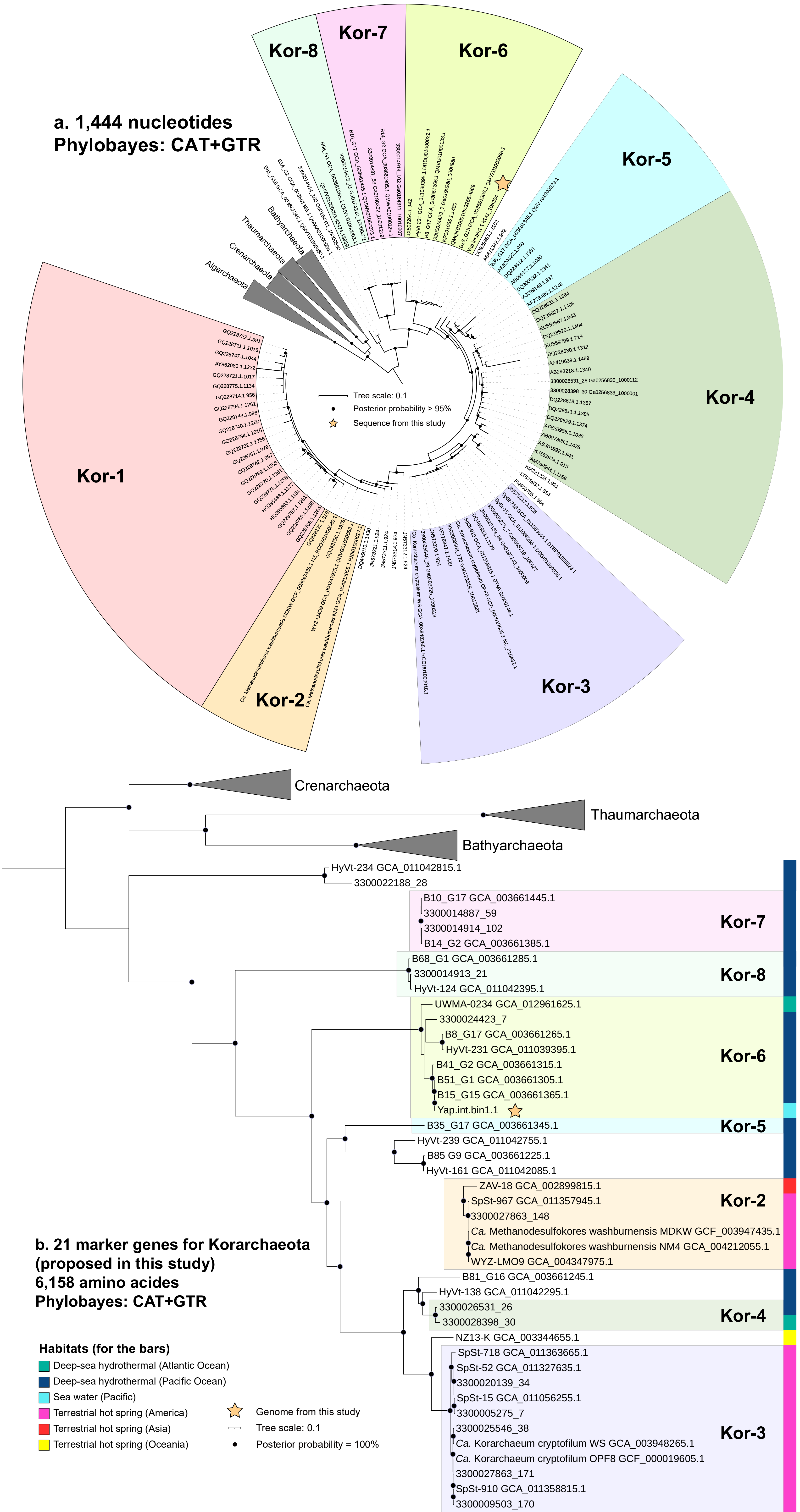

a. 1,444 nucleotides
Phylobayes: CAT+GTR
Kor-8
Kor-7
Kor-6
Kor-5
Kor-4
Kor-3
Kor-2
Kor-1
Bathyarchaeota
Thaumarchaeota
Crenarchaeota
Aigarchaeota
Tree scale: 0.1
Posterior probability > 95%
Sequence from this study
Crenarchaeota
Thaumarchaeota
Bathyarchaeota
HyVt-234 GCA_011042815.1
3300022188_28
B10_G17 GCA_003661445.1
3300014887_59
3300014914_102
B14_G2 GCA_003661385.1
Kor-7
B68_G1 GCA_003661285.1
3300014913_21
HyVt-124 GCA_011042395.1
Kor-8
UWMA-0234 GCA_012961625.1
3300024423_7
B8_G17 GCA_003661265.1
HyVt-231 GCA_011039395.1
B41_G2 GCA_003661315.1
B51_G1 GCA_003661305.1
B15_G15 GCA_003661365.1
Yap.int.bin1.1
Kor-6
B35_G17 GCA_003661345.1
Kor-5
HyVt-239 GCA_011042755.1
B85 G9 GCA_003661225.1
HyVt-161 GCA_011042085.1
ZAV-18 GCA_002899815.1
SpSt-967 GCA_011357945.1
3300027863_148
Ca. Methanodesulfokores washburnensis MDKW GCF_003947435.1
Ca. Methanodesulfokores washburnensis NM4 GCA_004212055.1
WYZ-LMO9 GCA_004347975.1
Kor-2
B81_G16 GCA_003661245.1
HyVt-138 GCA_011042295.1
3300026531_26
3300028398_30
Kor-4
NZ13-K GCA_003344655.1
SpSt-718 GCA_011363665.1
SpSt-52 GCA_011327635.1
3300020139_34
SpSt-15 GCA_011056255.1
3300005275_7
3300025546_38
Ca. Korarchaeum cryptofilum WS GCA_003948265.1
Ca. Korarchaeum cryptofilum OPF8 GCF_000019605.1
3300027863_171
SpSt-910 GCA_011358815.1
3300009503_170
Kor-3
b. 21 marker genes for Korarchaeota
(proposed in this study)
6,158 amino acides
Phylobayes: CAT+GTR
Habitats (for the bars)
Deep-sea hydrothermal (Atlantic Ocean)
Deep-sea hydrothermal (Pacific Ocean)
Sea water (Pacific)
Terrestrial hot spring (America)
Terrestrial hot spring (Asia)
Terrestrial hot spring (Oceania)
Genome from this study
Tree scale: 0.1
Posterior probability = 100%

Figure S5

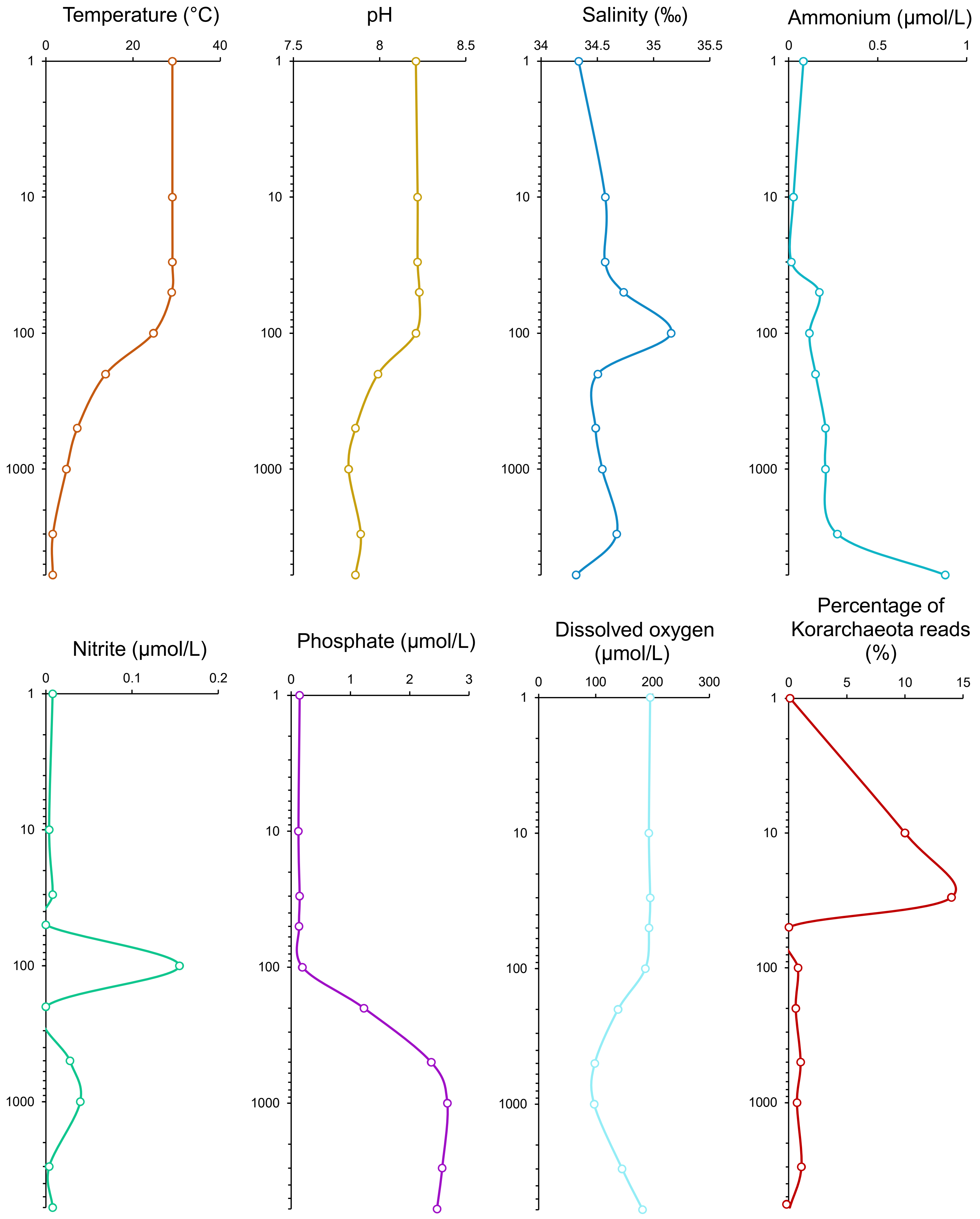

Temperature (°C)
pH
Salinity (‰)
Ammonium (μmol/L)
Nitrite (μmol/L)
Phosphate (μmol/L)
Dissolved oxygen (μmol/L)
Percentage of Korarchaeota reads (%)

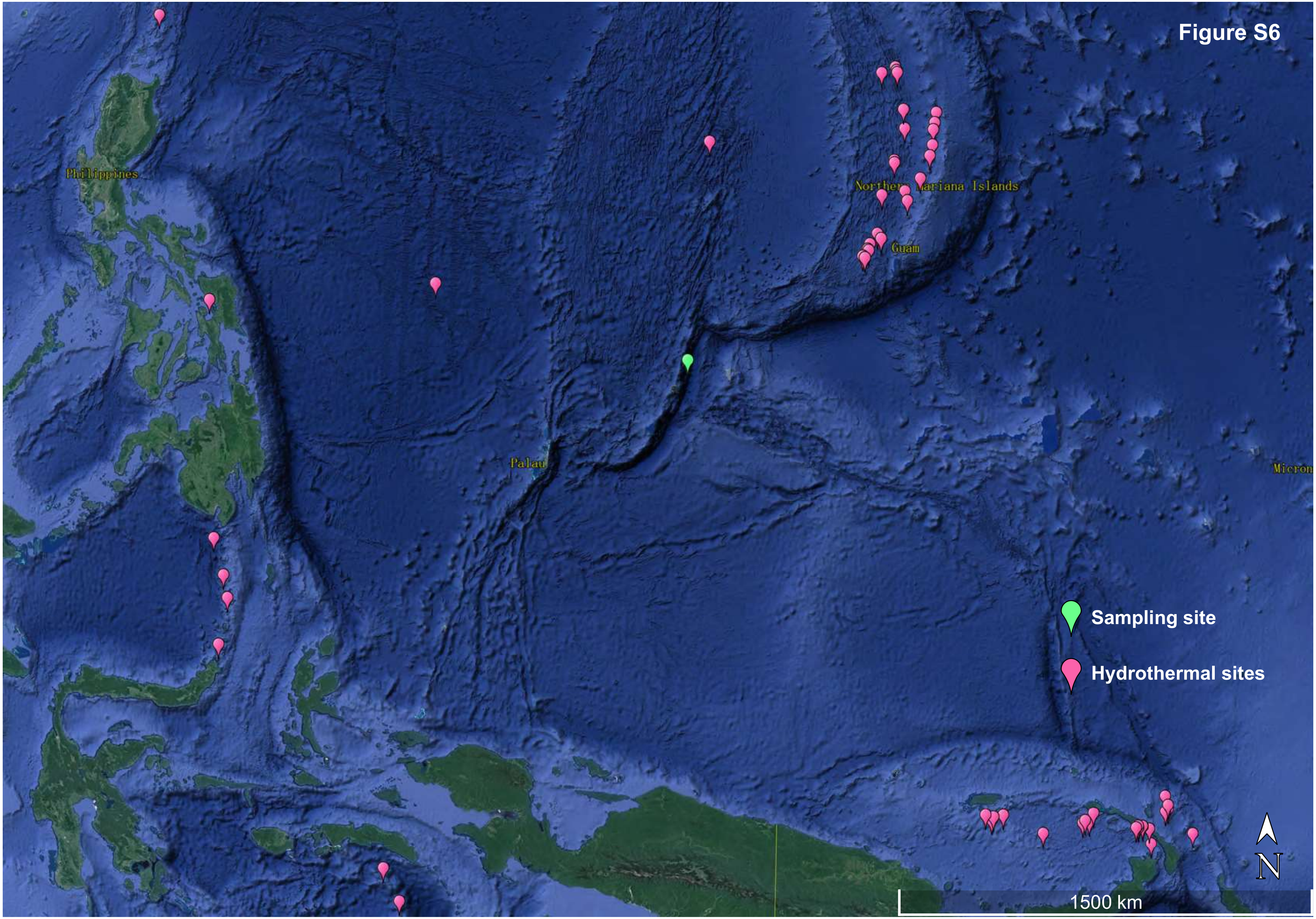

Figure S6
Philippines
Northern Mariana Islands
Guam
Palau
Micron
Sampling site
Hydrothermal sites
N
1500 km

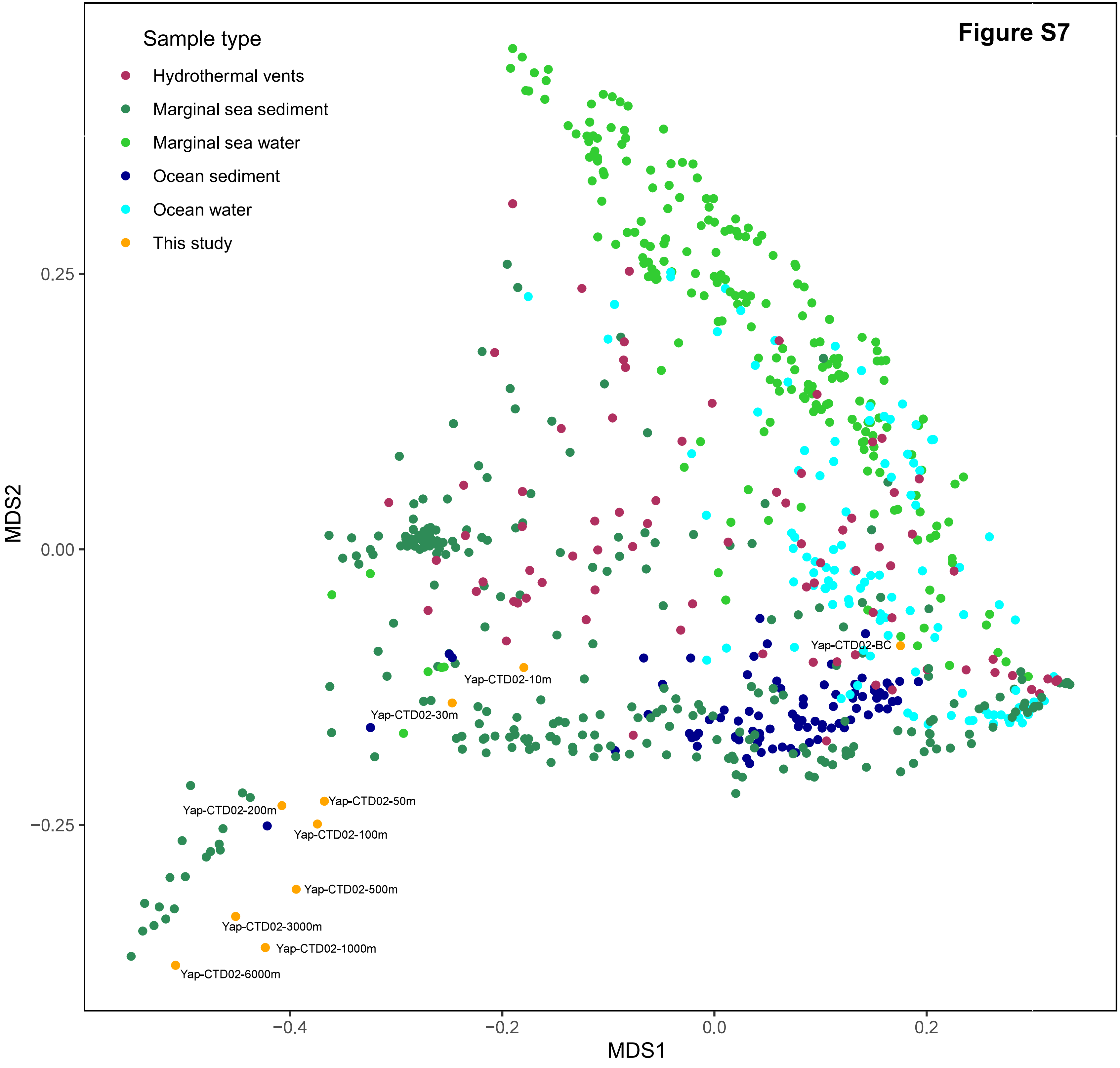
Figure S7
Sample type
Hydrothermal vents
Marginal sea sediment
Marginal sea water
Ocean sediment
Ocean water
This study
Yap-CTD02-BC
Yap-CTD02-10m
Yap-CTD02-30m
Yap-CTD02-50m
Yap-CTD02-200m
Yap-CTD02-100m
Yap-CTD02-500m
Yap-CTD02-3000m
Yap-CTD02-1000m
Yap-CTD02-6000m
MDS2
0.25
0.00
−0.25
MDS1
−0.4
−0.2
0.0
0.2

Figure S8

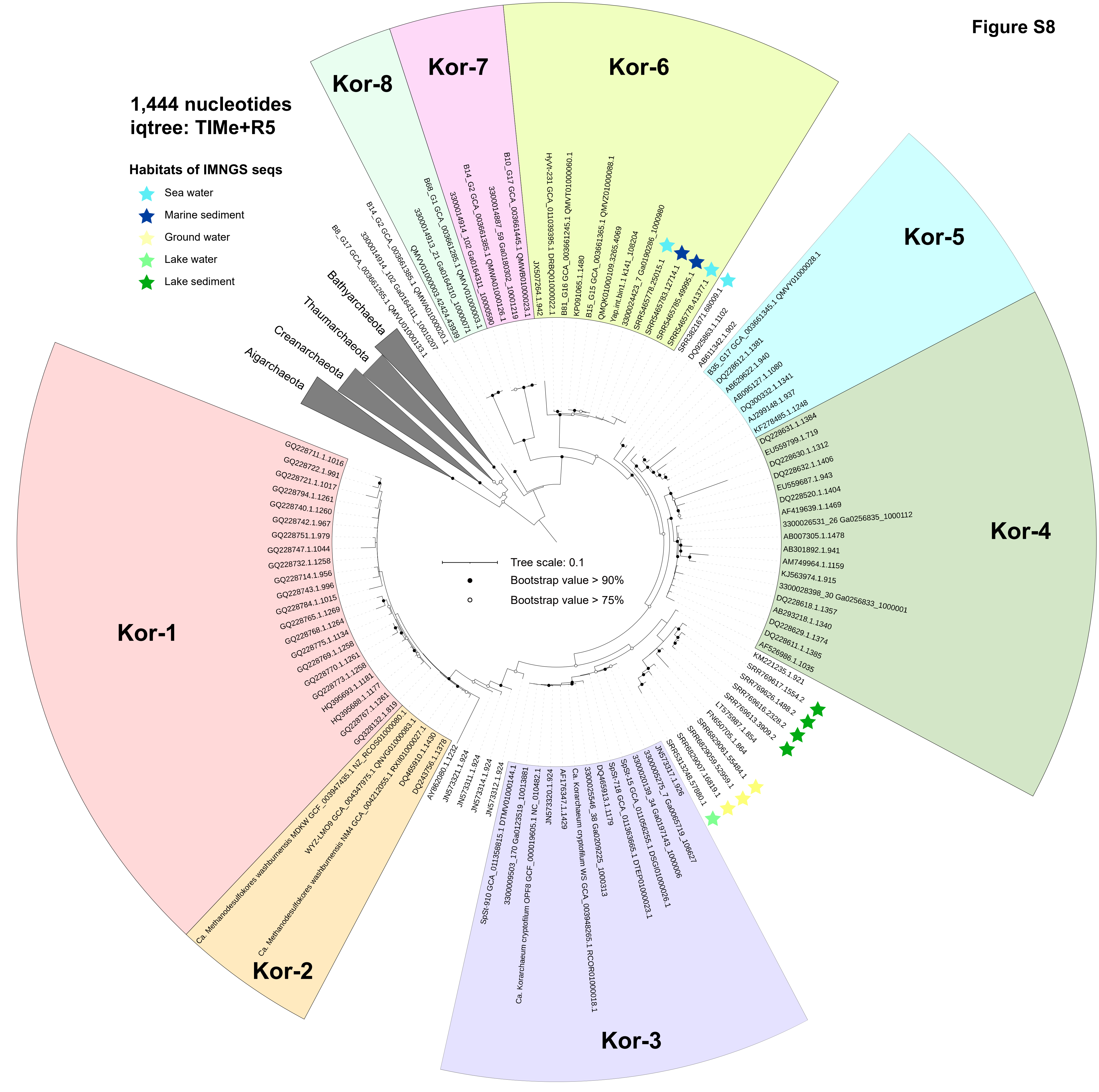
1,444 nucleotides
iqtree: TIMe+R5
Habitats of IMNGS seqs
Sea water
Marine sediment
Ground water
Lake water
Lake sediment
Kor-1
Kor-2
Kor-3
Kor-4
Kor-5
Kor-6
Kor-7
Kor-8
Bathyarchaeota
Thaumarchaeota
Creanarchaeota
Aigarchaeota
Tree scale: 0.1
Bootstrap value > 90%
Bootstrap value > 75%
GQ228711.1.1016
GQ228722.1.991
GQ228721.1.1017
GQ228794.1.1261
GQ228740.1.1260
GQ228742.1.967
GQ228751.1.979
GQ228747.1.1044
GQ228732.1.1258
GQ228714.1.956
GQ228743.1.996
GQ228784.1.1015
GQ228765.1.1269
GQ228768.1.1264
GQ228775.1.1134
GQ228769.1.1258
GQ228770.1.1261
GQ228773.1.1258
HQ395693.1.1181
HQ395688.1.1177
GQ228767.1.1261
GQ328132.1.819
Ca. Methanodesulfokores washburnensis MDKW GCF_003947435.1 NZ_RCOS01000080.1
WYZ-LMO9 GCA_004347975.1 QNVG01000083.1
Ca. Methanodesulfokores washburnensis NM4 GCA_004212055.1 RXII01000027.1
DQ465910.1.1430
DQ243756.1.1378
AY862080.1.1232
JN573321.1.924
JN573311.1.924
JN573314.1.924
JN573312.1.924
SpSt-910 GCA_011358815.1 DTMV01000144.1
3300009503_170 Ga0123519_10013881
Ca. Korarchaeum cryptofilum OPF8 GCF_000019605.1 NC_010482.1
JN573320.1.924
AF176347.1.1429
Ca. Korarchaeum cryptofilum WS GCA_003948265.1 RCOR01000018.1
3300025546_38 Ga0209225_1000313
DQ465913.1.1179
SpSt-718 GCA_011363665.1 DTEP01000023.1
SpSt-15 GCA_011056255.1 DSGI01000026.1
3300020139_34 Ga0197143_1000006
3300005275_7 Ga0065719_106627
JN573317.1.926
SRR5313248.57880.1
SRR6829007.16819.1
SRR6829059.52959.1
SRR6829061.55484.1
FN650705.1.864
LT575987.1.854
SRR769613.3909.2
SRR769616.2328.2
SRR769626.1488.2
SRR769617.1554.2
KM221235.1.921
AF526986.1.1035
DQ228611.1.1385
DQ228629.1.1374
AB293218.1.1340
DQ228618.1.1357
3300028398_30 Ga0256833_1000001
KJ563974.1.915
AM749964.1.1159
AB301892.1.941
AB007305.1.1478
3300026531_26 Ga0256835_1000112
AF419639.1.1469
DQ228520.1.1404
EU559687.1.943
DQ228632.1.1406
DQ228630.1.1312
EU559799.1.719
DQ228631.1.1384
KF278485.1.1248
AJ299148.1.937
DQ300332.1.1341
AB095127.1.1080
AB629622.1.940
DQ228612.1.1381
B35_G17 GCA_003661345.1 QMVY01000028.1
AB611342.1.902
DQ925863.1.1102
SRR3821671.68009.1
SRR5465778.41377.1
SRR5465785.49995.1
SRR5465783.12714.1
SRR5465778.25015.1
3300024423_7 Ga0190286_1000980
Yap.int.bin1.1 k141_108204
QMQK01000109.3265.4069
B15_G15 GCA_003661365.1 QMVZ01000088.1
KP091065.1.1480
B81_G16 GCA_003661245.1 QMVT01000060.1
HyVt-231 GCA_011039395.1 DRBQ01000022.1
JX507264.1.942
B10_G17 GCA_003661445.1 QMWB01000023.1
3300014887_59 Ga0180302_10001219
B14_G2 GCA_003661385.1 QMWA01000126.1
3300014914_102 Ga0164311_10000590
B68_G1 GCA_003661285.1 QMVV01000003.1
3300014913_21 Ga0164310_10000071
QMVV01000003.42424.43939
B14_G2 GCA_003661385.1 QMWA01000020.1
3300014914_102 Ga0164311_10010207
B8_G17 GCA_003661265.1 QMVU01000133.1

Figure S9

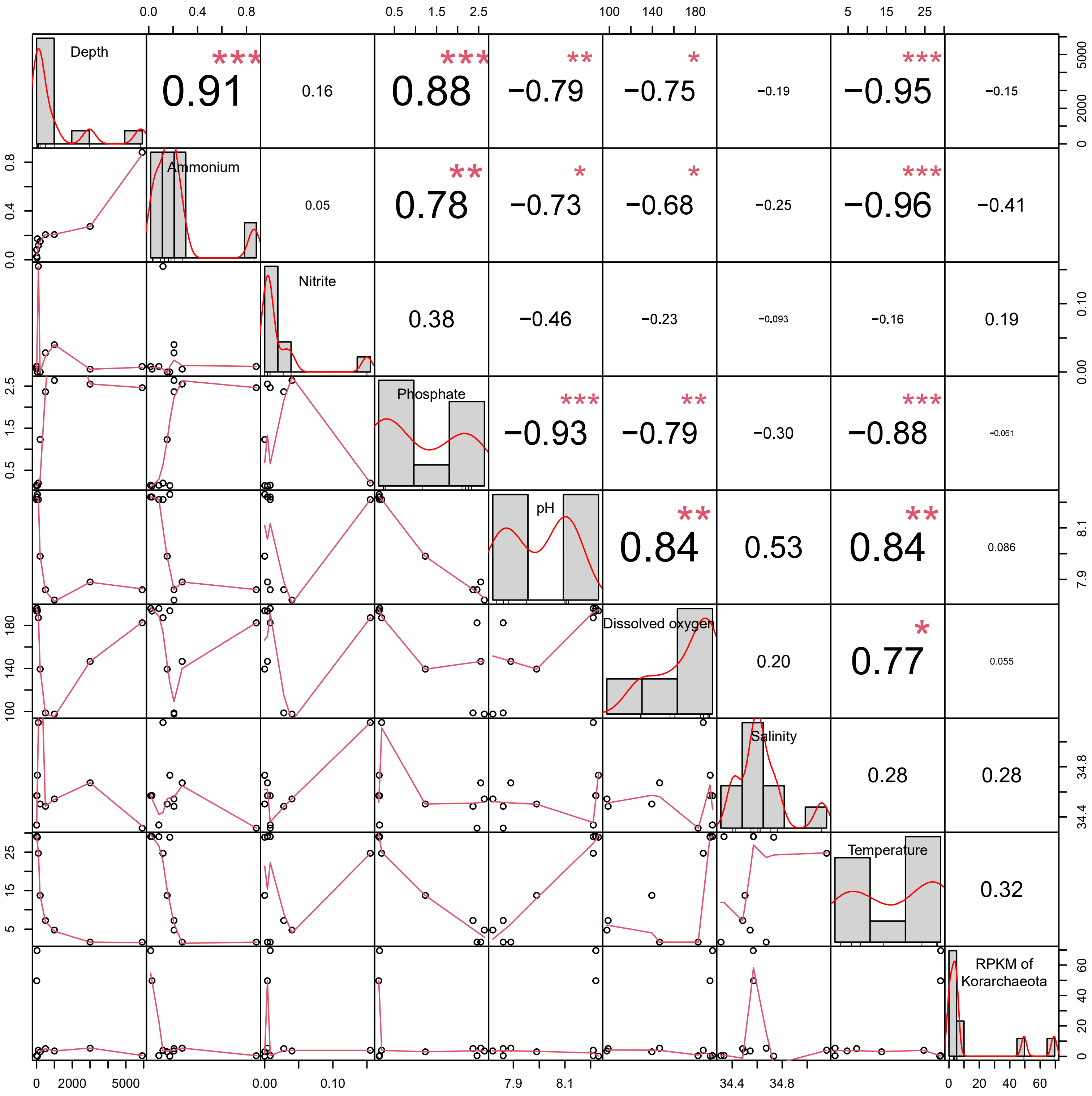

* $P < 0.05$ ** $P < 0.01$ *** $P < 0.001$

Figure S10

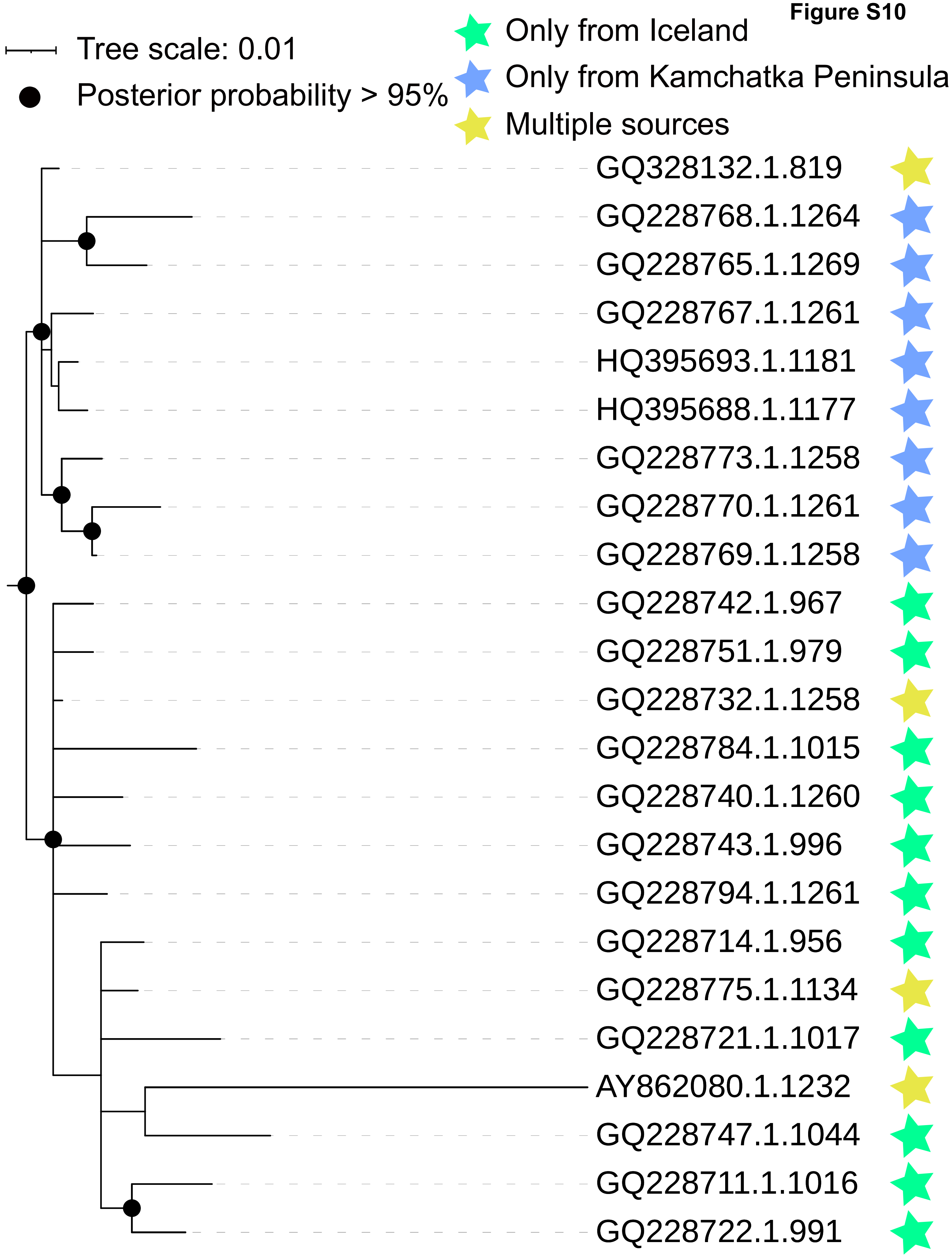
Tree scale: 0.01
Posterior probability > 95%
Only from Iceland
Only from Kamchatka Peninsula
Multiple sources
GQ328132.1.819
GQ228768.1.1264
GQ228765.1.1269
GQ228767.1.1261
HQ395693.1.1181
HQ395688.1.1177
GQ228773.1.1258
GQ228770.1.1261
GQ228769.1.1258
GQ228742.1.967
GQ228751.1.979
GQ228732.1.1258
GQ228784.1.1015
GQ228740.1.1260
GQ228743.1.996
GQ228794.1.1261
GQ228714.1.956
GQ228775.1.1134
GQ228721.1.1017
AY862080.1.1232
GQ228747.1.1044
GQ228711.1.1016
GQ228722.1.991

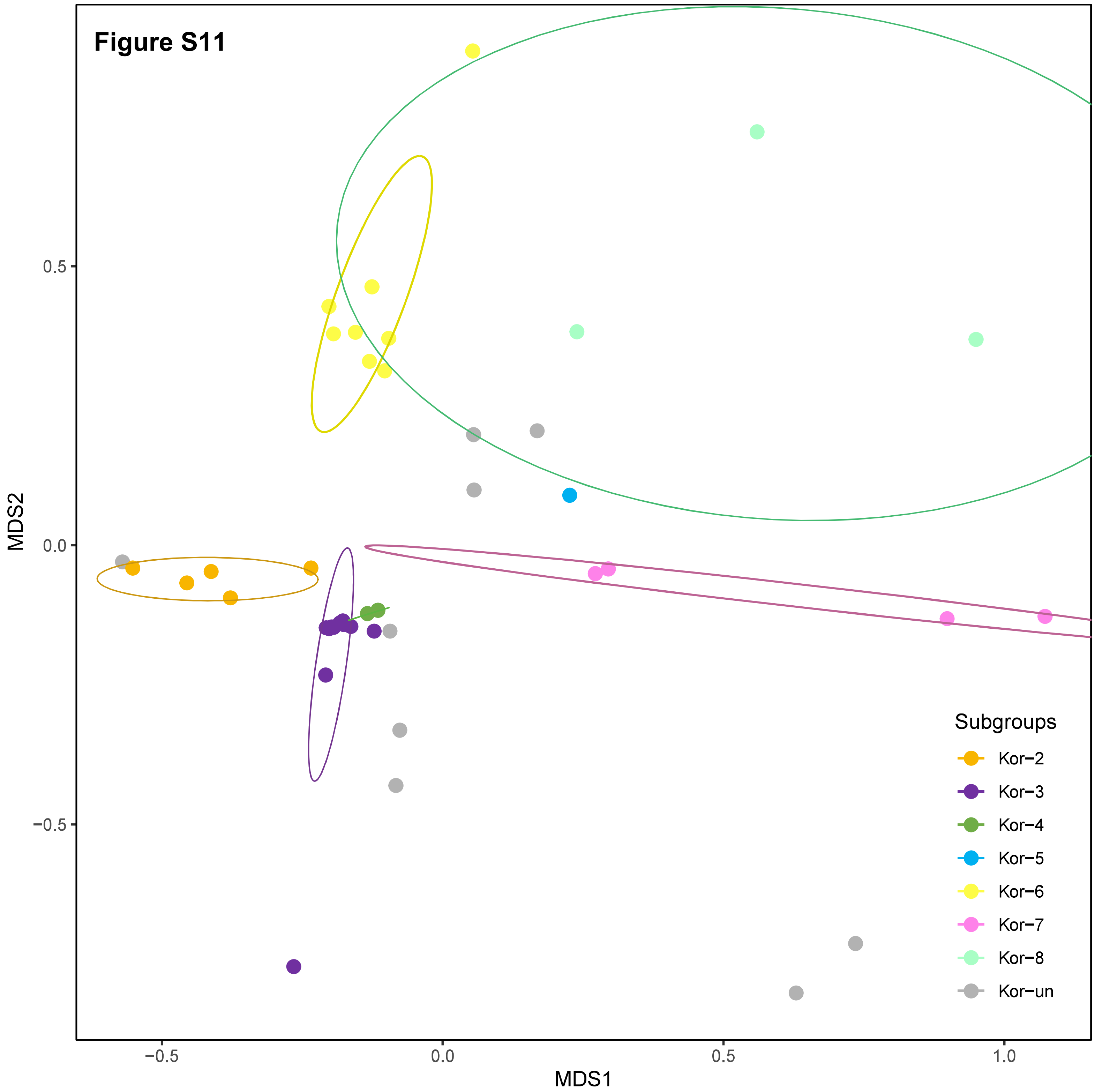
Figure S11
MDS1
MDS2
0.5
0.0
−0.5
−0.5
0.0
0.5
1.0
Subgroups
Kor−2
Kor−3
Kor−4
Kor−5
Kor−6
Kor−7
Kor−8
Kor−un

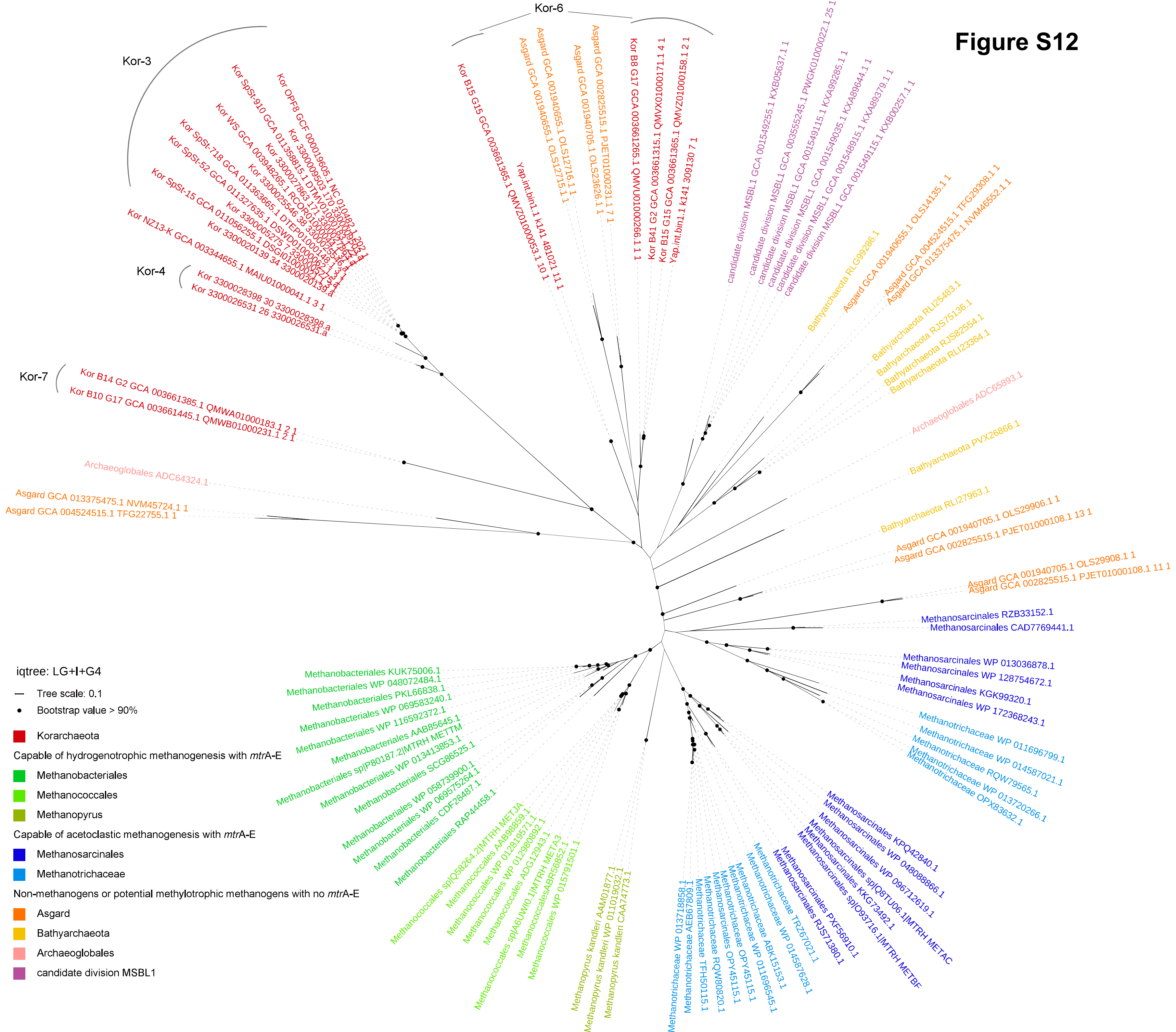
Kor-3
Kor-4
Kor-6
Kor-7
Kor OPF8 GCF 000019605.1 NC 010482.1 202
Kor SpSt-910 GCA 011358815.1 DTMV01000030.1 3
Kor WS GCA 003948265.1 RCOR01000054.1 7
Kor SpSt-718 GCA 011363665.1 DTEP01000148.1 3
Kor SpSt-52 GCA 011327635.1 DSWD01000062.1 3
Kor SpSt-15 GCA 011056255.1 DSGI01000062.1 5
Kor NZ13-K GCA 003344655.1 MAIU01000041.1 3 1
Kor 3300028398 30 3300028398.a
Kor 3300026531 26 3300026531.a
Kor B14 G2 GCA 003661385.1 QMWA01000183.1 2 1
Kor B10 G17 GCA 003661445.1 QMWB01000231.1 2 1
Archaeoglobales ADC64324.1
Asgard GCA 013375475.1 NVM45724.1 1
Asgard GCA 004524515.1 TFG22755.1 1
Kor B15 G15 GCA 003661365.1 QMVZ01000053.1 10 1
Yap.int.bin1.1 k141 481021 11 1
Asgard GCA 001940655.1 OLS12715.1 1
Asgard GCA 001940655.1 OLS12716.1 1
Asgard GCA 001940705.1 OLS23626.1 1
Asgard GCA 002825515.1 PJET01000231.1 7 1
Kor B8 G17 GCA 003661265.1 QMVU01000266.1 1
Kor B41 G2 GCA 003661315.1 QMVX01000171.1 4 1
Kor B15 G15 GCA 003661365.1 QMVZ01000158.1 2 1
Yap.int.bin1.1 k141 309130 7 1
candidate division MSBL1 GCA 001549255.1 KXB05637.1 1
candidate division MSBL1 GCA 003555245.1 PWGK01000022.1 25 1
candidate division MSBL1 GCA 001549115.1 KXA99285.1 1
candidate division MSBL1 GCA 001549035.1 KXA89644.1 1
candidate division MSBL1 GCA 001548915.1 KXA89379.1 1
candidate division MSBL1 GCA 001549115.1 KXB00257.1 1
Bathyarchaeota RLG99286.1
Asgard GCA 001940655.1 OLS14135.1 1
Asgard GCA 004524515.1 TFG29308.1 1
Asgard GCA 013375475.1 NVM46552.1 1
Bathyarchaeota RLI25483.1
Bathyarchaeota RJS75136.1
Bathyarchaeota RJS82554.1
Bathyarchaeota RLI23364.1
Archaeoglobales ADC65893.1
Bathyarchaeota PVX26866.1
Bathyarchaeota RLI27963.1
Asgard GCA 001940705.1 OLS29906.1 1
Asgard GCA 002825515.1 PJET01000108.1 13 1
Asgard GCA 001940705.1 OLS29908.1 1
Asgard GCA 002825515.1 PJET01000108.1 11 1
Methanosarcinales RZB33152.1
Methanosarcinales CAD7769441.1
Methanosarcinales WP 013036878.1
Methanosarcinales WP 128754672.1
Methanosarcinales KGK99320.1
Methanosarcinales WP 172368243.1
Methanotrichaceae WP 011696799.1
Methanotrichaceae WP 014587021.1
Methanotrichaceae RQW79565.1
Methanotrichaceae WP 013720266.1
Methanotrichaceae OPX83632.1
Methanosarcinales KPQ42840.1
Methanosarcinales WP 048088866.1
Methanosarcinales WP 096712619.1
Methanosarcinales sp|Q8TU06.1|MTRH METAC
Methanosarcinales KKG73492.1
Methanosarcinales sp|O93716.1|MTRH METBF
Methanosarcinales PXF56910.1
Methanosarcinales RJS71380.1
Methanotrichaceae TRZ67021.1
Methanotrichaceae WP 014587628.1
Methanotrichaceae ABK15153.1
Methanotrichaceae WP 011696545.1
Methanotrichaceae OPY45115.1
Methanosarcinales OPY45115.1
Methanotrichaceae RQW80820.1
Methanotrichaceae TFH50115.1
Methanotrichaceae AEB67809.1
Methanotrichaceae WP 013718858.1
Methanopyrus kandleri CAA74773.1
Methanopyrus kandleri WP 011019032.1
Methanopyrus kandleri AAM01877.1
Methanococcales WP 015791501.1
Methanococcales ABR56852.1
Methanococcales sp|A6UWI0.1|MTRH METAE
Methanococcales ADG12943.1
Methanococcales WP 012980892.1
Methanococcales WP 012819571.1
Methanococcales AAB98859.1
Methanococcales sp|Q58264.2|MTRH METJA
Methanobacteriales RAP44458.1
Methanobacteriales CDF28487.1
Methanobacteriales WP 069575264.1
Methanobacteriales WP 058739900.1
Methanobacteriales SCG86525.1
Methanobacteriales WP 013413853.1
Methanobacteriales sp|P80187.2|MTRH METTM
Methanobacteriales AAB85645.1
Methanobacteriales WP 116592372.1
Methanobacteriales WP 069583240.1
Methanobacteriales PKL66838.1
Methanobacteriales WP 048072484.1
Methanobacteriales KUK75006.1
iqtree: LG+I+G4
Tree scale: 0.1
Bootstrap value > 90%
Korarchaeota
Capable of hydrogenotrophic methanogenesis with mtrA-E
Methanobacteriales
Methanococcales
Methanopyrus
Capable of acetoclastic methanogenesis with mtrA-E
Methanosarcinales
Methanotrichaceae
Non-methanogens or potential methylotrophic methanogens with no mtrA-E
Asgard
Bathyarchaeota
Archaeoglobales
candidate division MSBL1

Figure S12

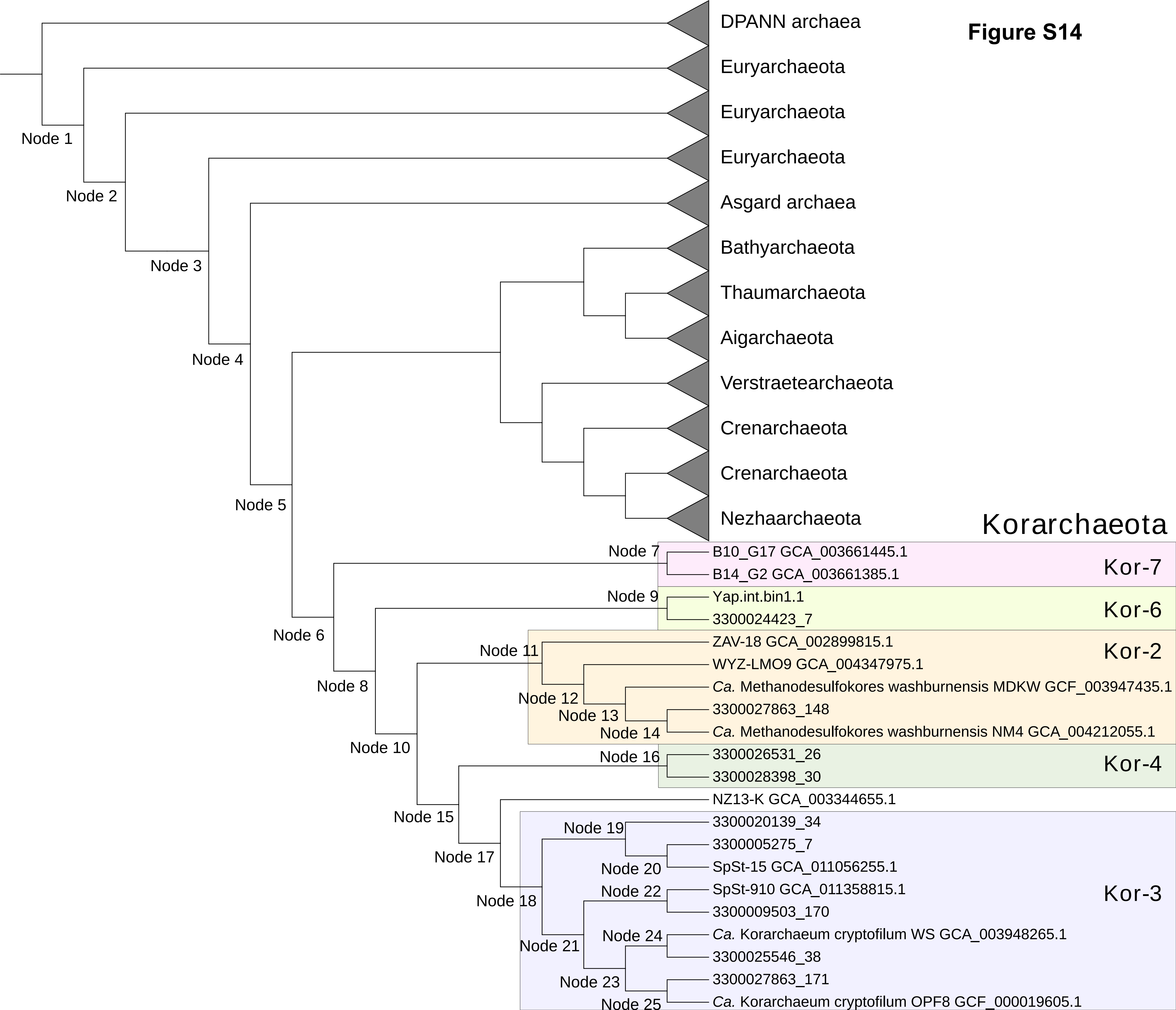

Figure S14
DPANN archaea
Euryarchaeota
Euryarchaeota
Euryarchaeota
Asgard archaea
Bathyarchaeota
Thaumarchaeota
Aigarchaeota
Verstraetearchaeota
Crenarchaeota
Crenarchaeota
Nezhaarchaeota
Korarchaeota
Node 1
Node 2
Node 3
Node 4
Node 5
Node 6
Node 7
B10_G17 GCA_003661445.1
B14_G2 GCA_003661385.1
Kor-7
Node 8
Node 9
Yap.int.bin1.1
3300024423_7
Kor-6
Node 10
Node 11
ZAV-18 GCA_002899815.1
WYZ-LMO9 GCA_004347975.1
Node 12
Ca. Methanodesulfokores washburnensis MDKW GCF_003947435.1
Node 13
3300027863_148
Node 14
Ca. Methanodesulfokores washburnensis NM4 GCA_004212055.1
Kor-2
Node 15
Node 16
3300026531_26
3300028398_30
Kor-4
Node 17
NZ13-K GCA_003344655.1
Node 18
Node 19
3300020139_34
3300005275_7
Node 20
SpSt-15 GCA_011056255.1
Node 21
Node 22
SpSt-910 GCA_011358815.1
3300009503_170
Node 23
Node 24
Ca. Korarchaeum cryptofilum WS GCA_003948265.1
3300025546_38
Node 25
3300027863_171
Ca. Korarchaeum cryptofilum OPF8 GCF_000019605.1
Kor-3

Figure S16

a. concatenated sequences of mcrABCDG

iqtree: LG+C60+F+G

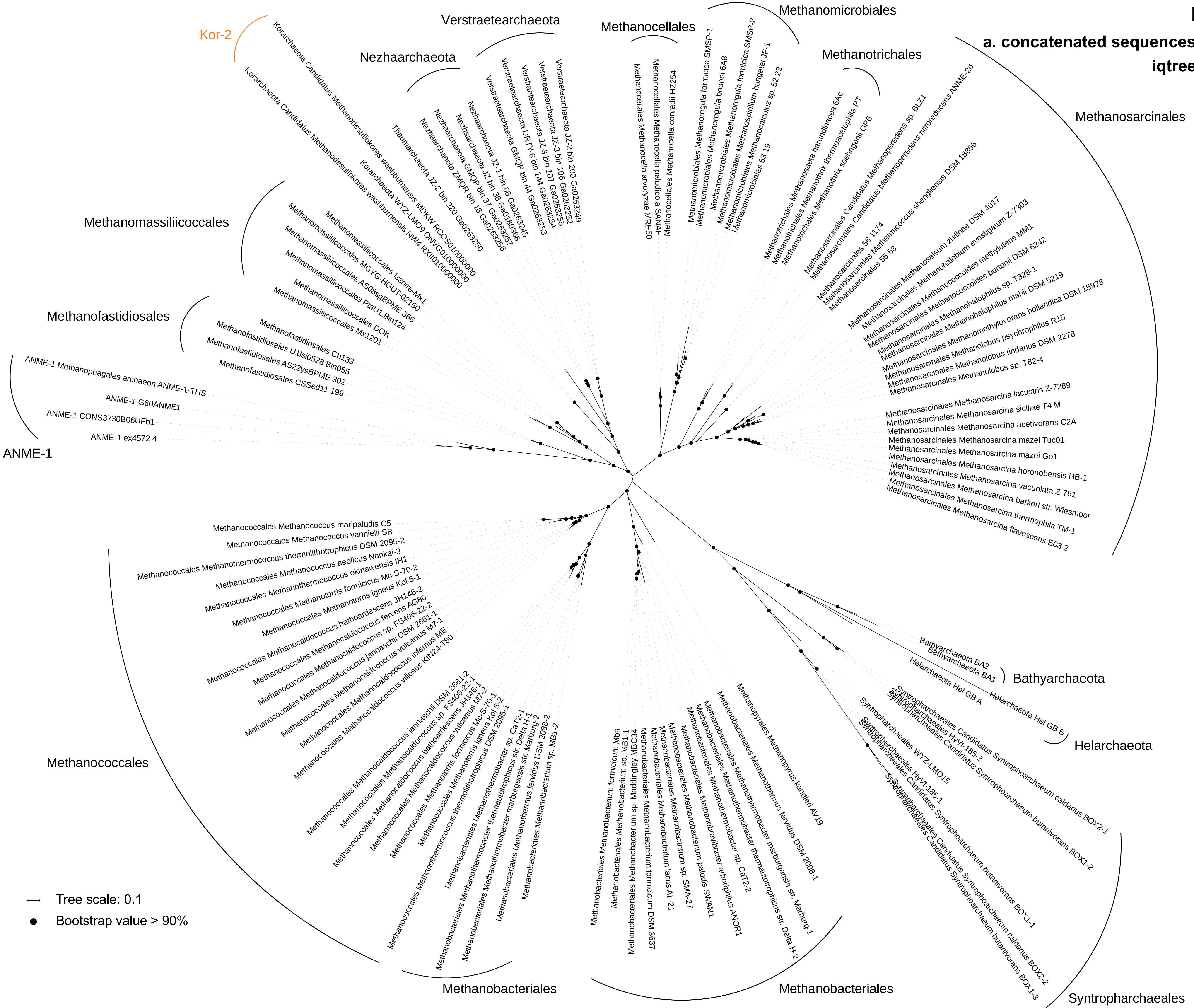

Figure S16

b. *mcrA*

iqtree: LG+C50+F+G

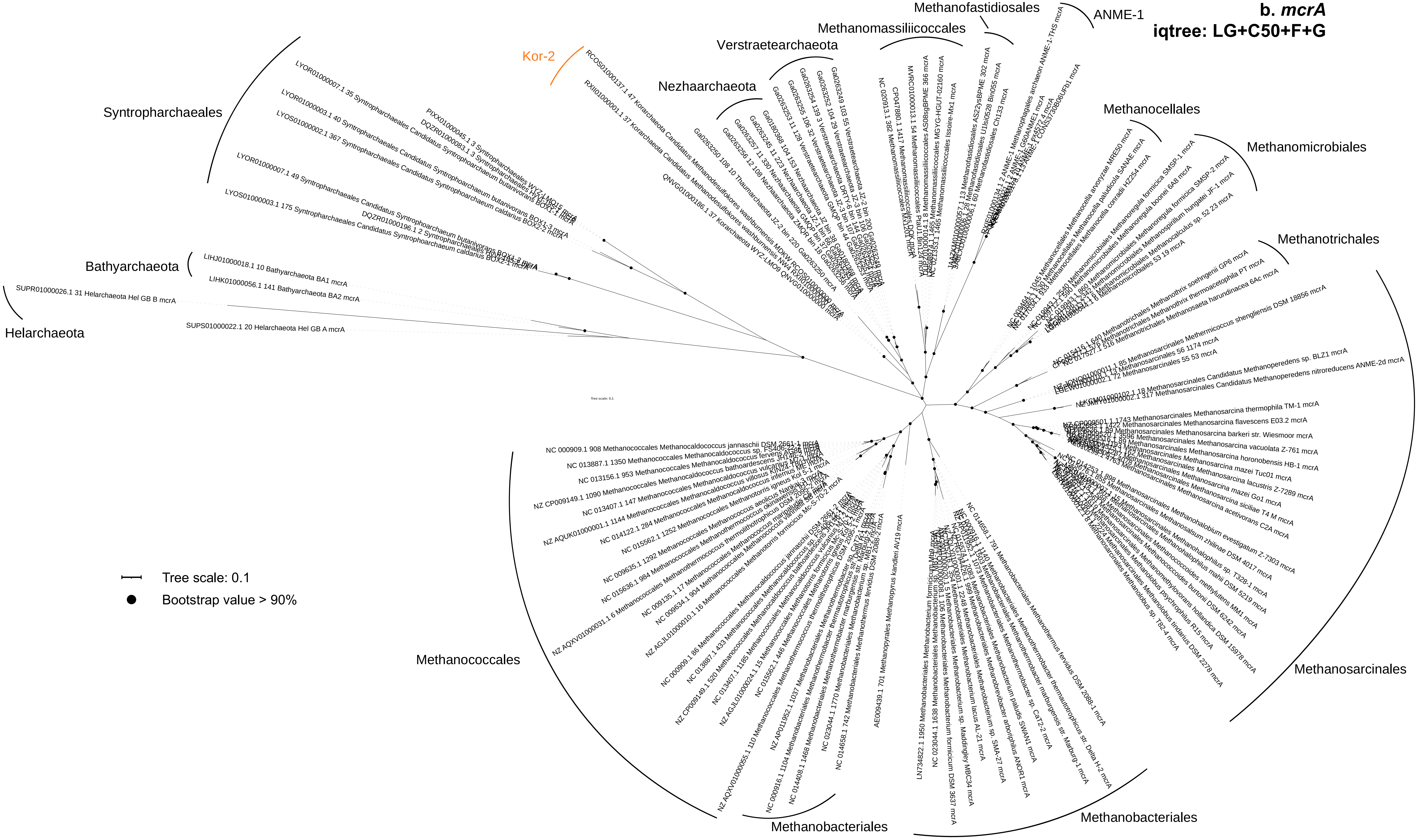

Figure S17

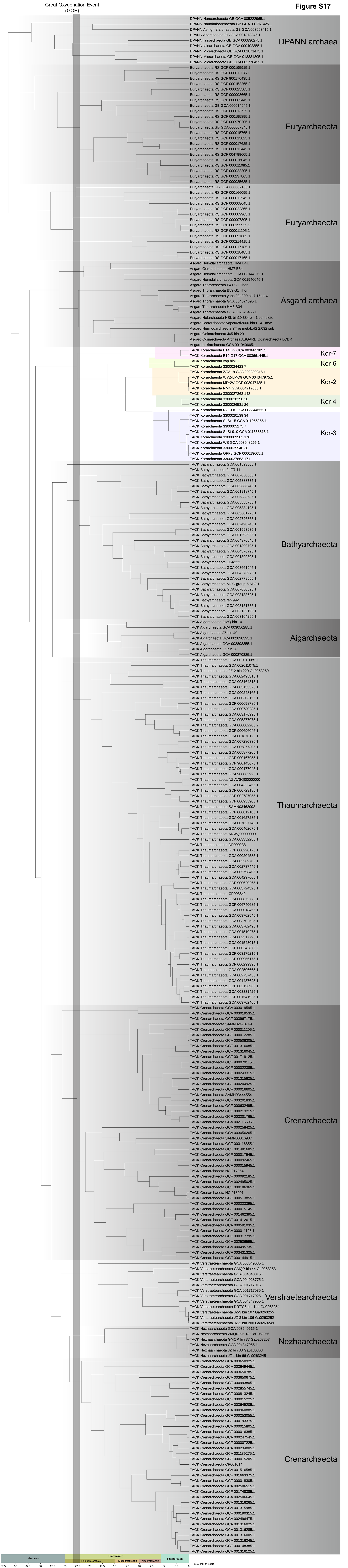

Great Oxygenation Event
(GOE)
DPANN archaea
Euryarchaeota
Euryarchaeota
Asgard archaea
Kor-7
Kor-6
Kor-2
Kor-4
Kor-3
Bathyarchaeota
Aigarchaeota
Thaumarchaeota
Crenarchaeota
Verstraetearchaeota
Nezhaarchaeota
Crenarchaeota
Archean
Proterozoic
Paleoproterozoic
Mesoproterozoic
Neoproterozoic
Phanerozoic
(100 million years)

Figure S18

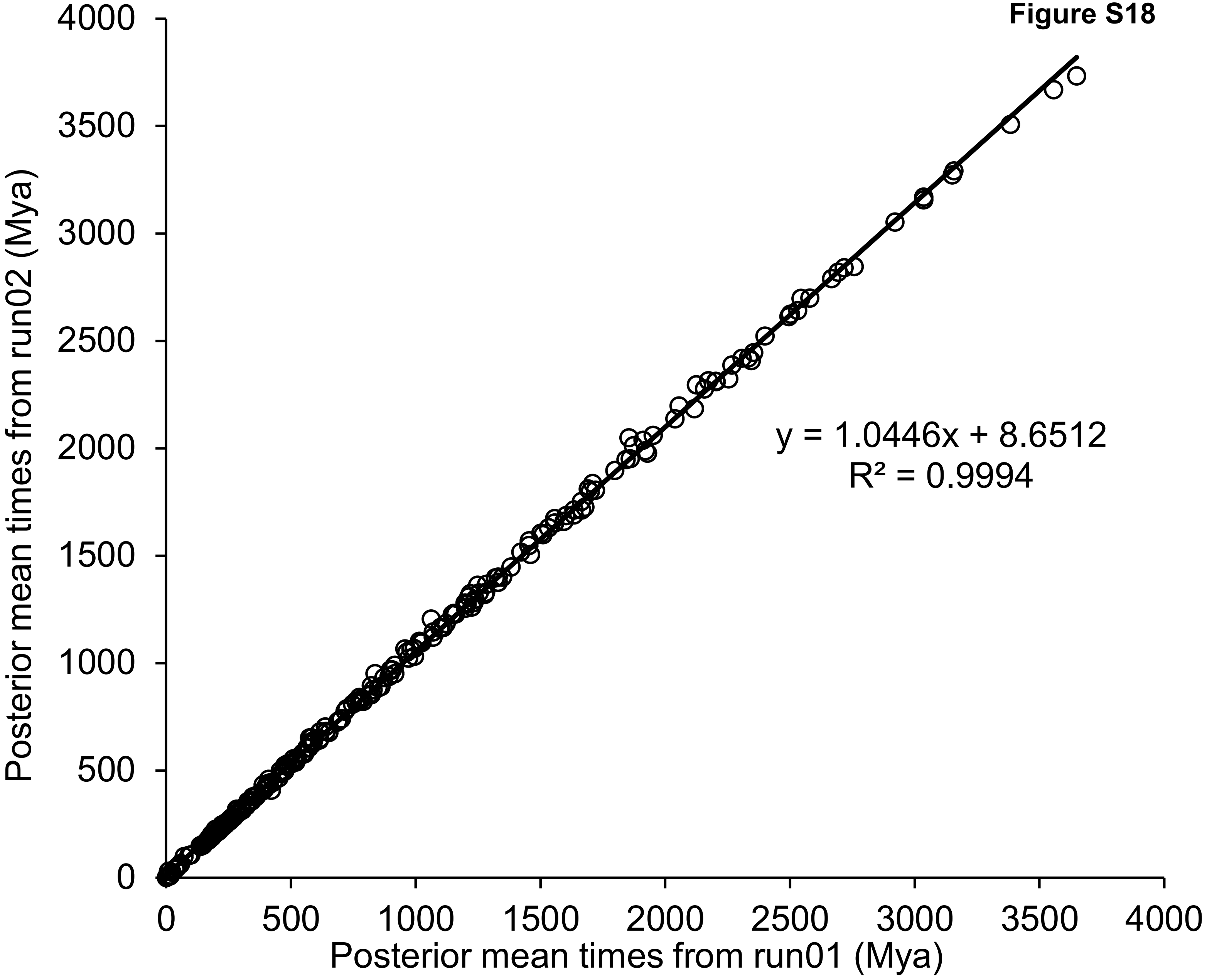

y = 1.0446x + 8.6512
R² = 0.9994
Posterior mean times from run02 (Mya)
Posterior mean times from run01 (Mya)
0
500
1000
1500
2000
2500
3000
3500
4000
