# Supplementary material for: Wood–Ljungdahl pathway found in novel marine Korarchaeota groups illuminates their evolutionary history
Source: mSystems. 2023 Jul 17;8(4):e00305-23. doi: 10.1128/msystems.00305-23 (PMC10469681; doi:10.1128/msystems.00305-23)

Kor−HOG0000025

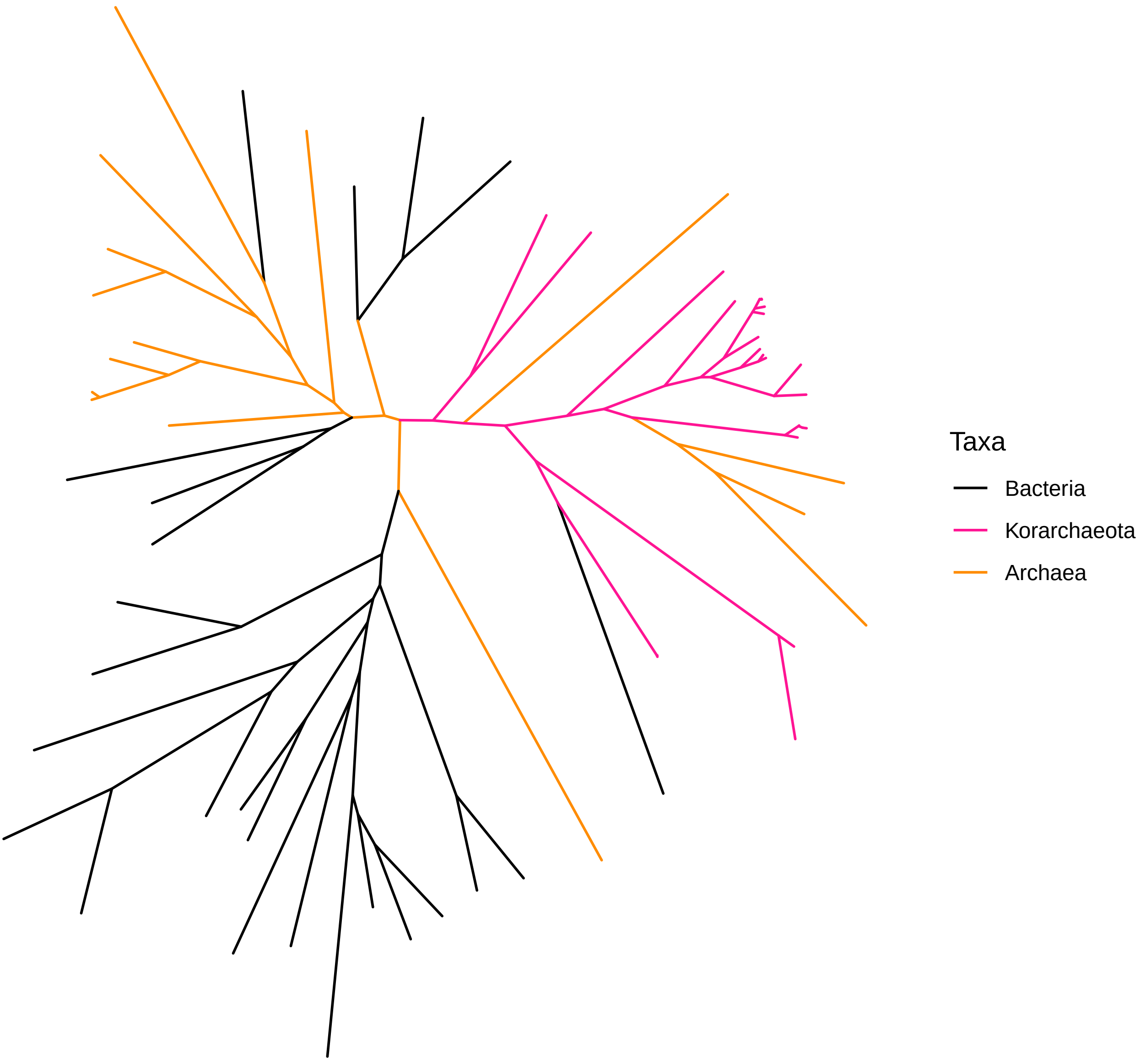

Kor−HOG0000102

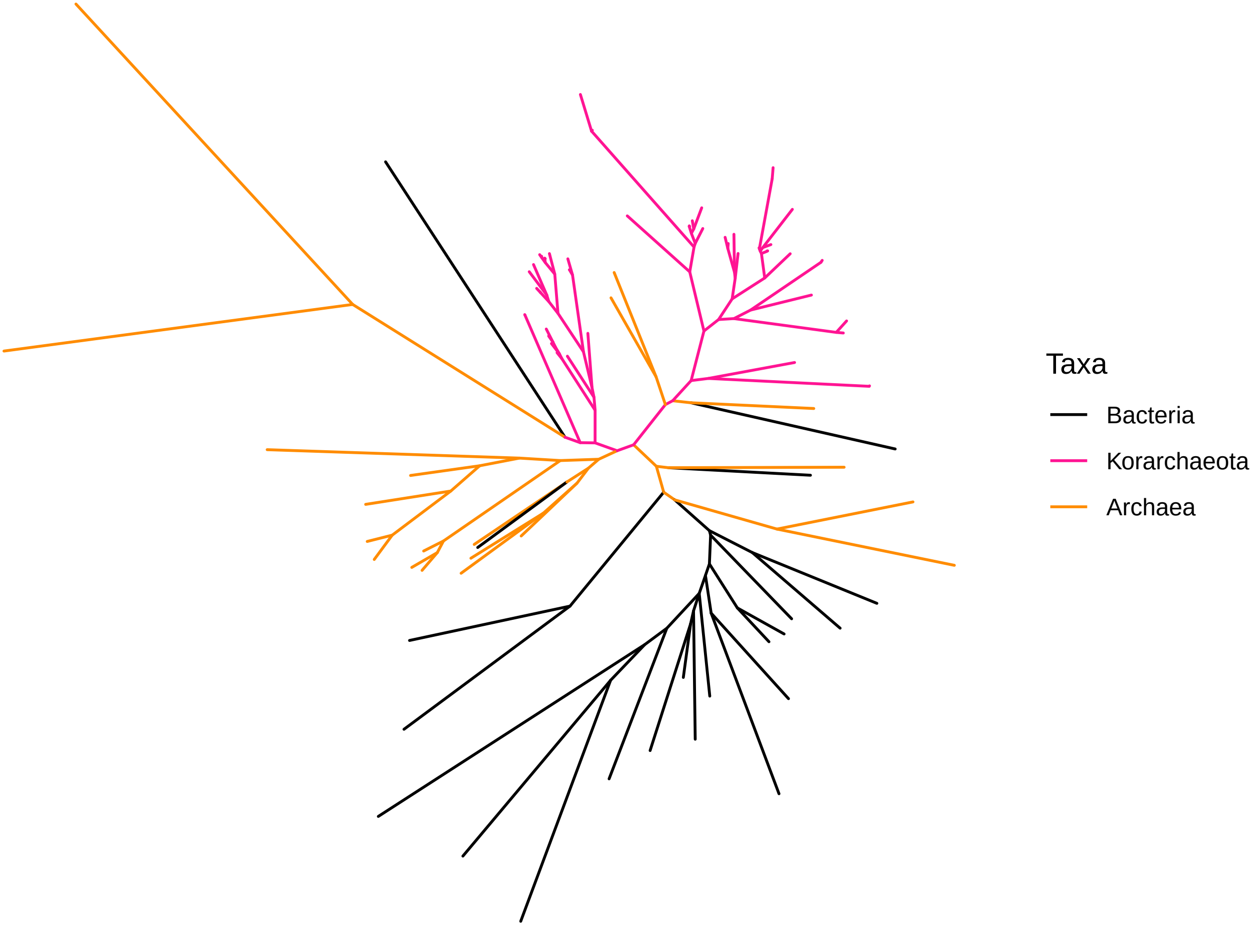

Kor−HOG0000208

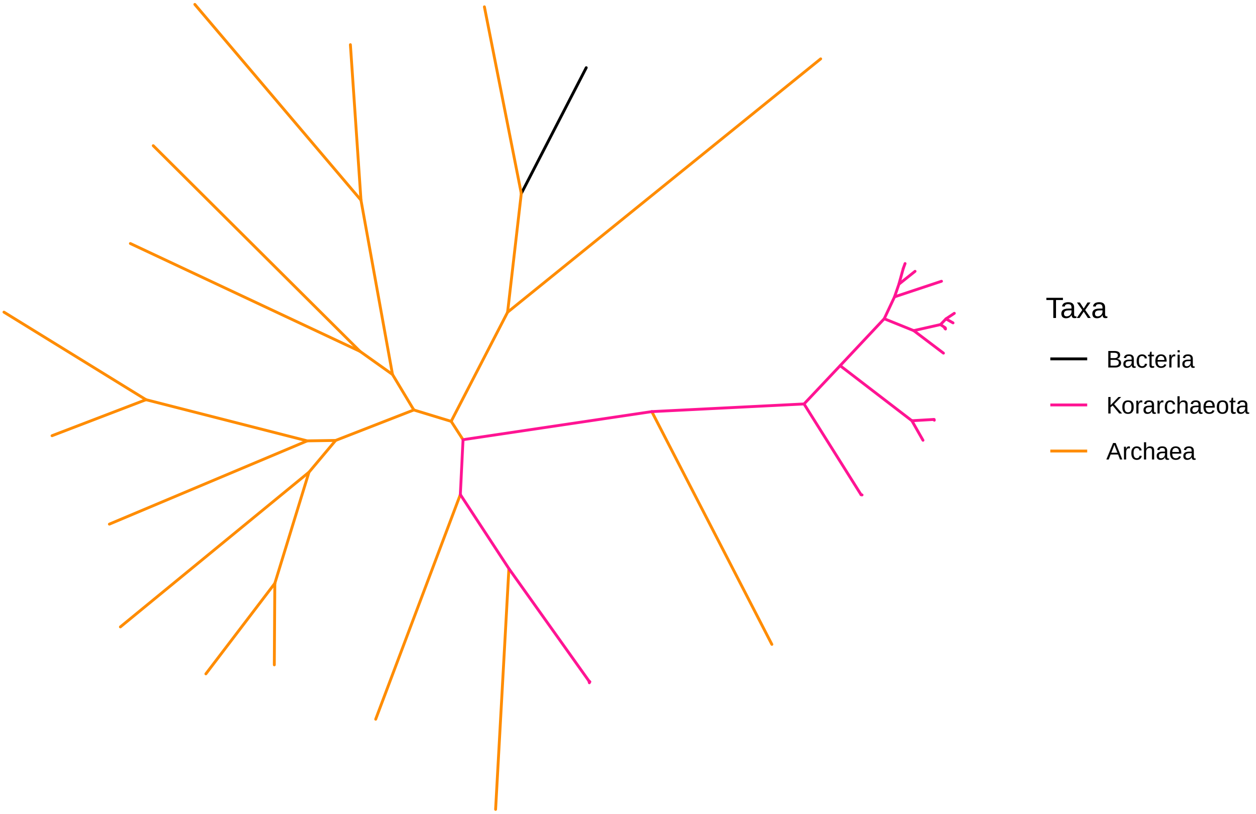

Kor−HOG0000209

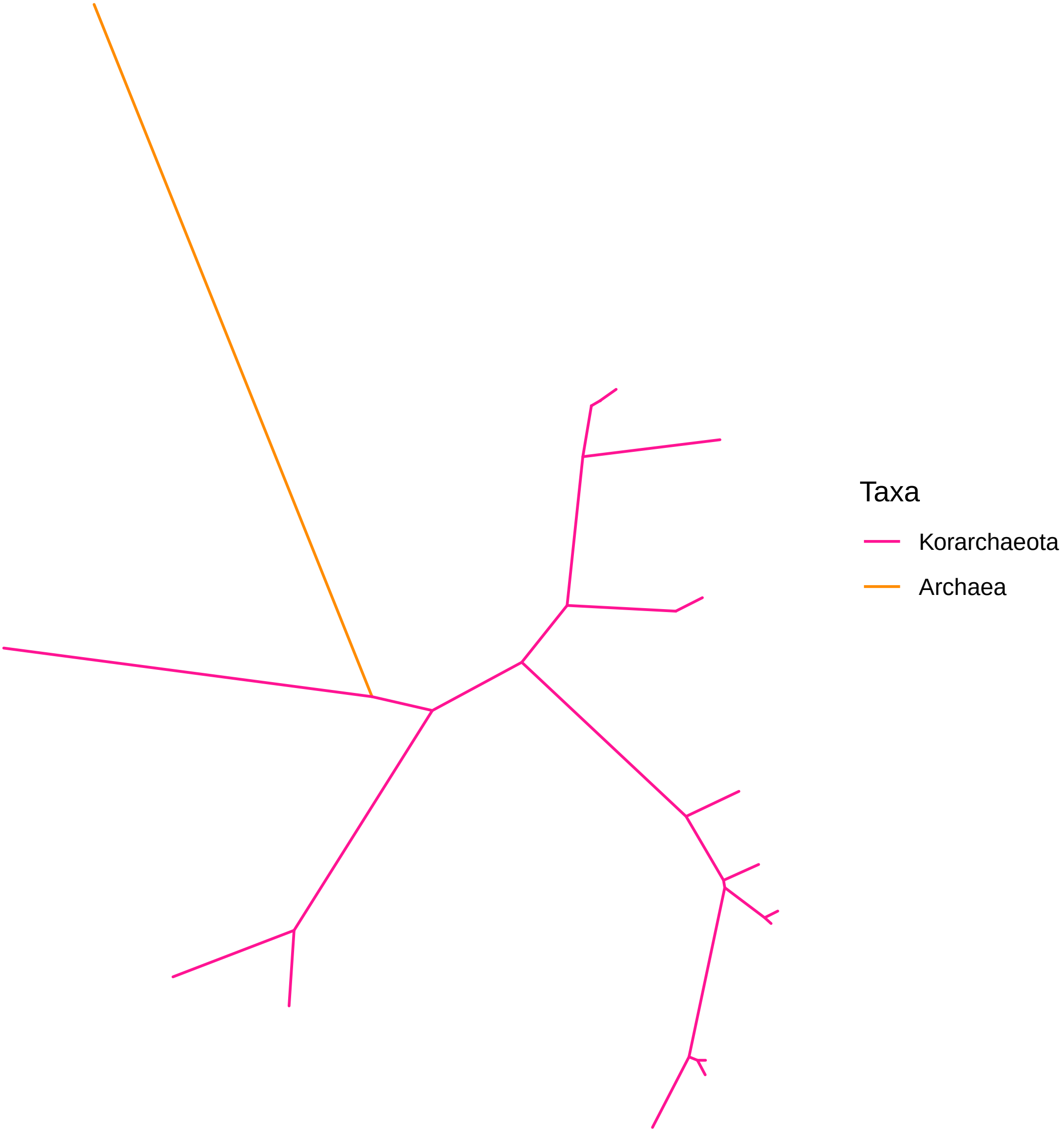

Kor−HOG0000304

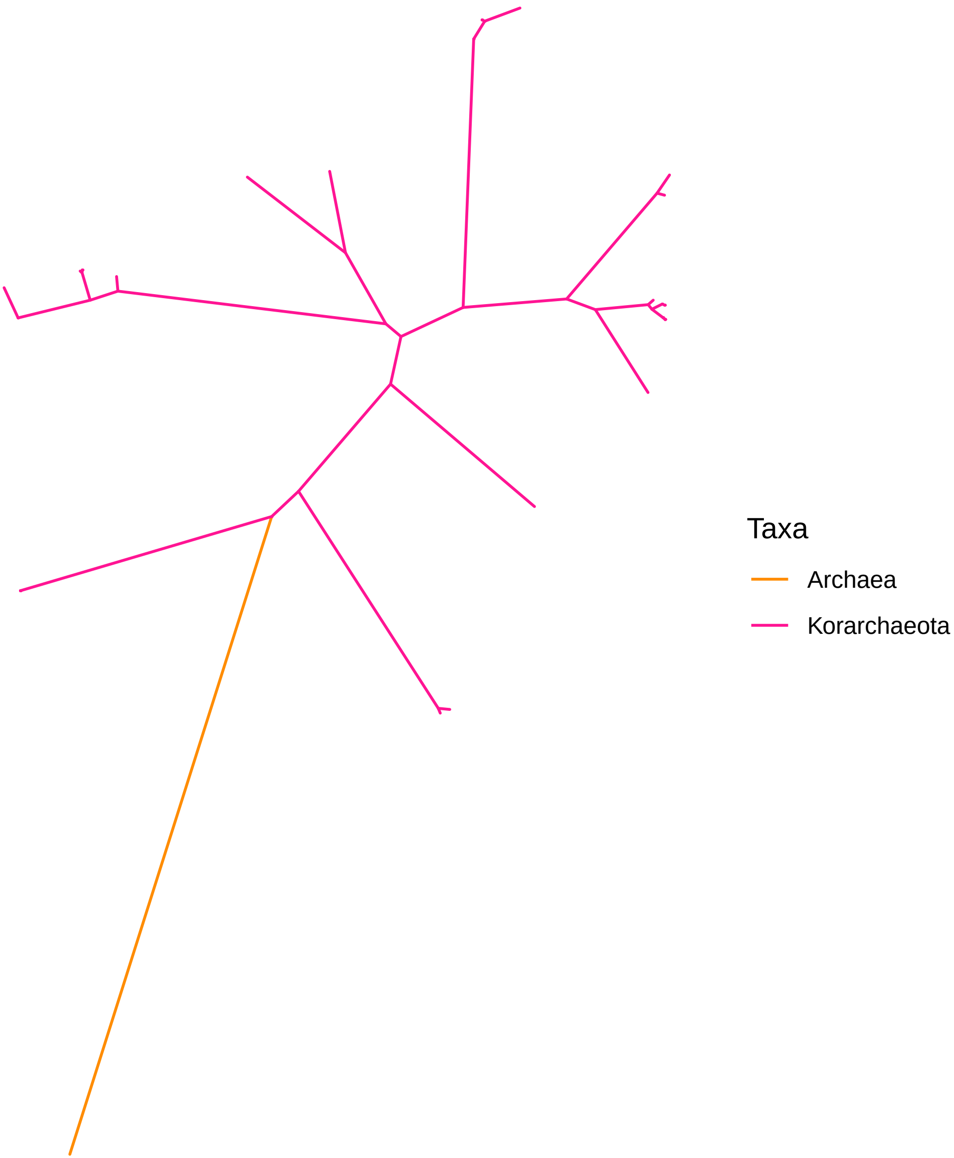

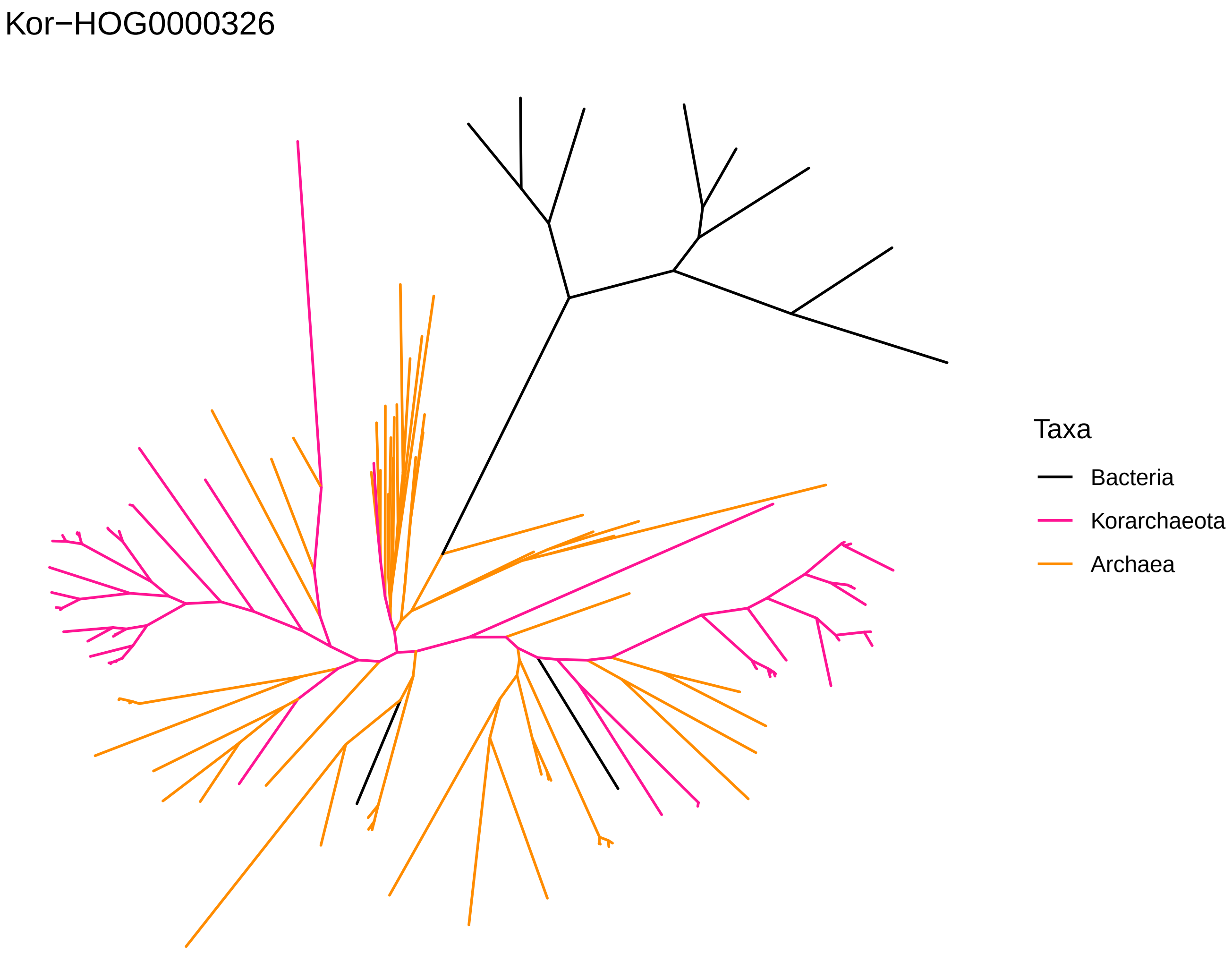

Kor−HOG0000326
Taxa
Bacteria
Korarchaeota
Archaea

Kor−HOG0000344

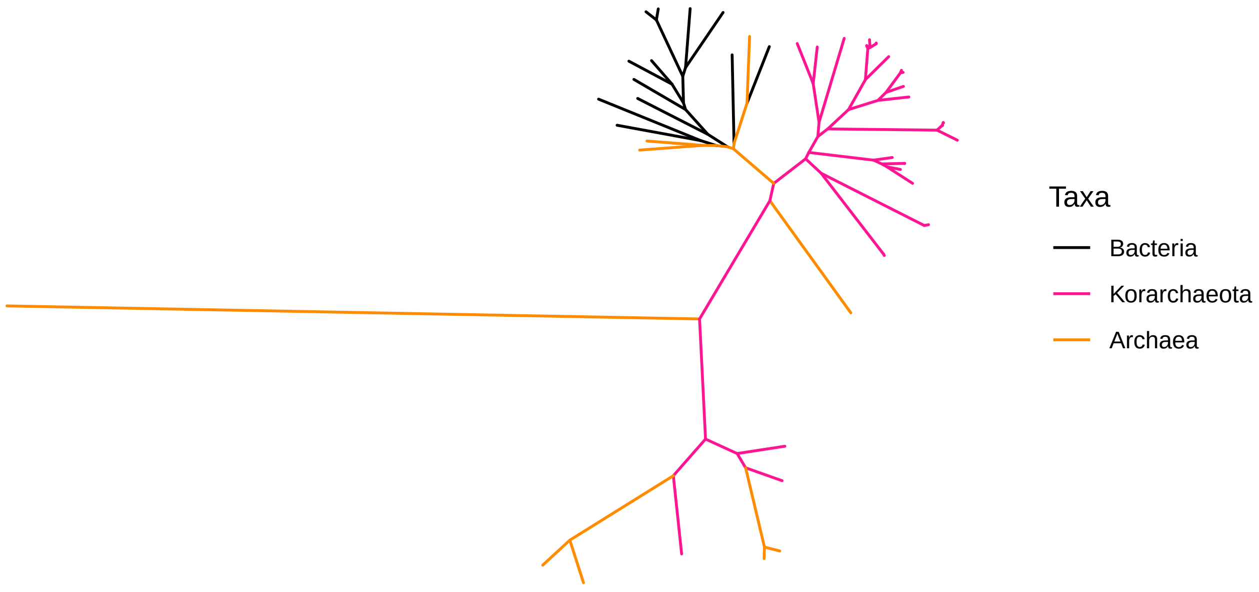

Kor−HOG0000347

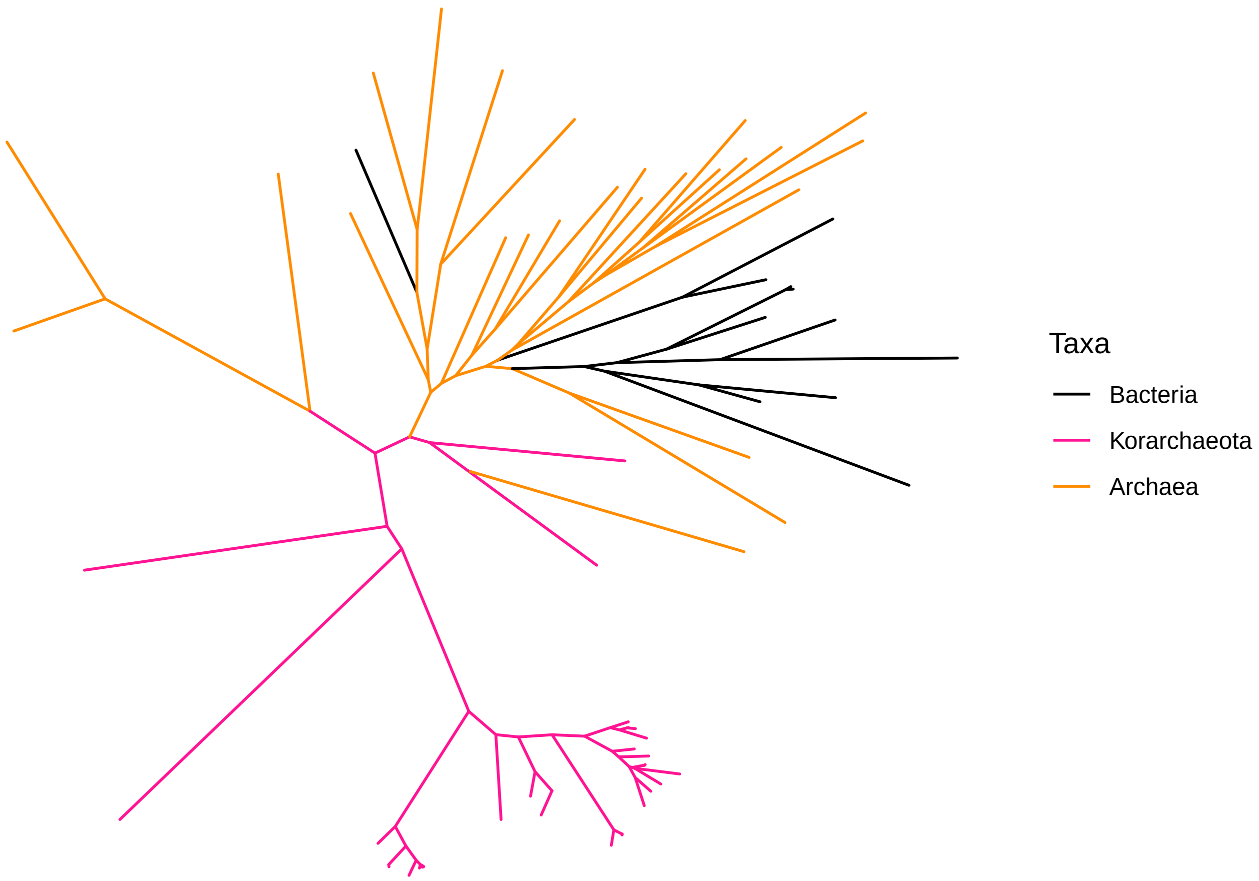

Kor−HOG0000364

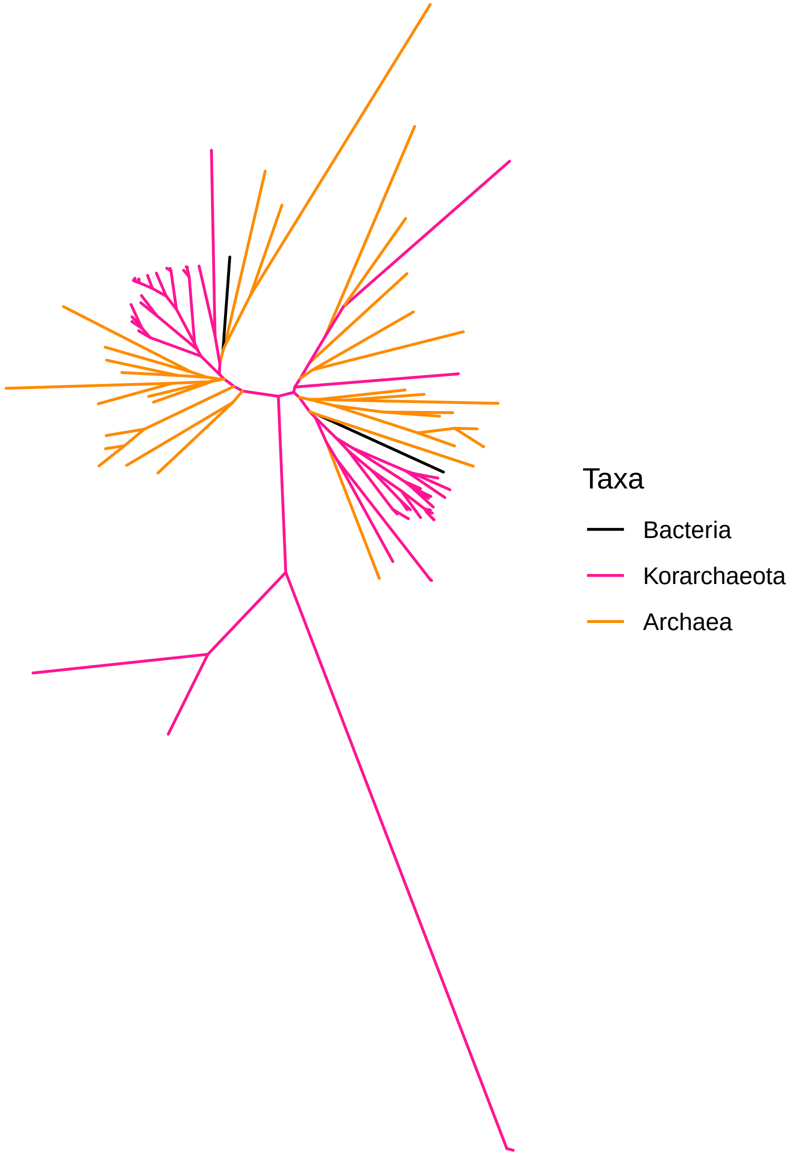

Kor−HOG0000402

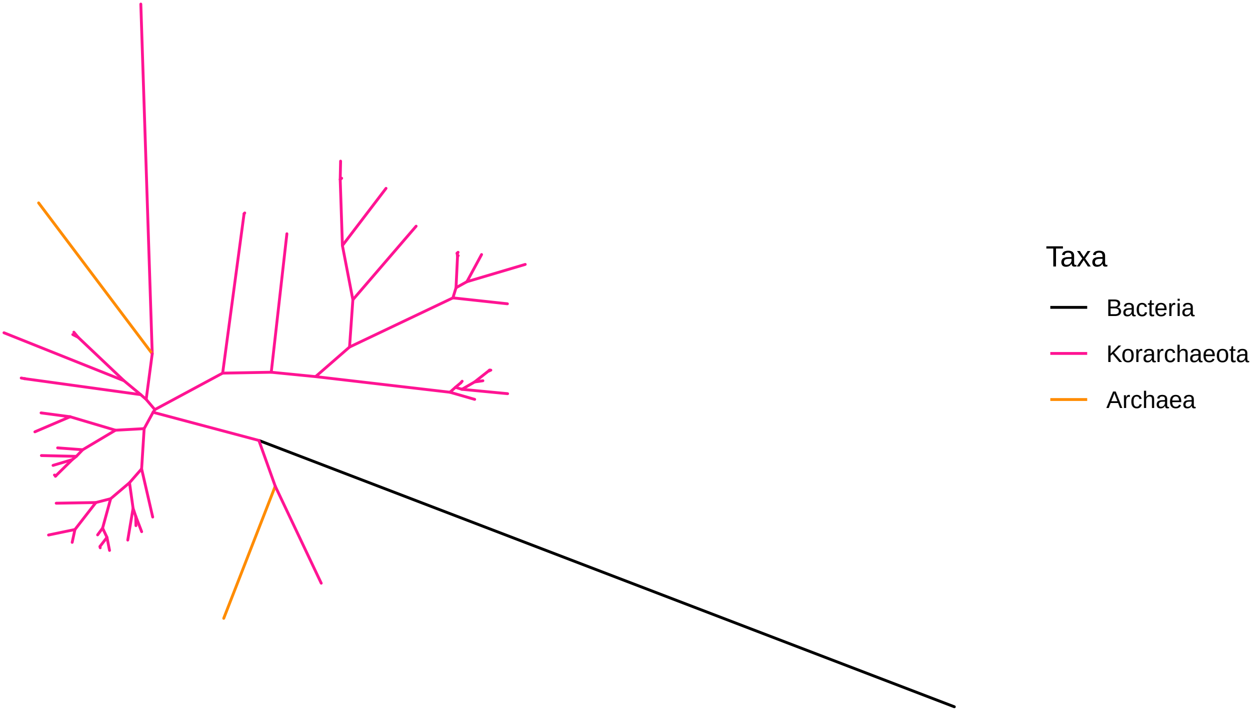

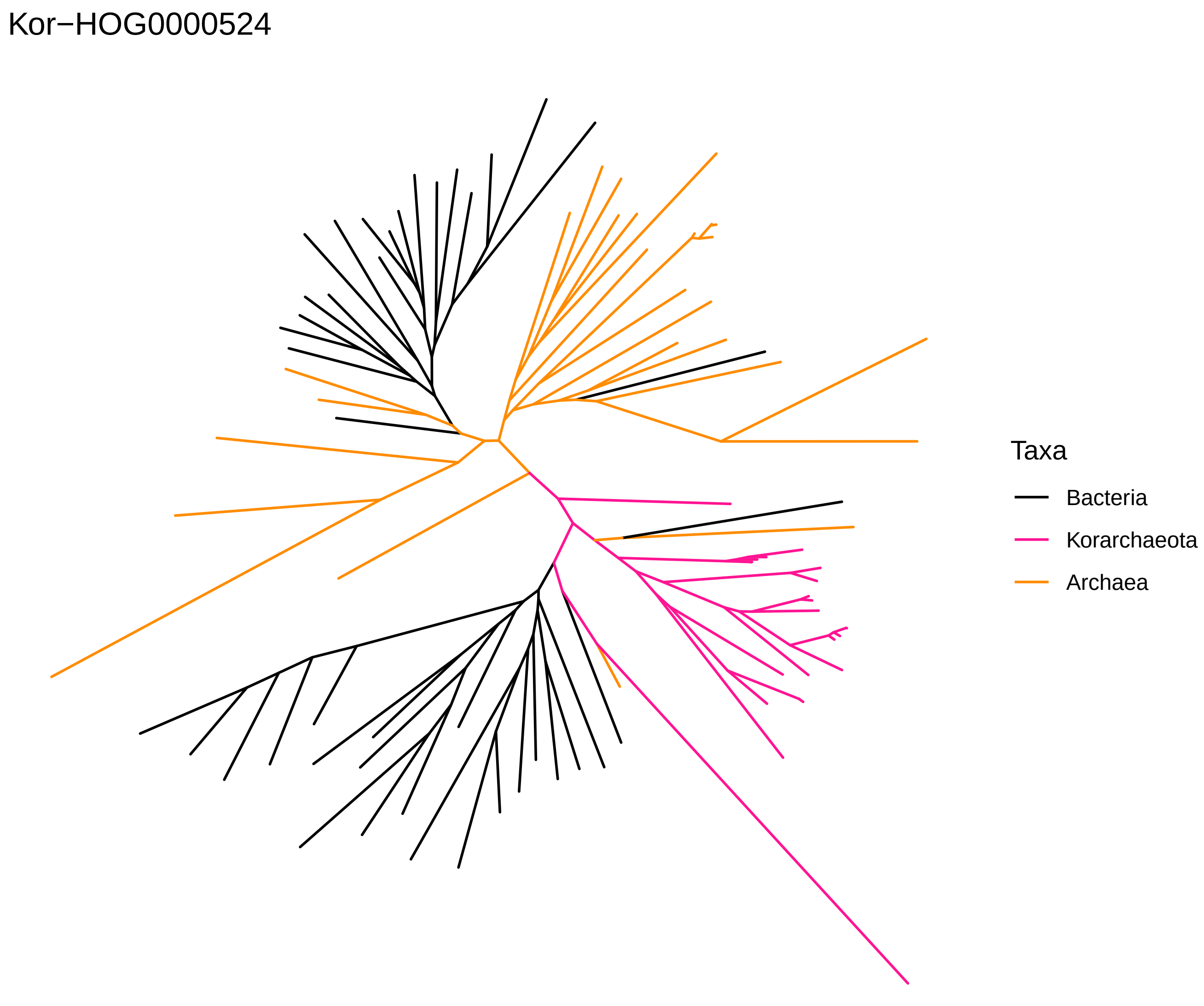

Kor−HOG0000524
Taxa
Bacteria
Korarchaeota
Archaea

Kor−HOG0000658

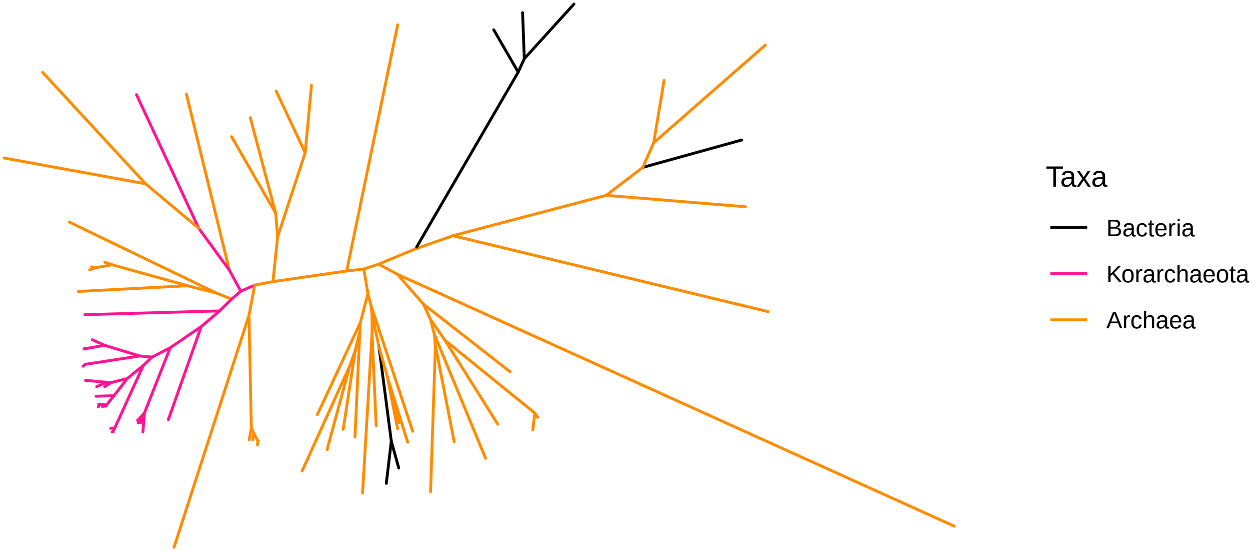

Kor−HOG0000660

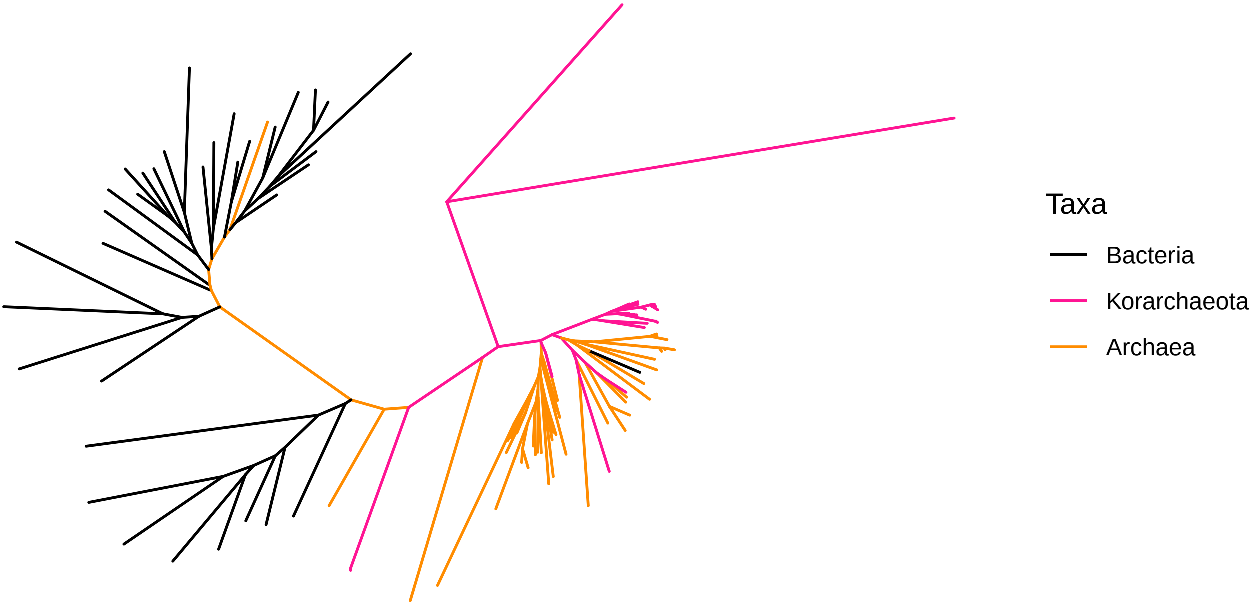

Kor−HOG0000683

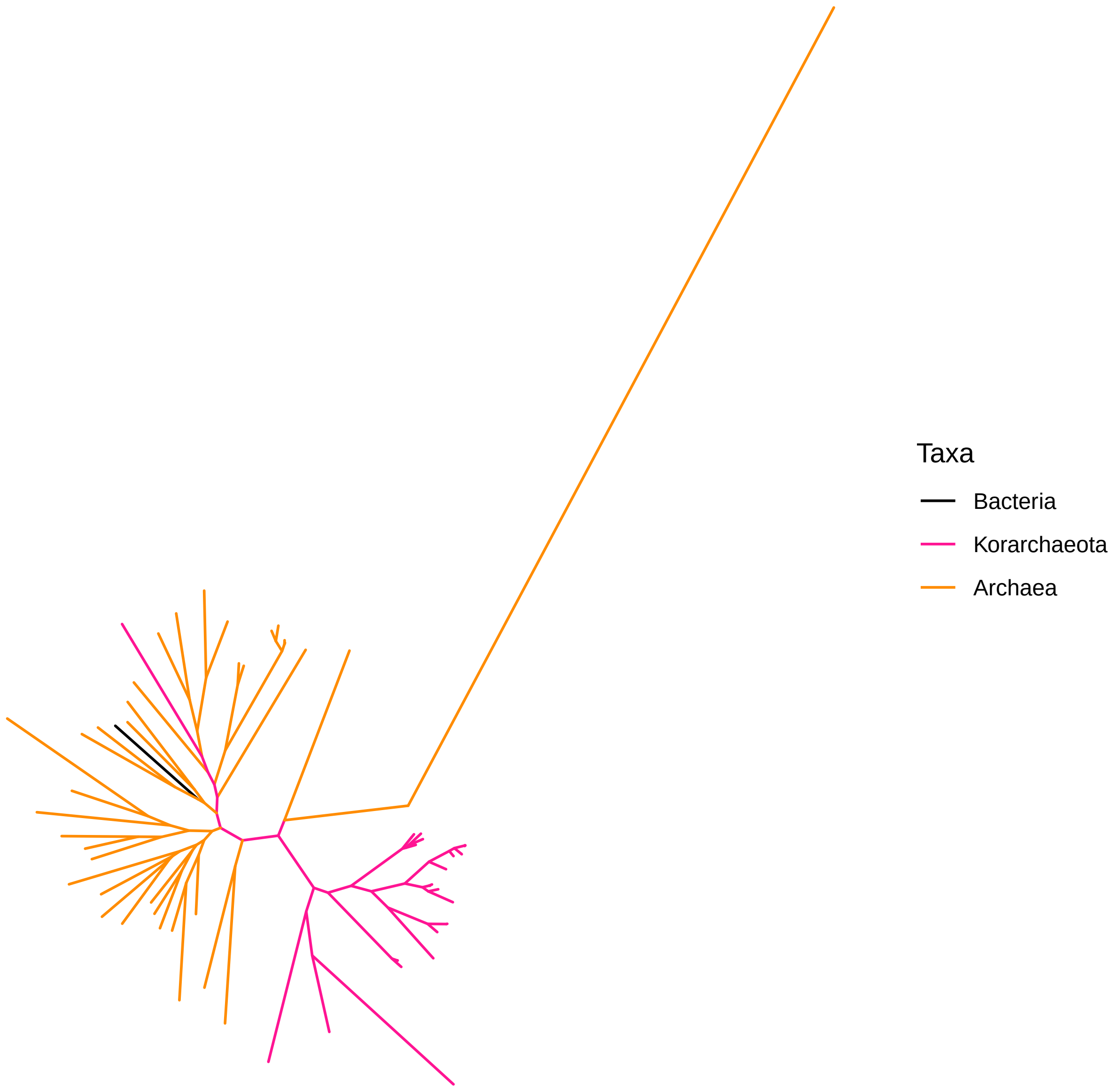

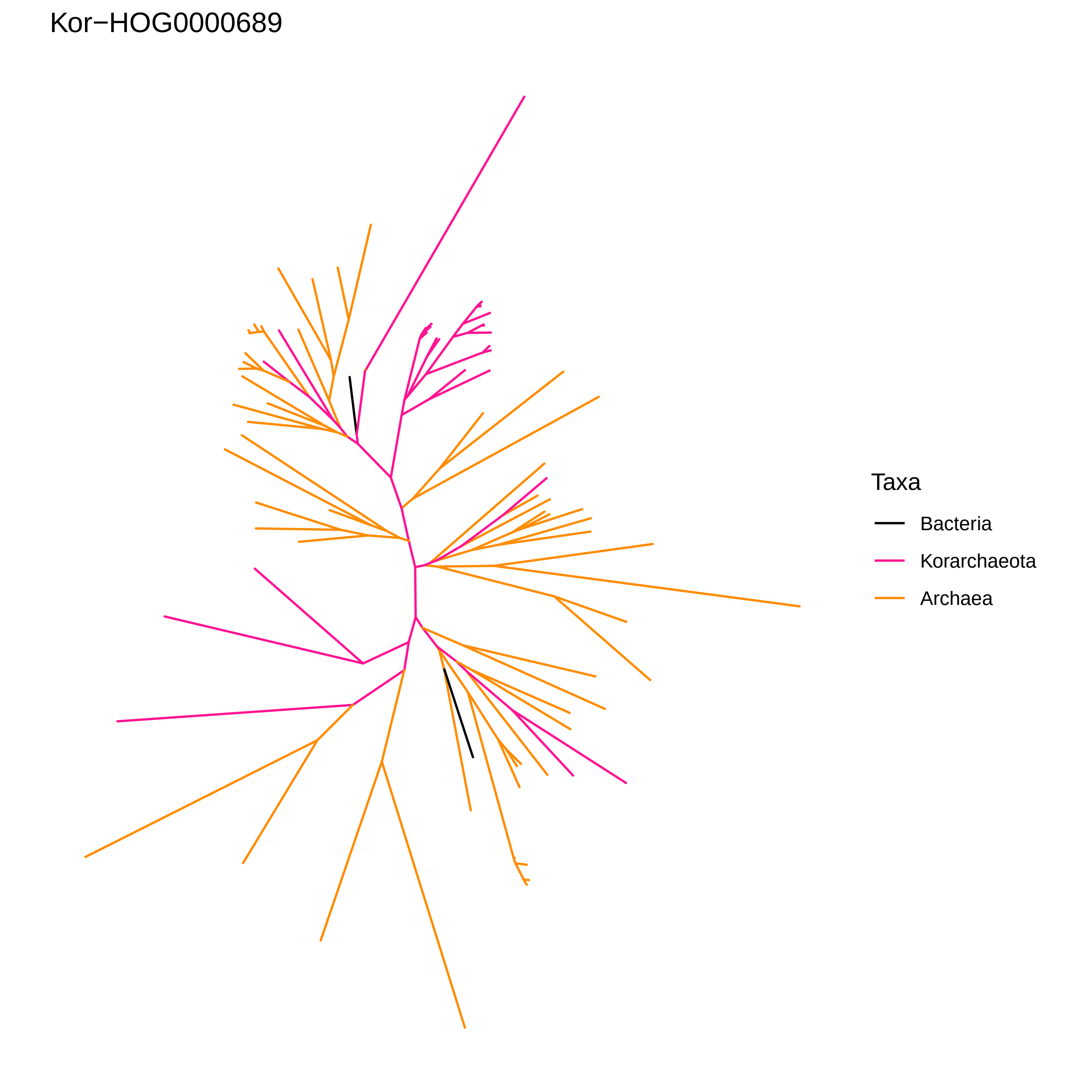

Kor−HOG0000689
Taxa
Bacteria
Korarchaeota
Archaea

Kor−HOG0000704

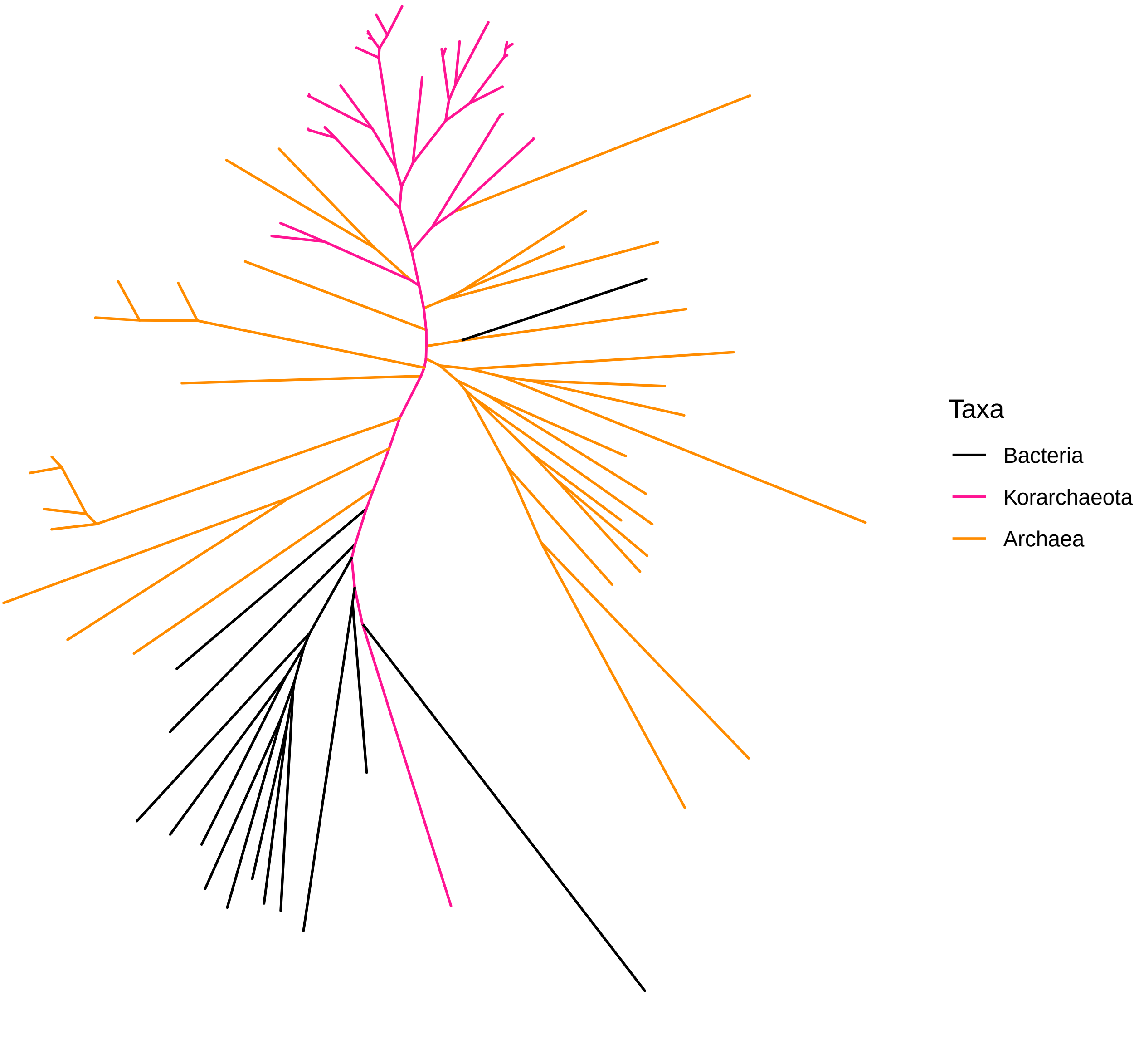

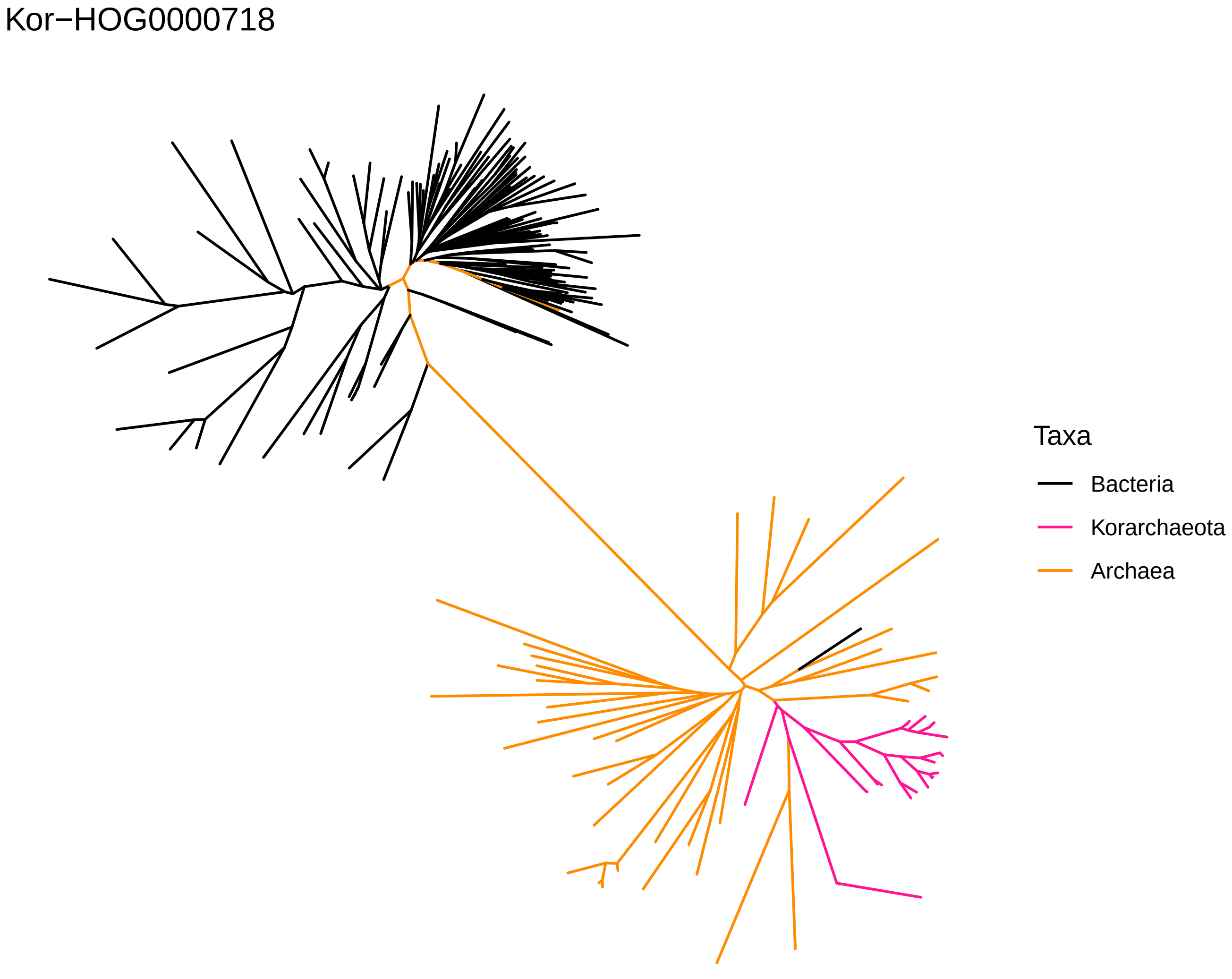
Kor−HOG0000718
Taxa
Bacteria
Korarchaeota
Archaea

Kor−HOG0000729

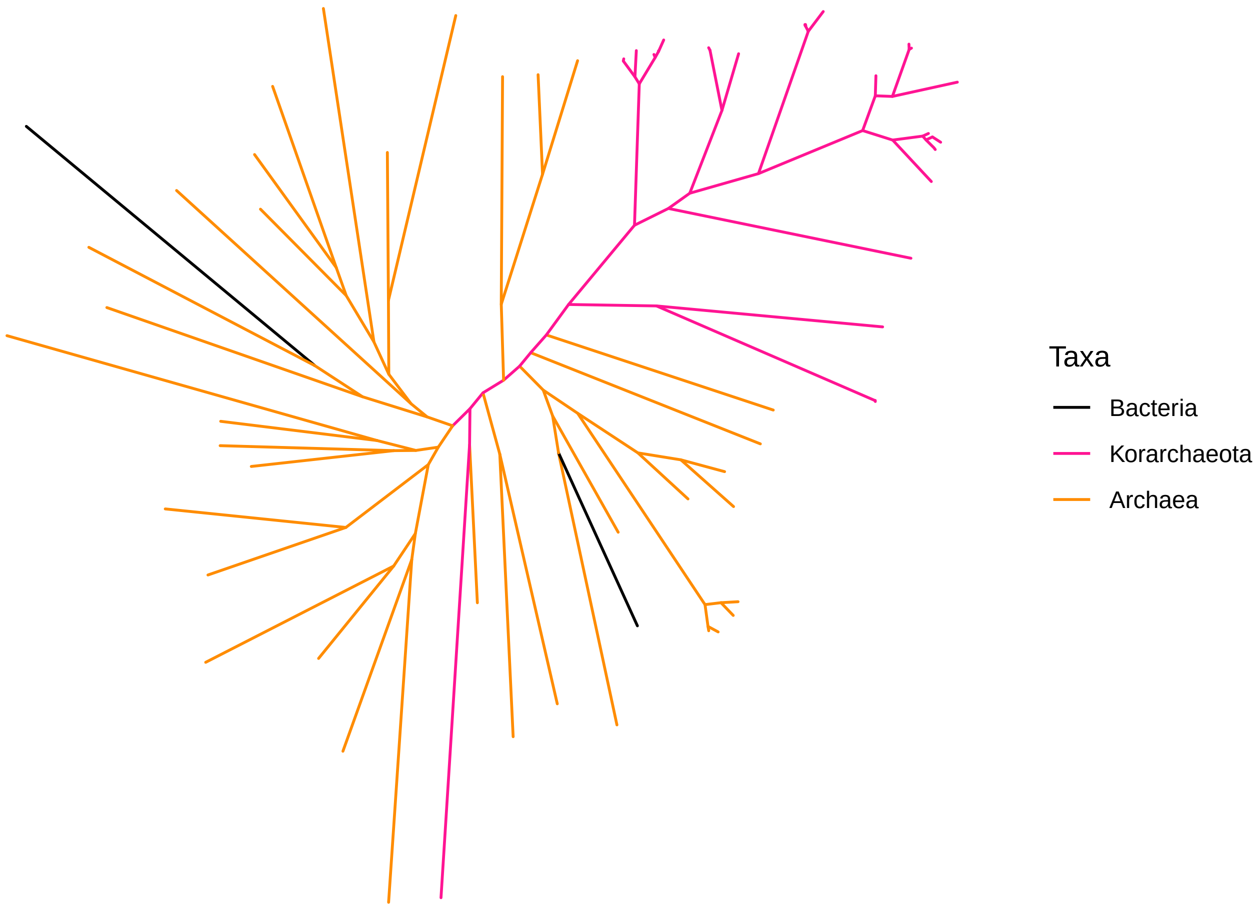

Kor−HOG0000731

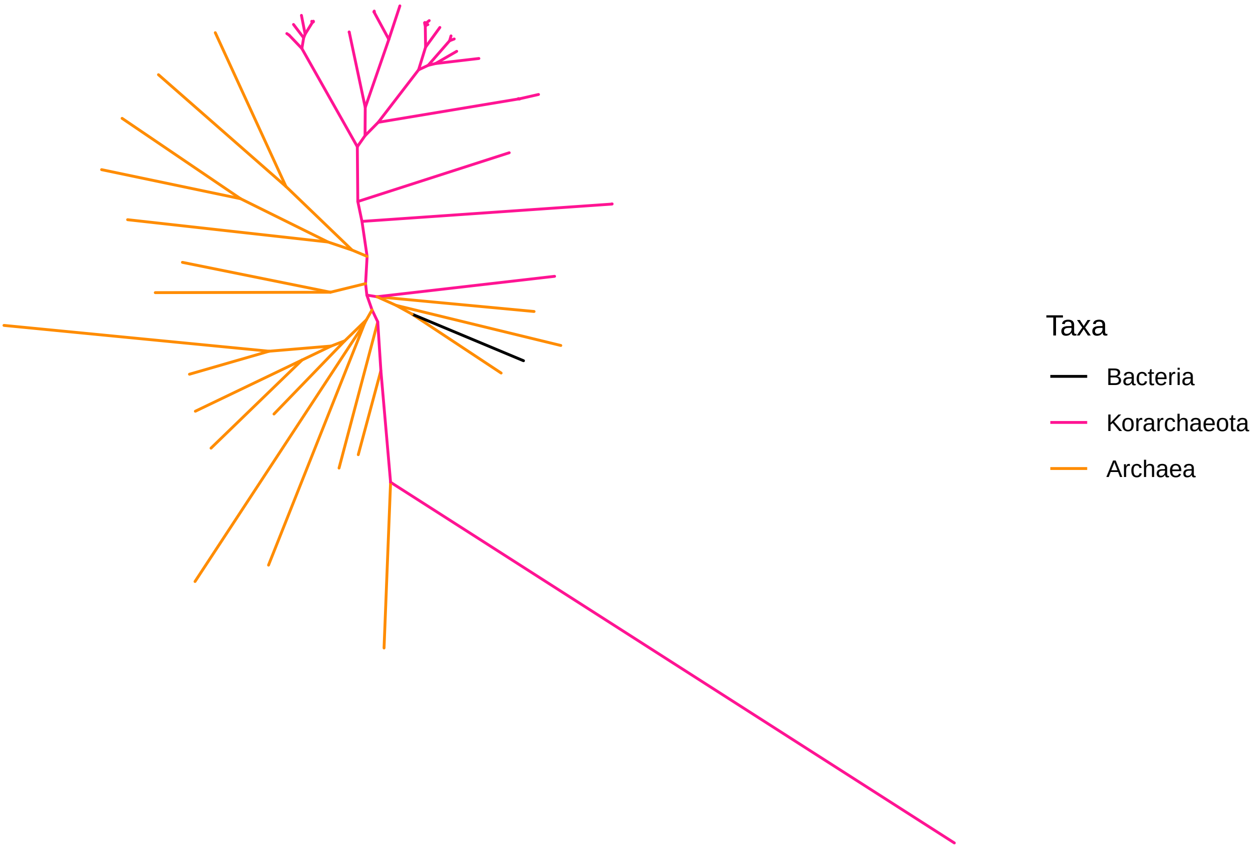

Kor−HOG0000738

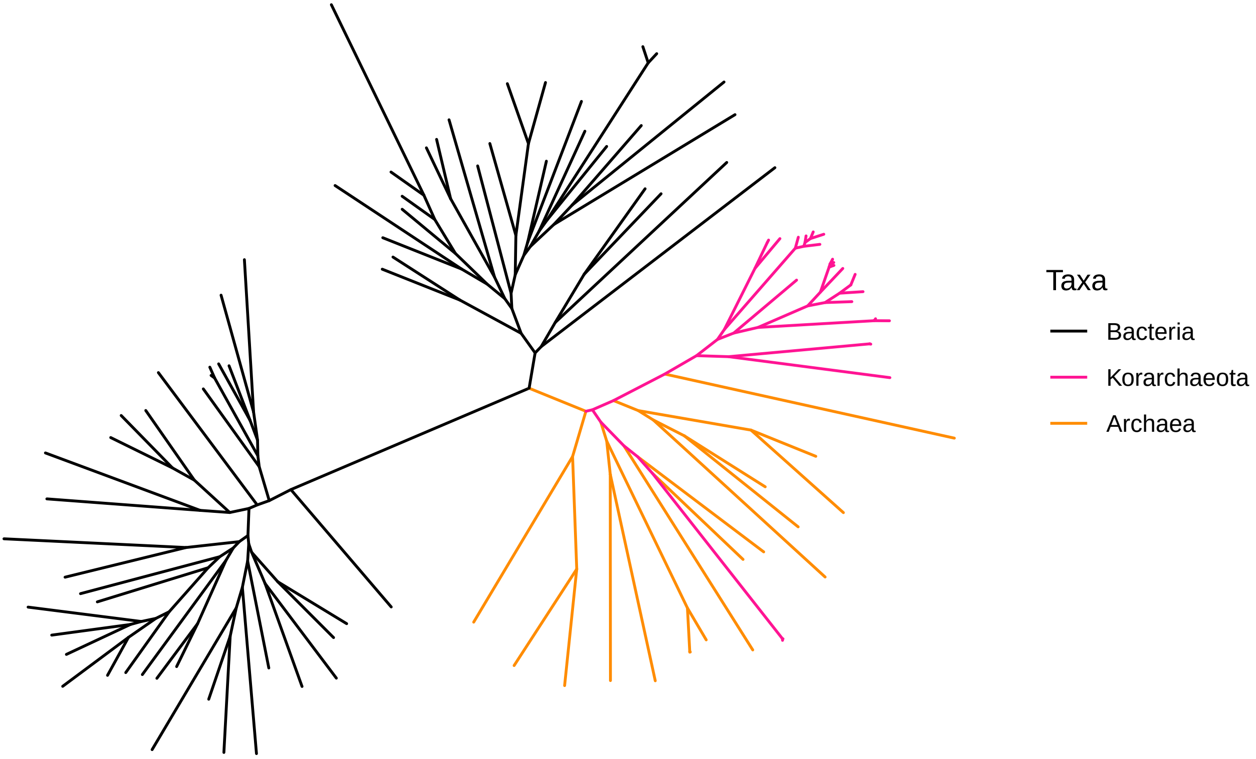

Kor−HOG0000766

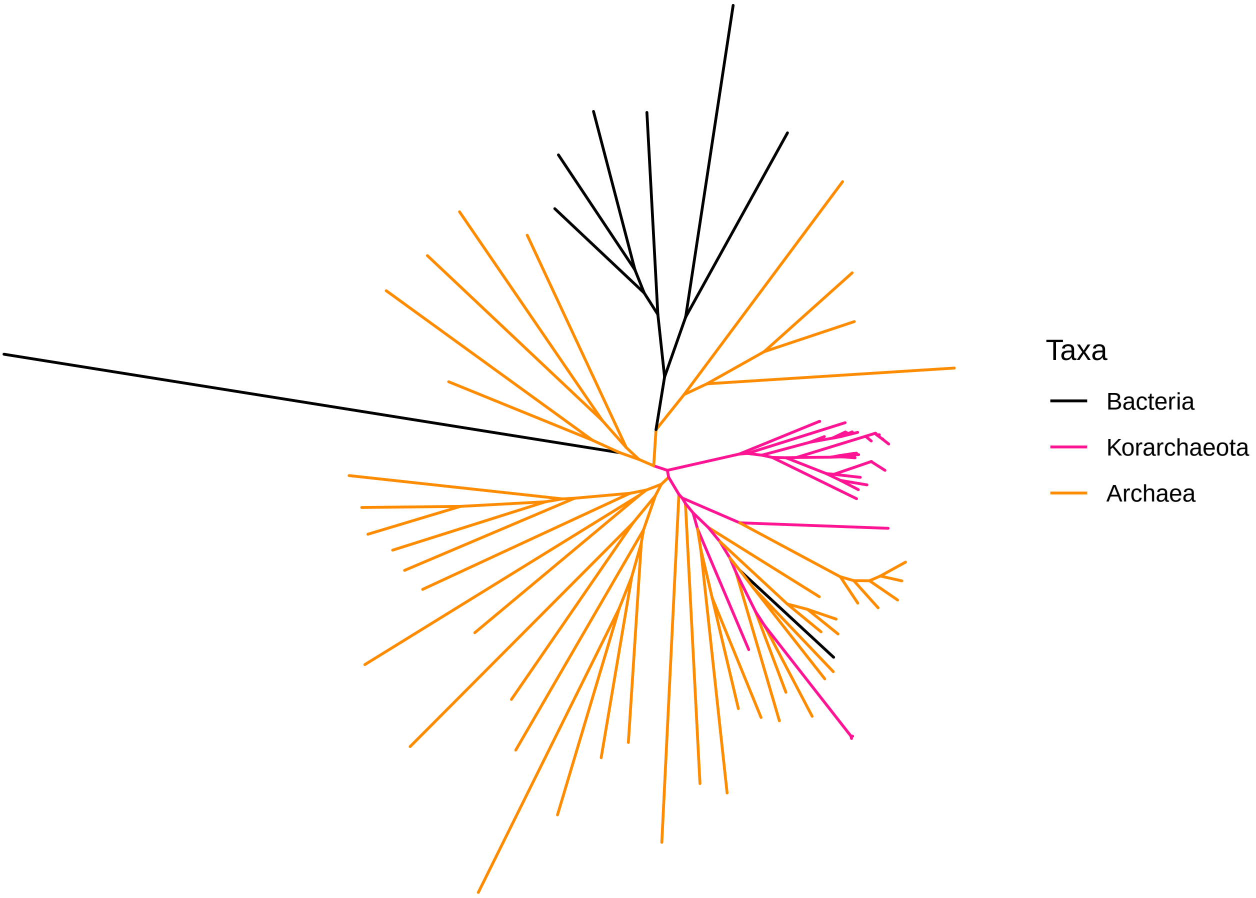

Kor−HOG0000768

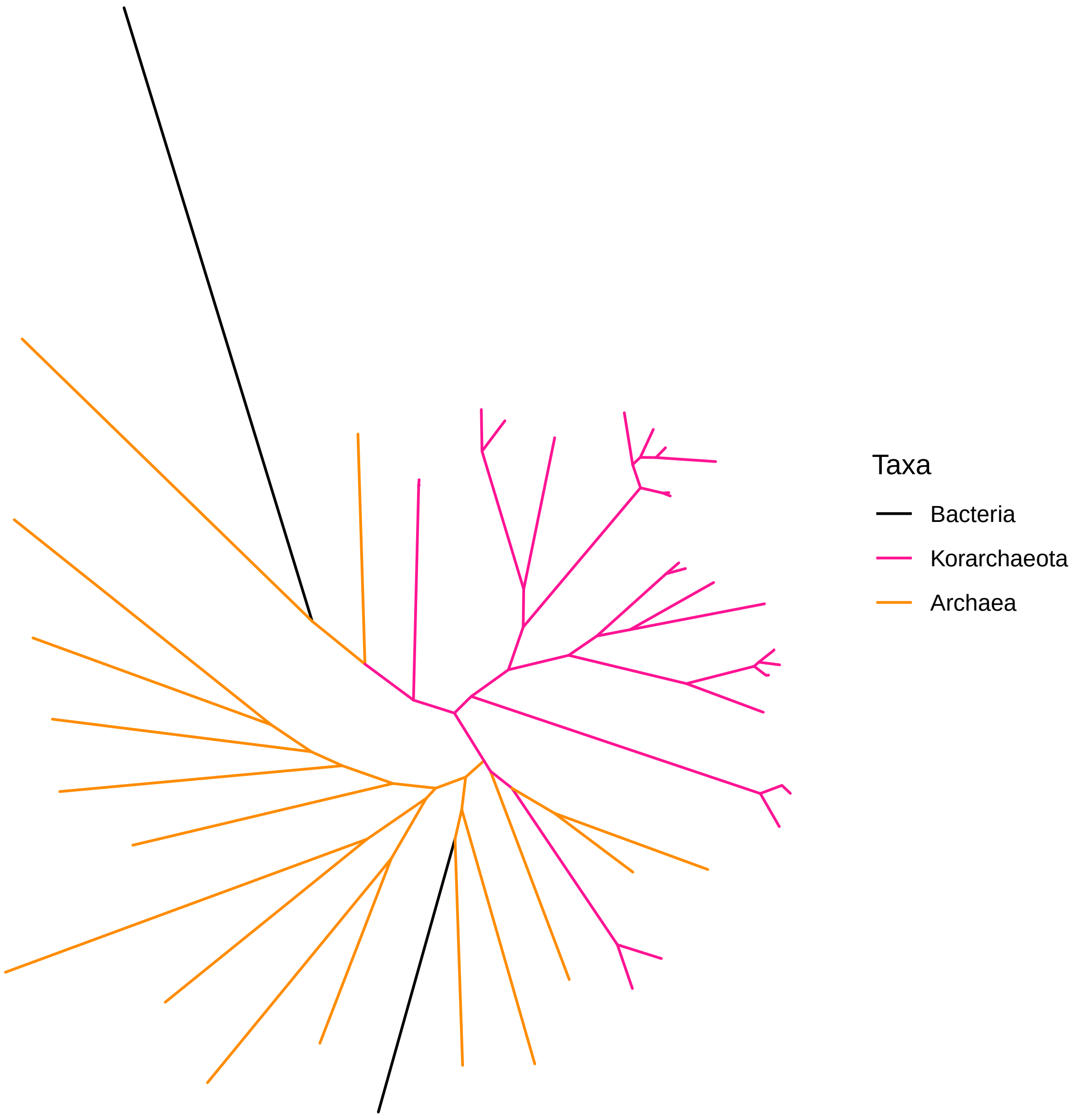

Kor−HOG0000778

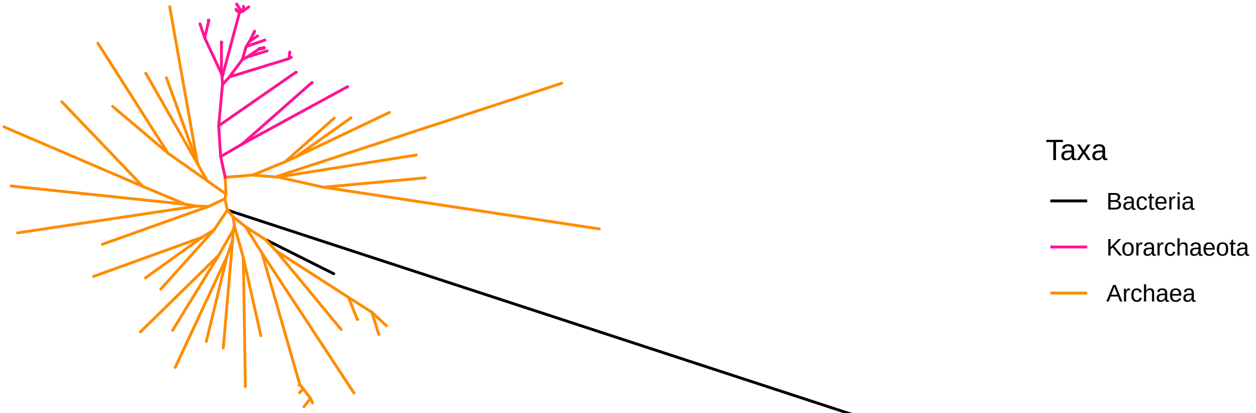

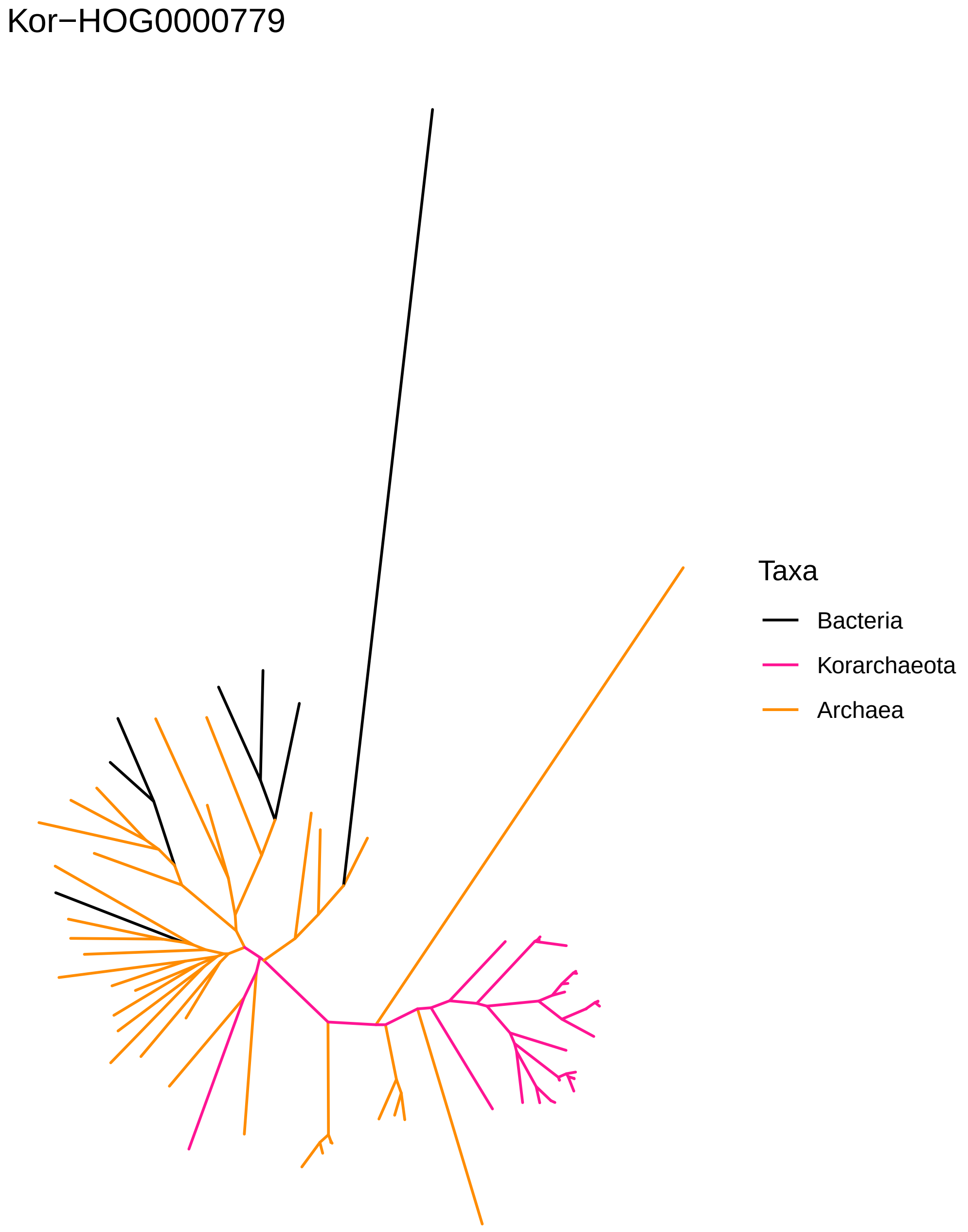
Kor−HOG0000779
Taxa
Bacteria
Korarchaeota
Archaea

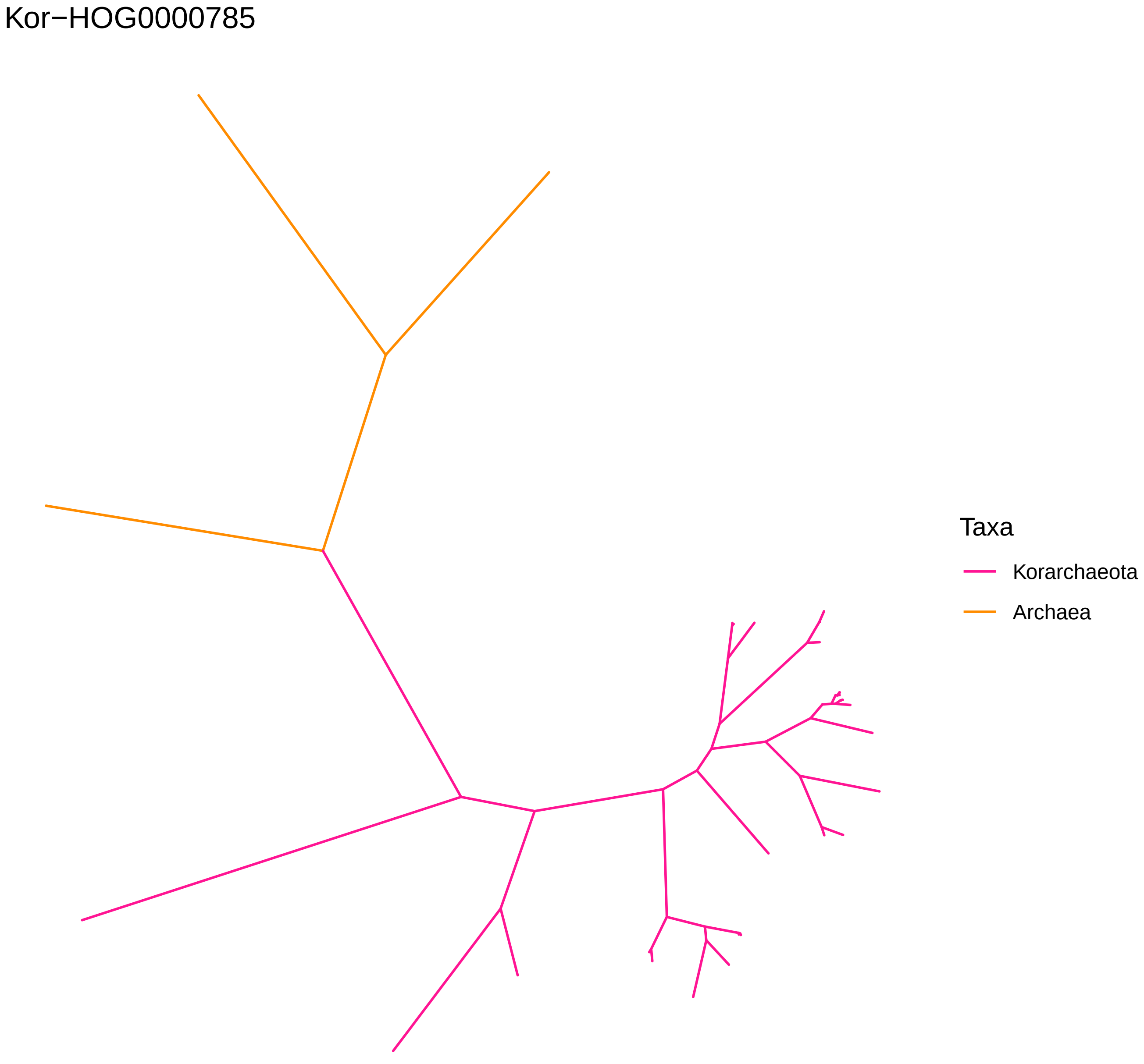

Kor−HOG0000785
Taxa
Korarchaeota
Archaea

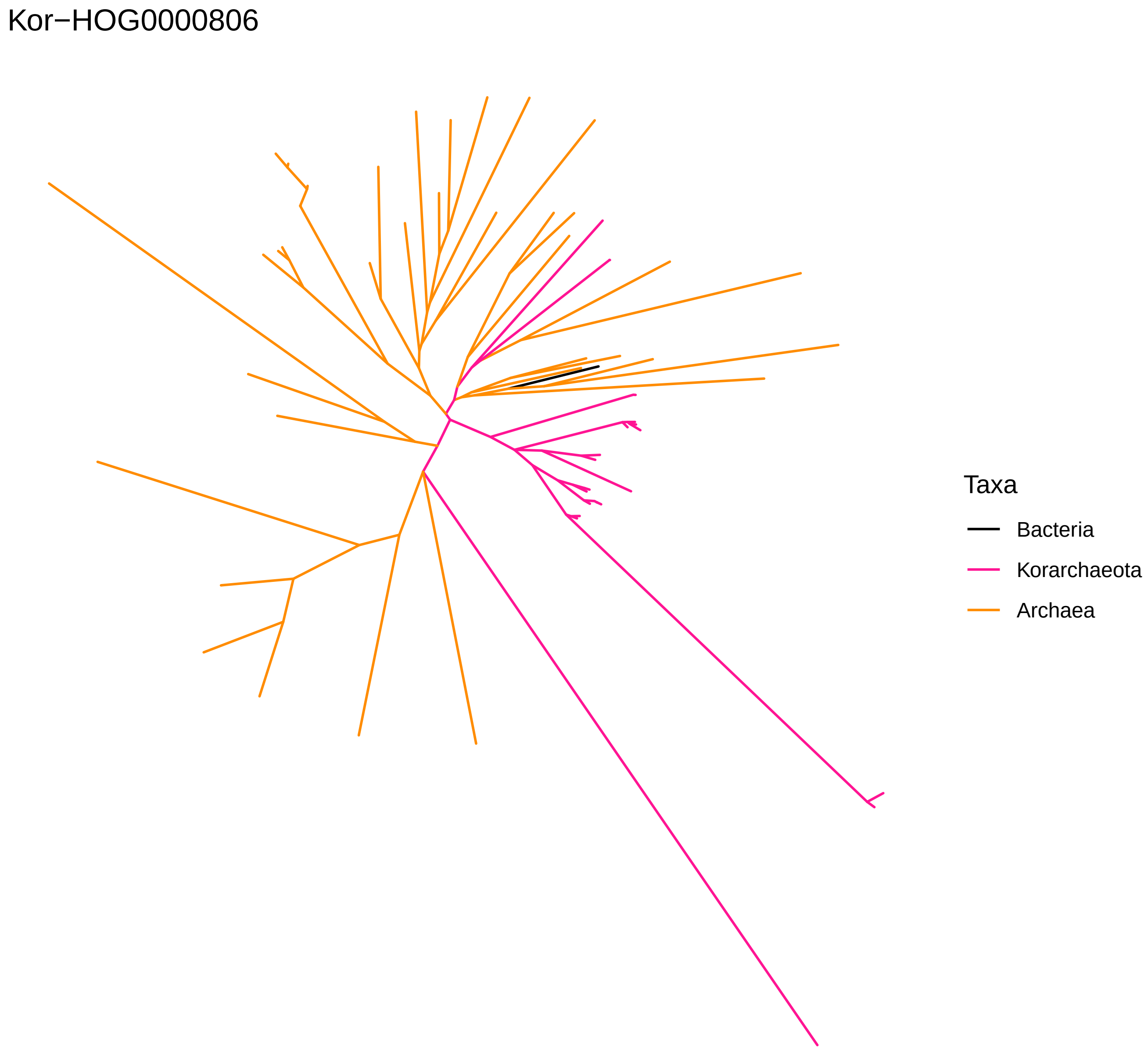
Kor−HOG0000806
Taxa
Bacteria
Korarchaeota
Archaea

Kor−HOG0000808

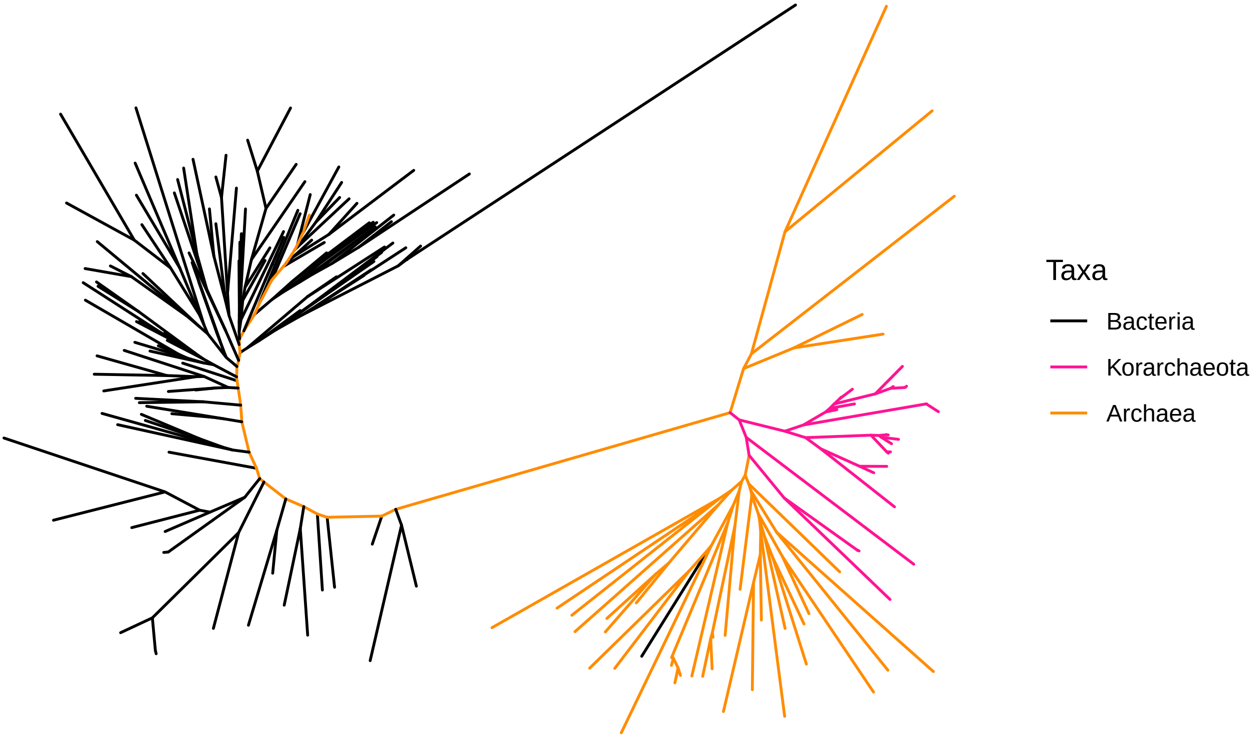

Kor−HOG0000811

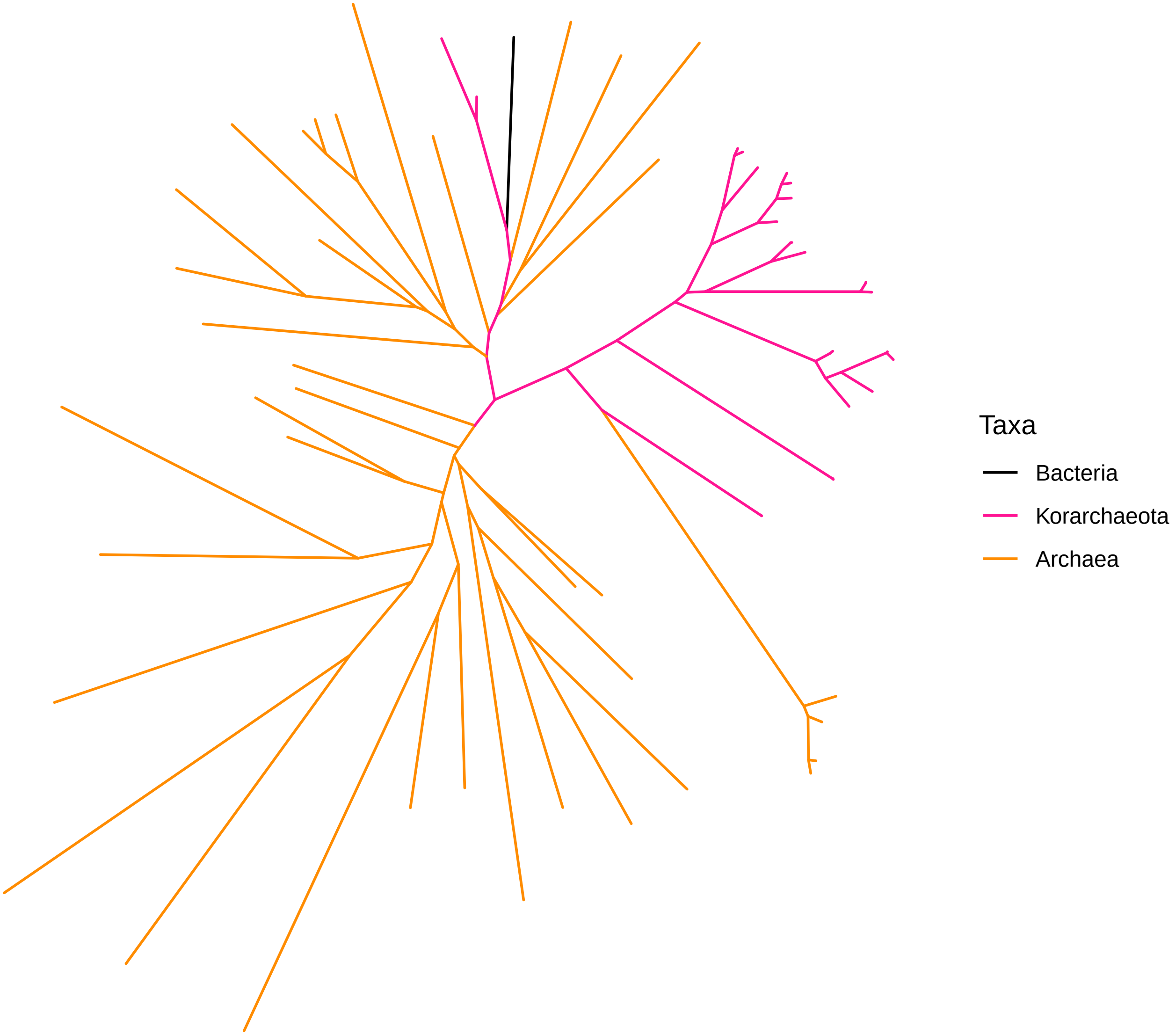

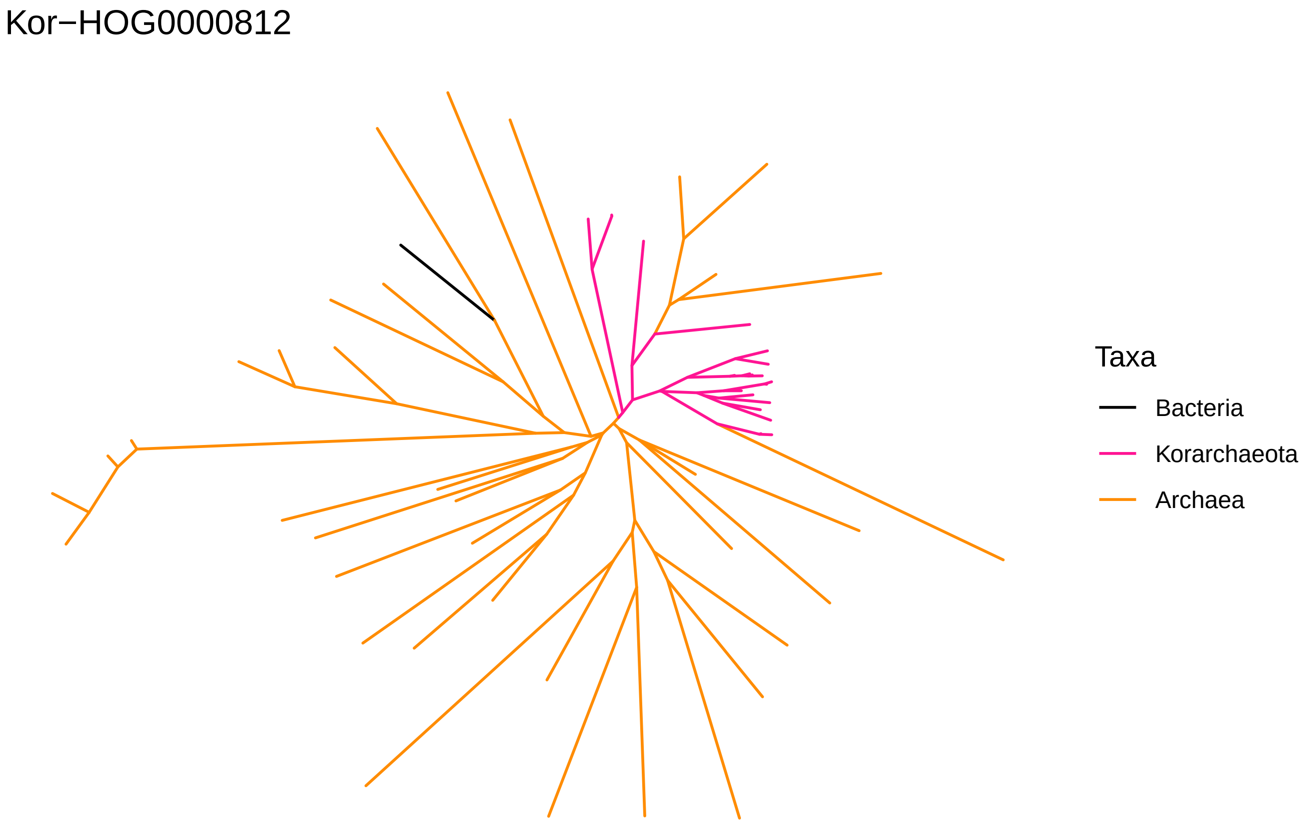
Kor−HOG0000812
Taxa
Bacteria
Korarchaeota
Archaea

Kor−HOG0000813

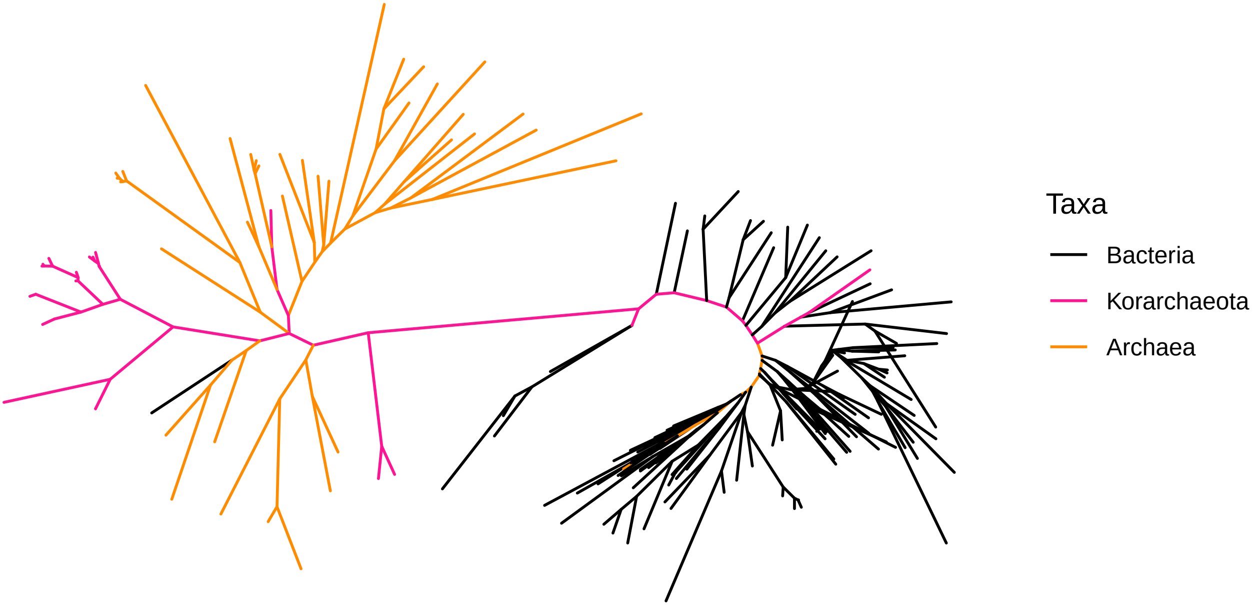

Kor−HOG0000825

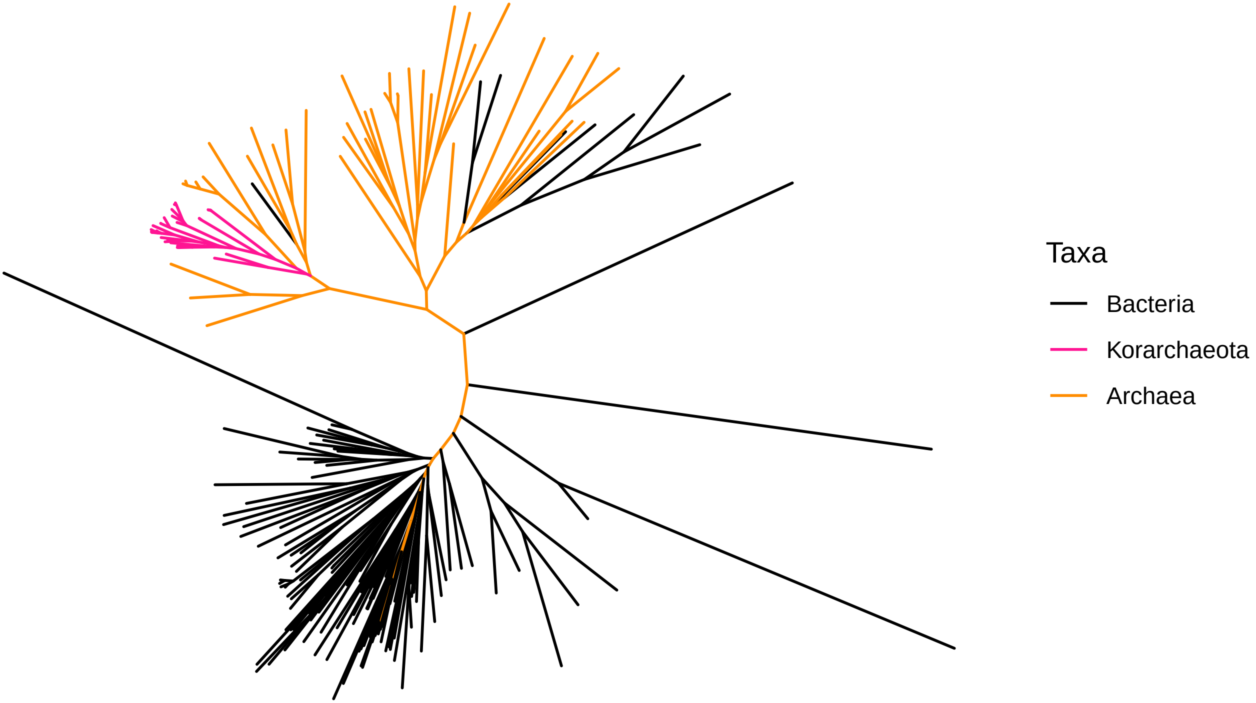

Kor−HOG0000827

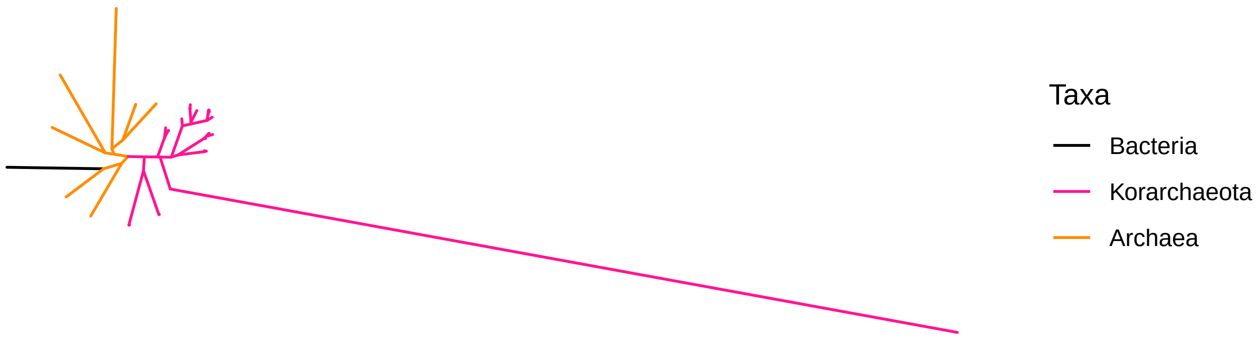

Kor−HOG0000828

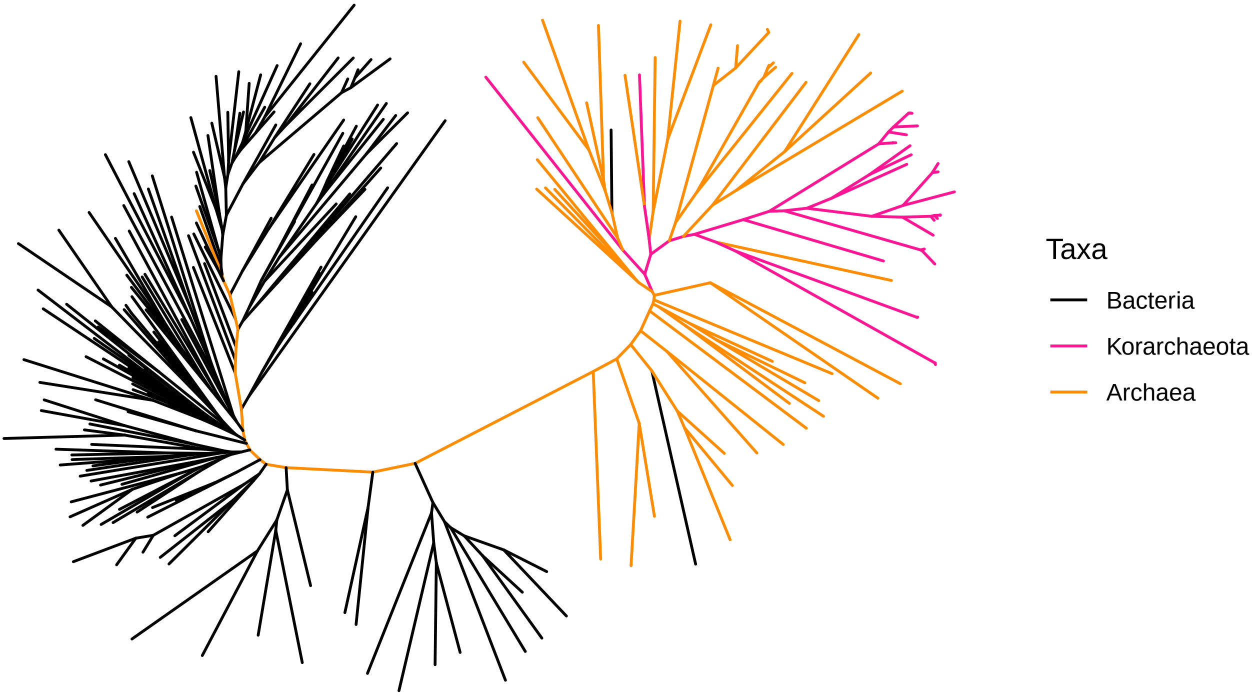

Kor−HOG0000832

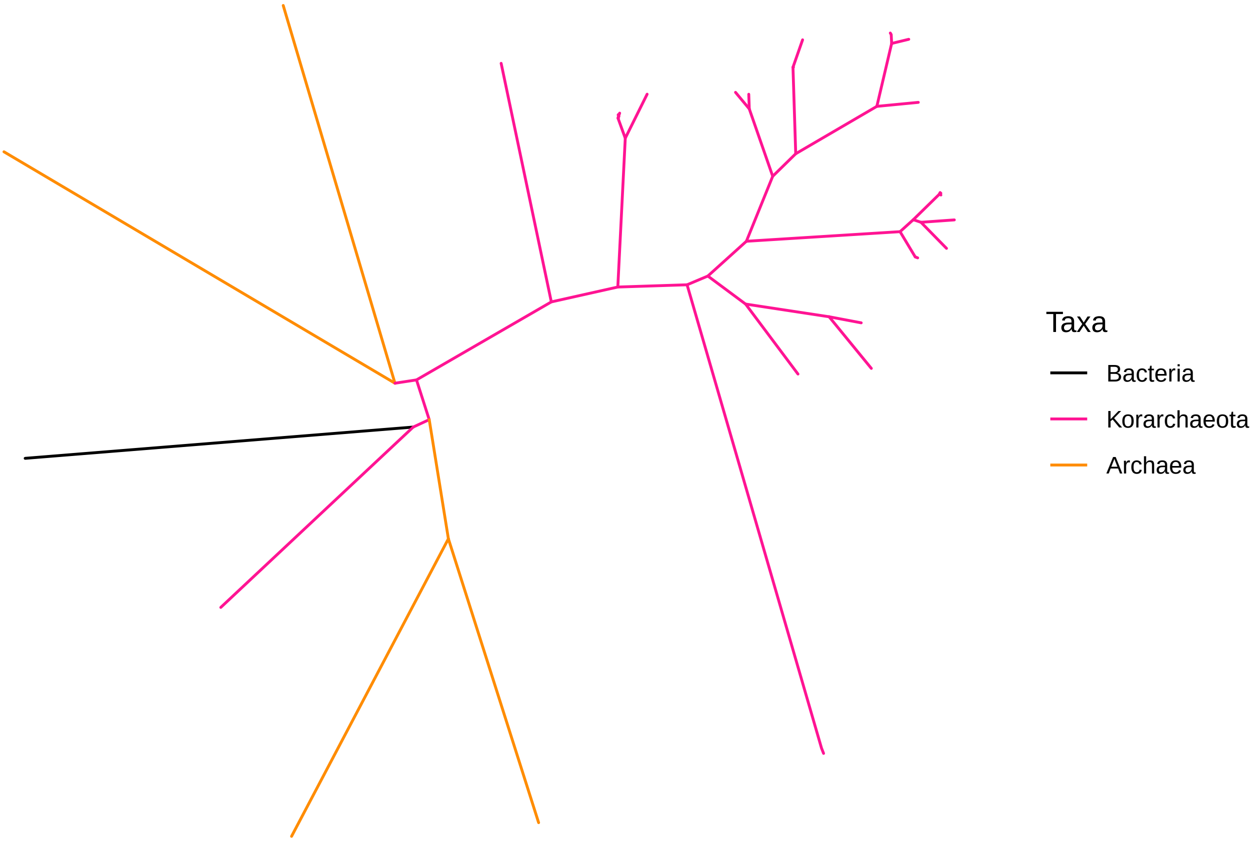

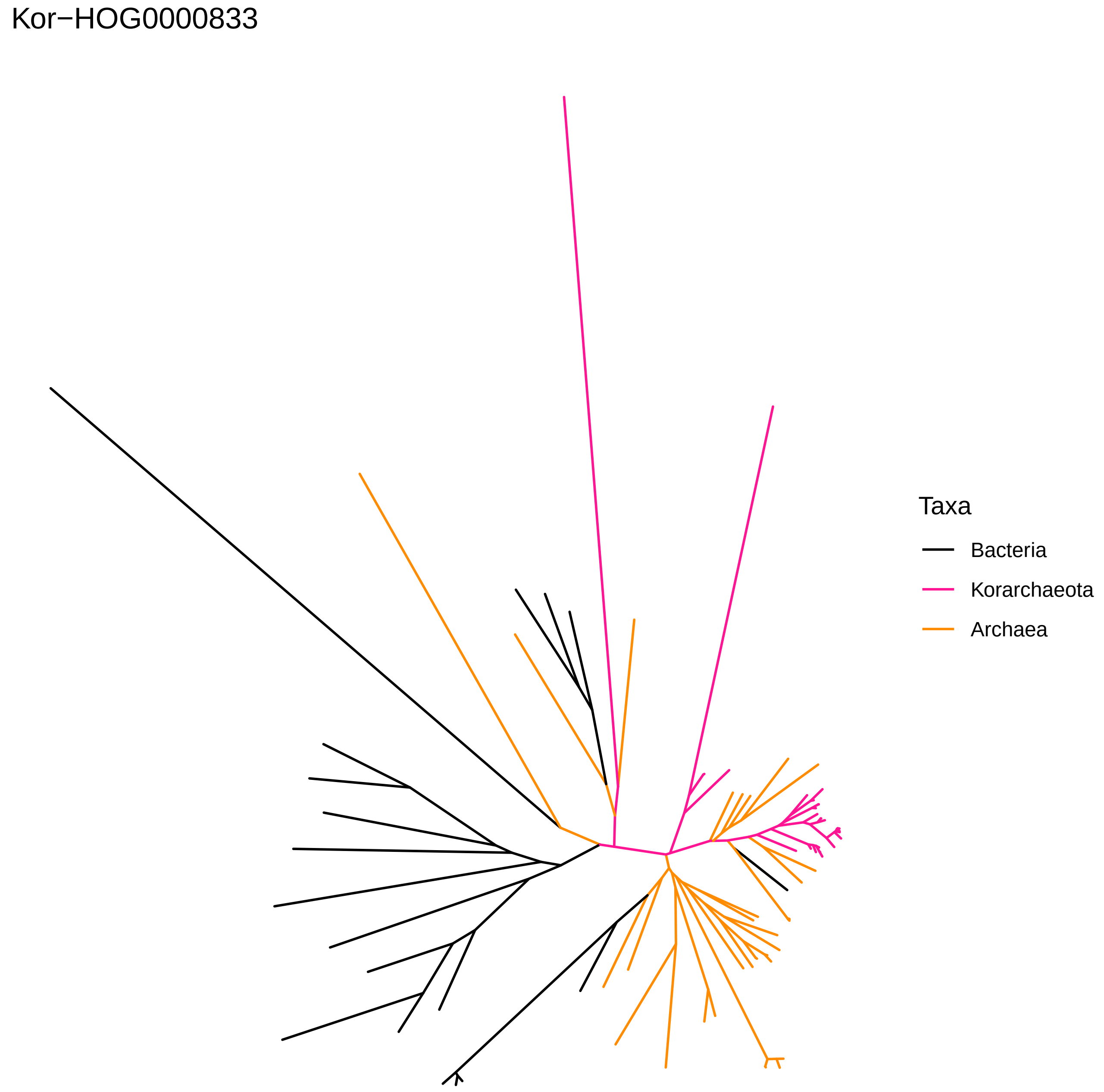

Kor−HOG0000833
Taxa
Bacteria
Korarchaeota
Archaea

Kor−HOG0000834

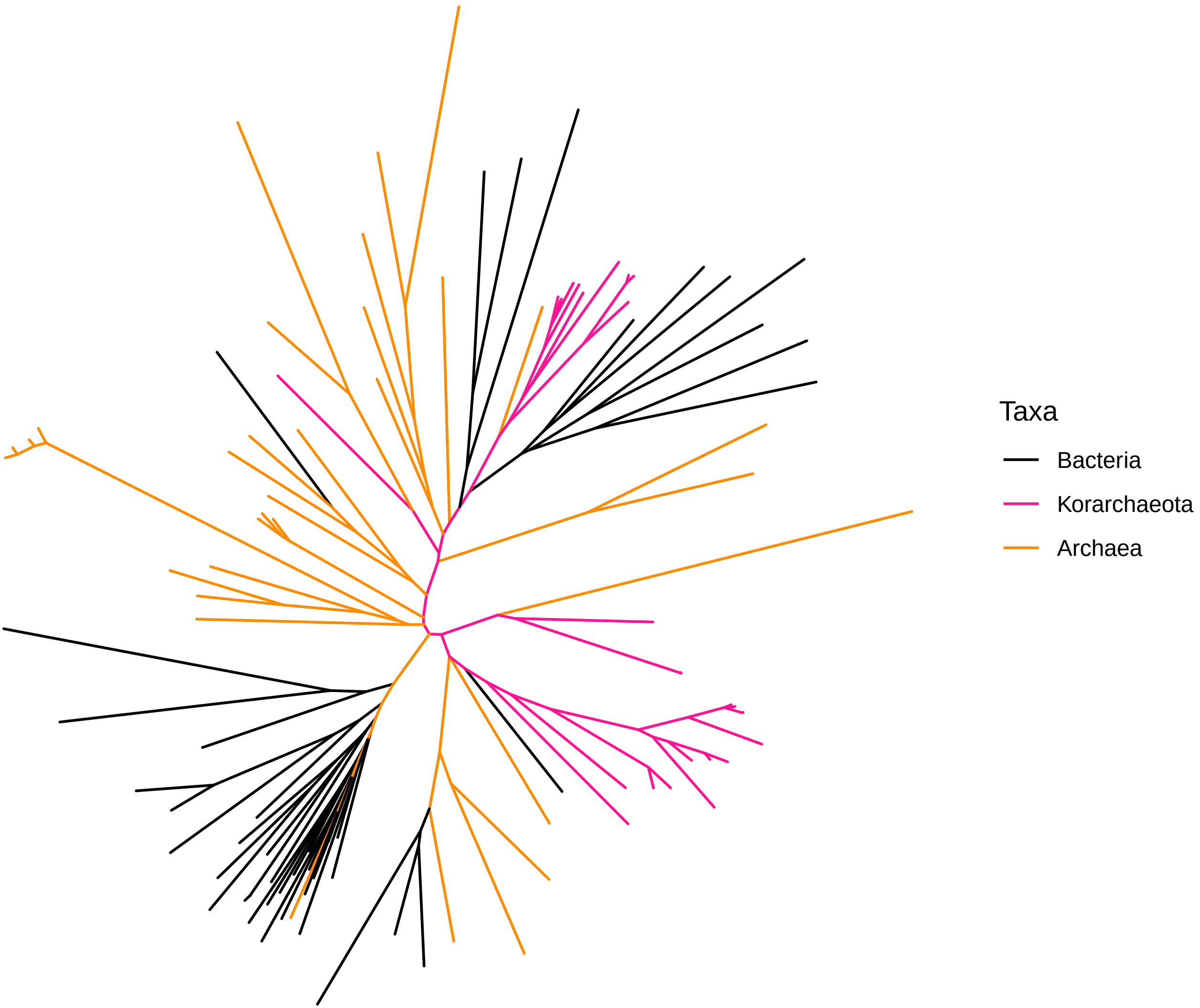

Kor−HOG0000852

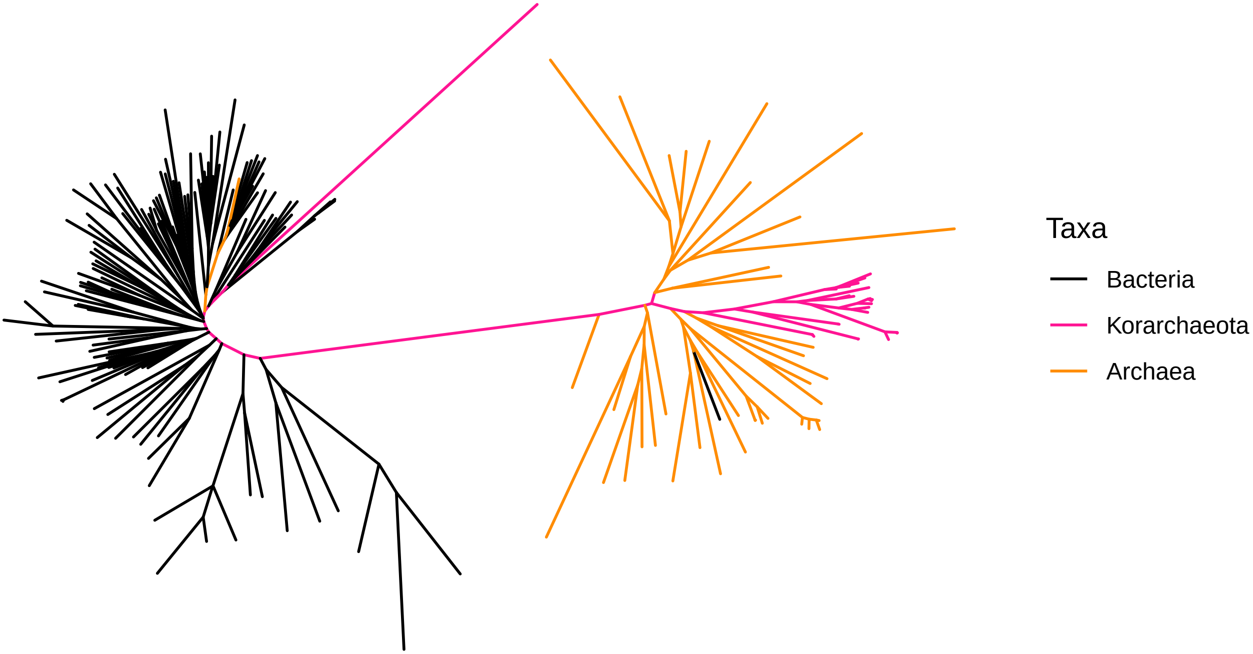

Kor−HOG0000859

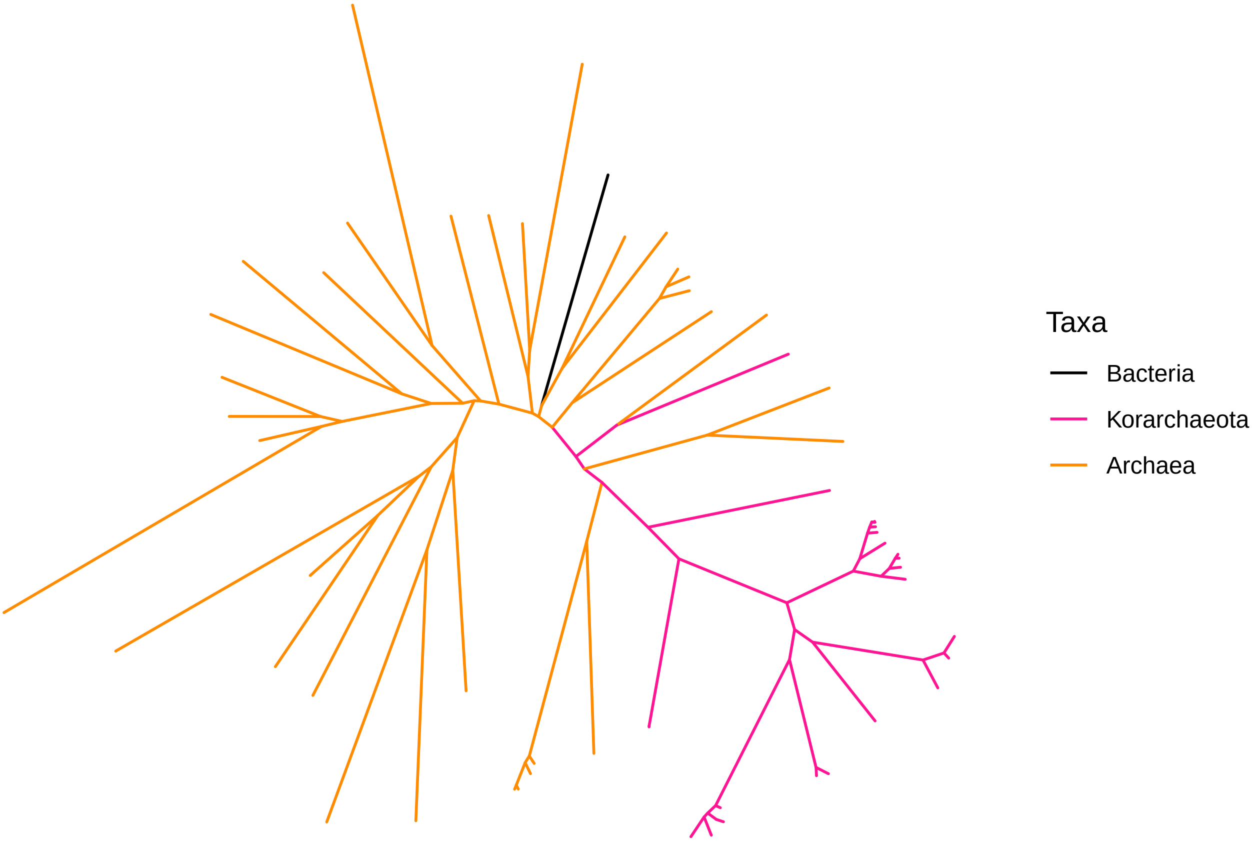

Kor−HOG0000865

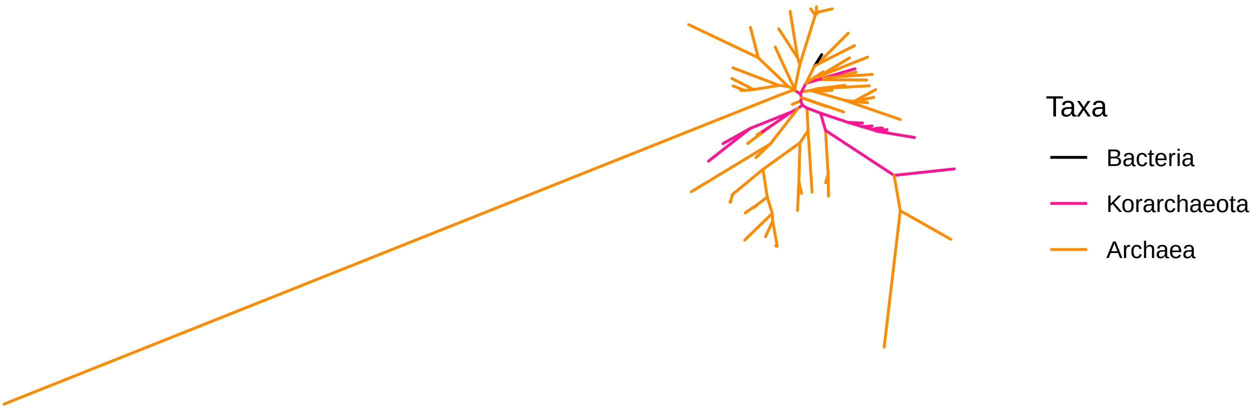

Kor−HOG0000869

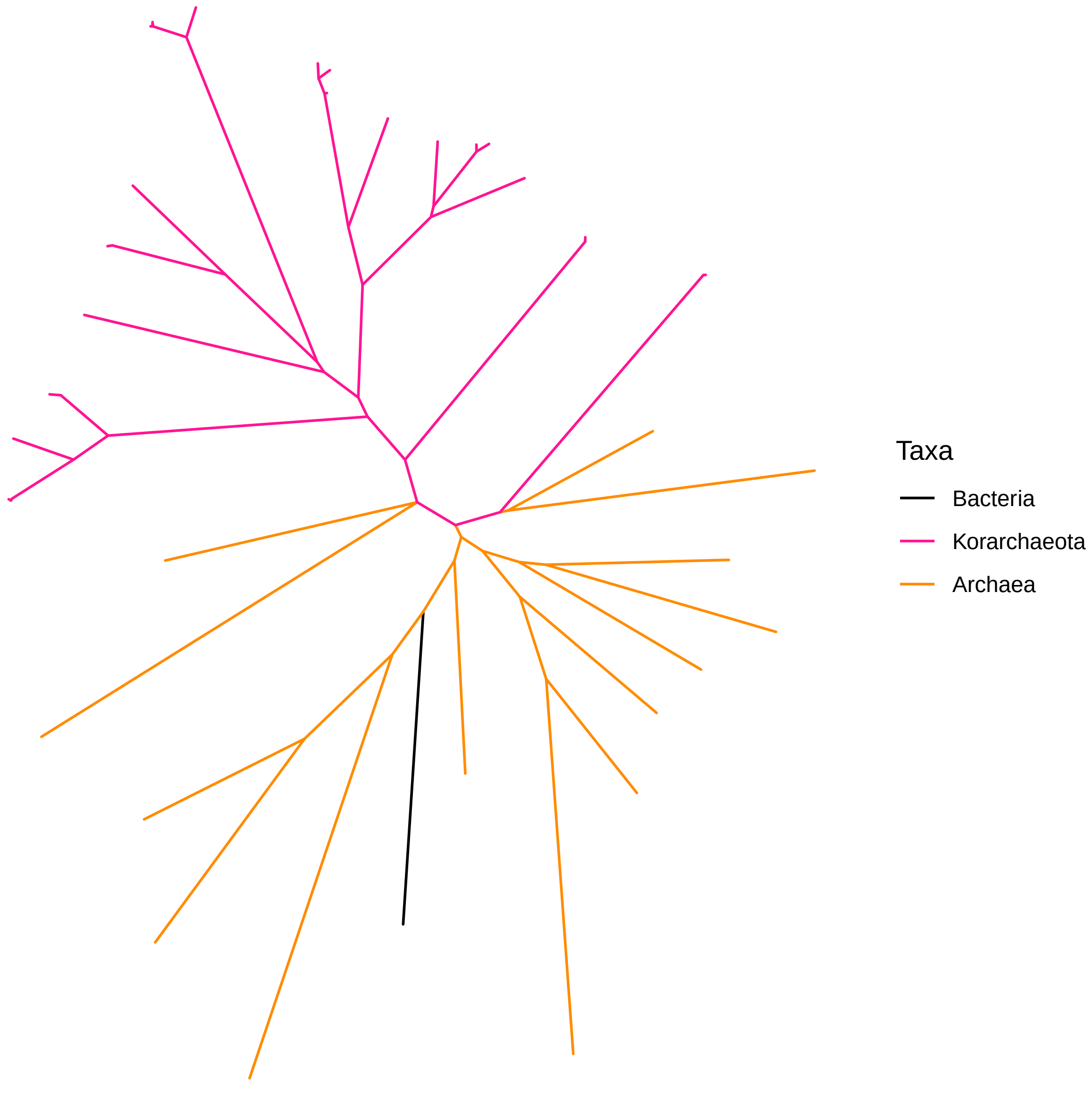

Kor−HOG0000871

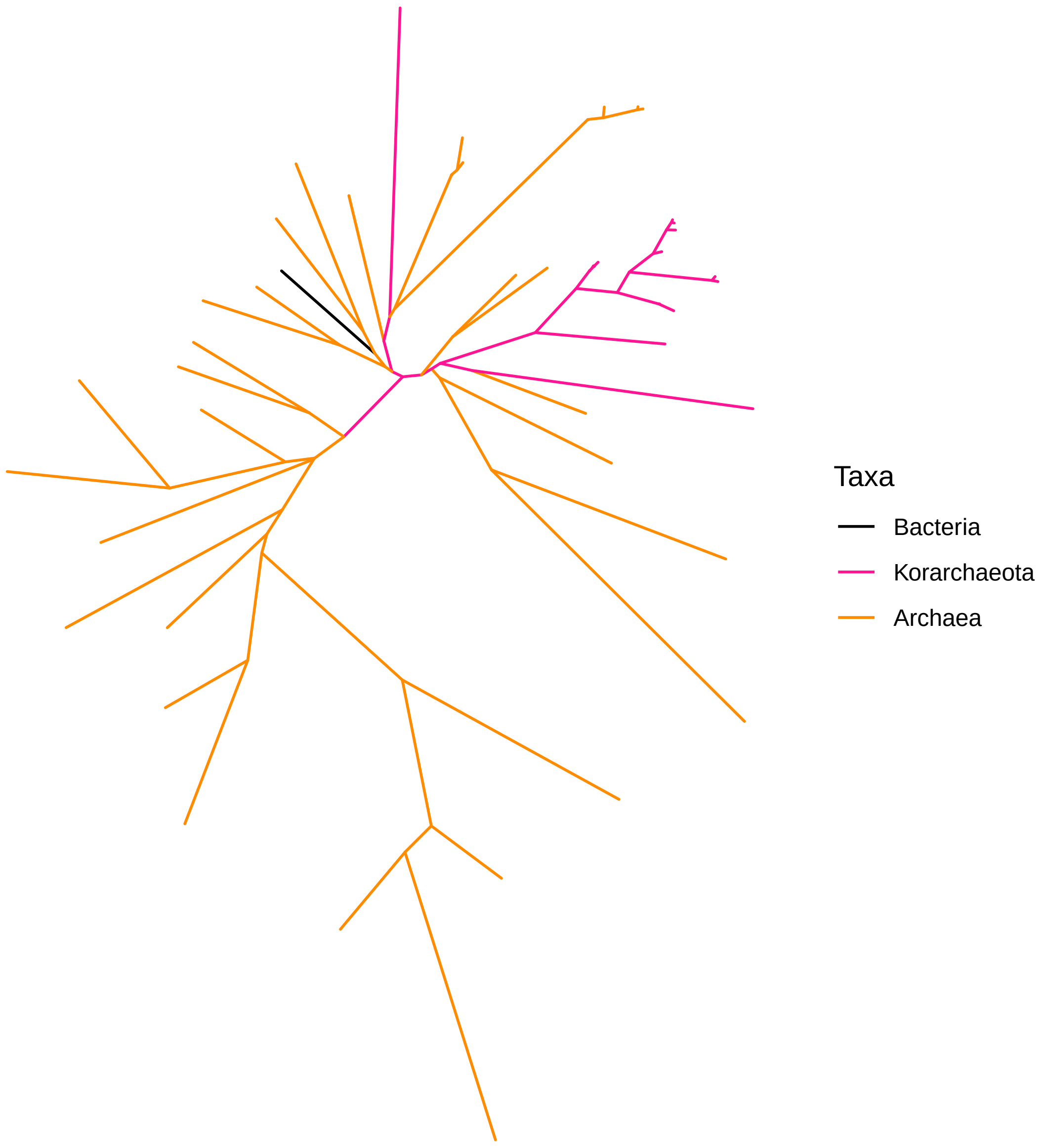

Kor−HOG0000872

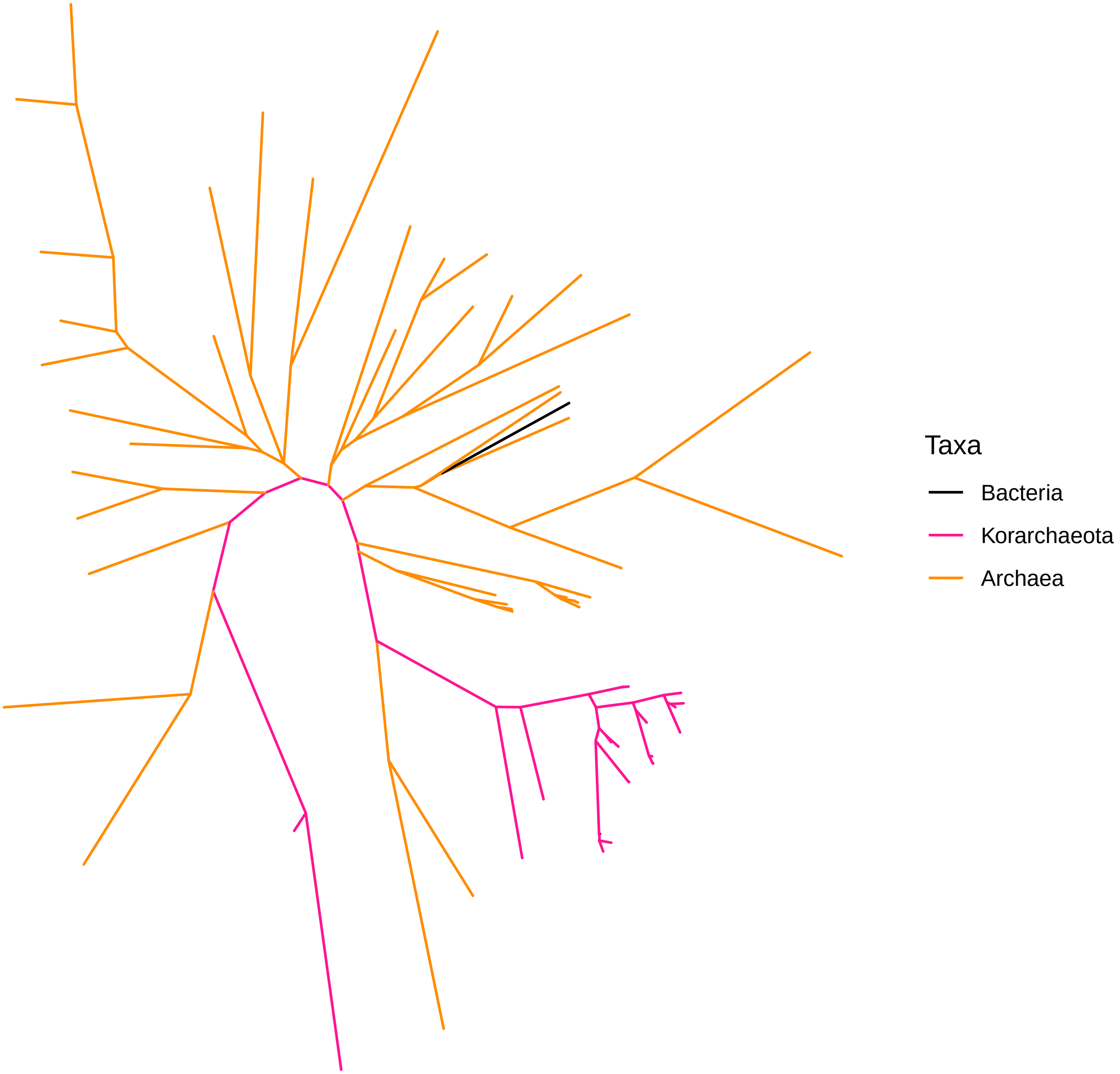

Kor−HOG0000875

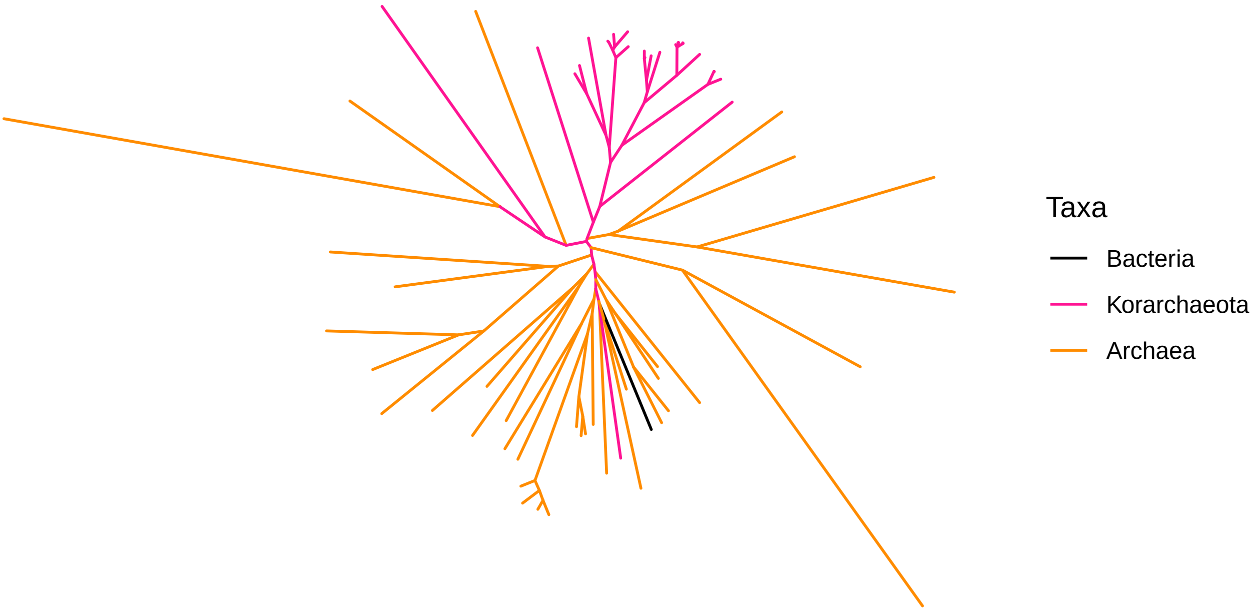

Kor−HOG0000876

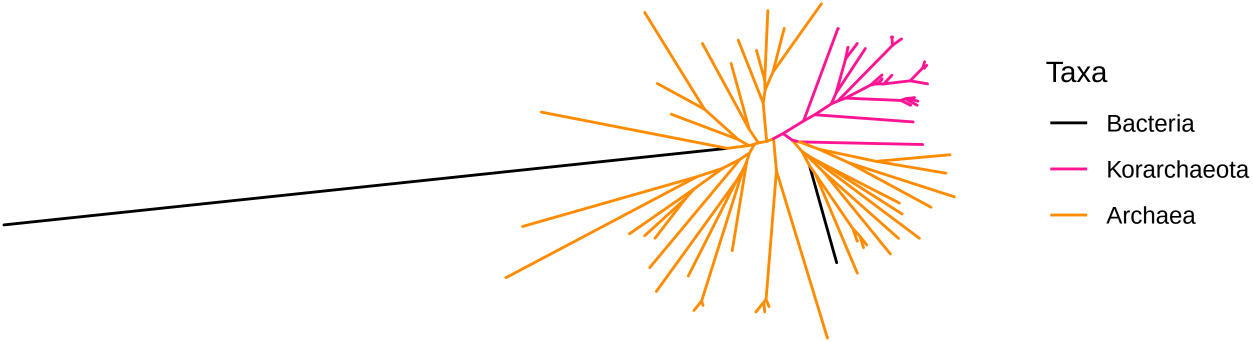

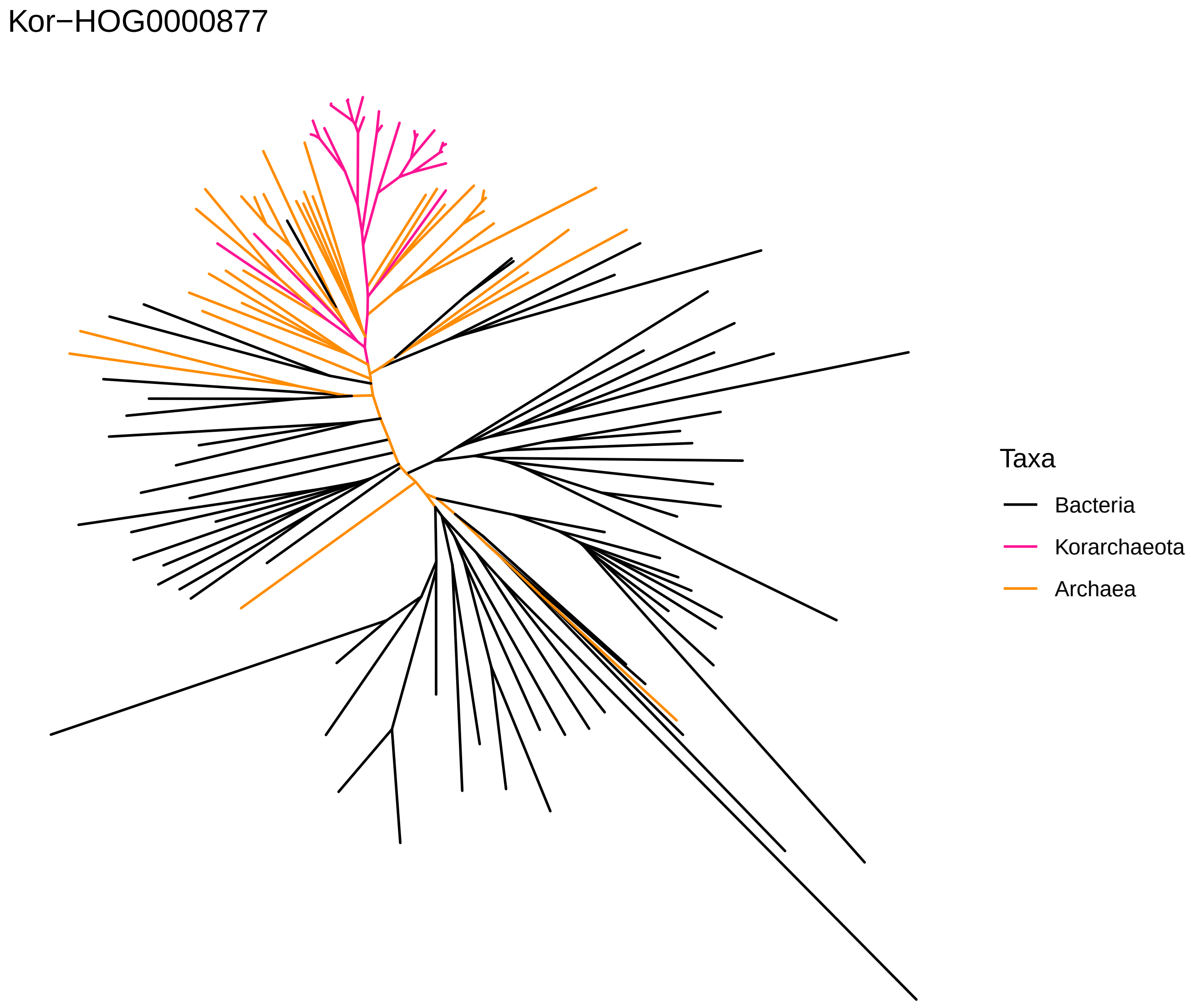
Kor−HOG0000877
Taxa
Bacteria
Korarchaeota
Archaea

Kor−HOG0000878

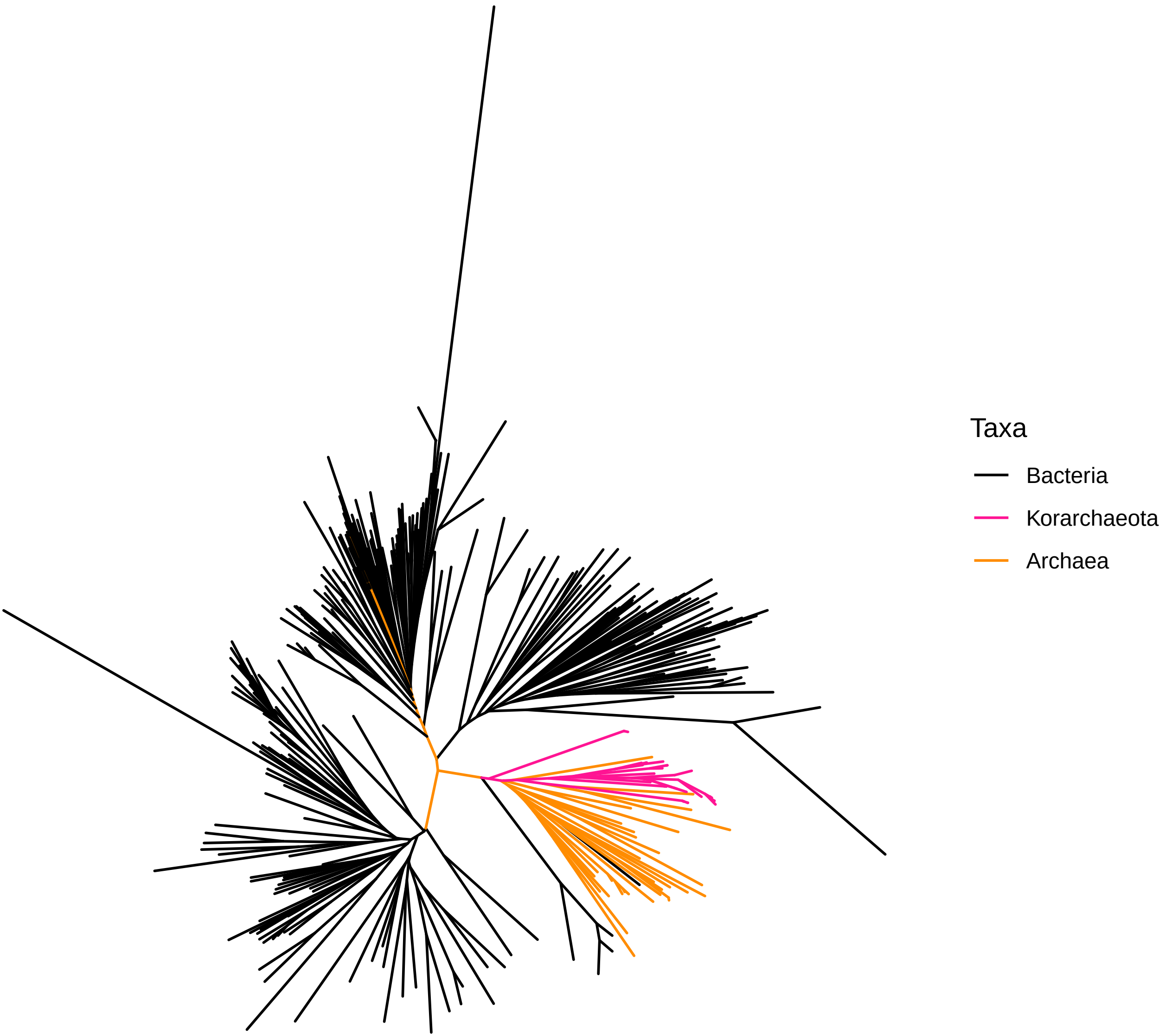

Kor−HOG0000879

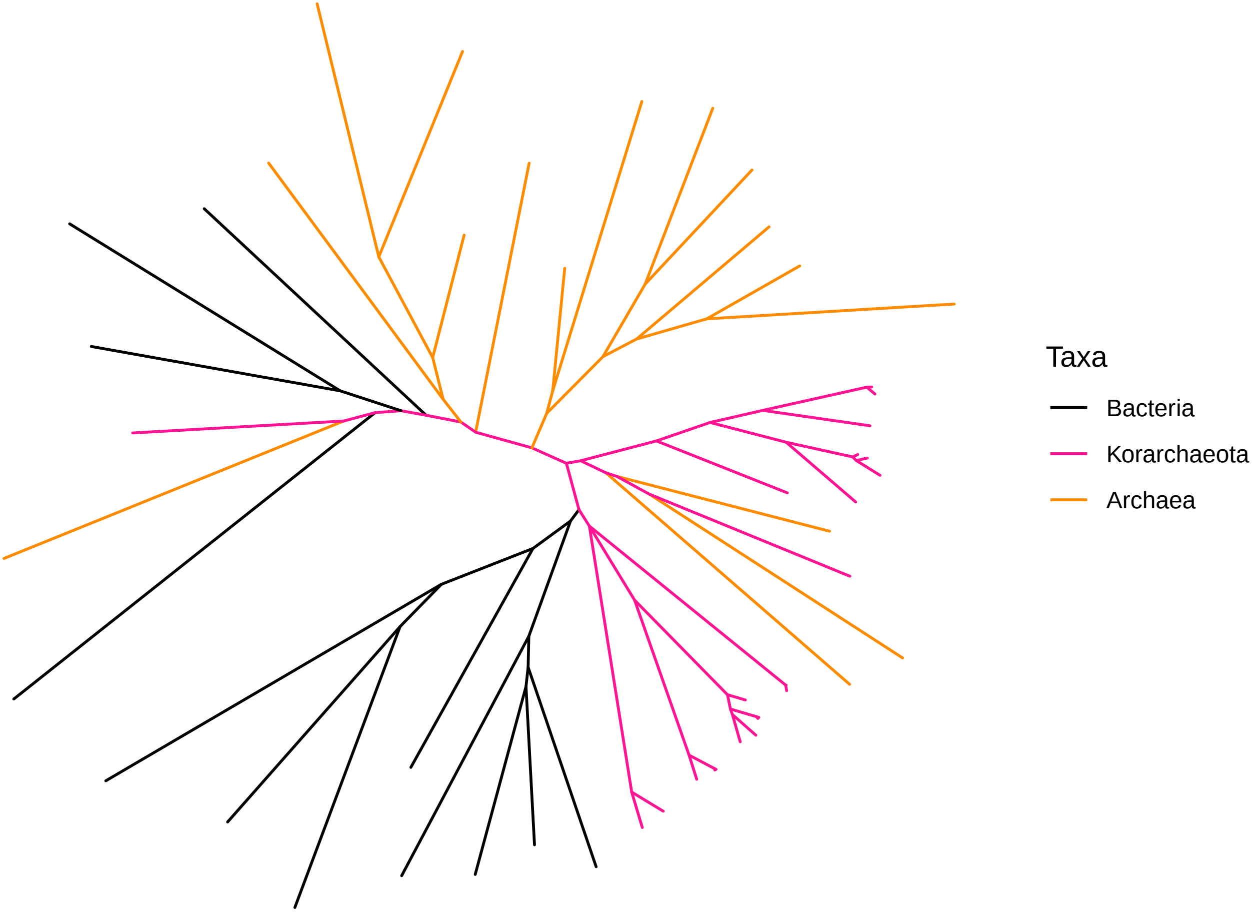

Kor−HOG0000881

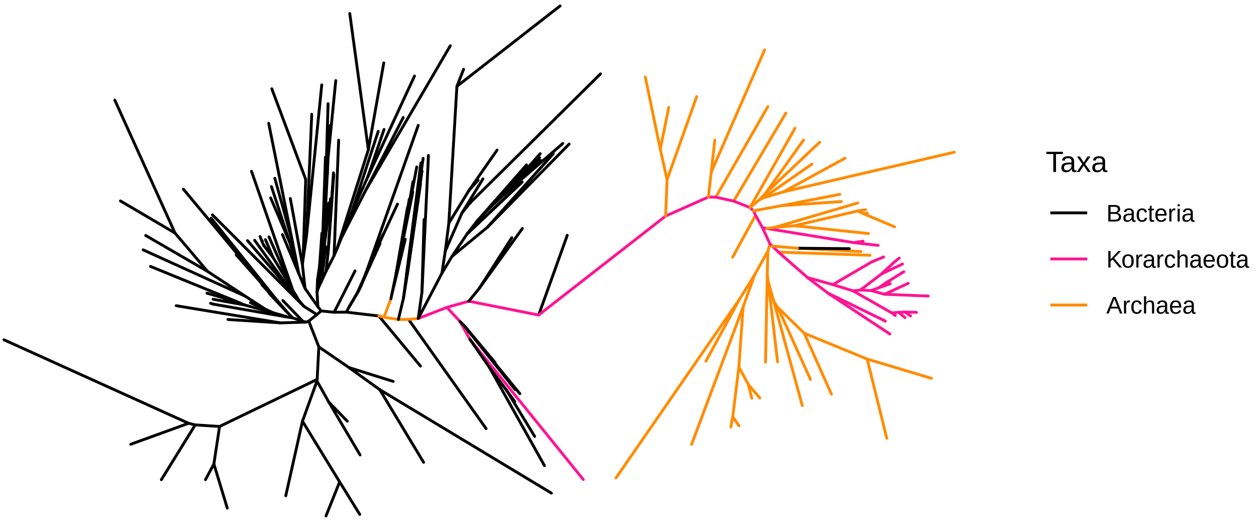

Kor−HOG0000882

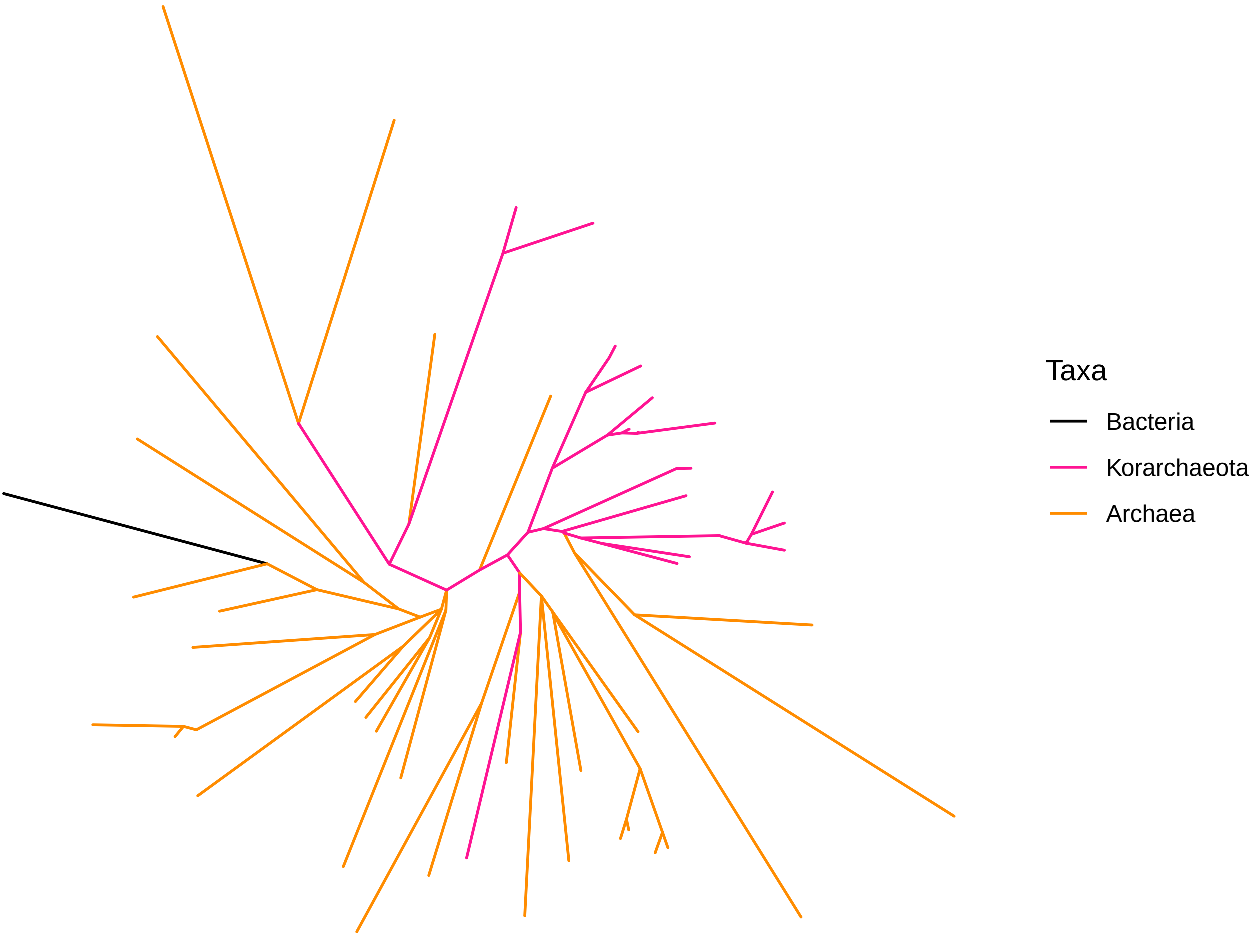

Kor−HOG0000883

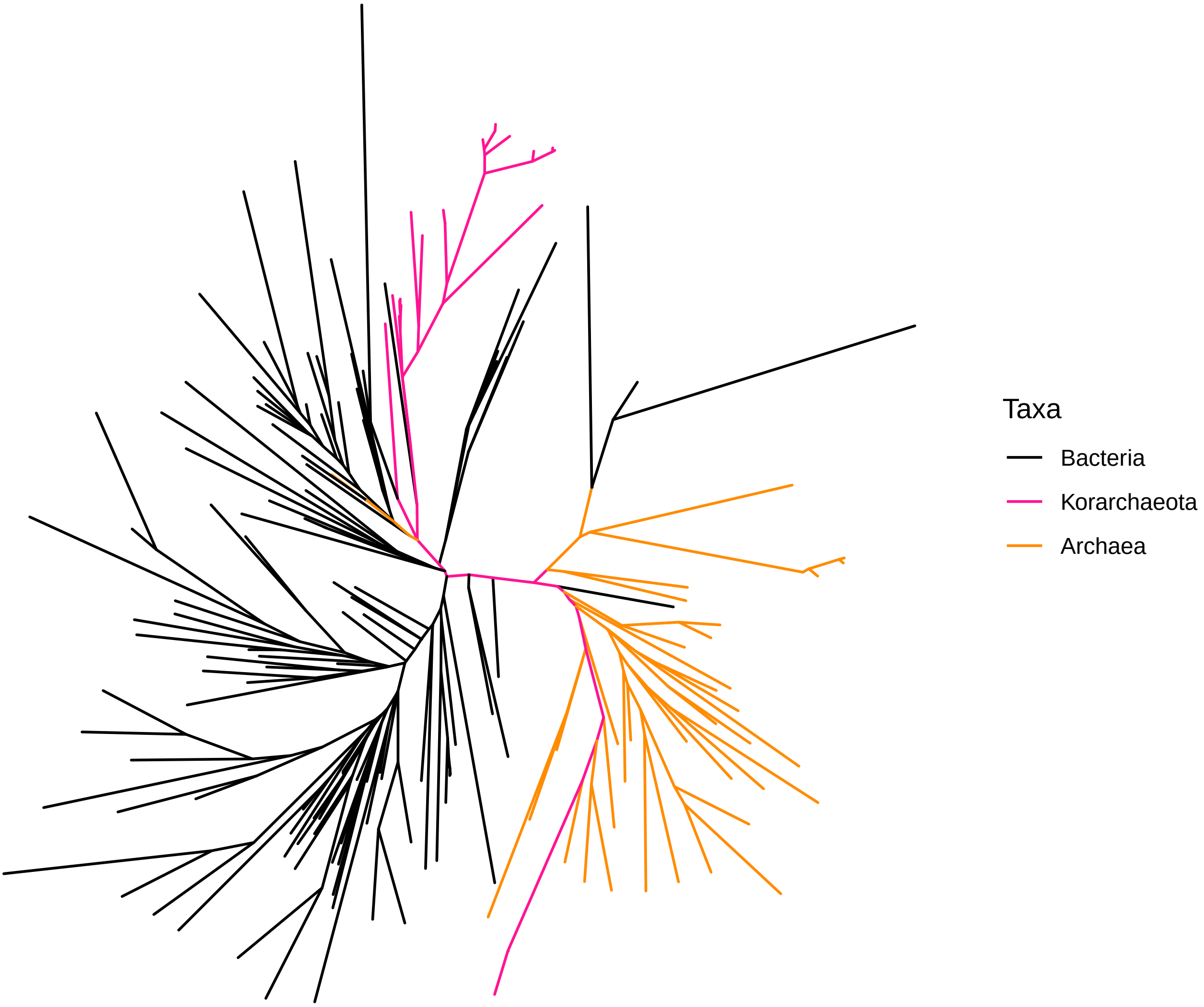

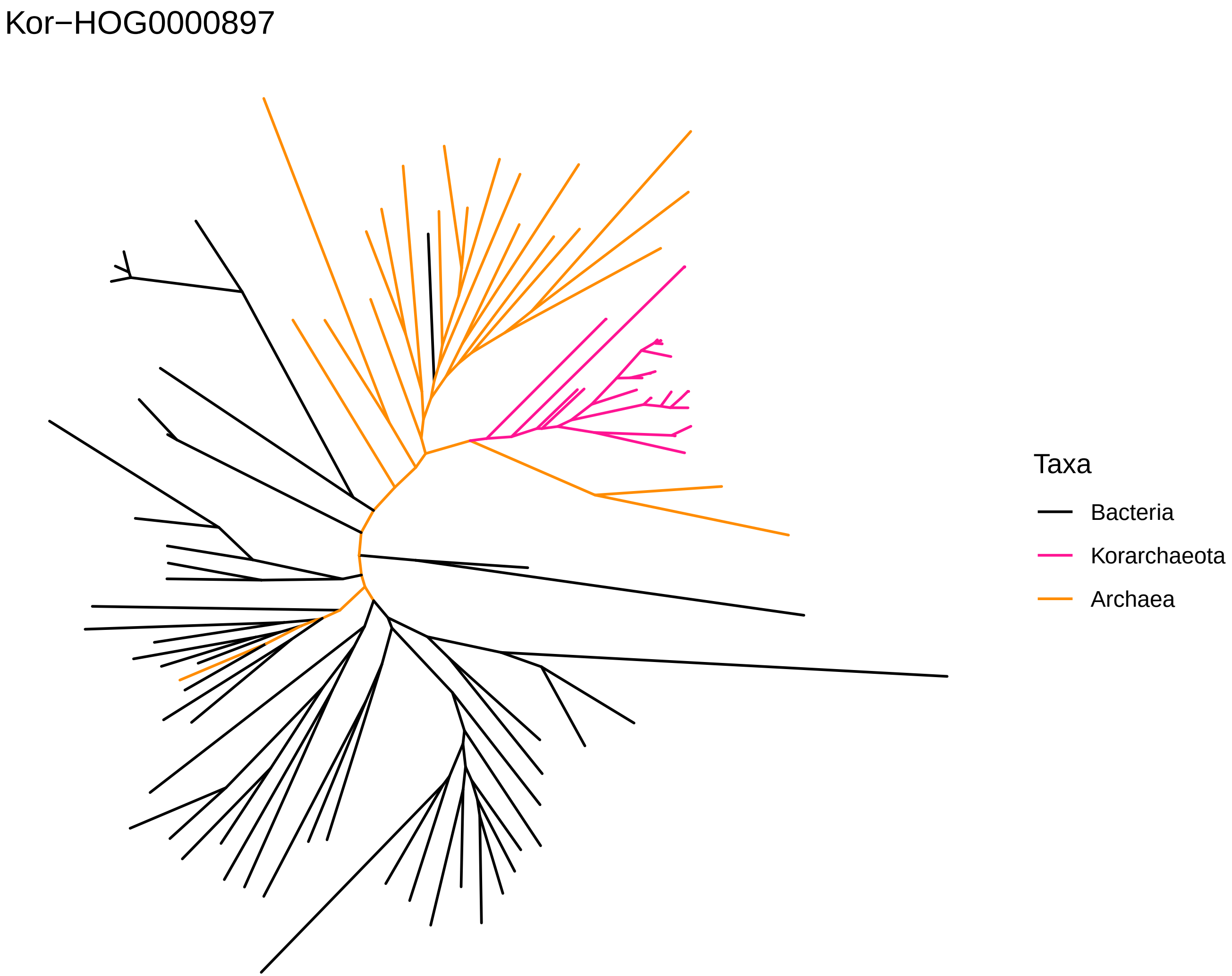
Kor−HOG0000897
Taxa
Bacteria
Korarchaeota
Archaea

Kor−HOG0000898

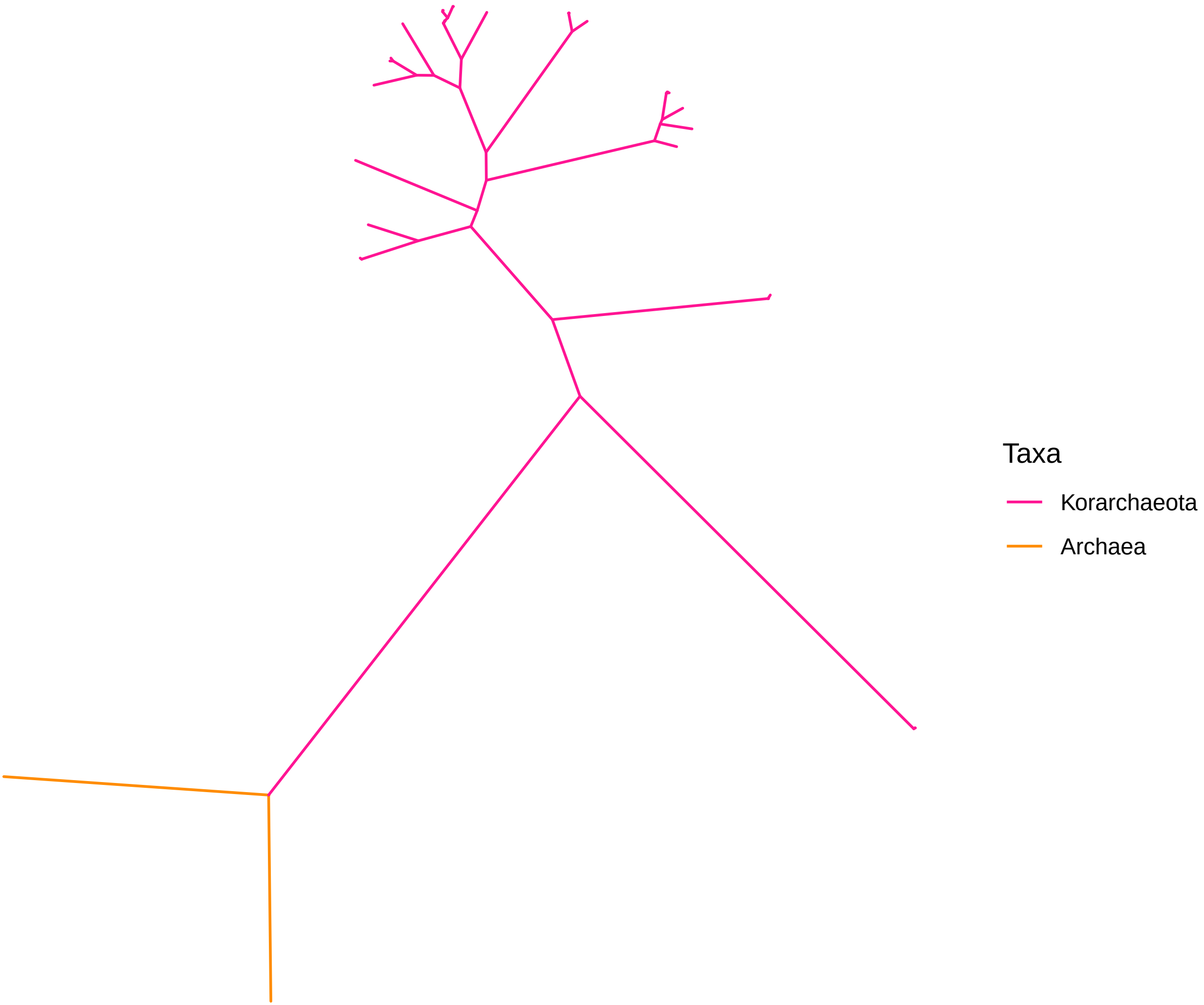

Kor−HOG0000905

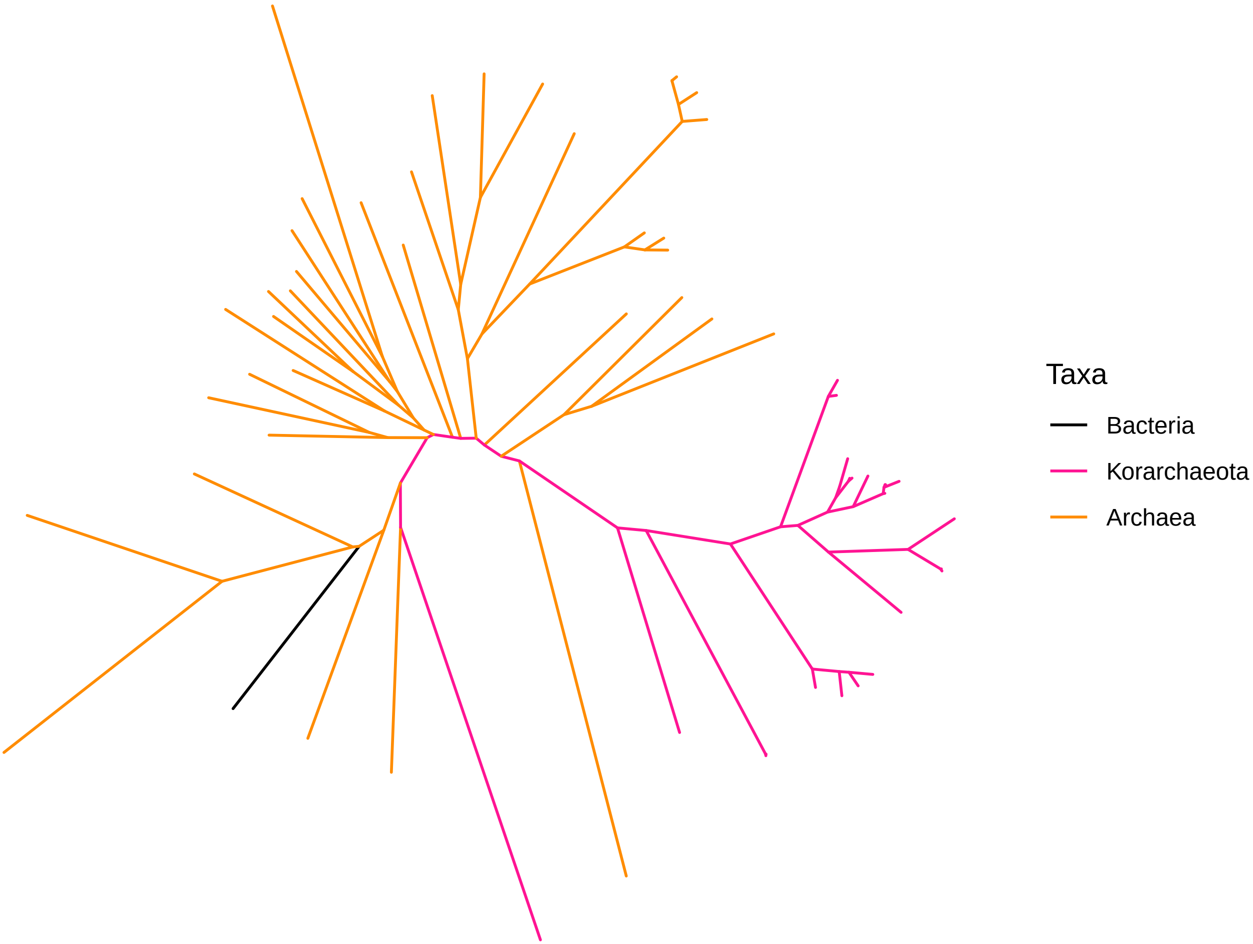

Kor−HOG0000911

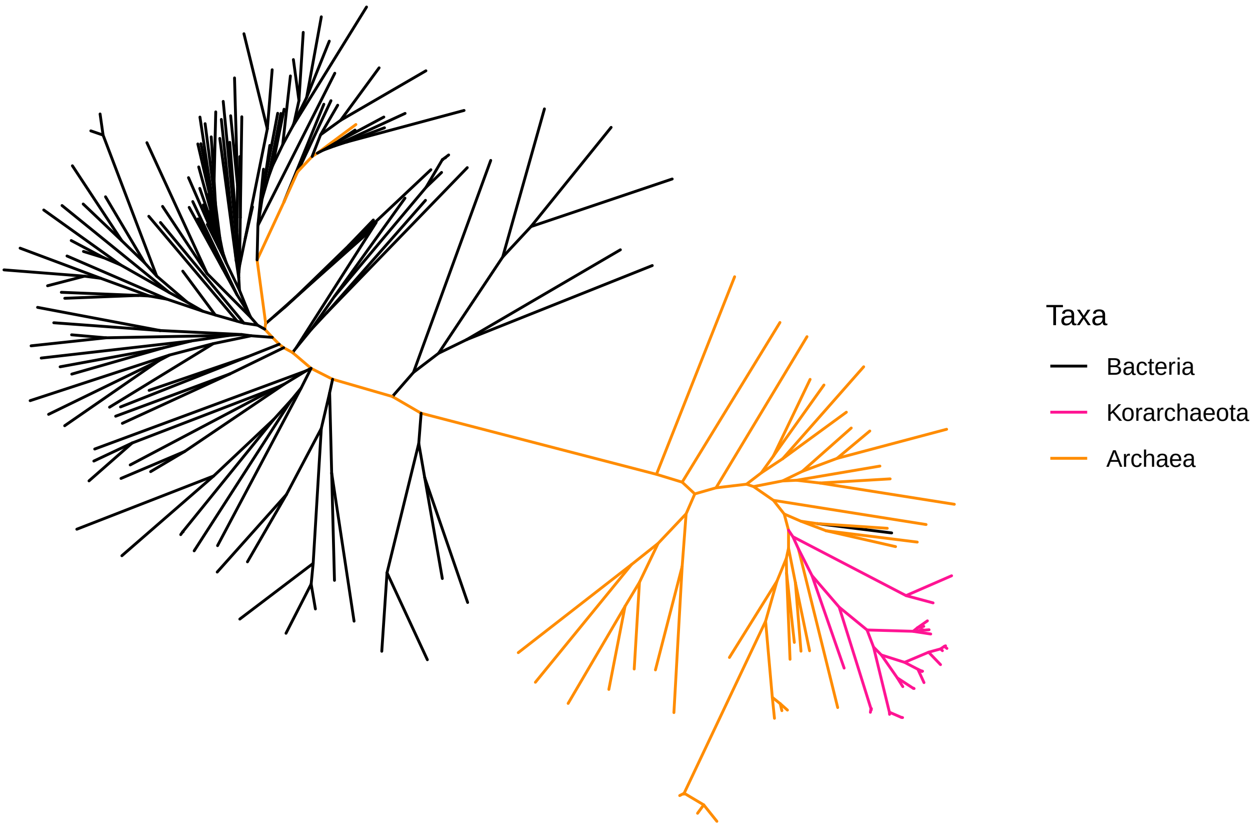

Kor−HOG0000924

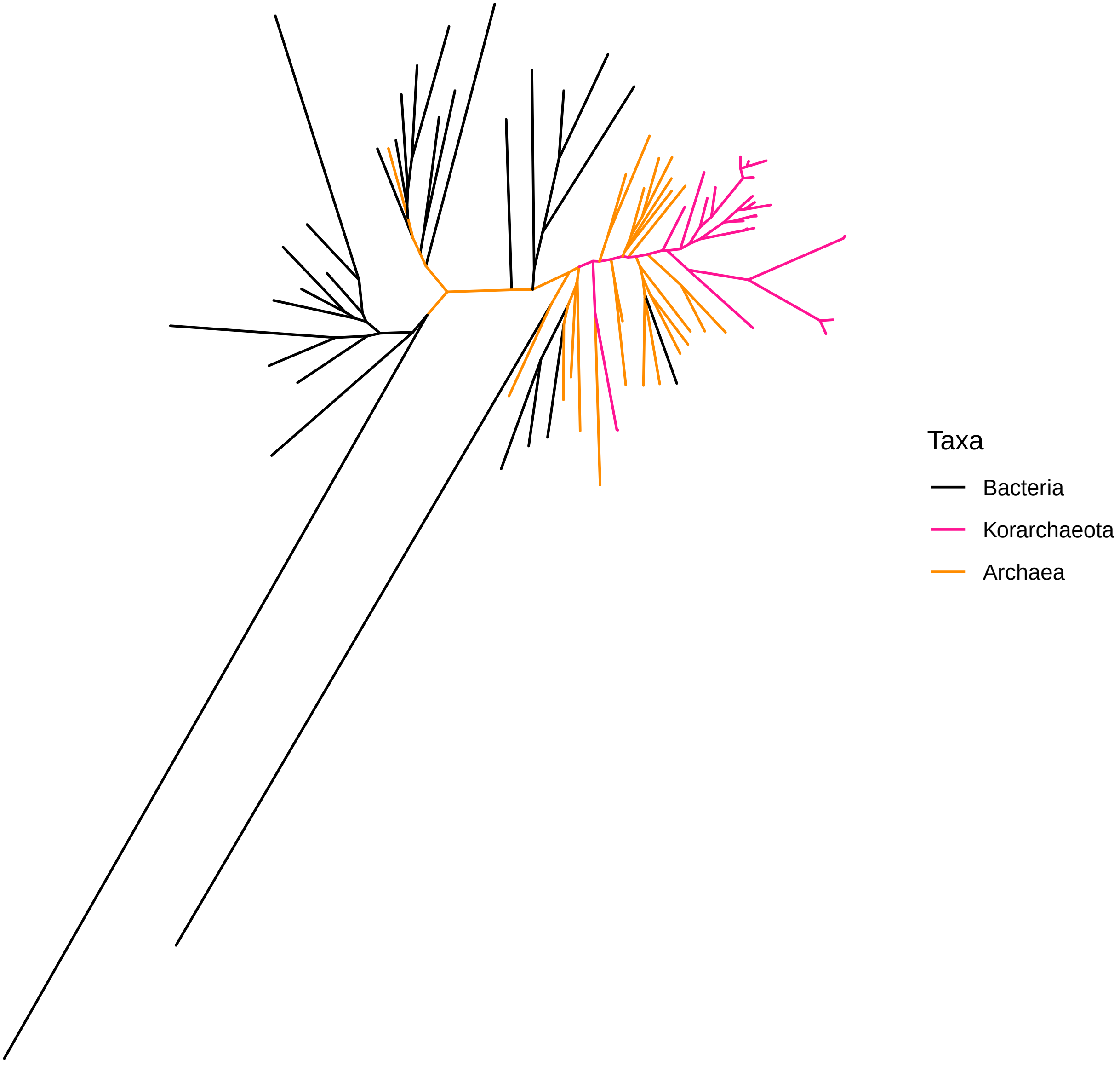

Kor−HOG0000925

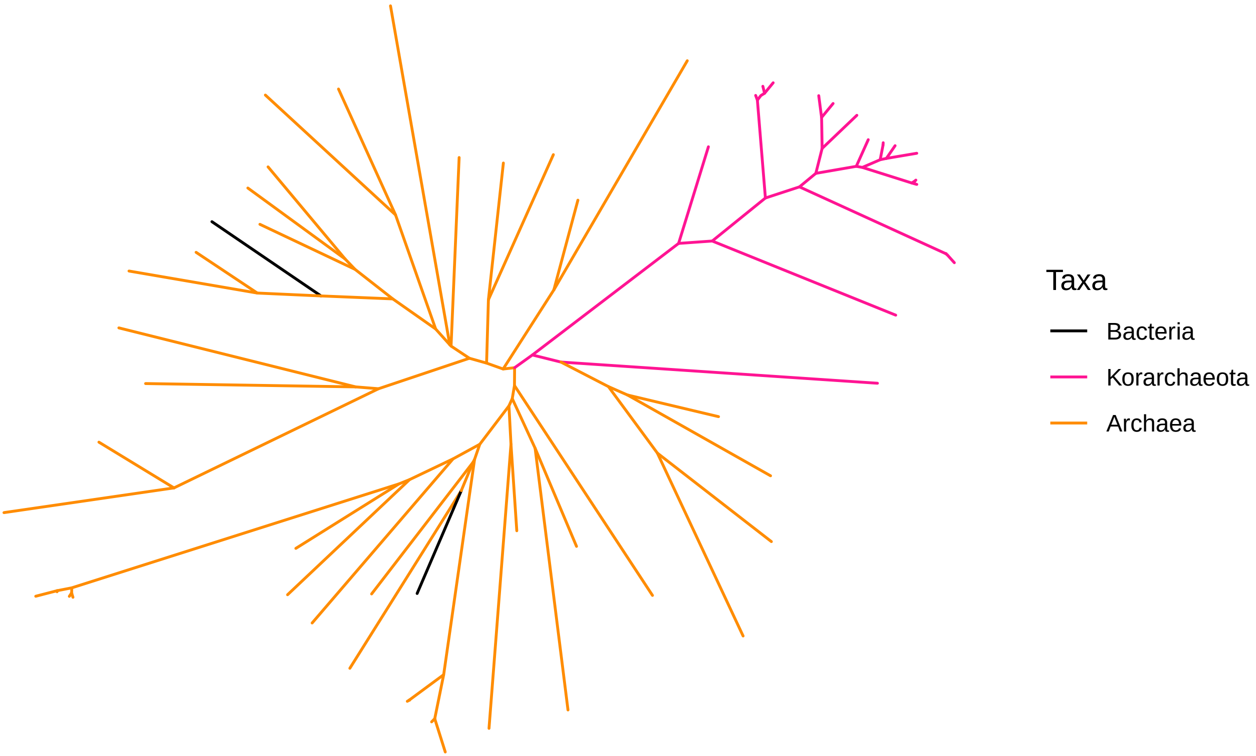

Kor−HOG0000927

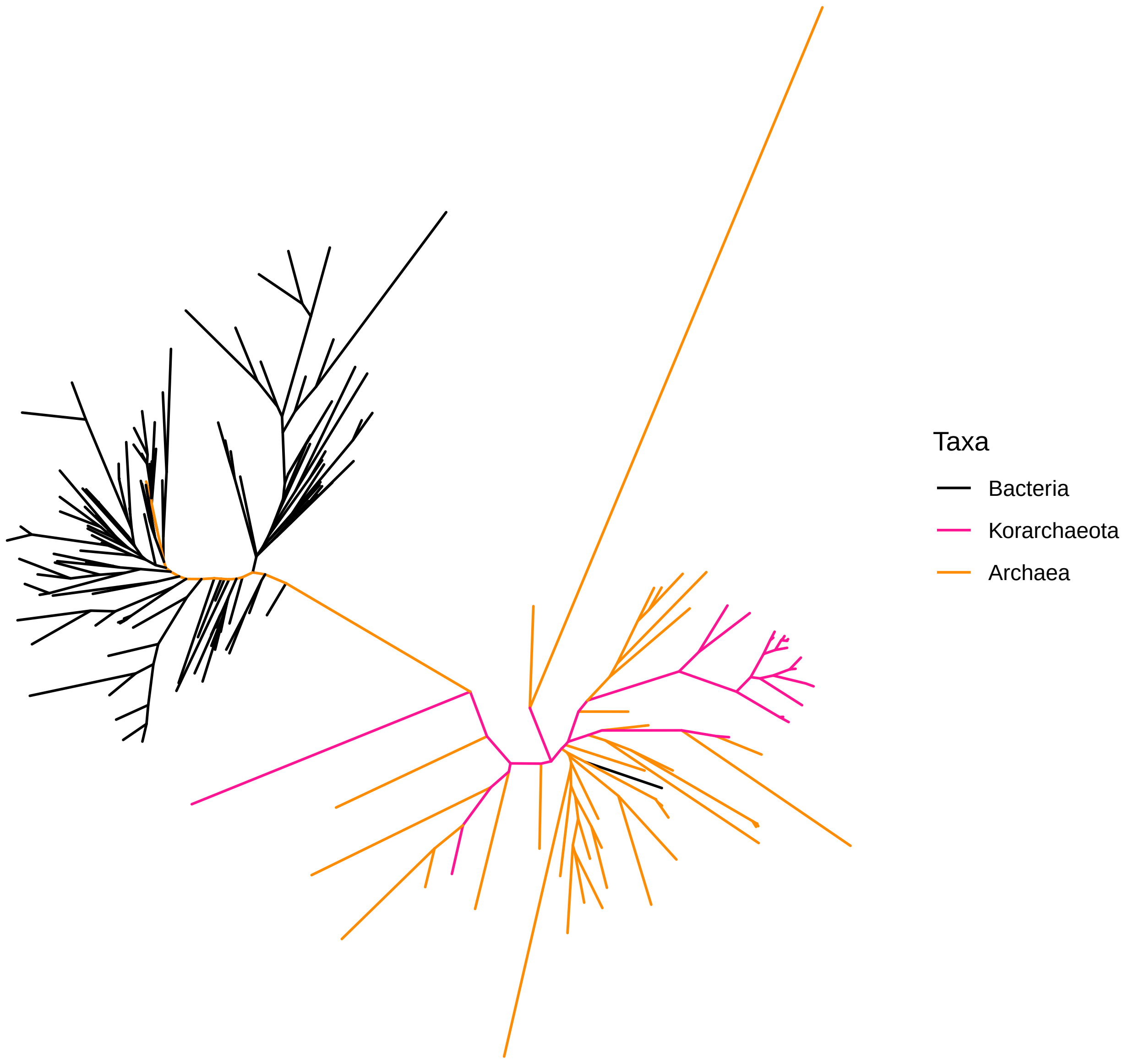

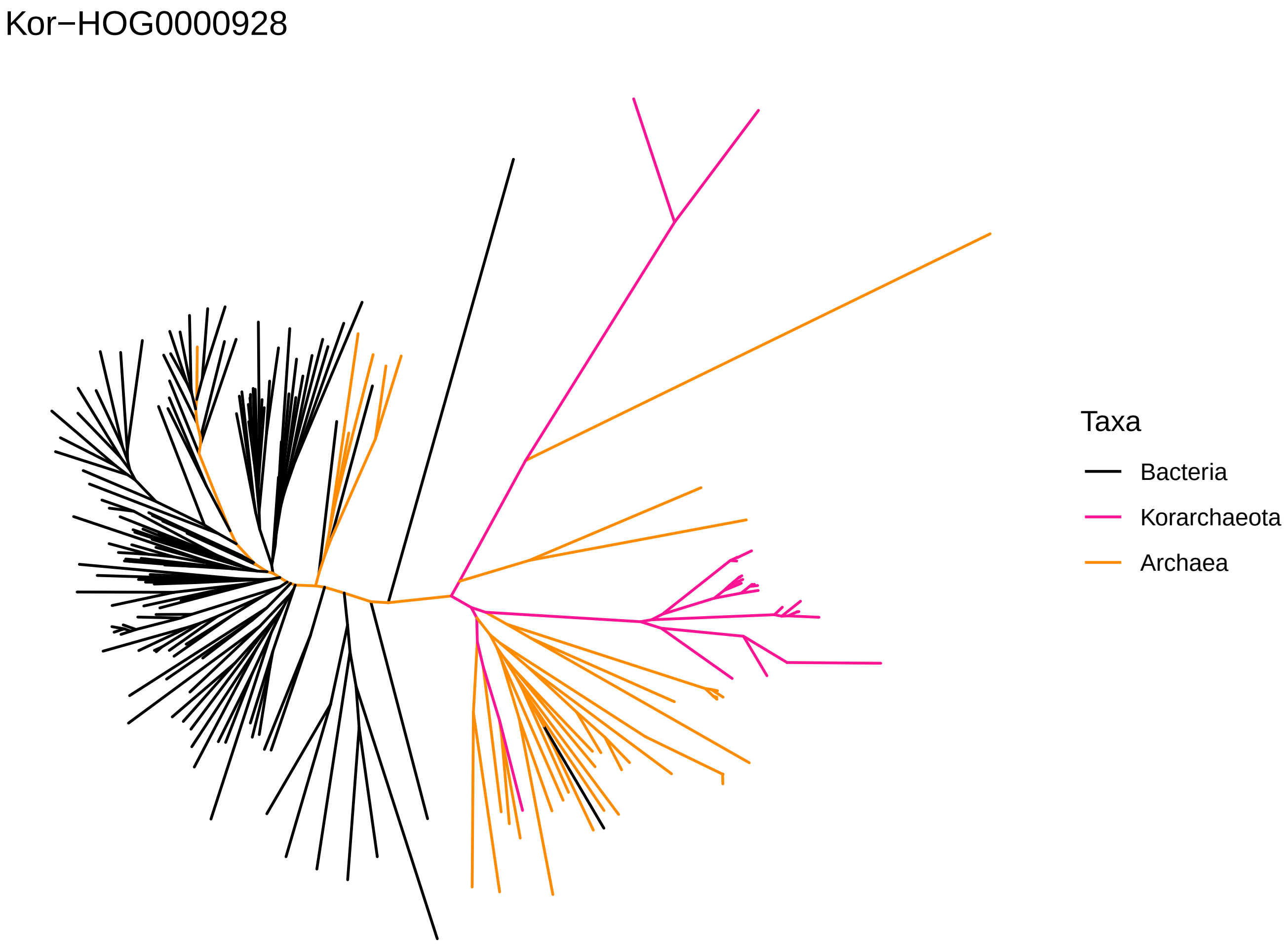
Kor−HOG0000928
Taxa
Bacteria
Korarchaeota
Archaea

Kor−HOG0000929

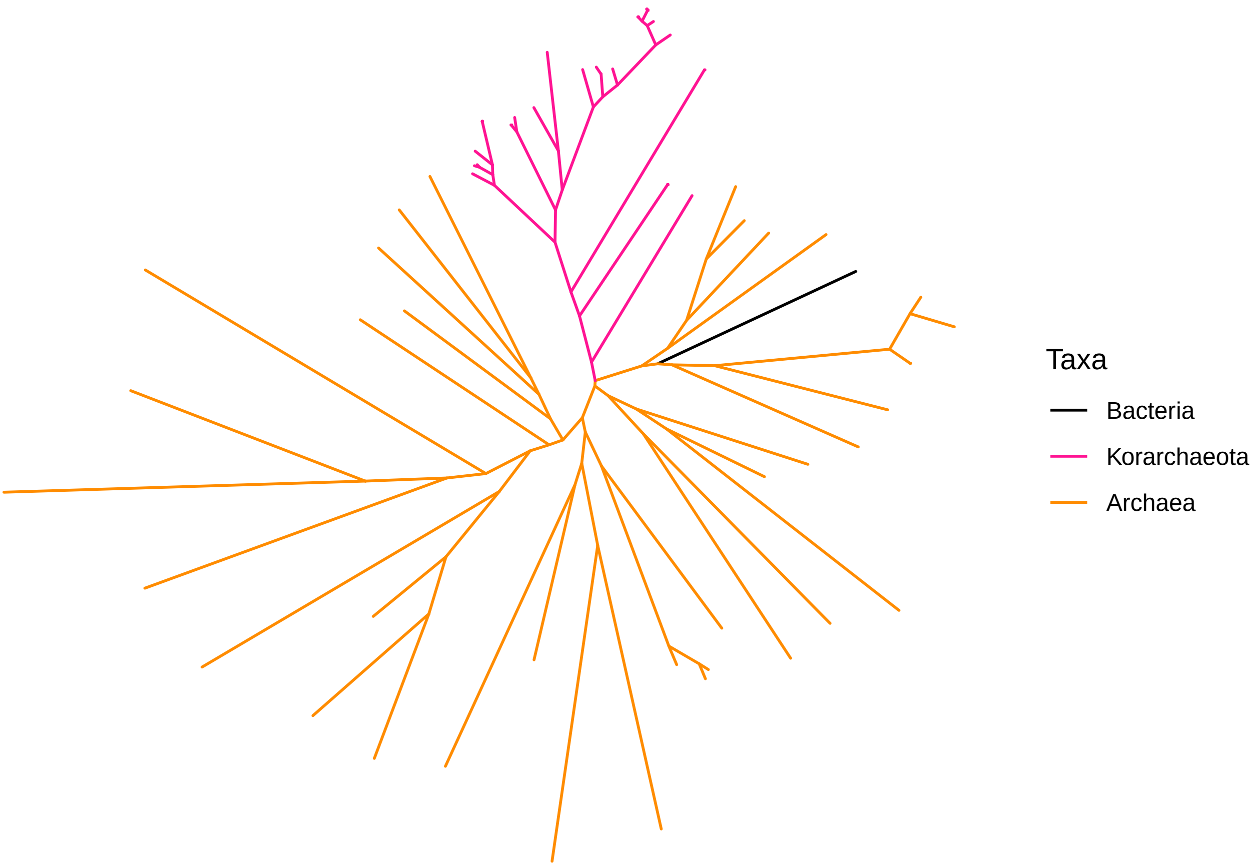

Kor−HOG0000930

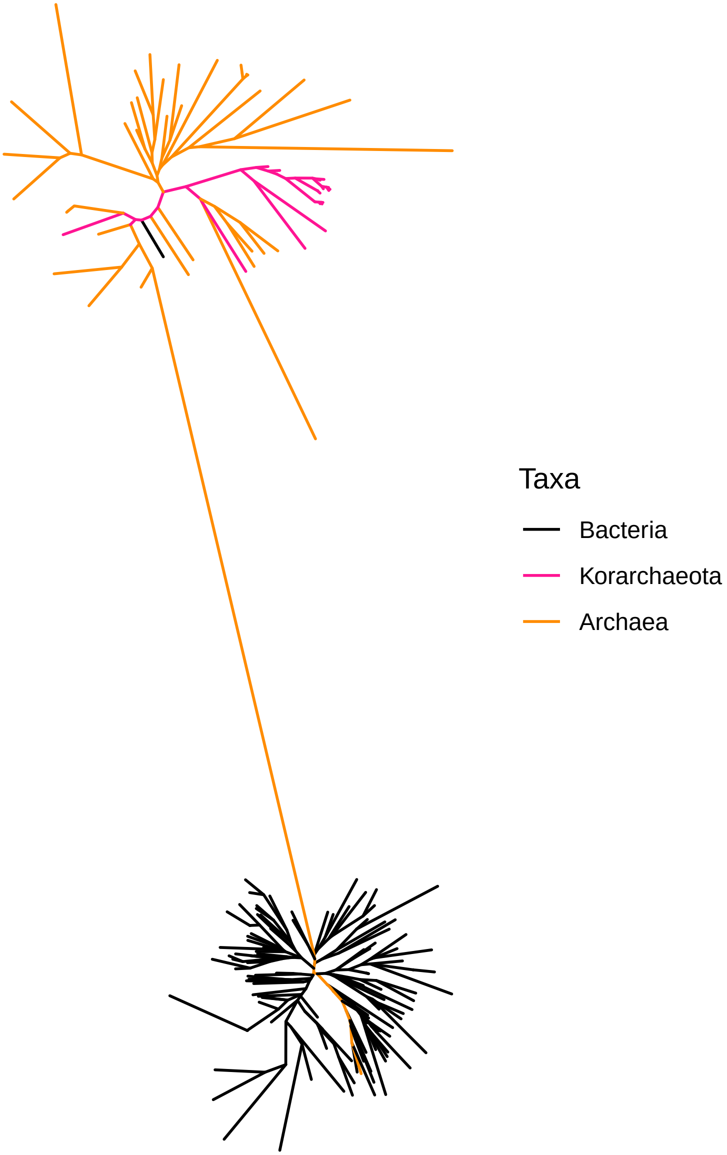

Kor−HOG0000932

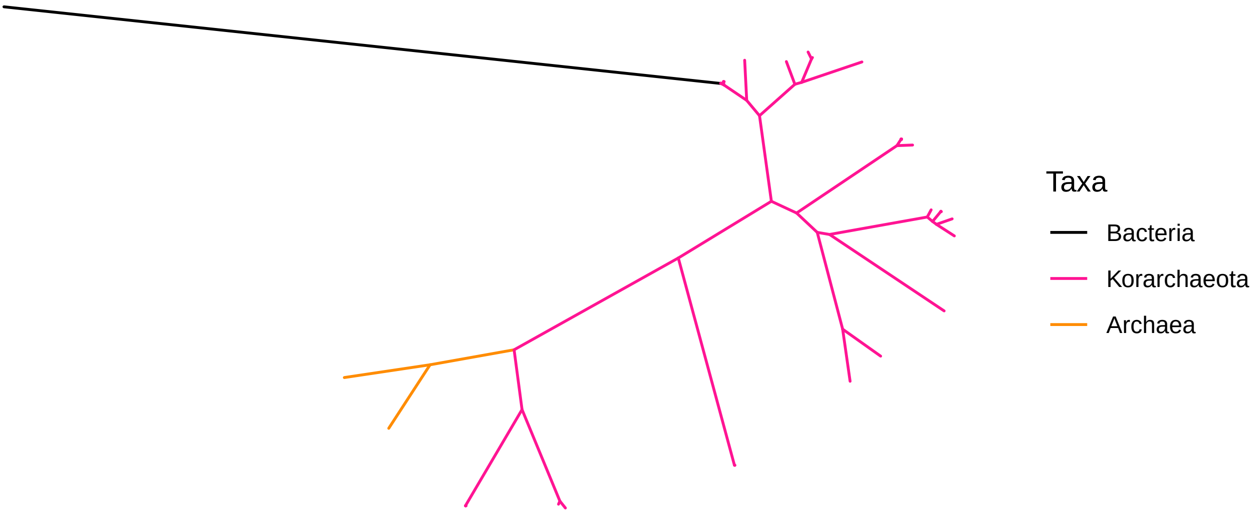

Kor−HOG0000933

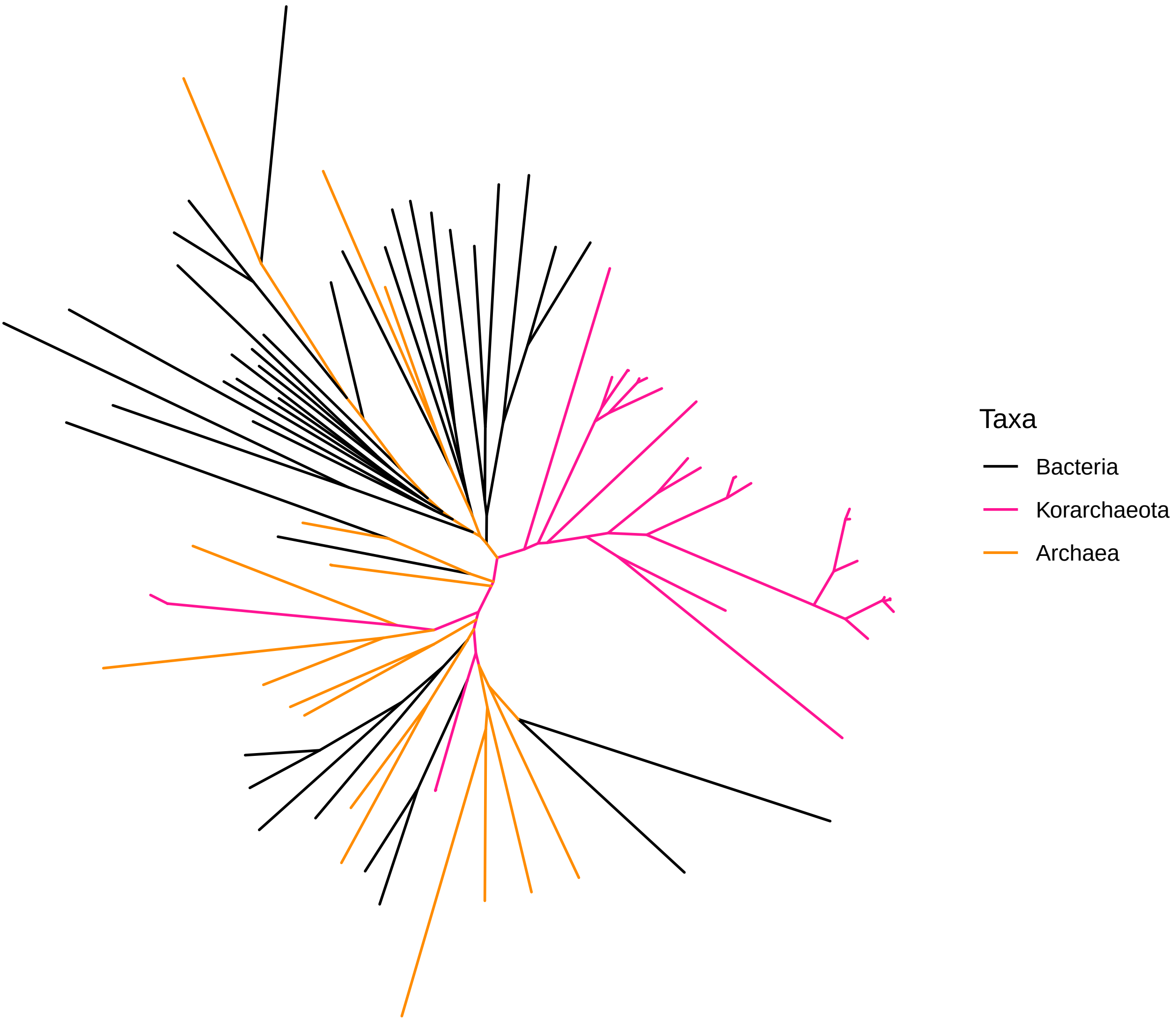

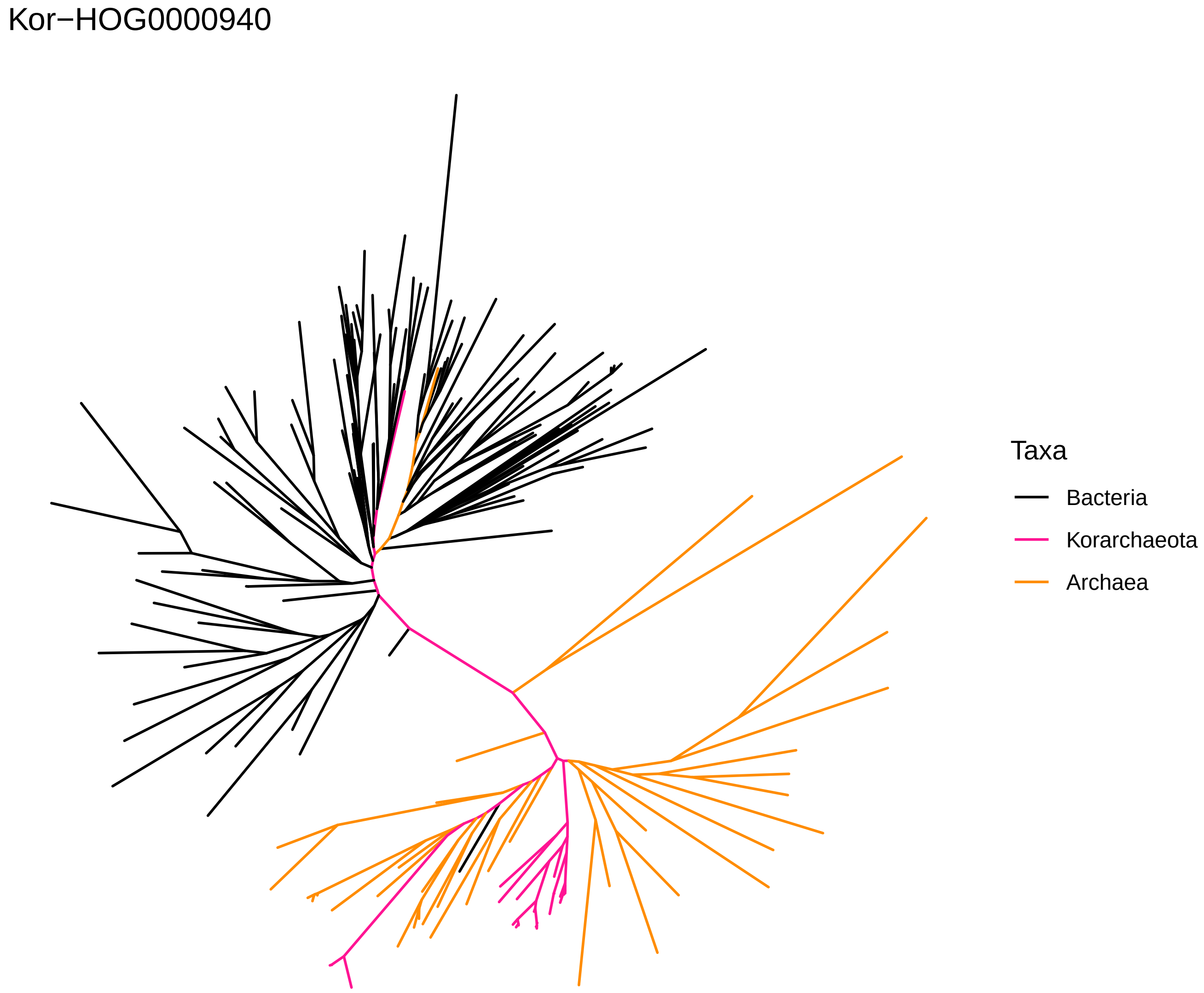

Kor−HOG0000940
Taxa
Bacteria
Korarchaeota
Archaea

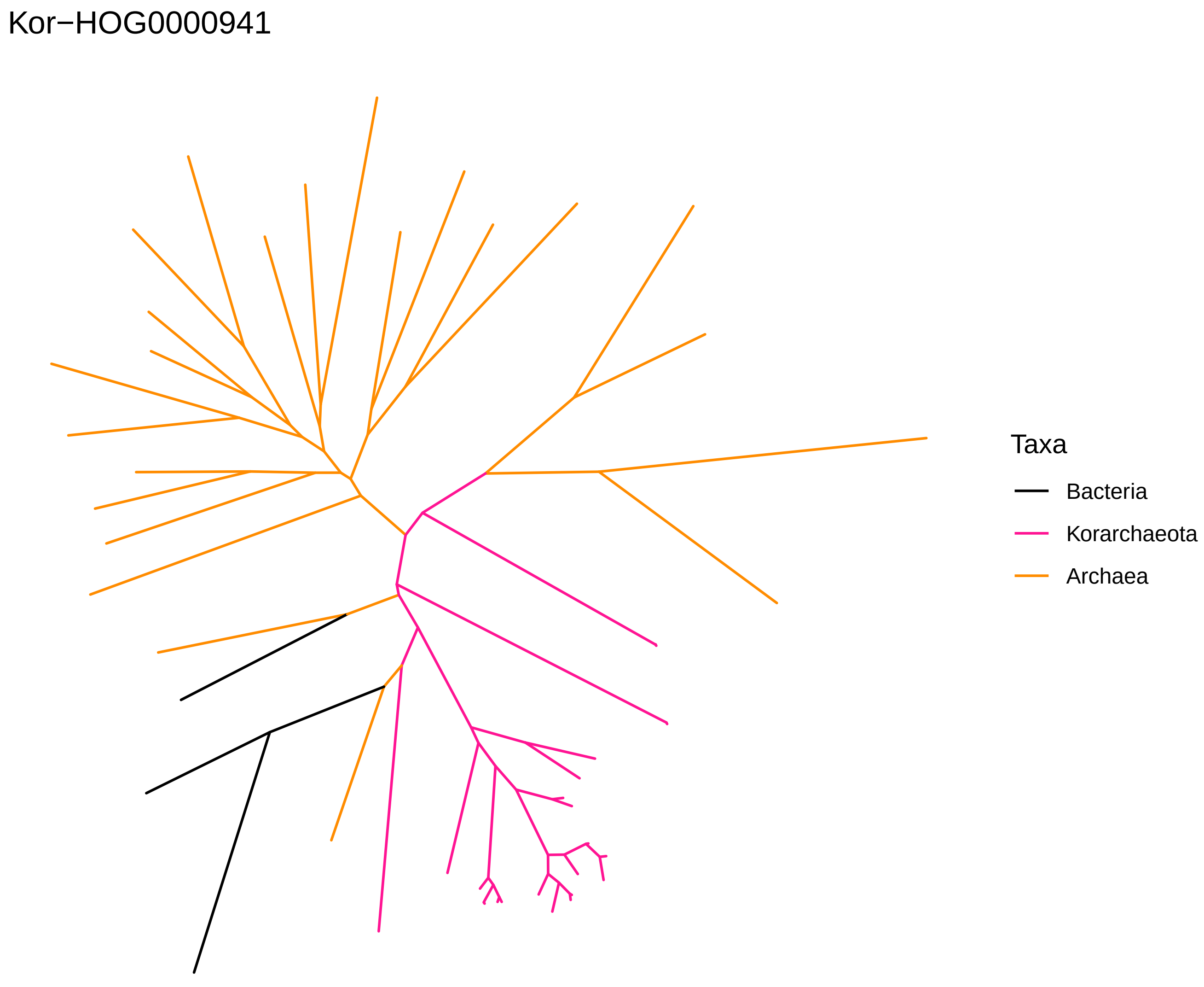
Kor−HOG0000941
Taxa
Bacteria
Korarchaeota
Archaea

Kor−HOG0000956

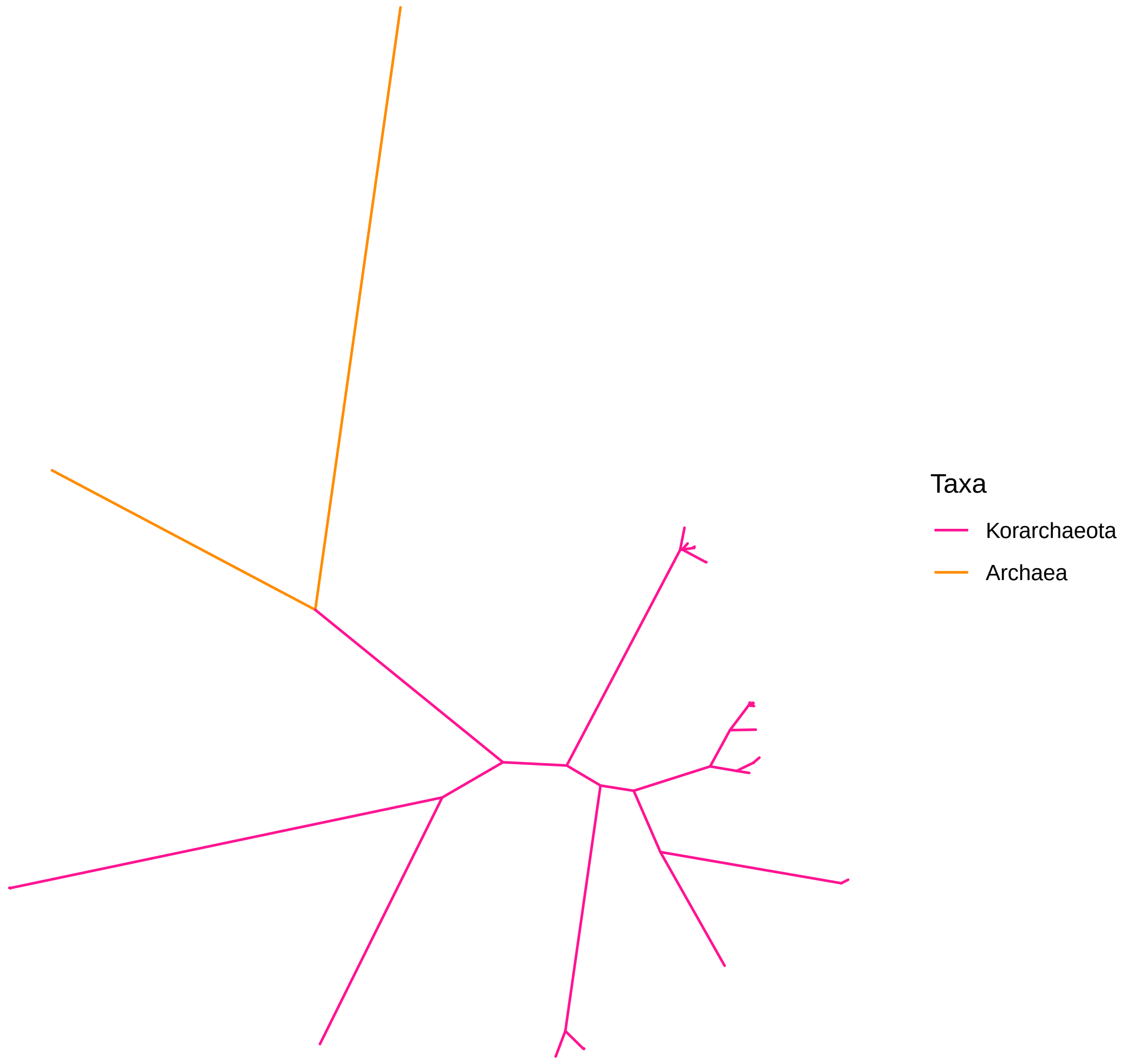

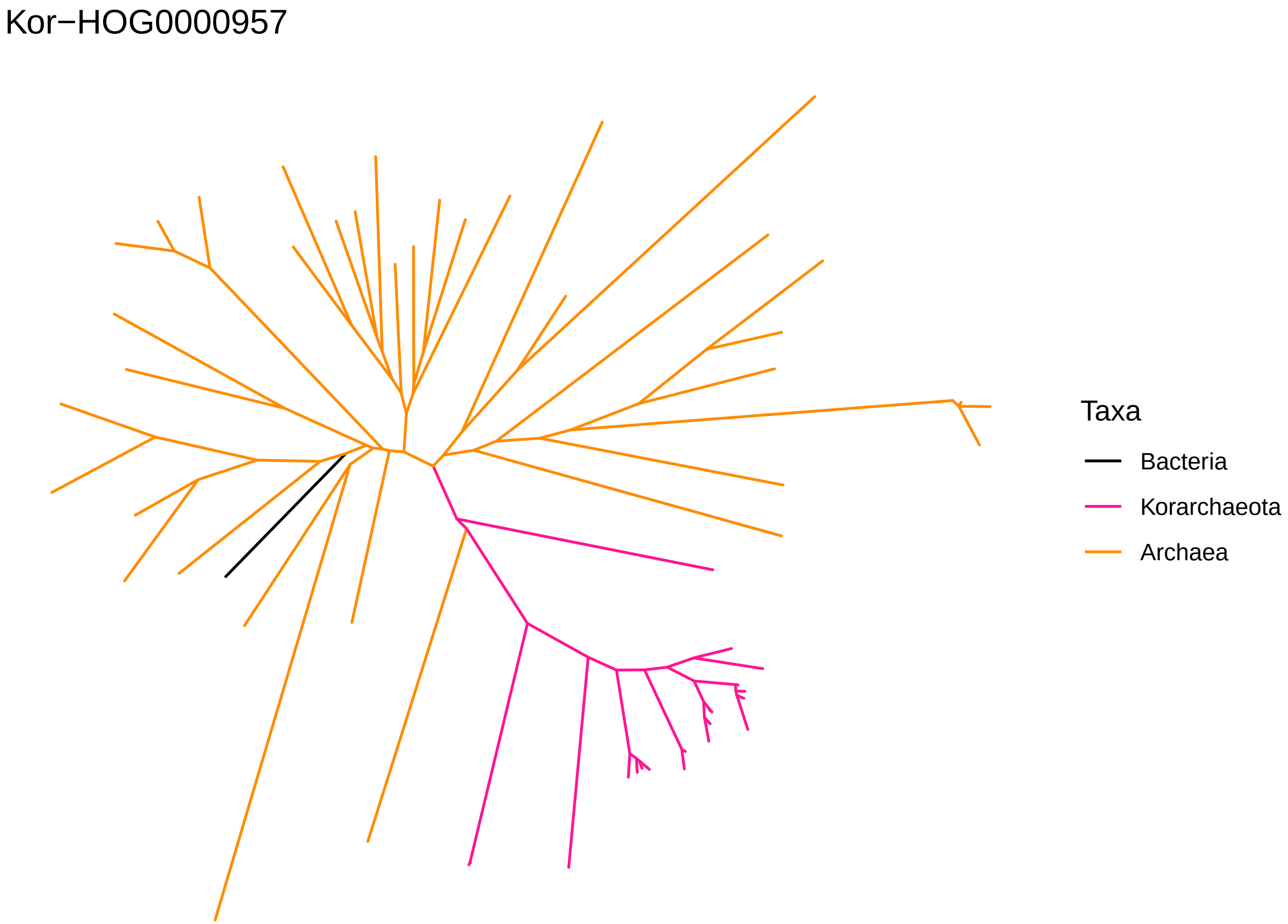
Kor−HOG0000957
Taxa
Bacteria
Korarchaeota
Archaea

Kor−HOG0000958

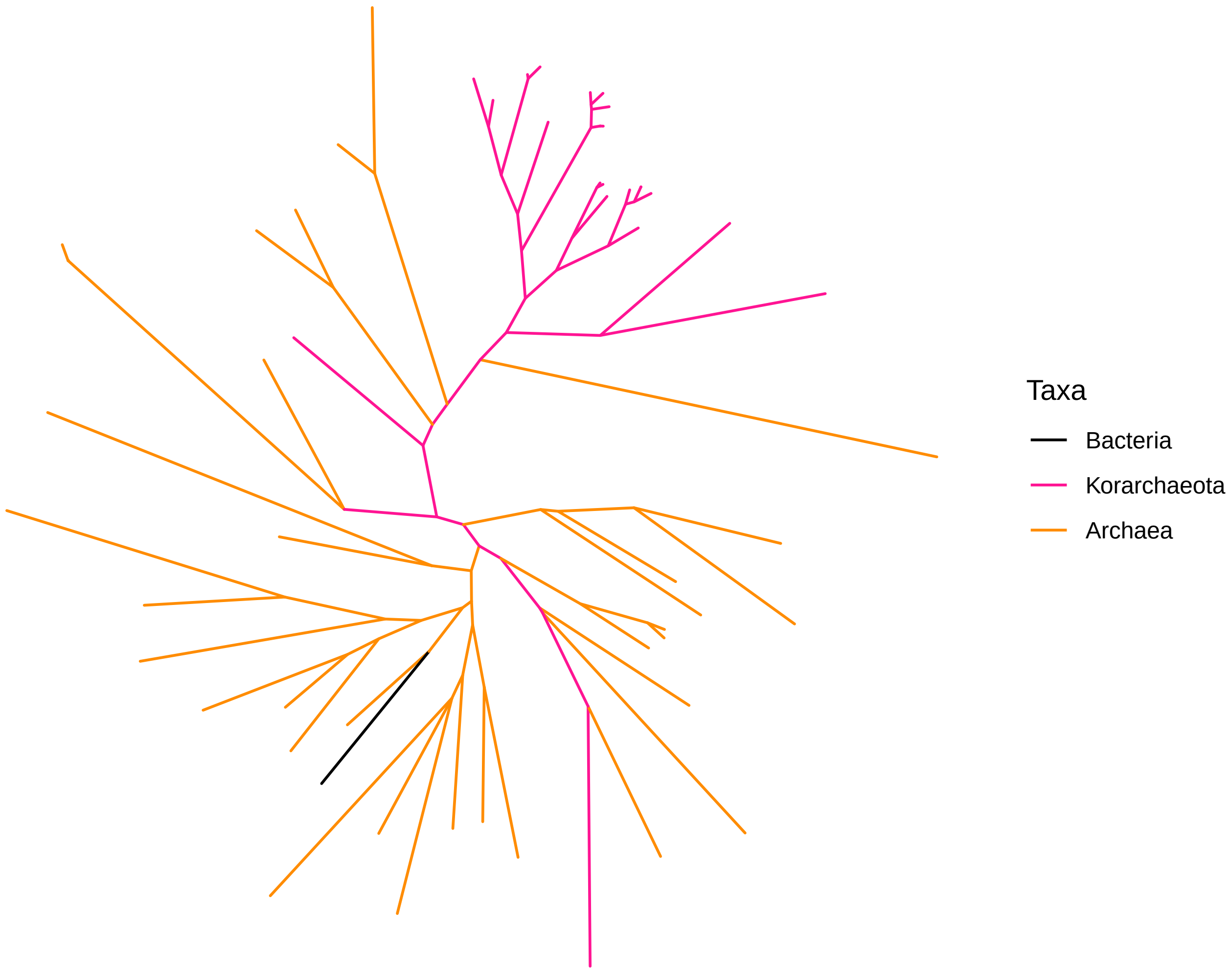

Kor−HOG0000966

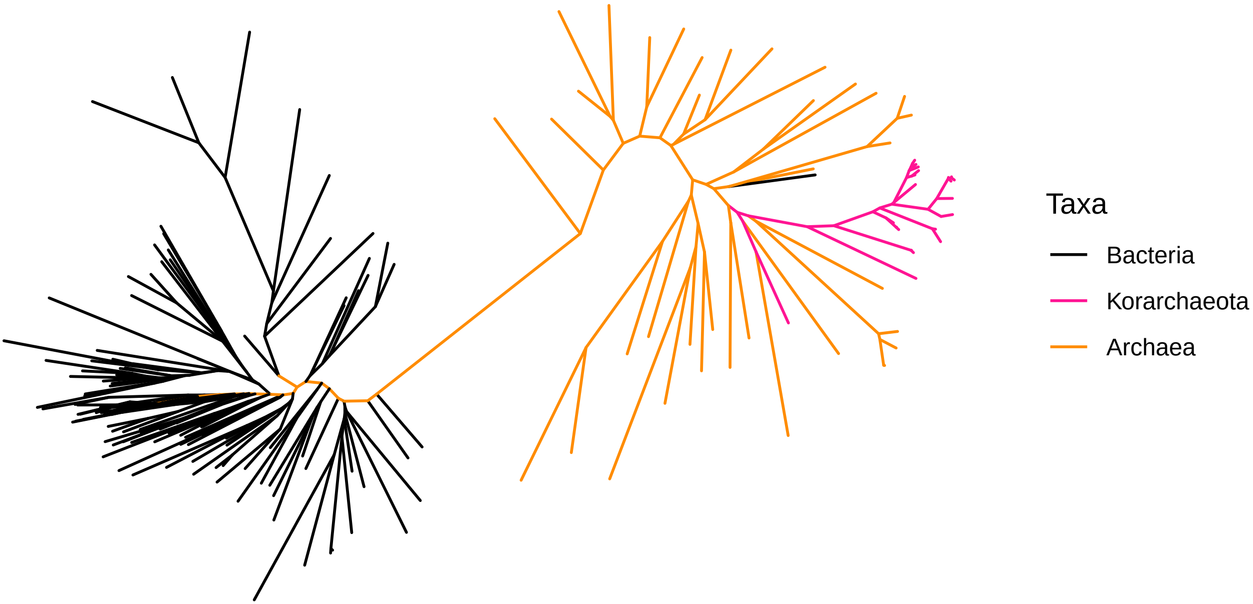

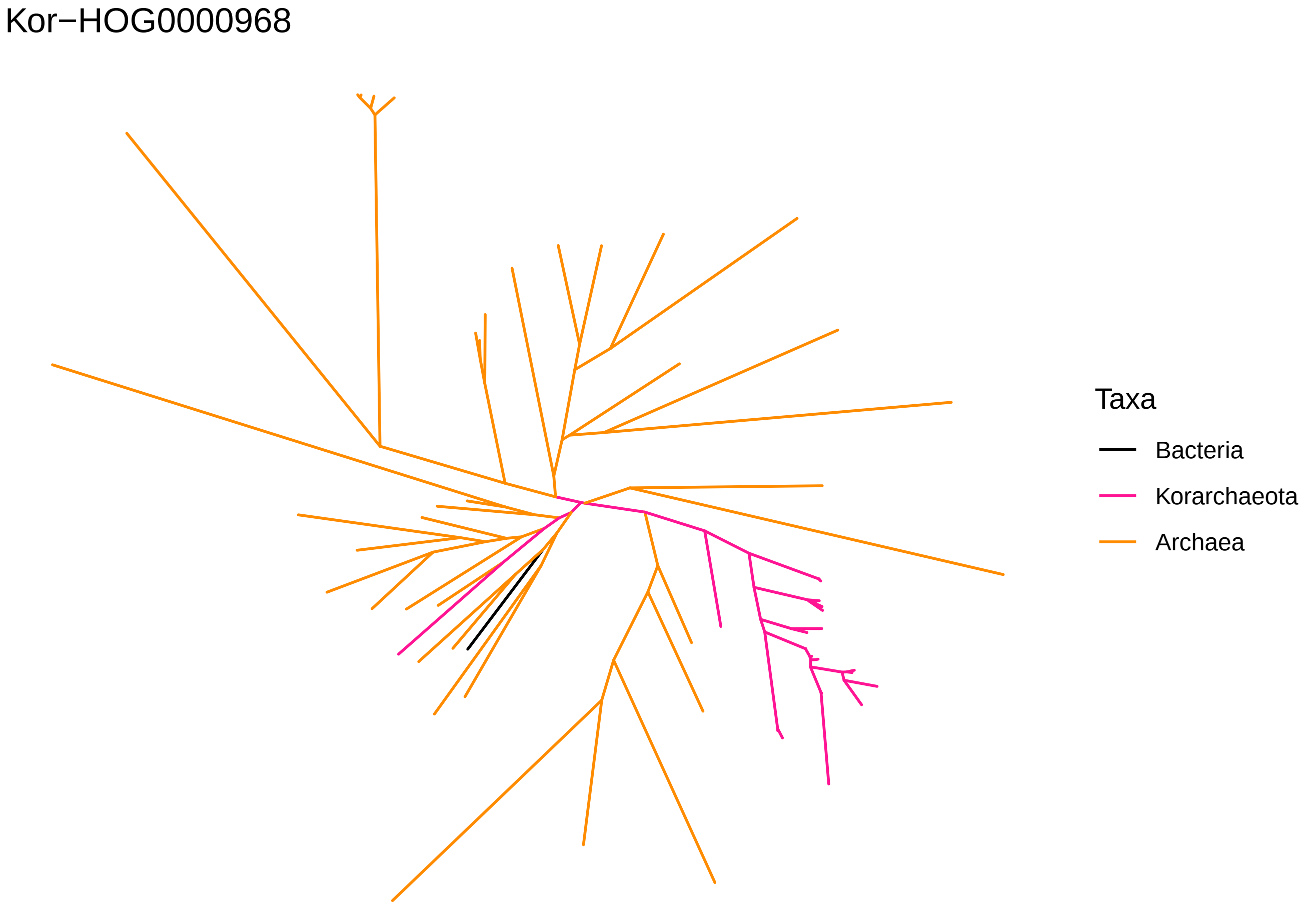
Kor−HOG0000968
Taxa
Bacteria
Korarchaeota
Archaea

Kor−HOG0000969

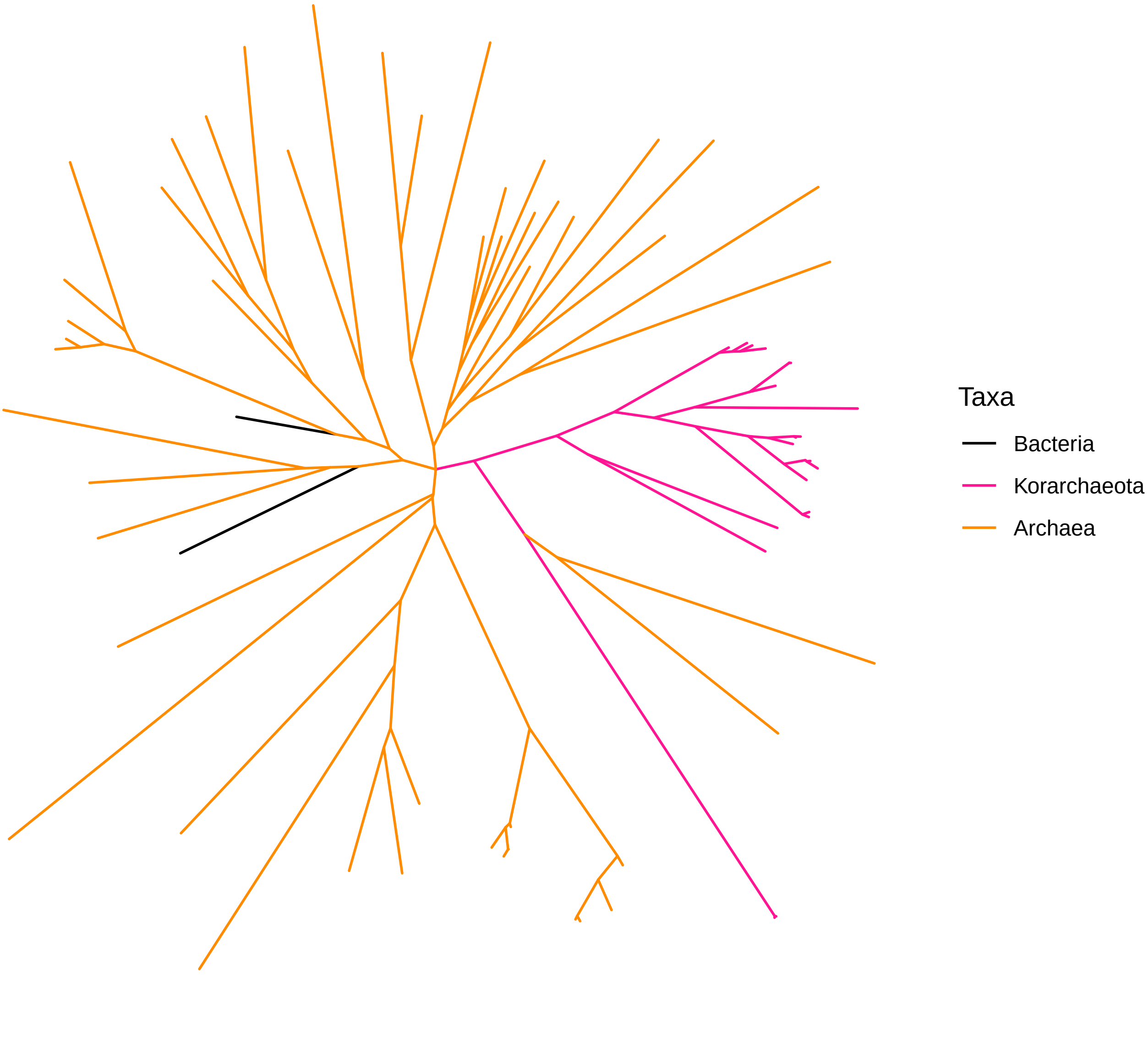

Kor−HOG0000970

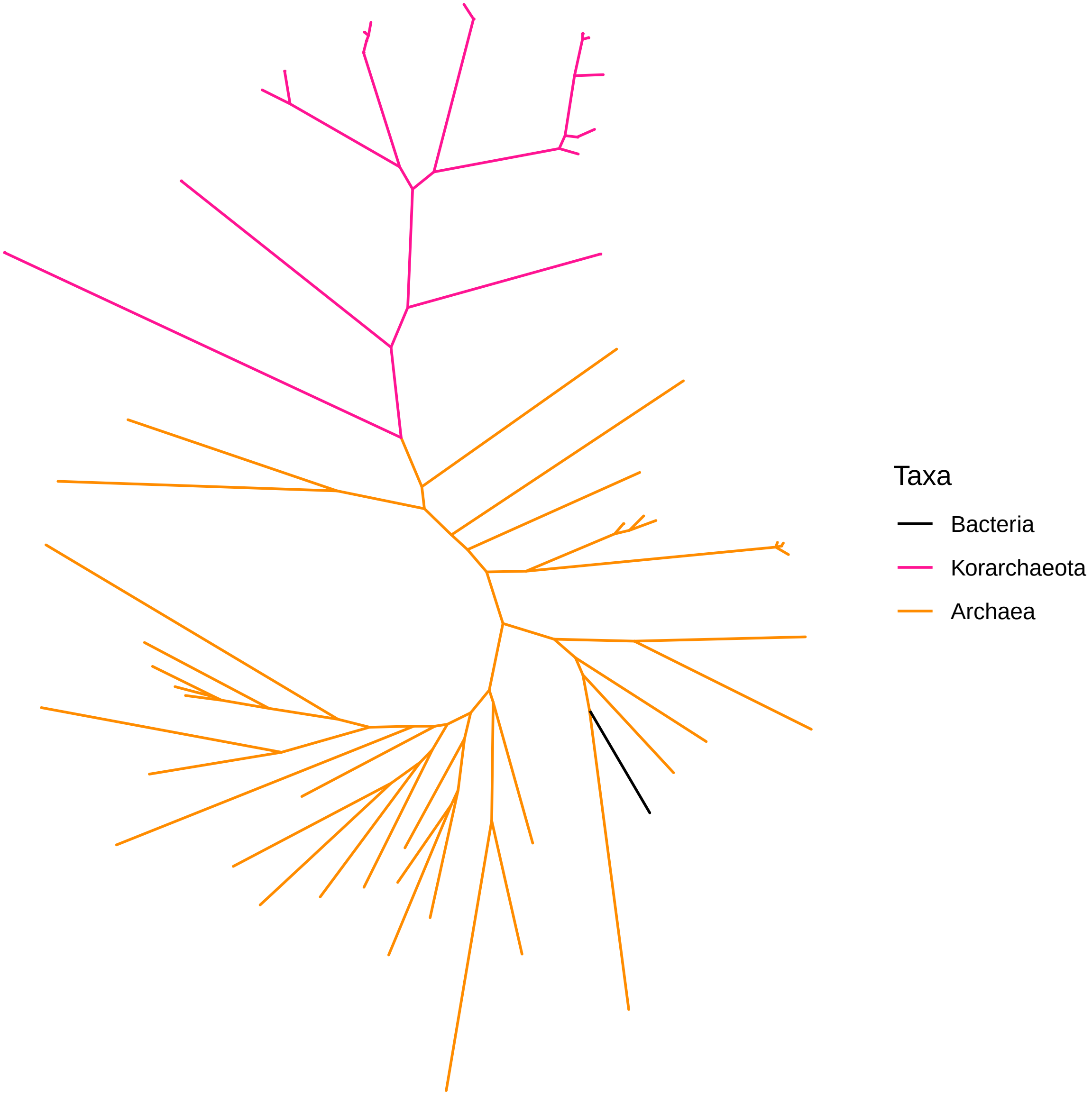

Kor−HOG0000973

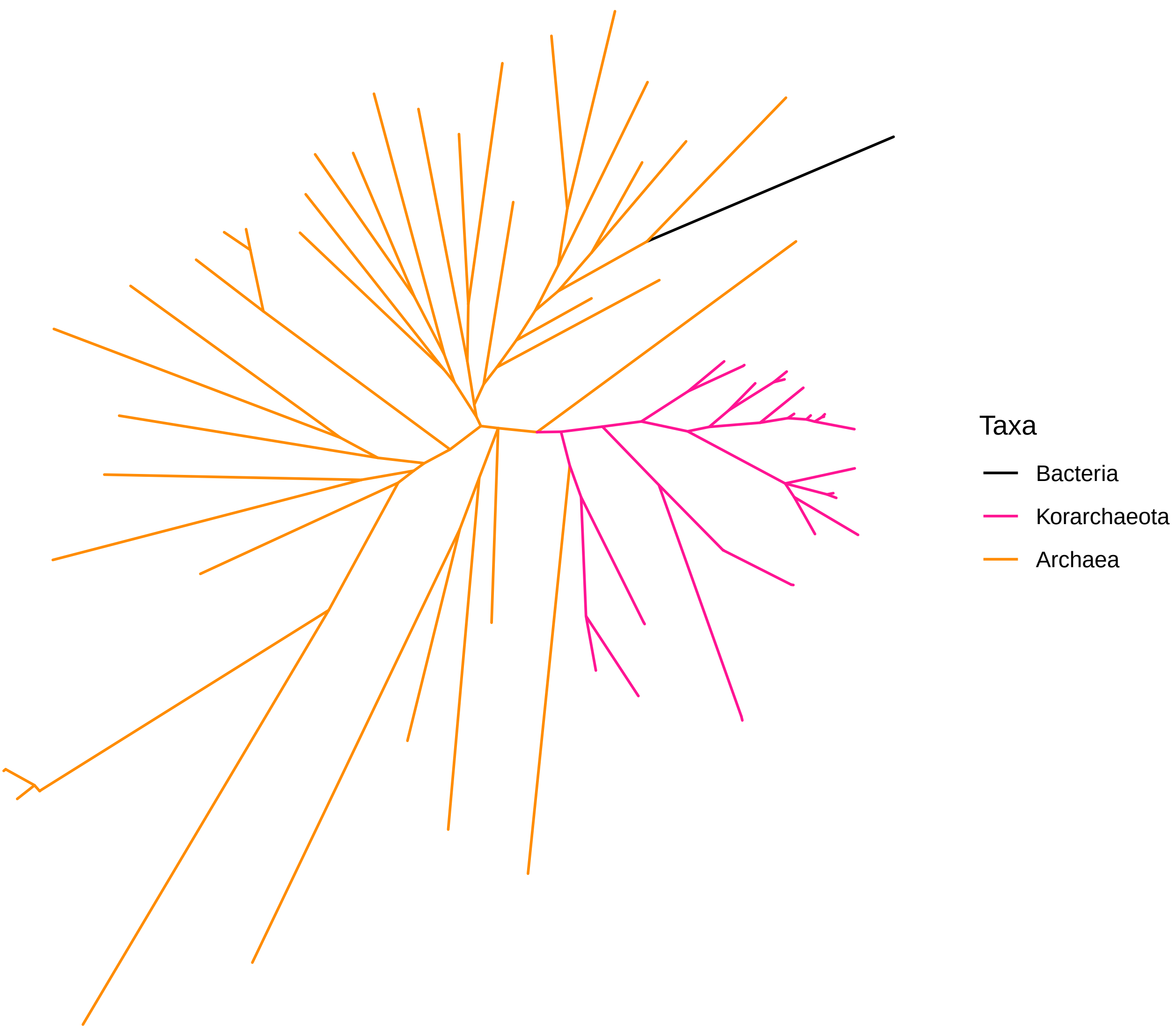

Kor−HOG0000974

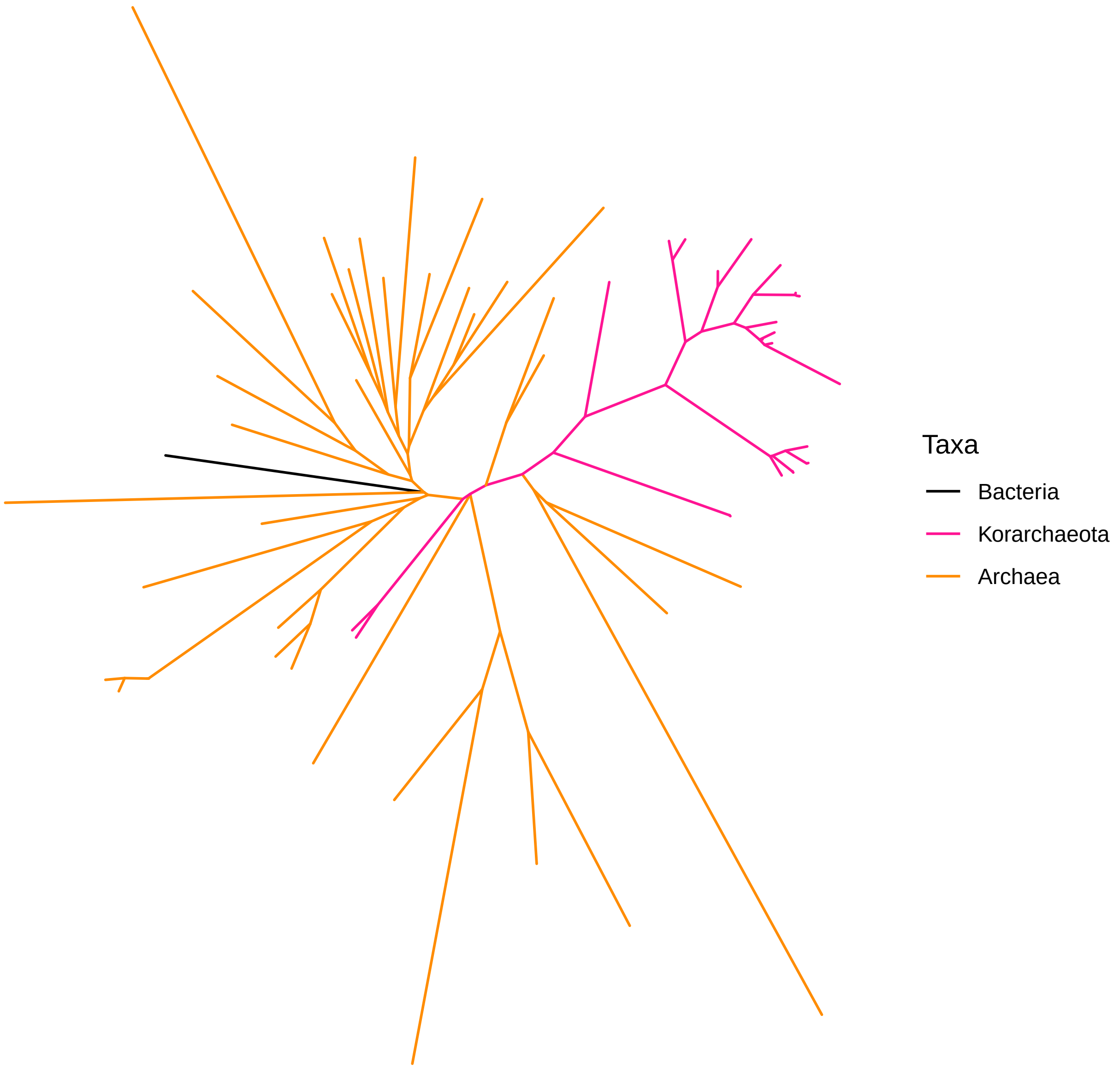

Kor−HOG0000987

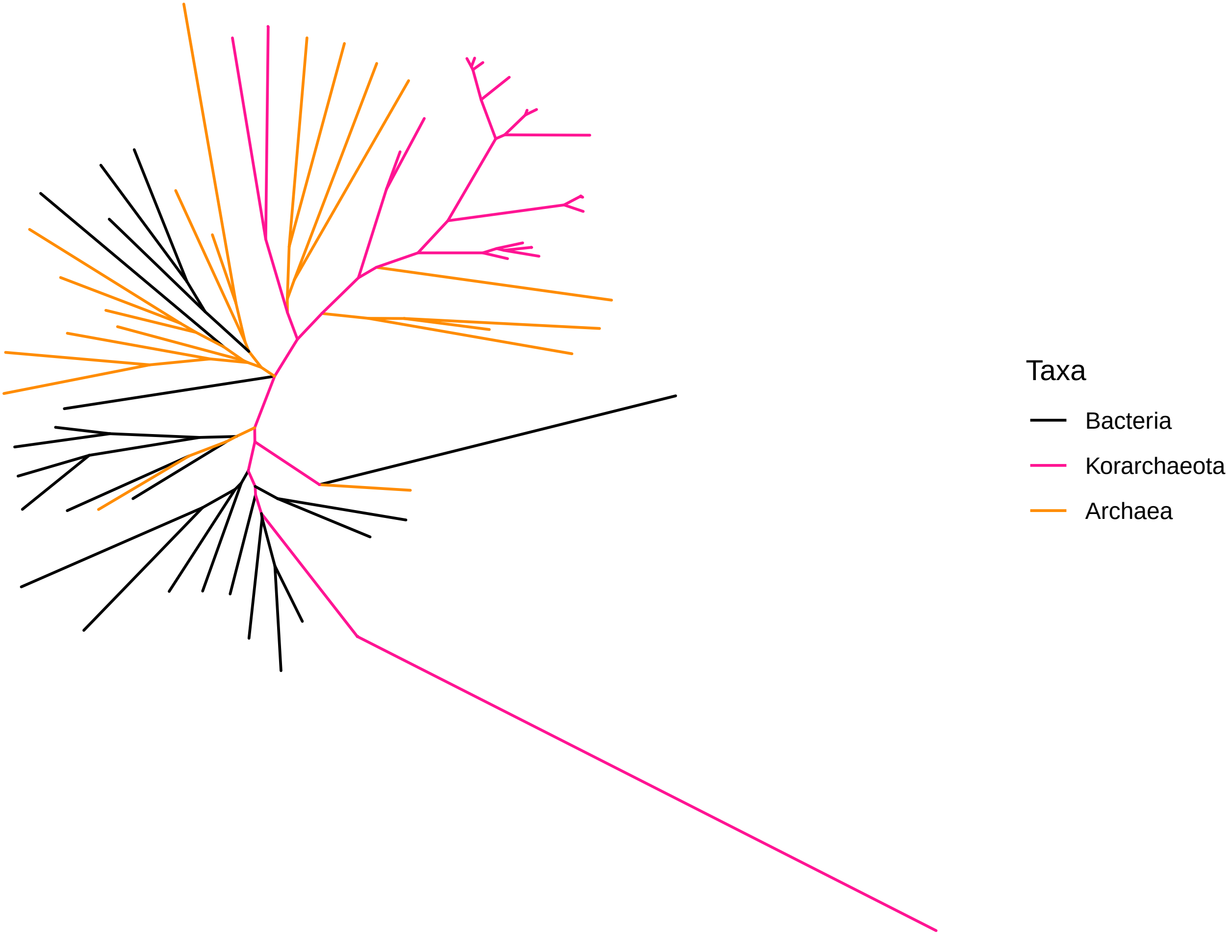

Kor−HOG0001003

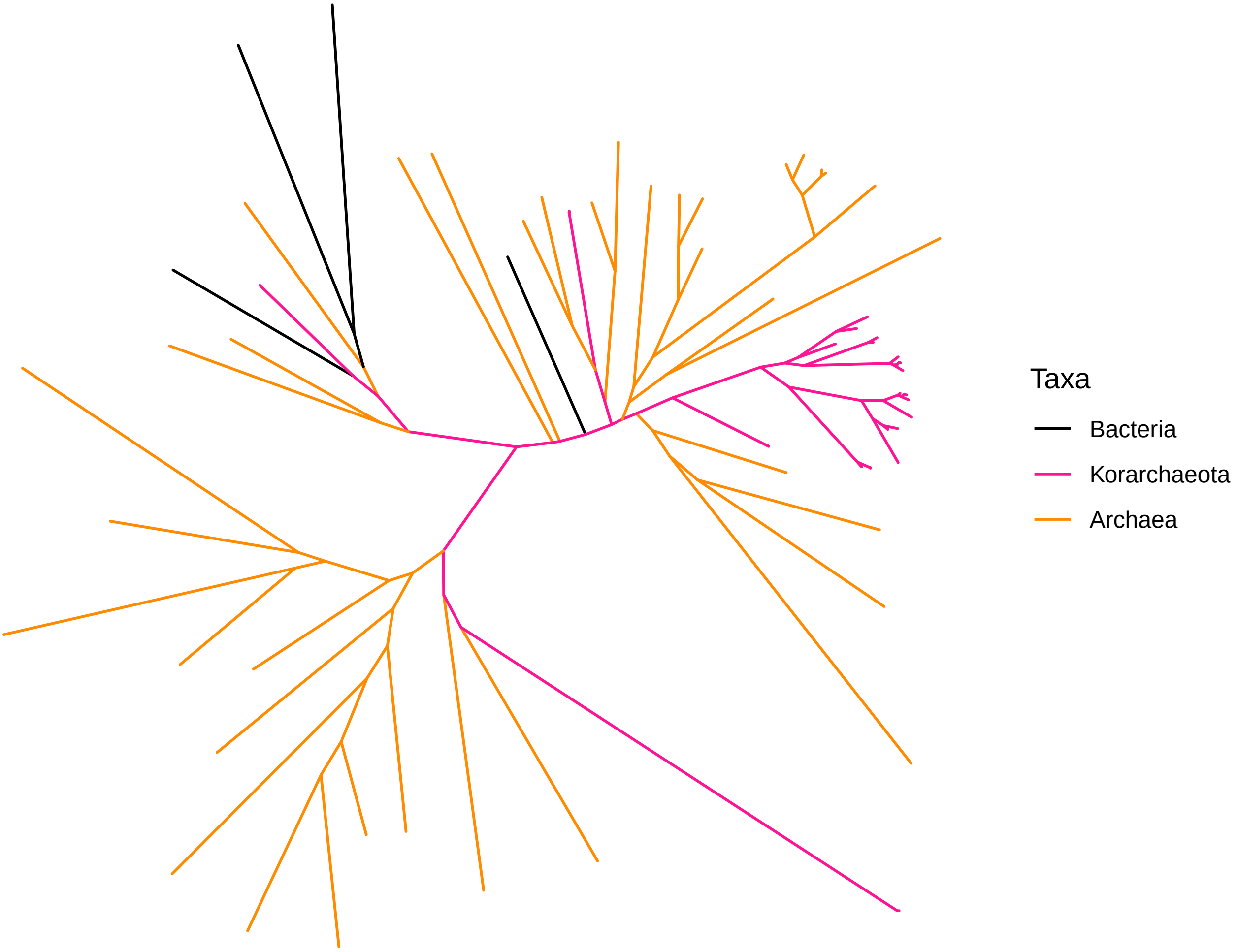

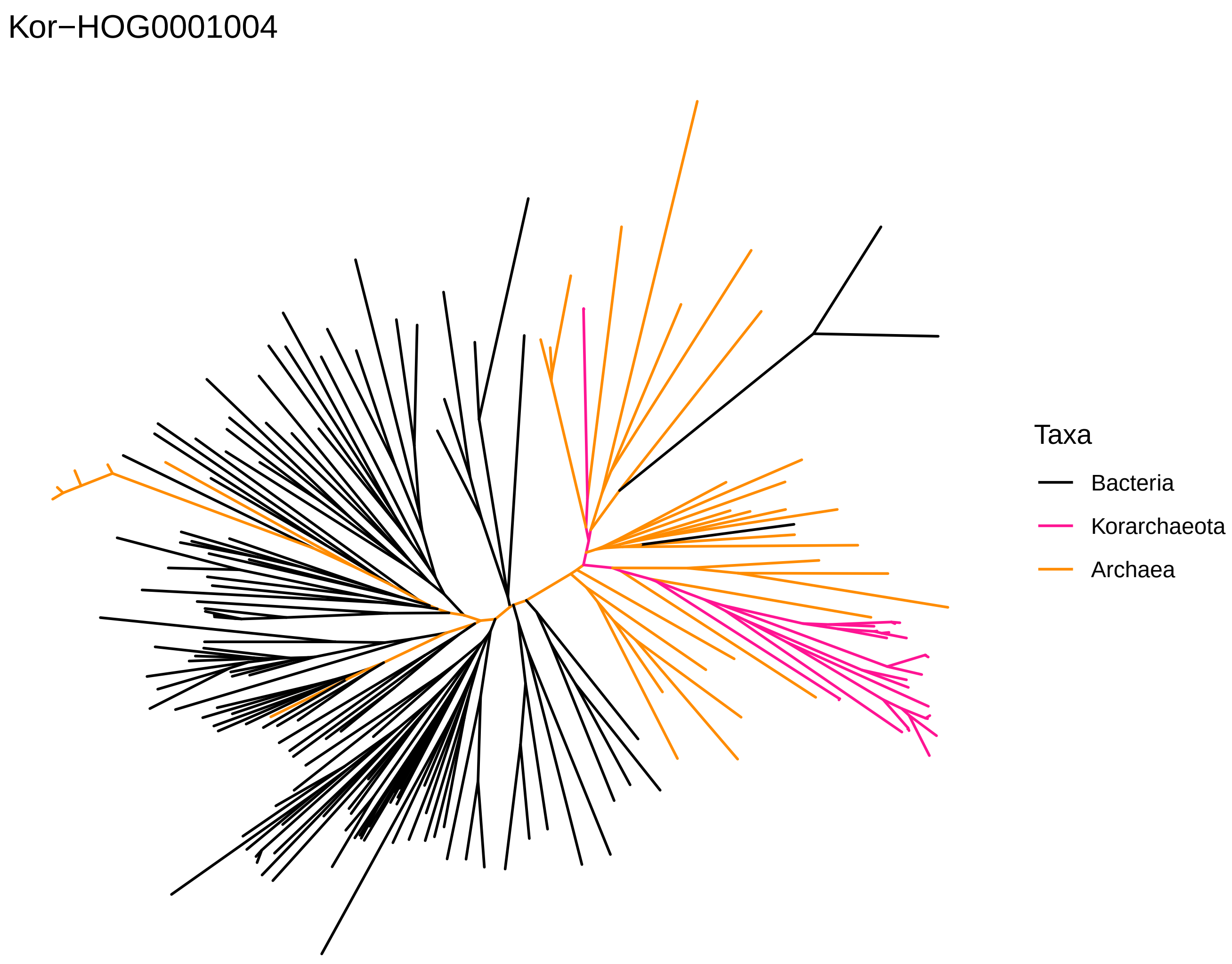
Kor−HOG0001004
Taxa
Bacteria
Korarchaeota
Archaea

Kor−HOG0001005

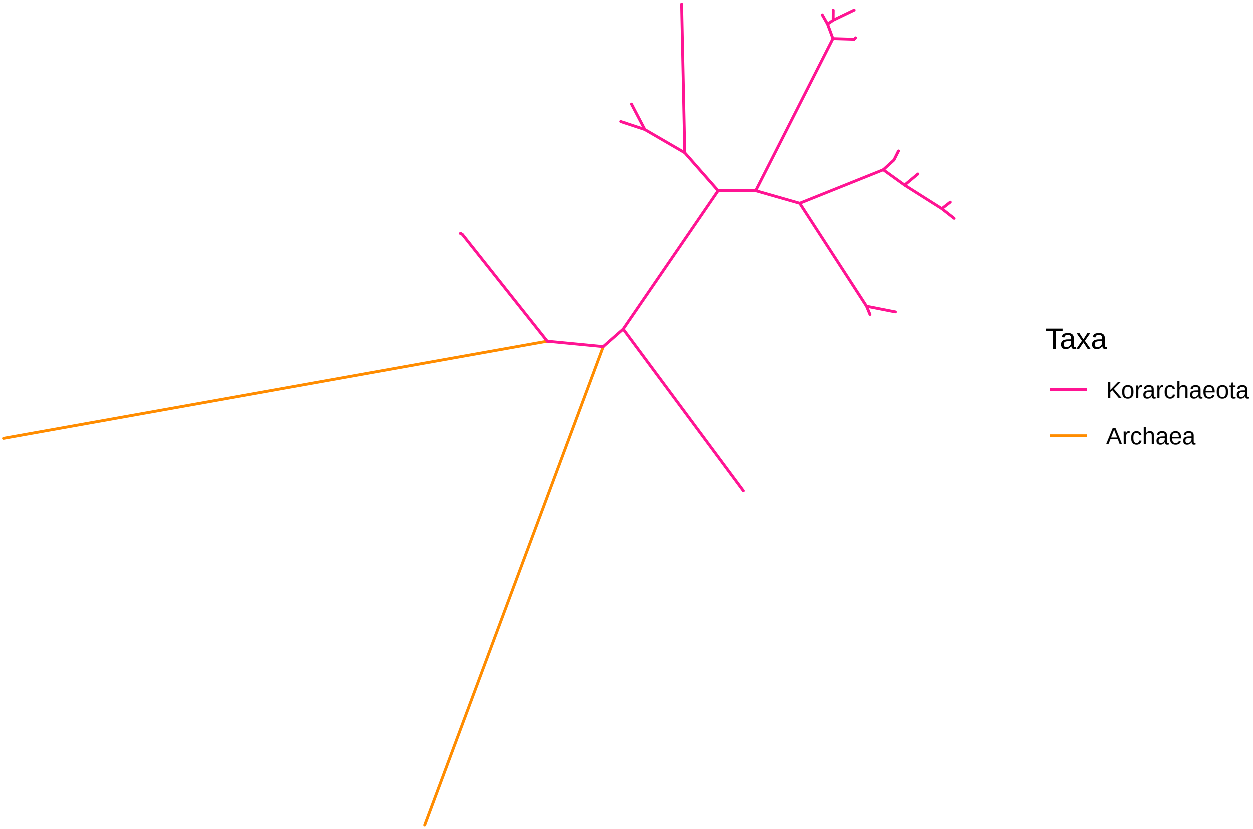

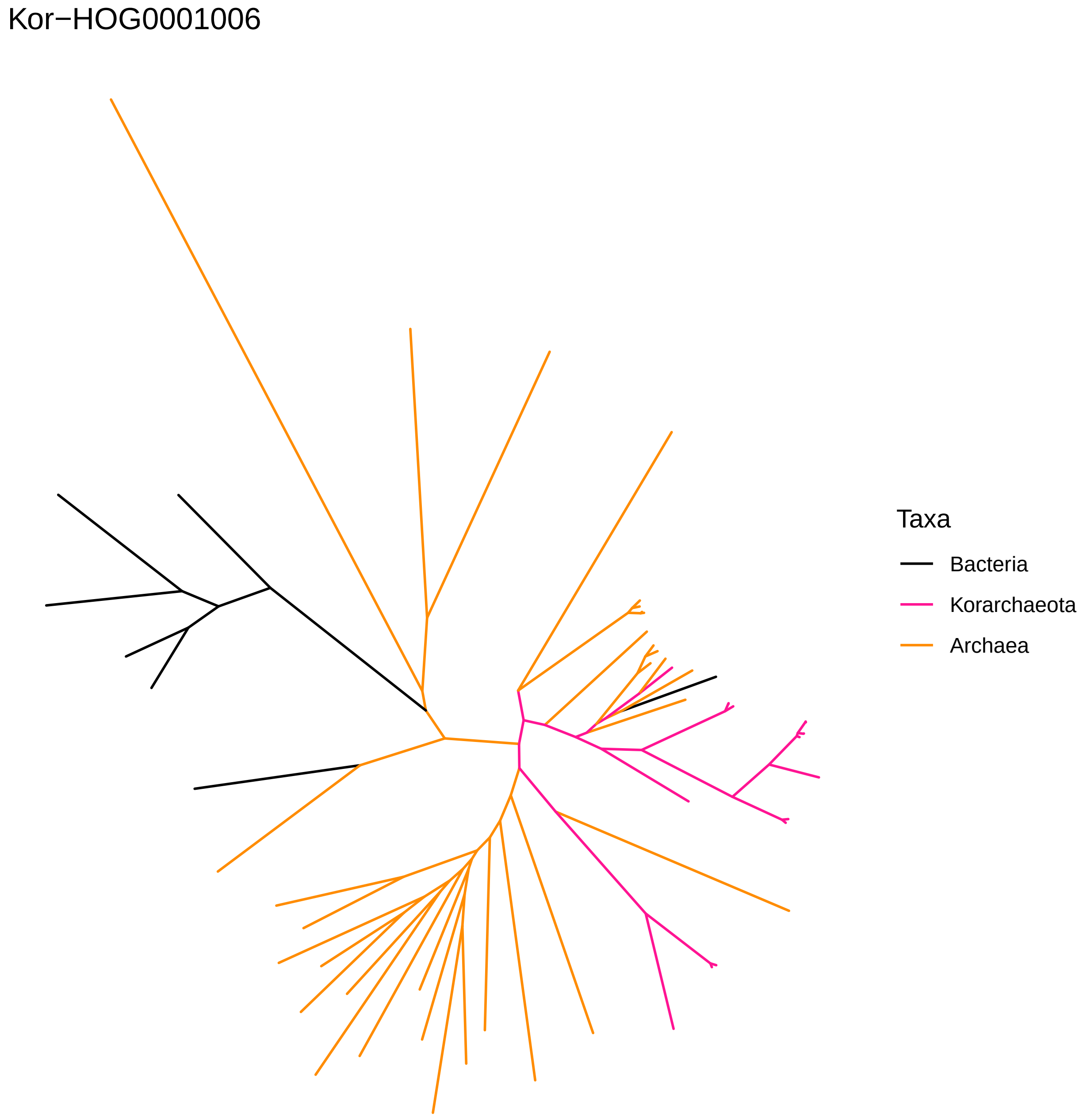

Kor−HOG0001006
Taxa
Bacteria
Korarchaeota
Archaea

Kor−HOG0001009

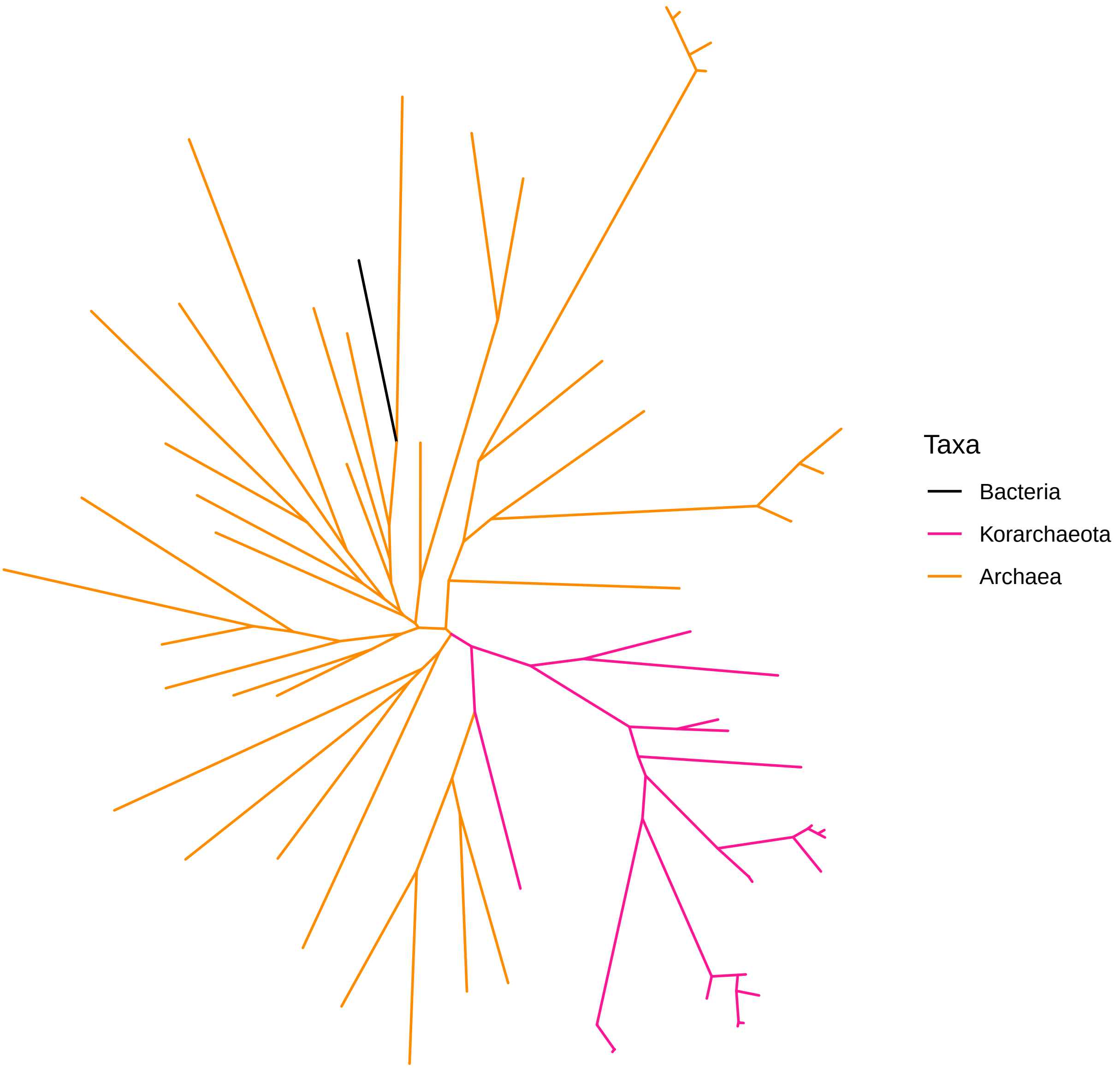

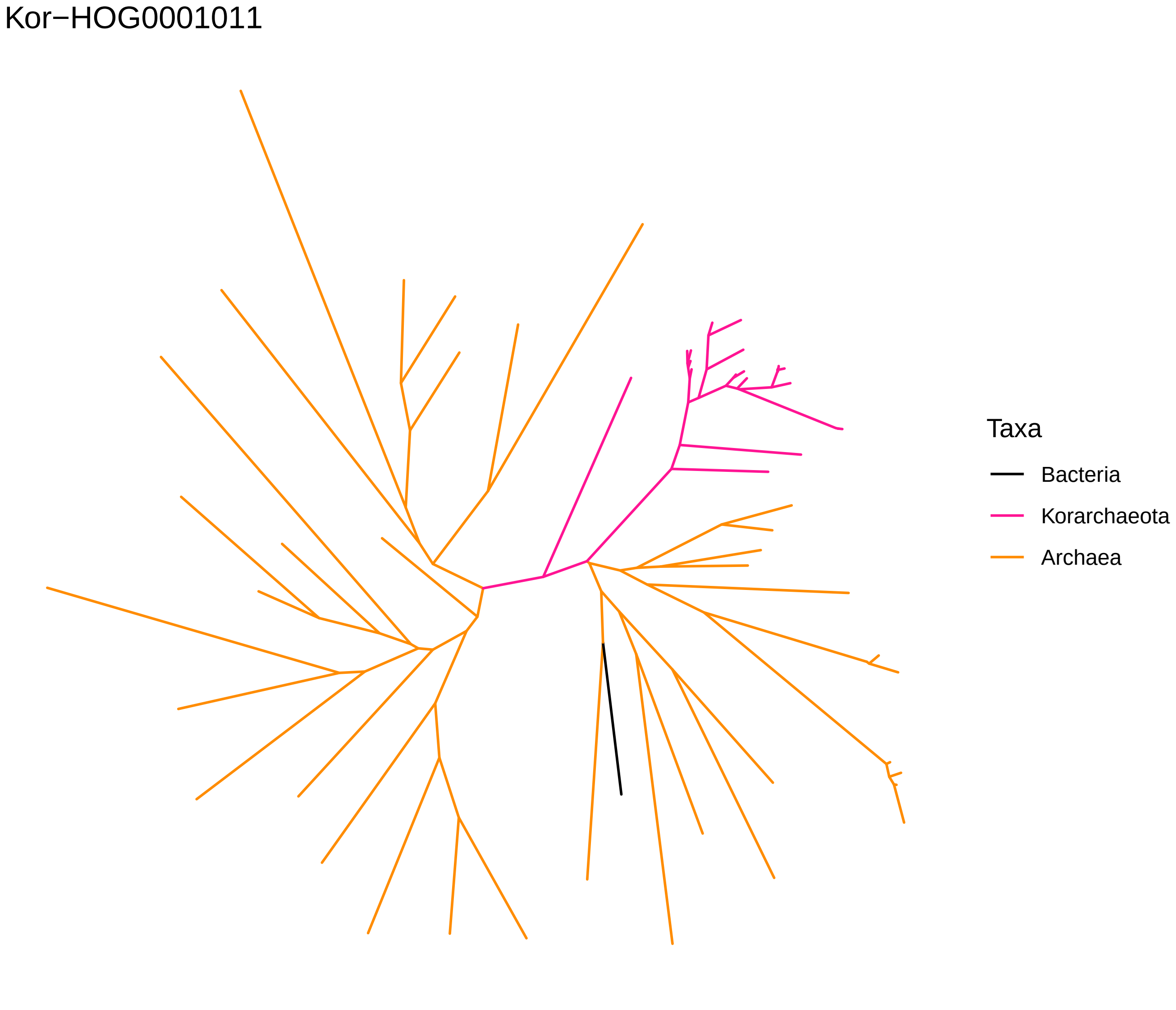

Kor−HOG0001011
Taxa
Bacteria
Korarchaeota
Archaea

Kor−HOG0001013

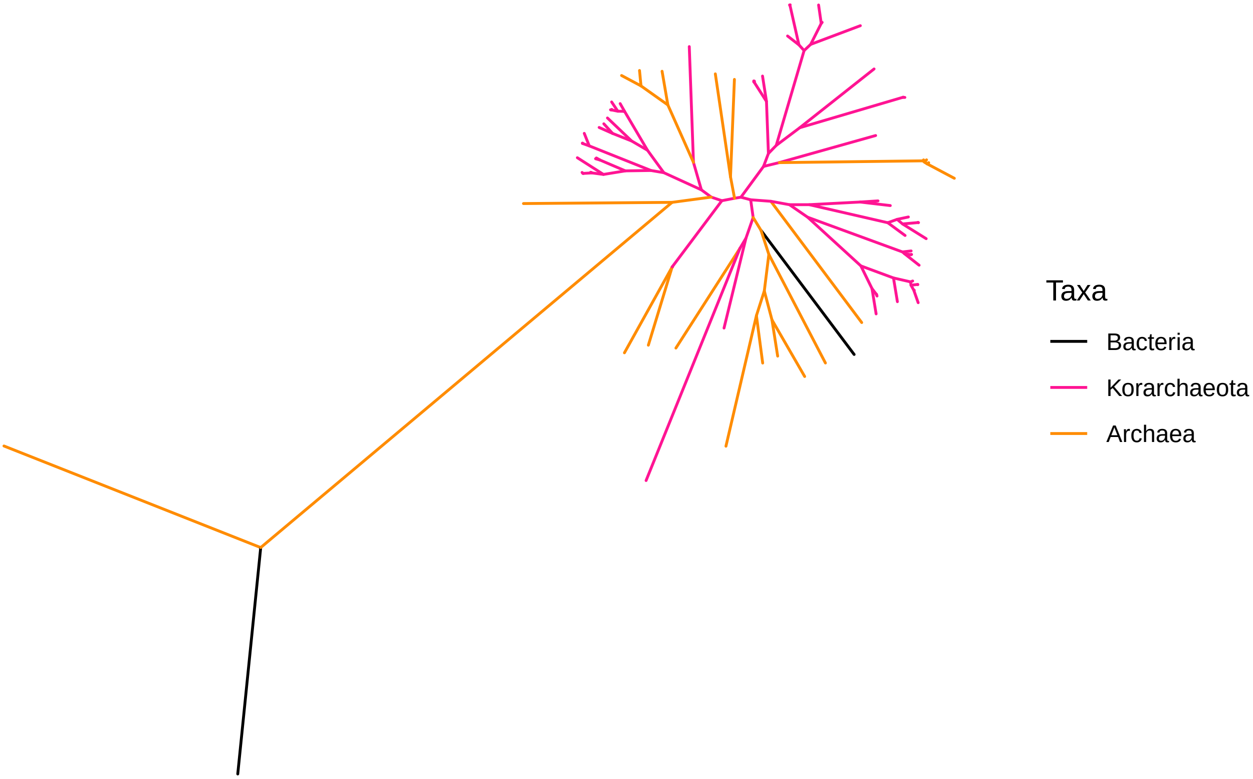

Kor−HOG0001017

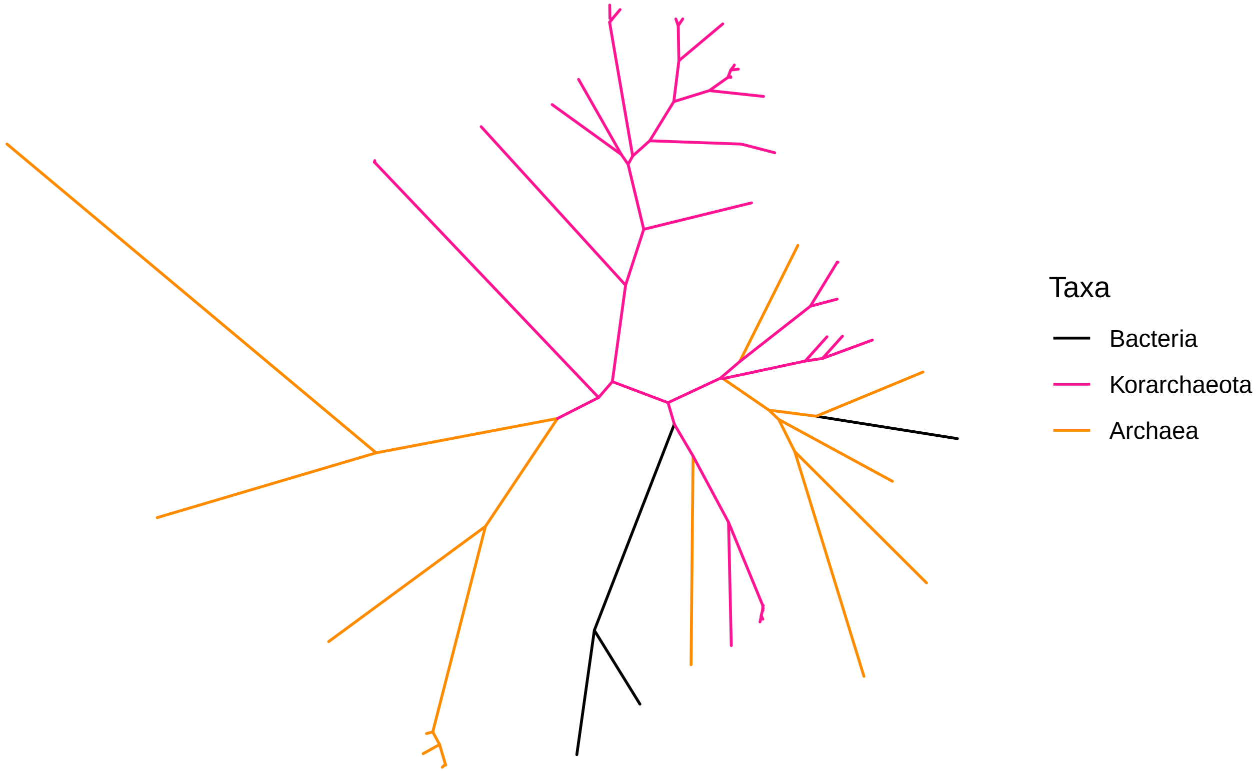

Kor−HOG0001018

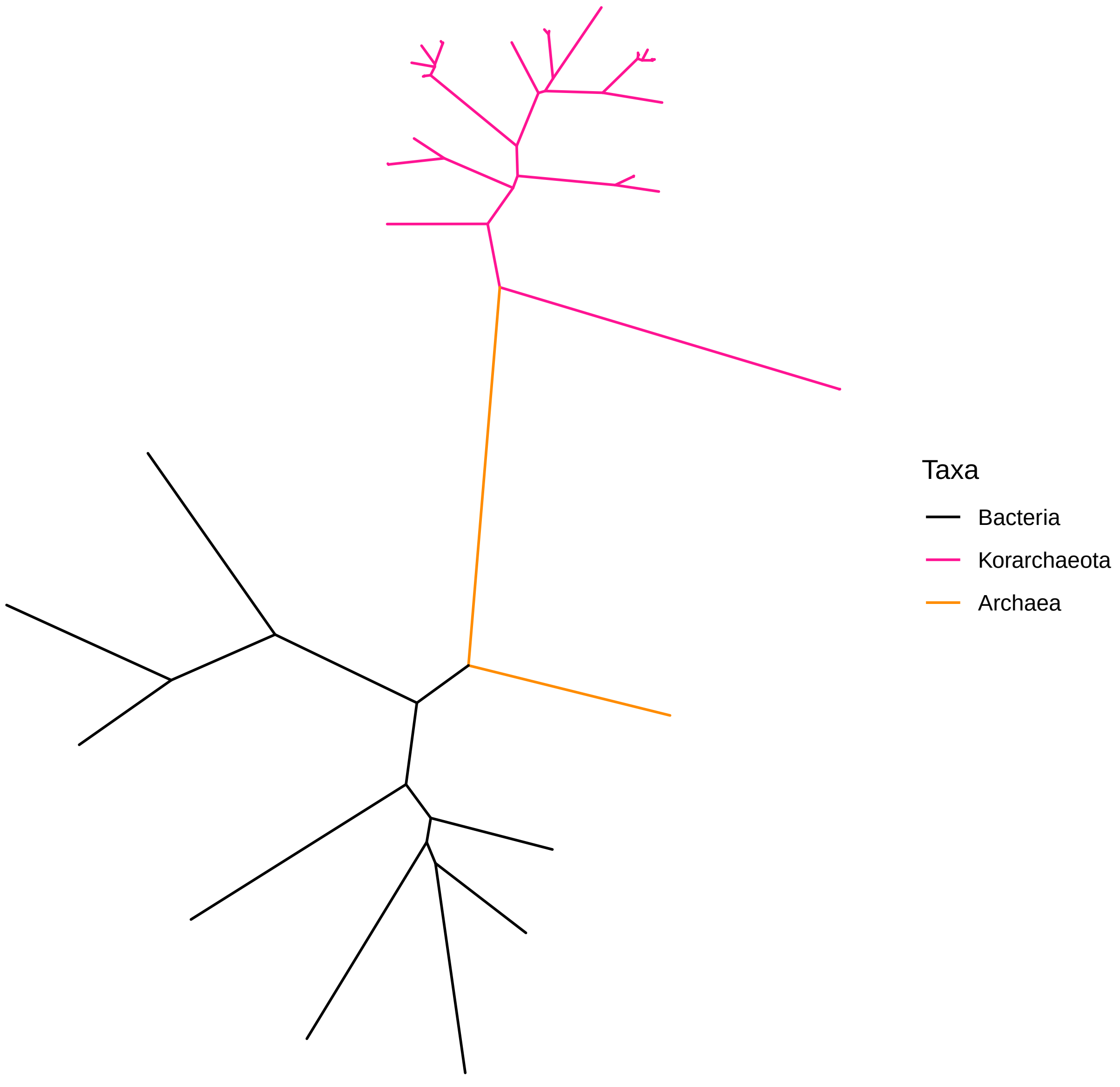

Kor−HOG0001021

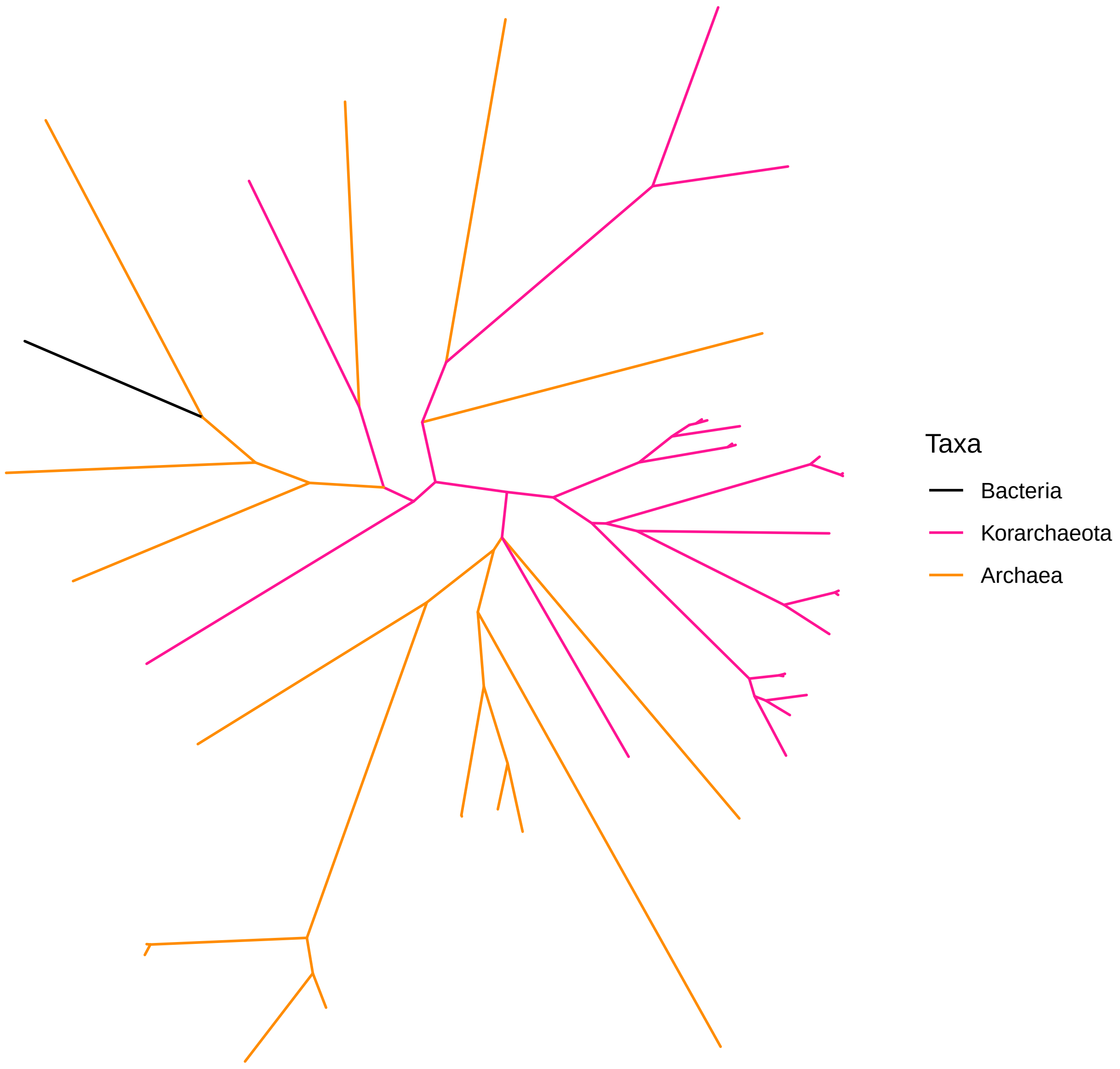

Kor−HOG0001022

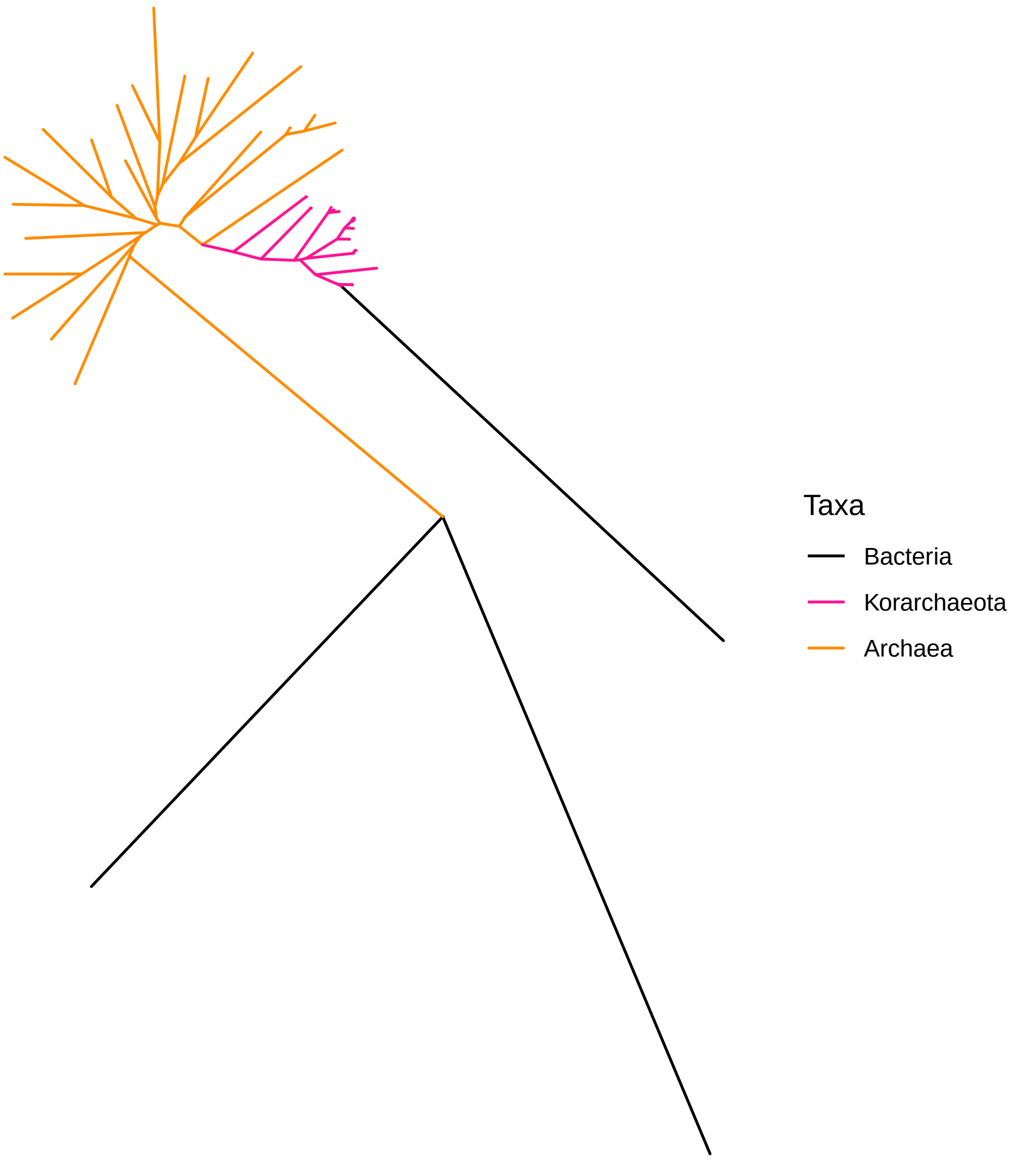

Kor−HOG0001023

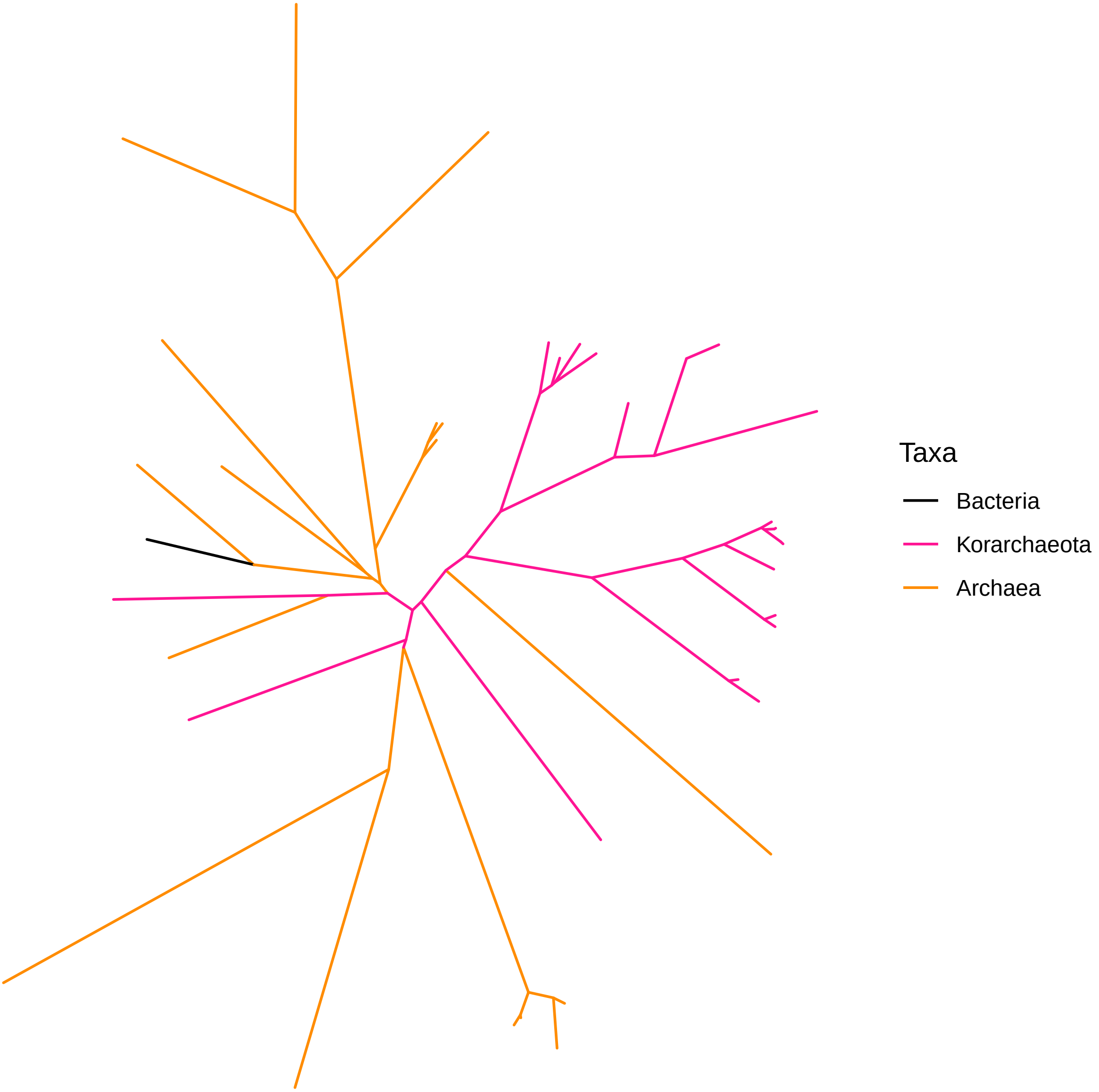

Kor−HOG0001029

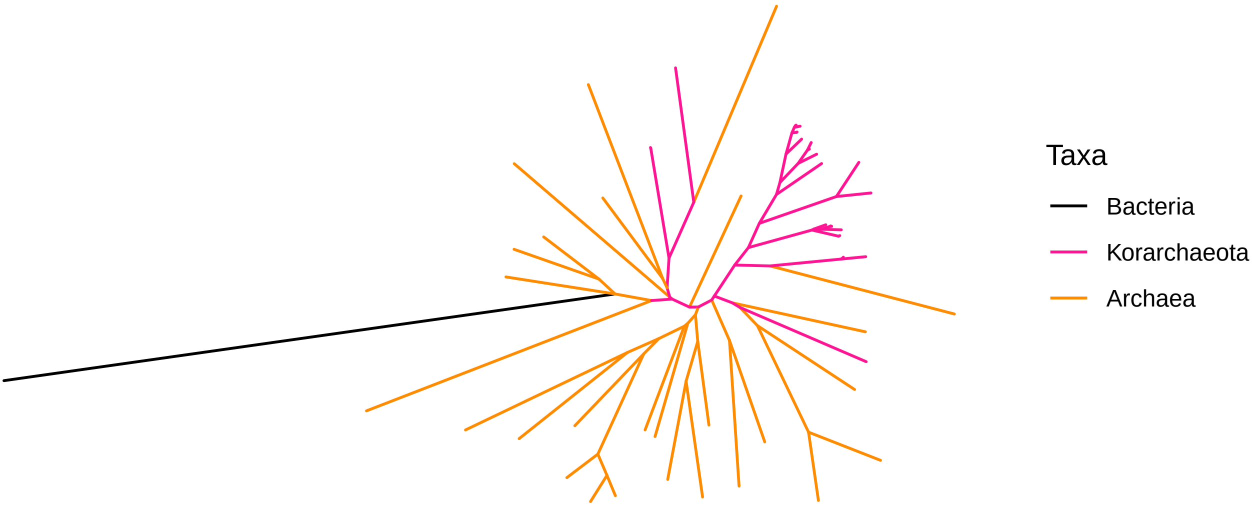

Kor−HOG0001031

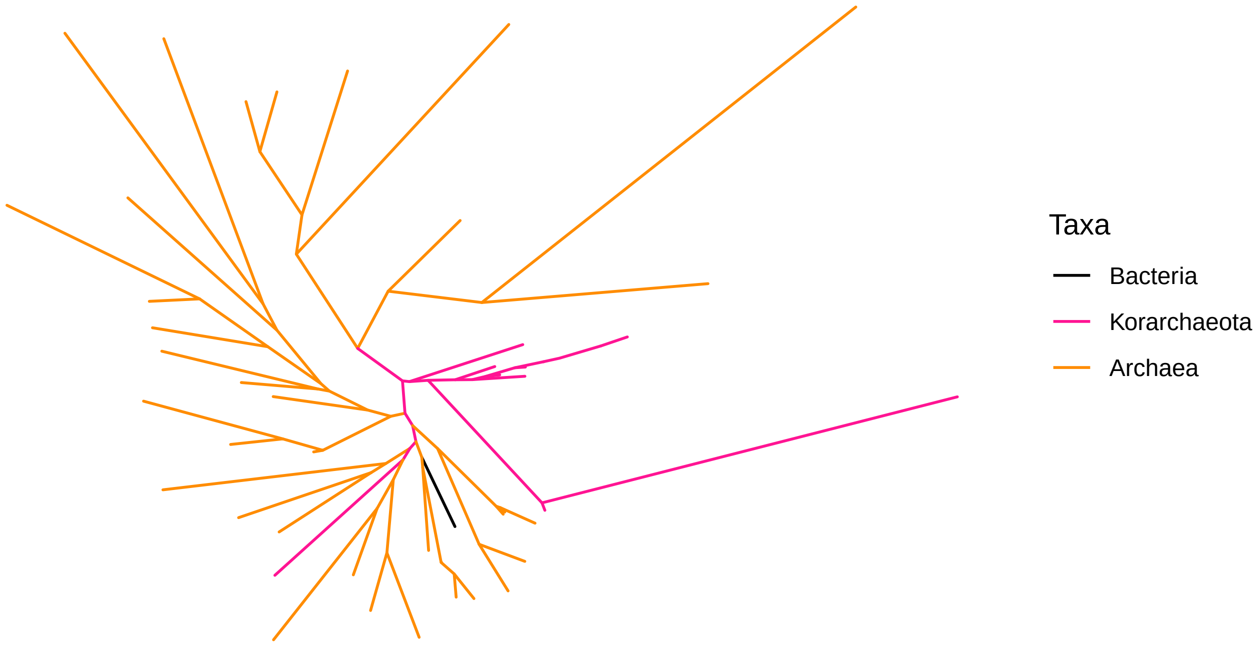

Kor−HOG0001033

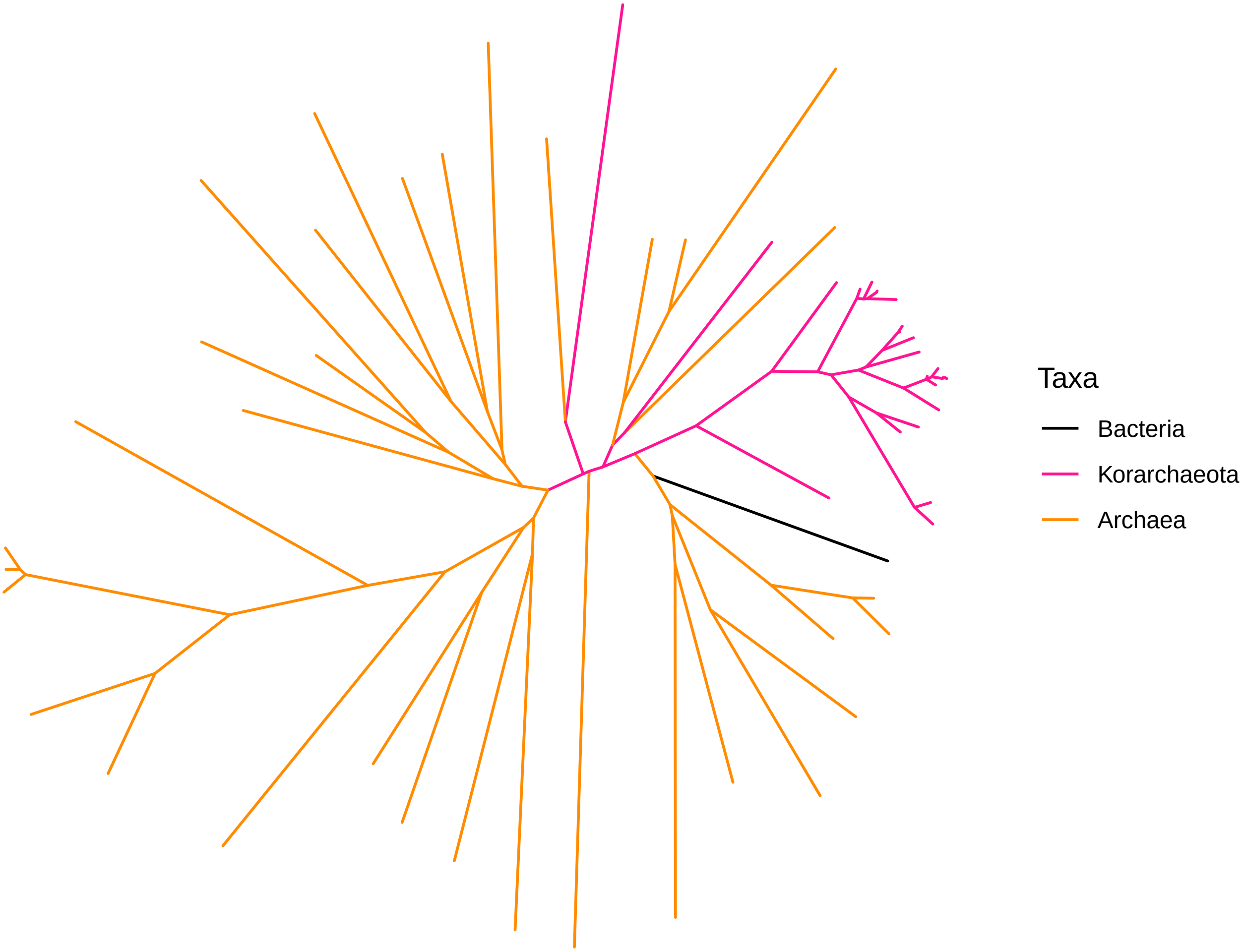

Kor−HOG0001040

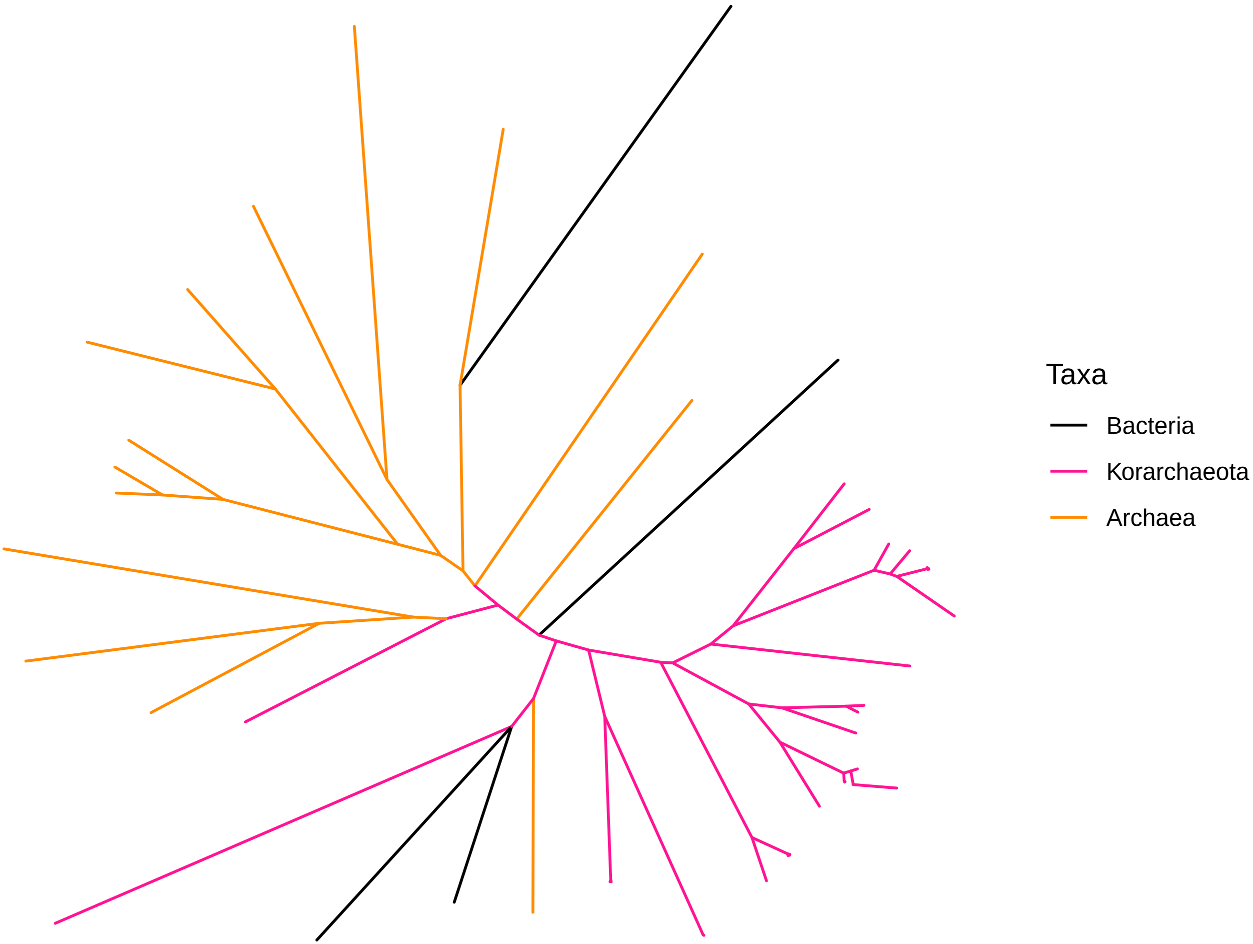

Kor−HOG0001045

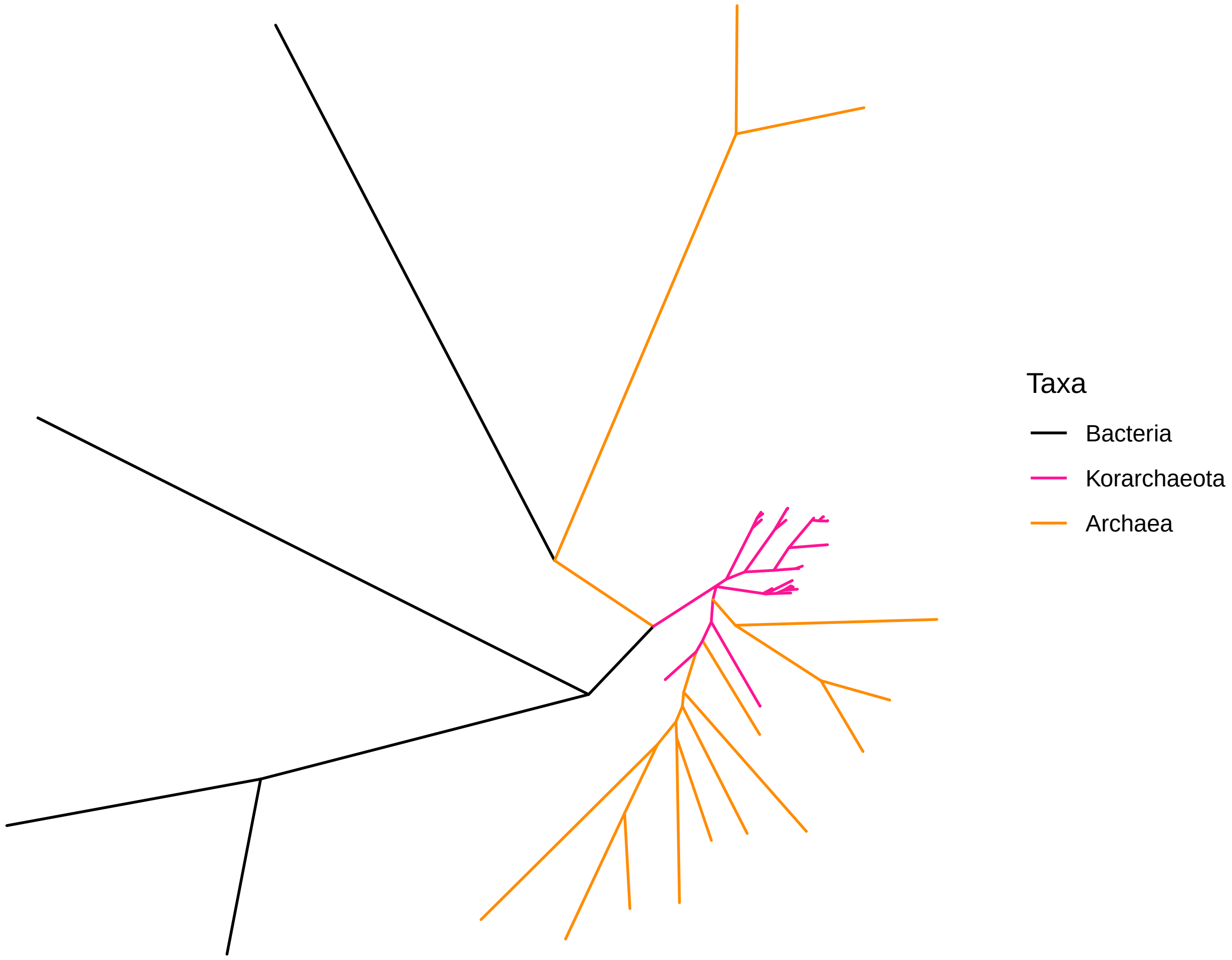

Kor−HOG0001046

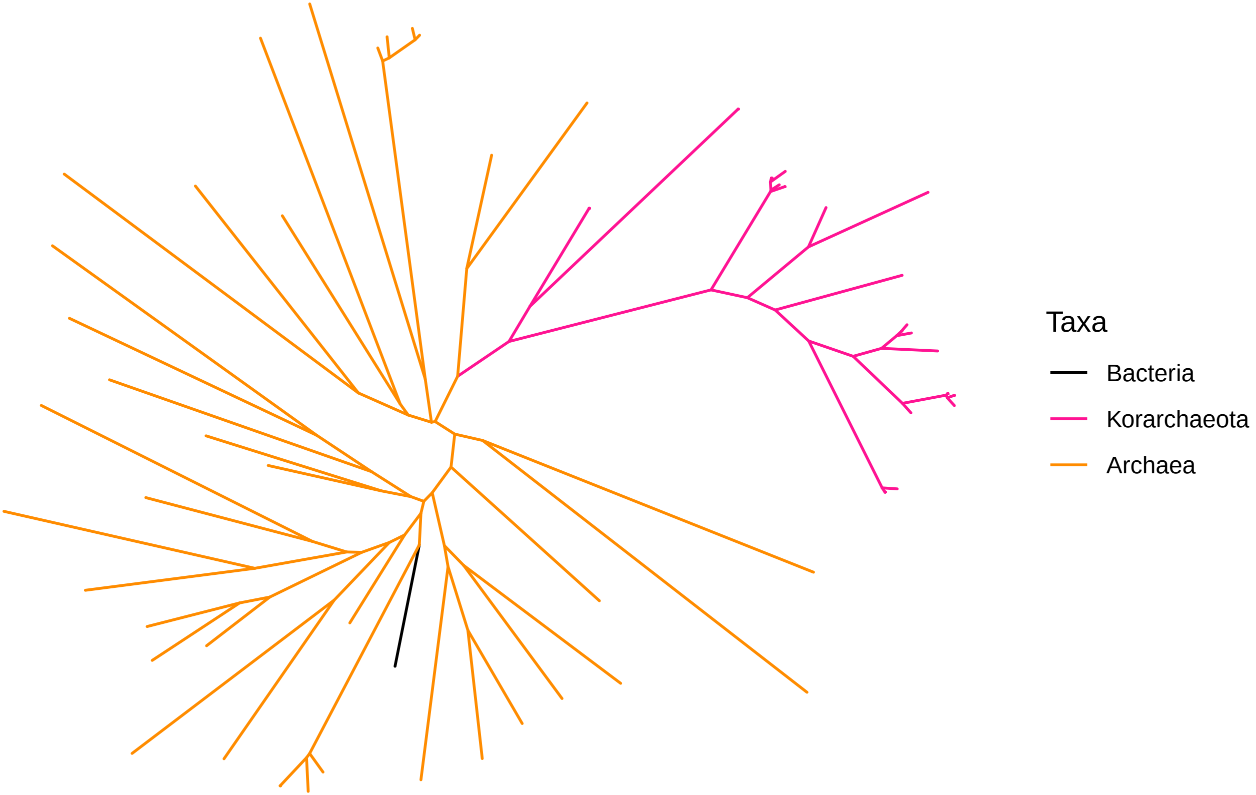

Kor−HOG0001047

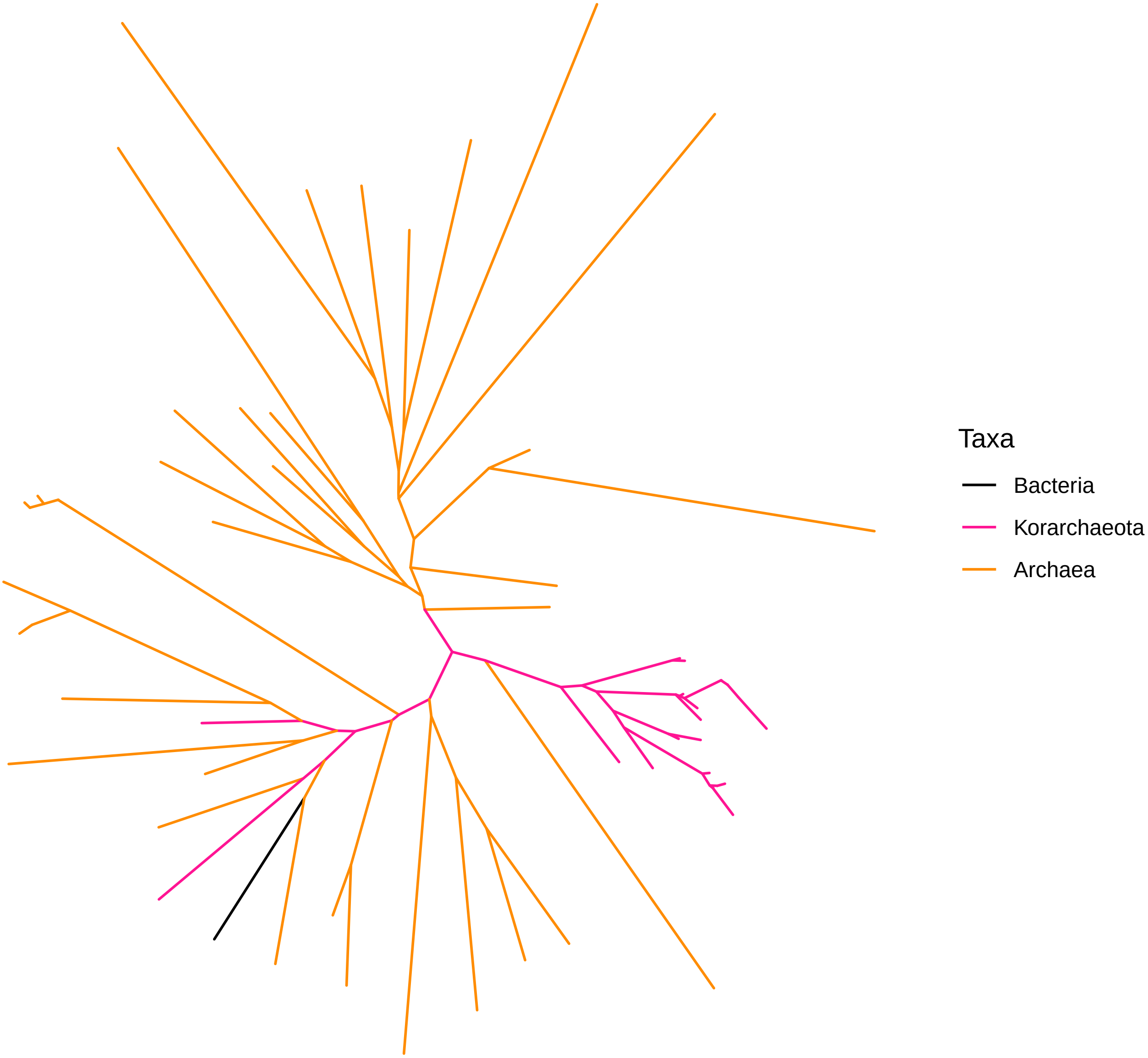

Kor−HOG0001050

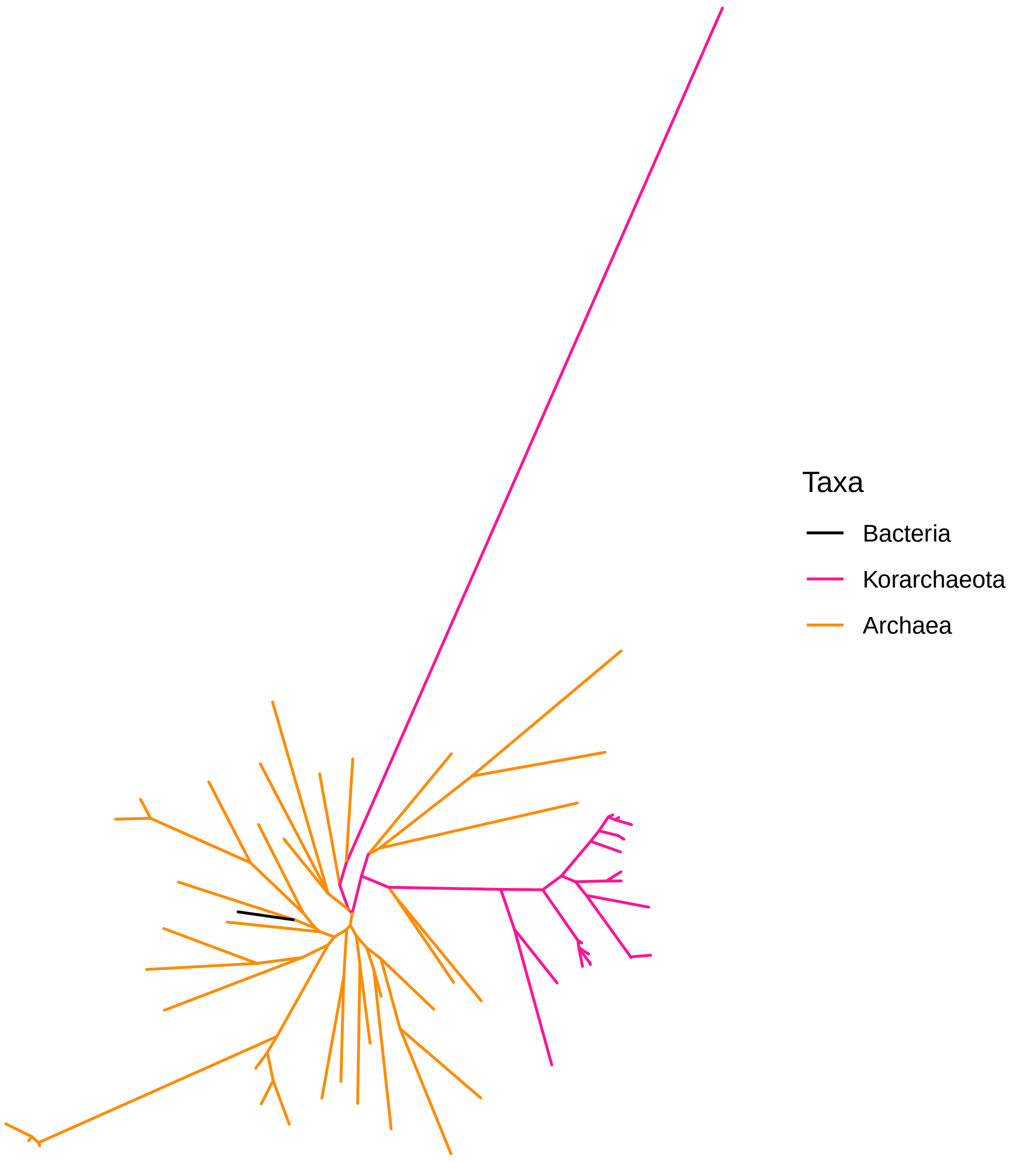

Kor−HOG0001095

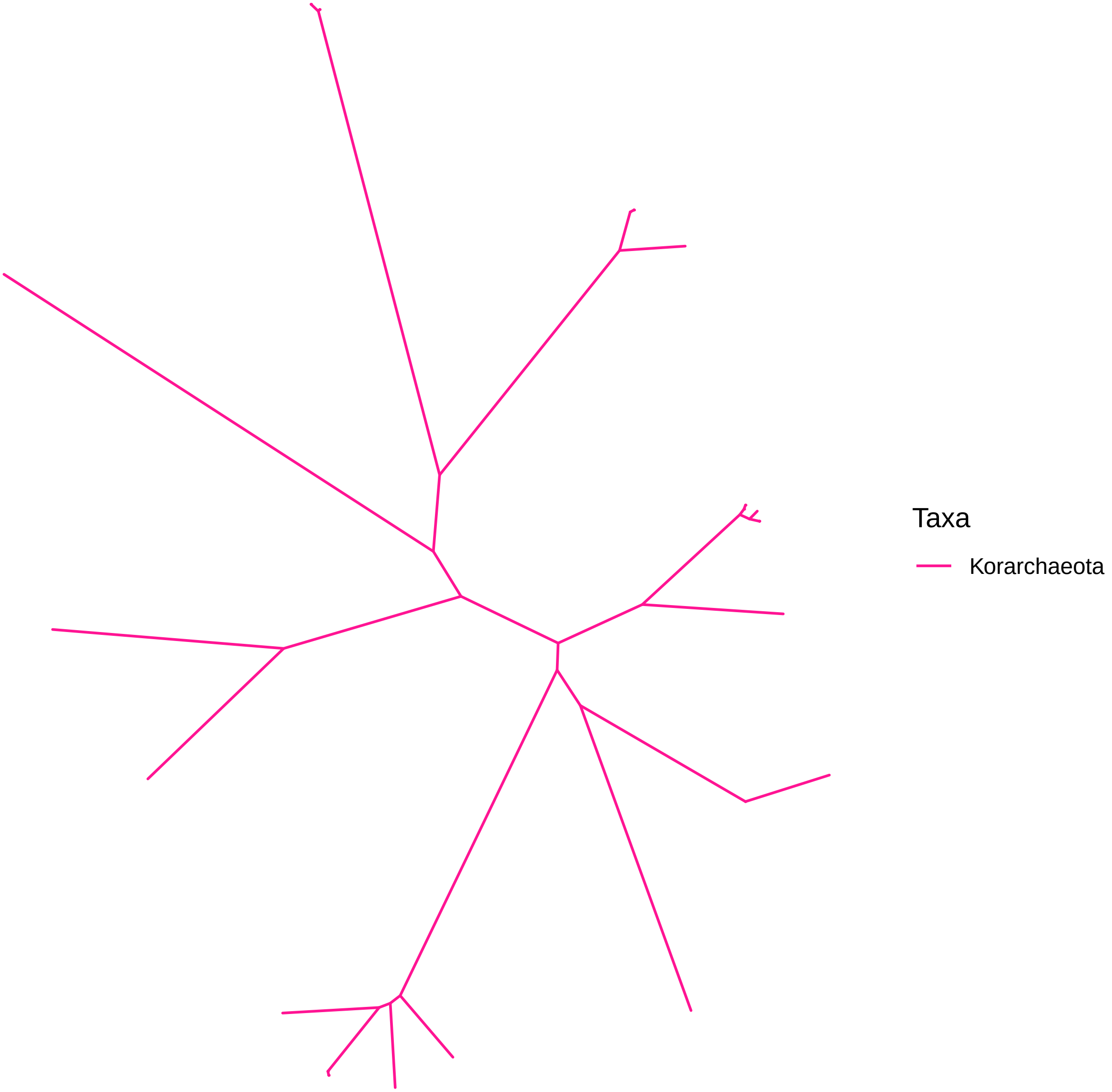

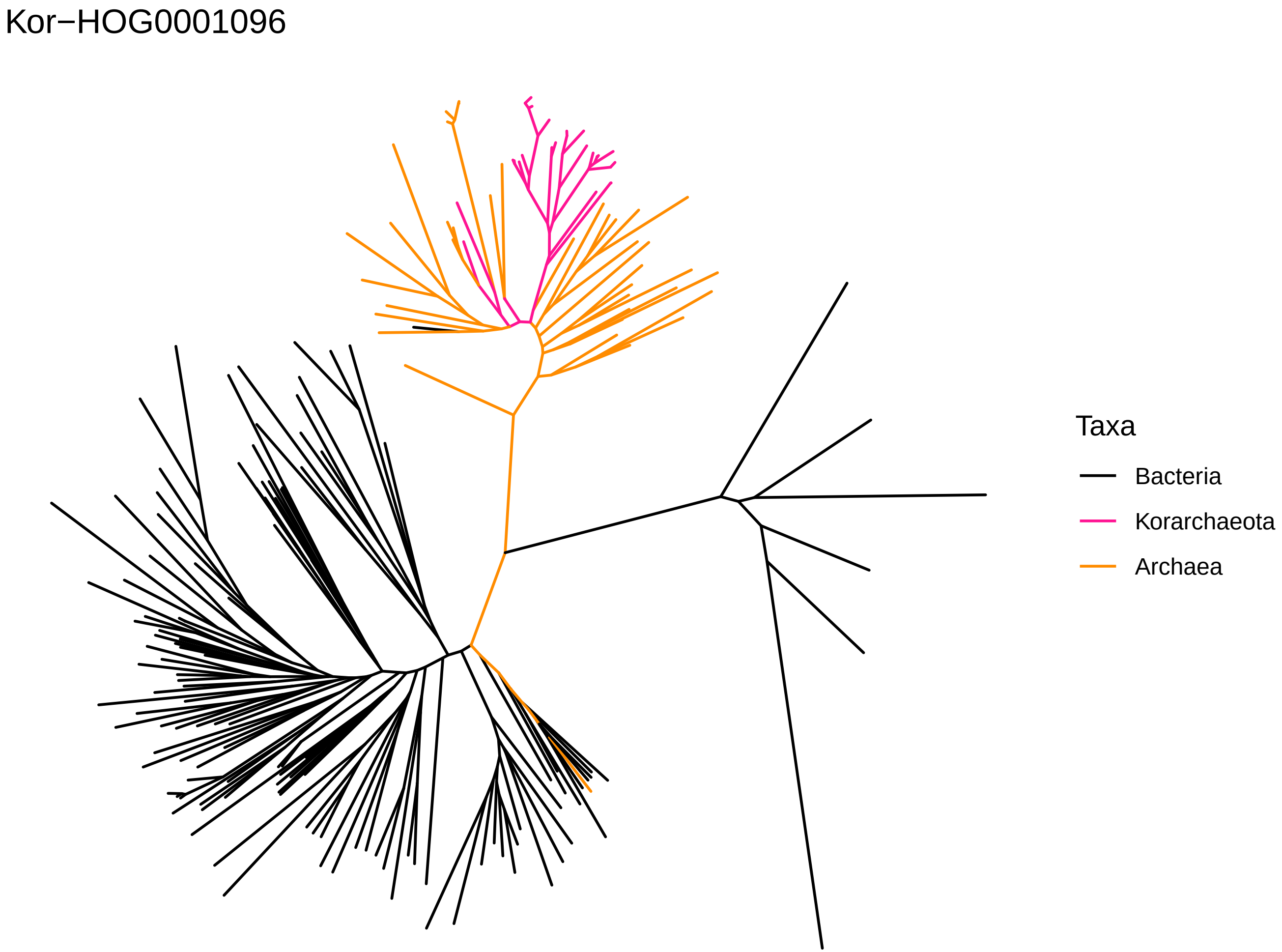

Kor−HOG0001096
Taxa
Bacteria
Korarchaeota
Archaea

Kor−HOG0001102

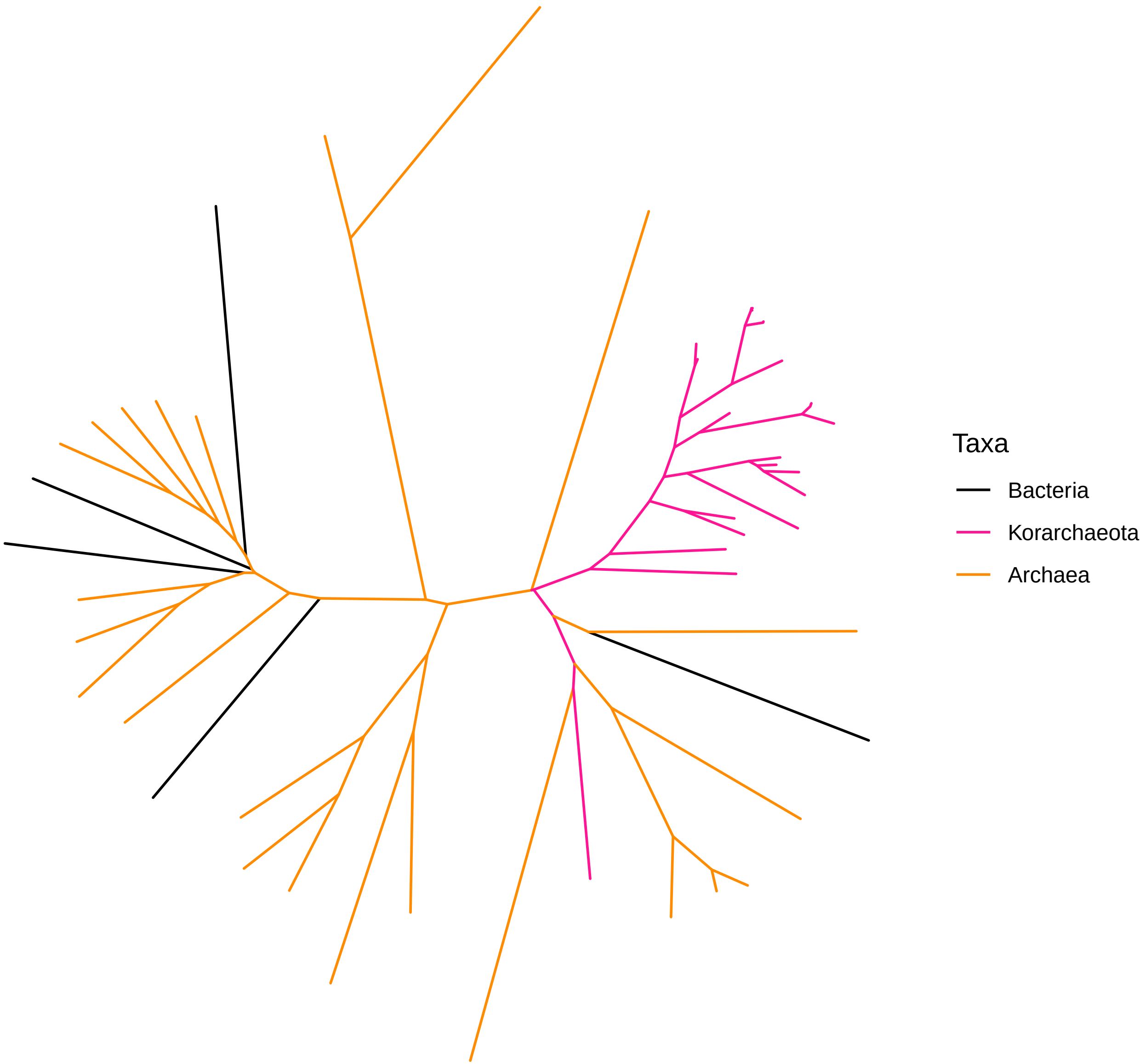

Kor−HOG0001104

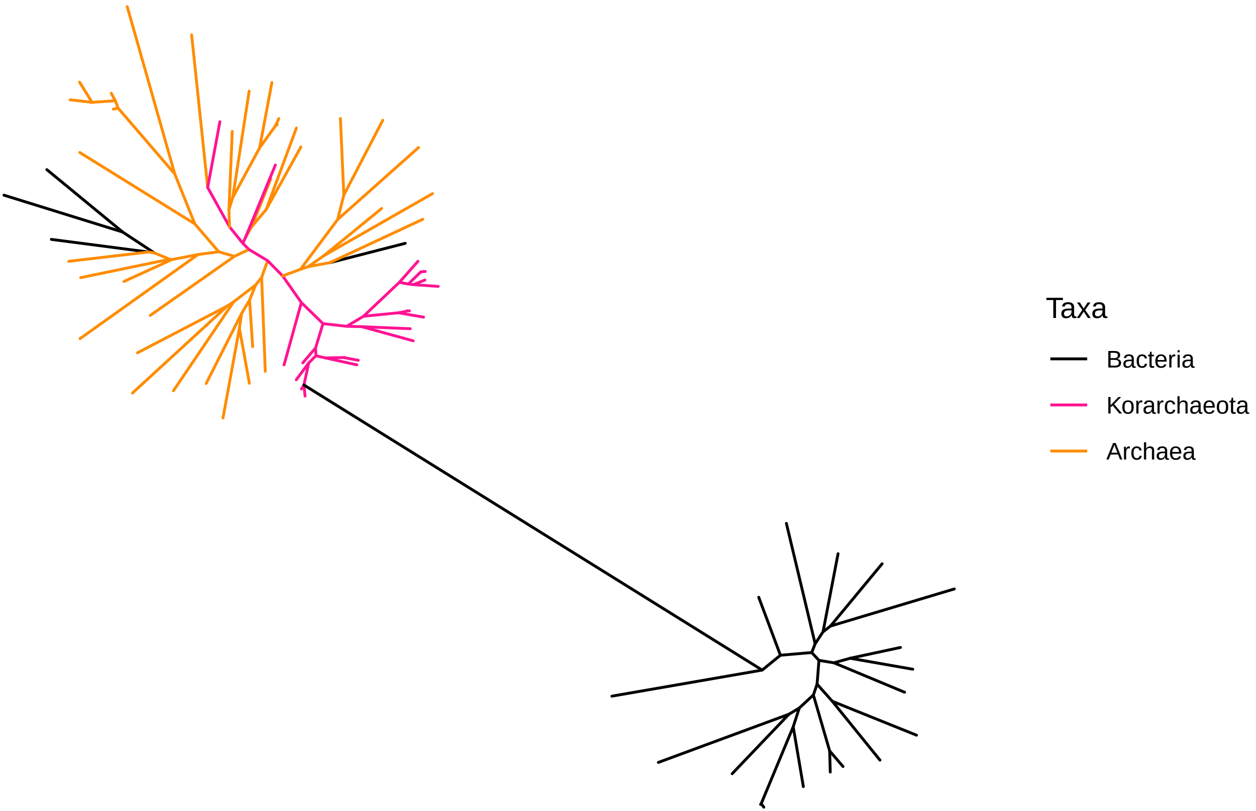

Kor−HOG0001106

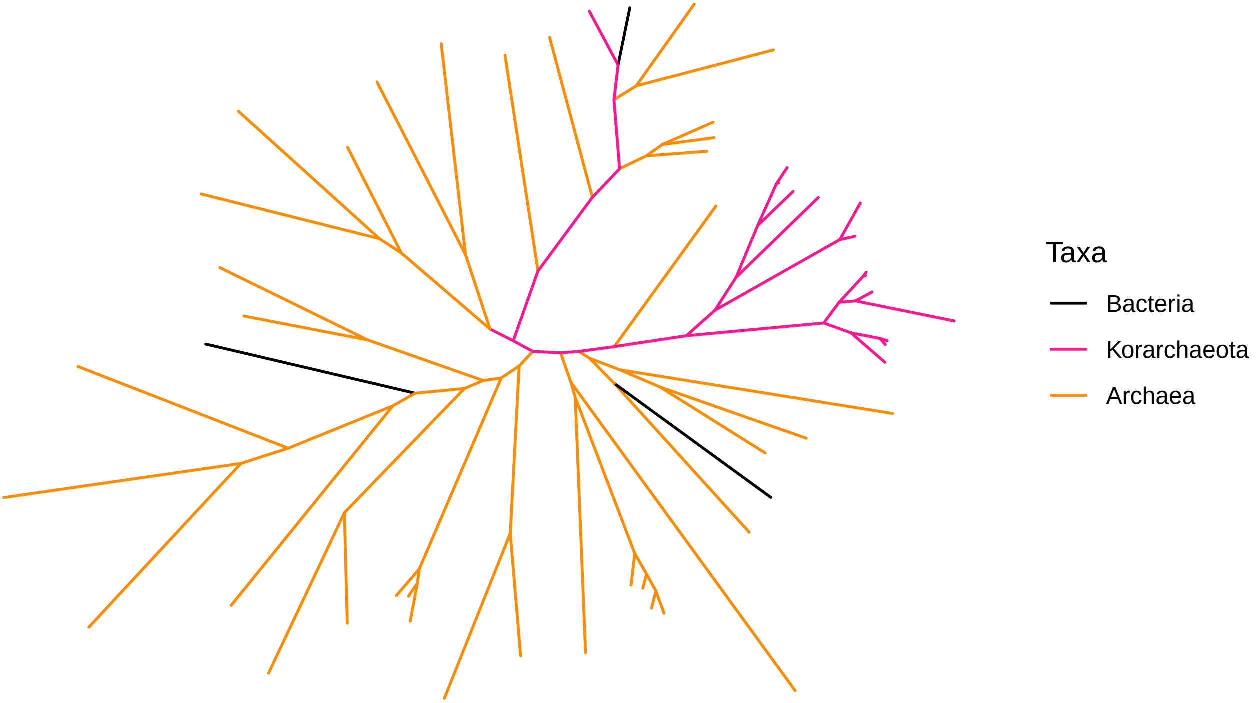

Kor−HOG0001114

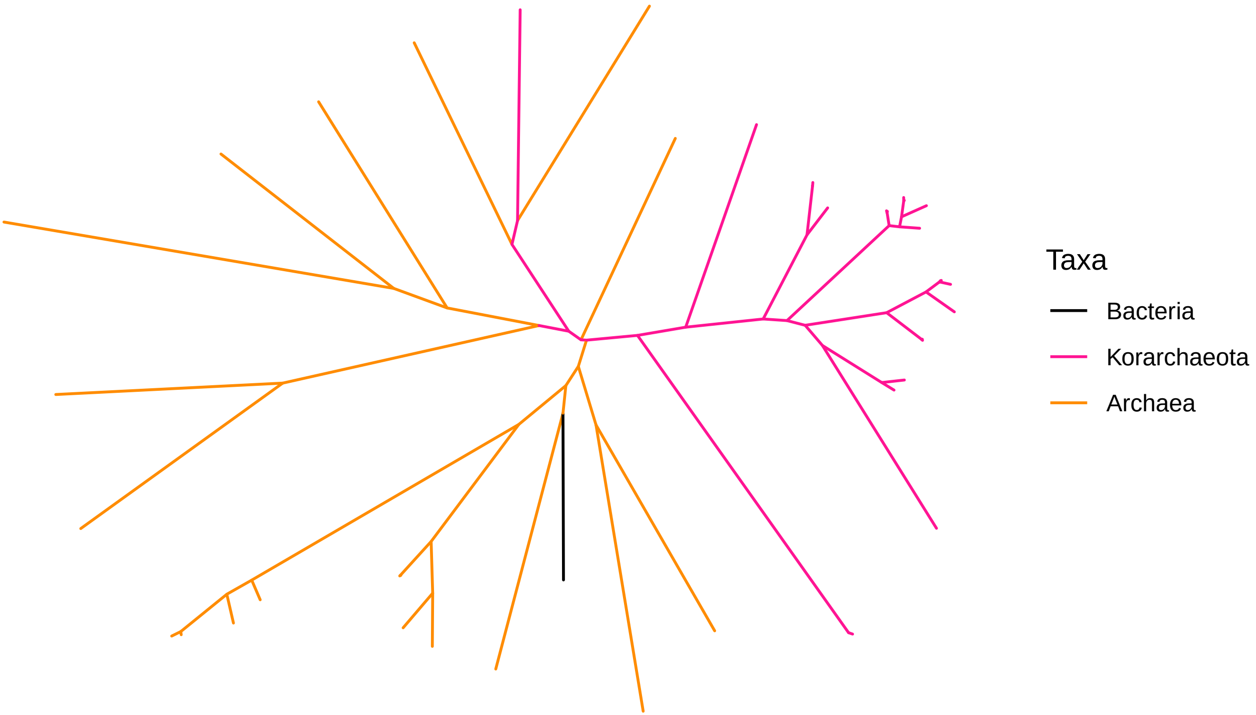

Kor−HOG0001116

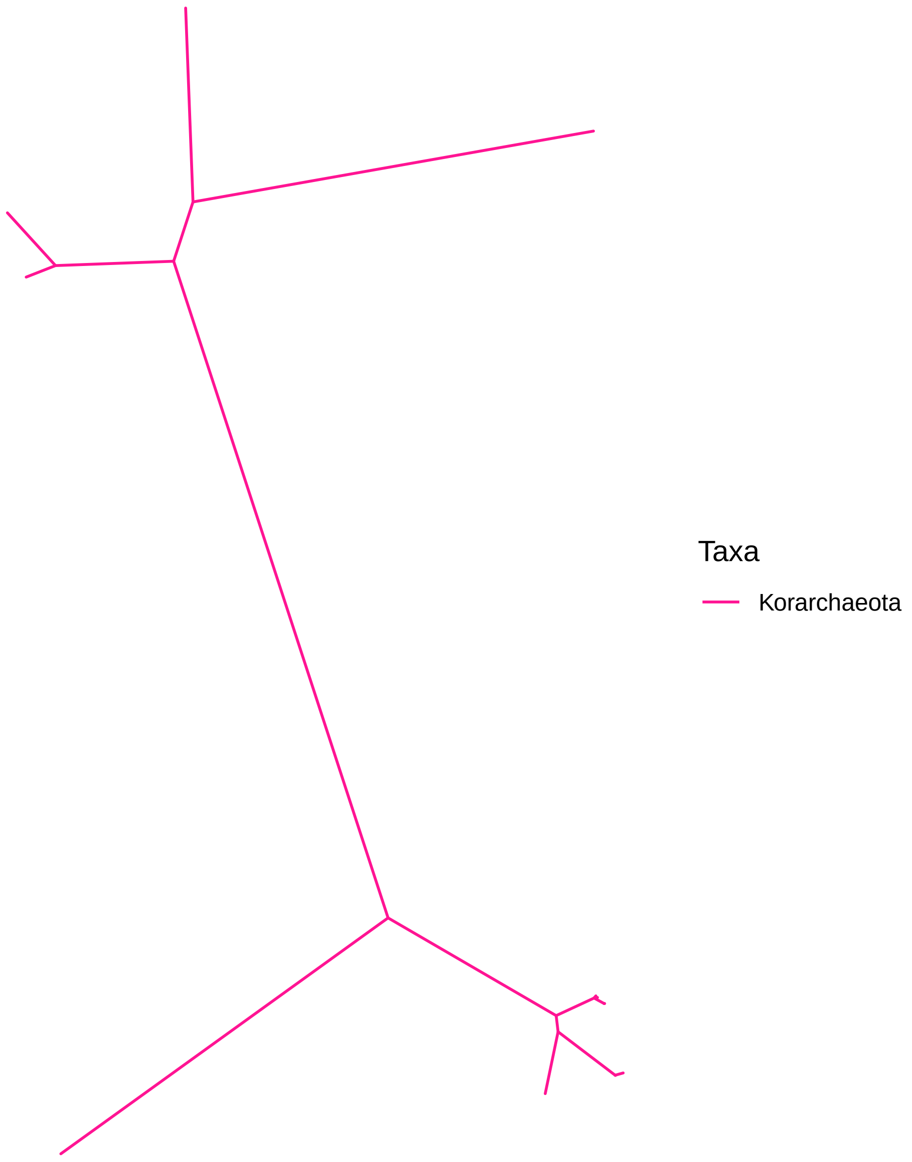

Kor−HOG0001129

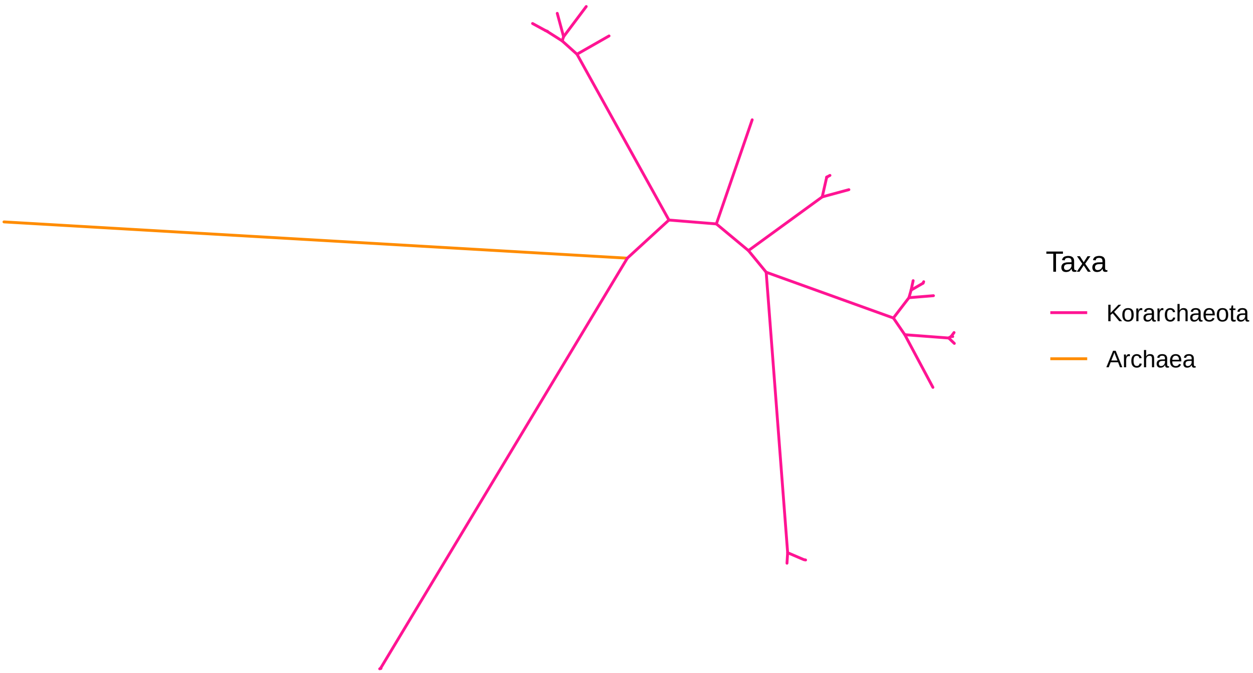

Kor−HOG0001131

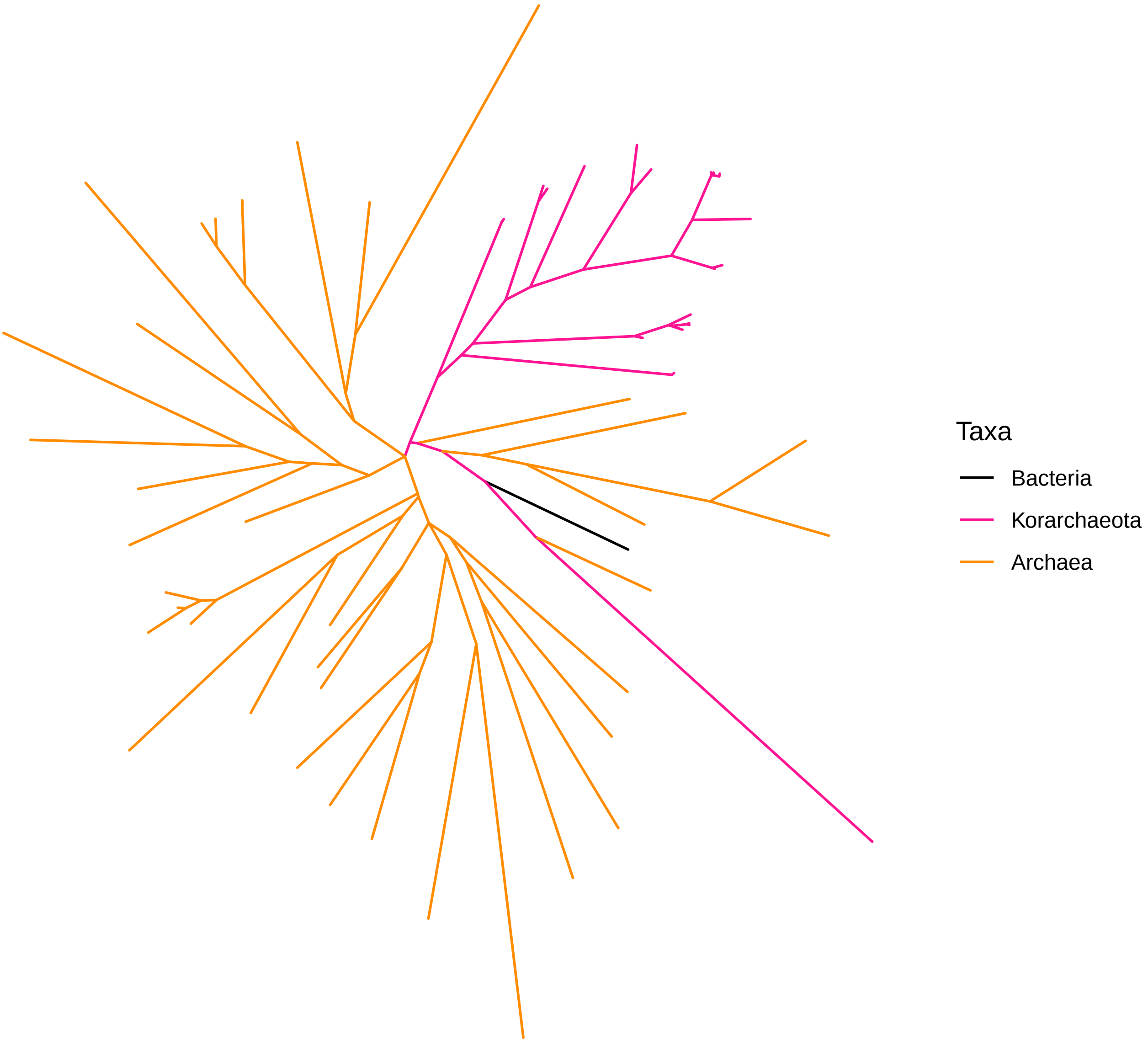

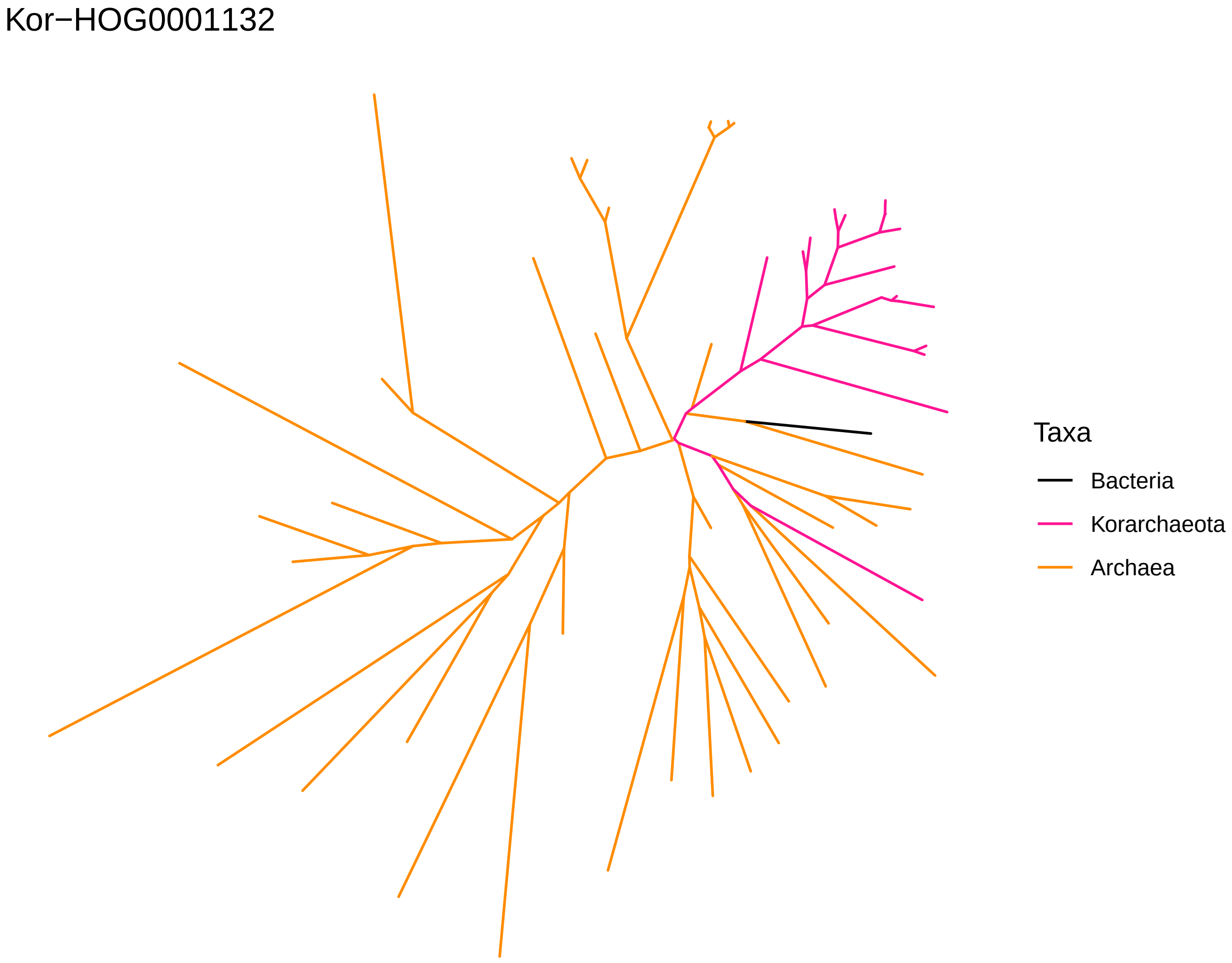
Kor−HOG0001132
Taxa
Bacteria
Korarchaeota
Archaea

Kor−HOG0001134

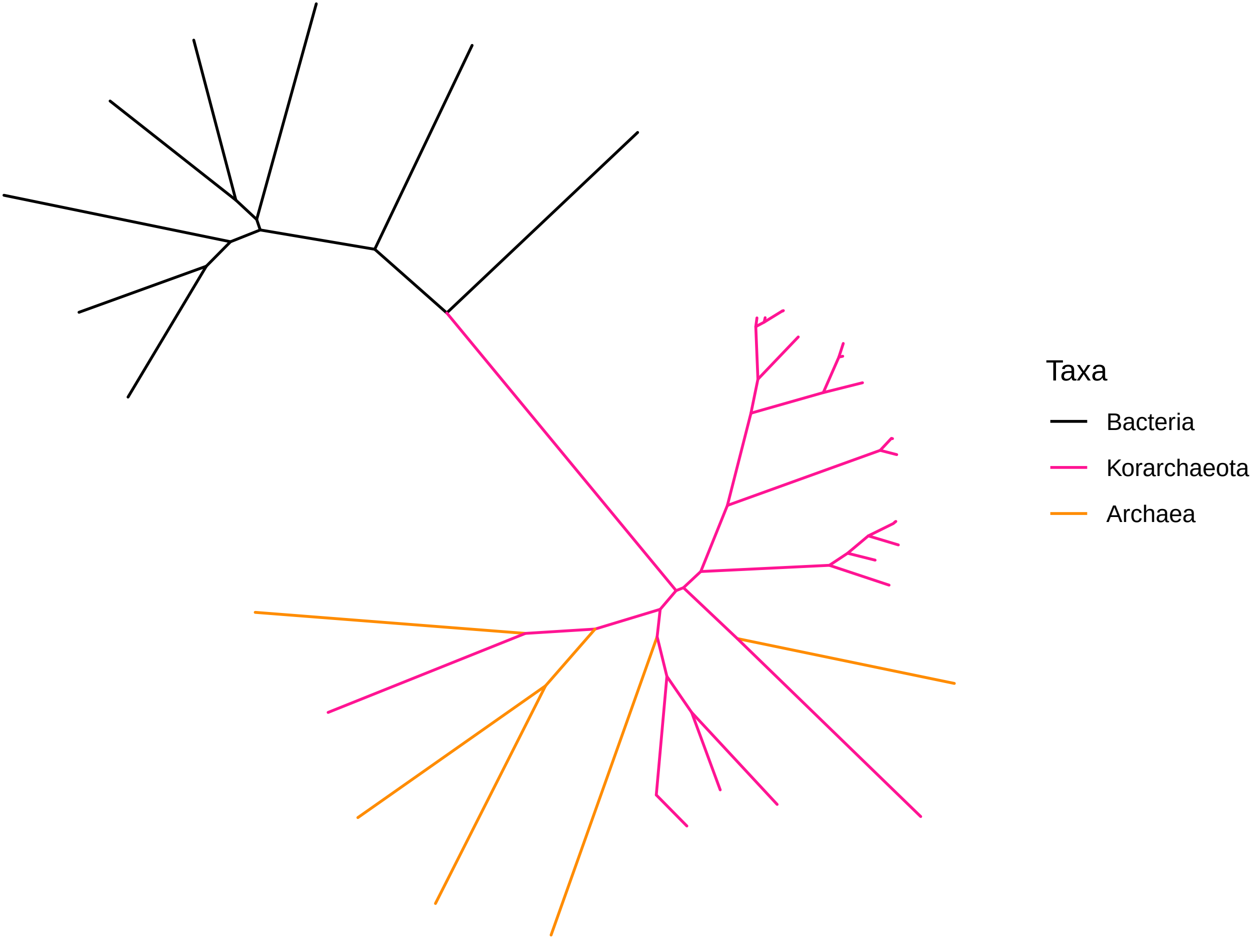

Kor−HOG0001139

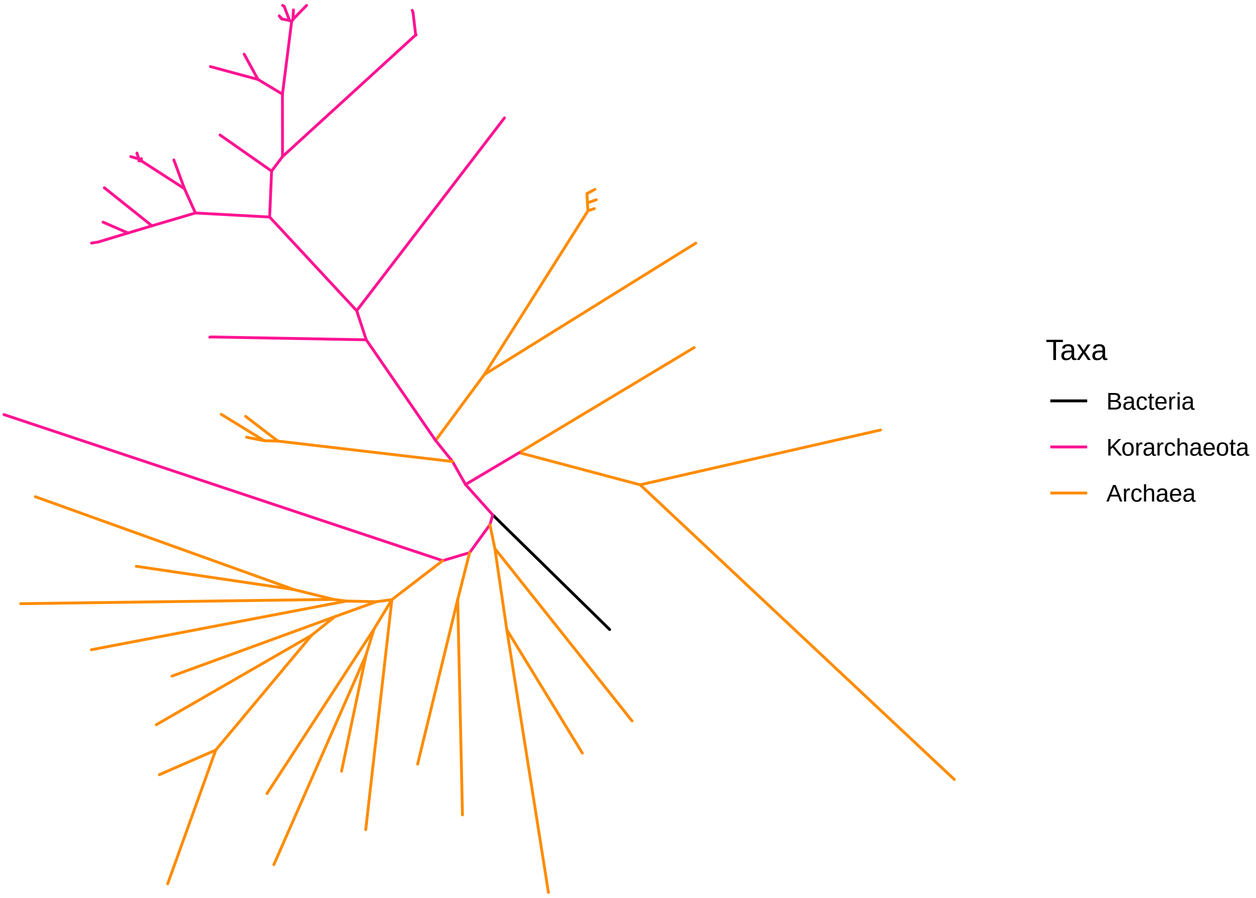

Kor−HOG0001140

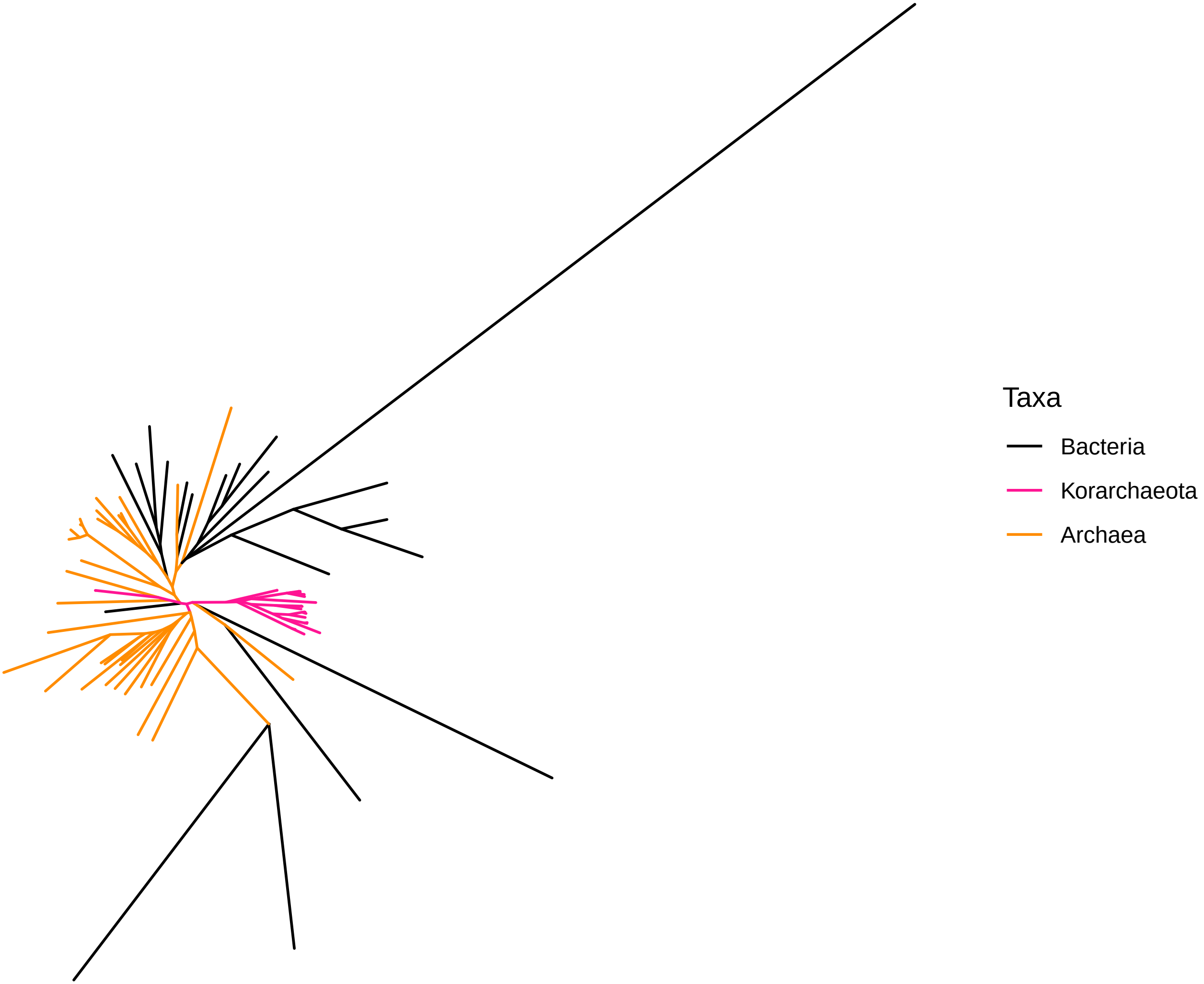

Kor−HOG0001188

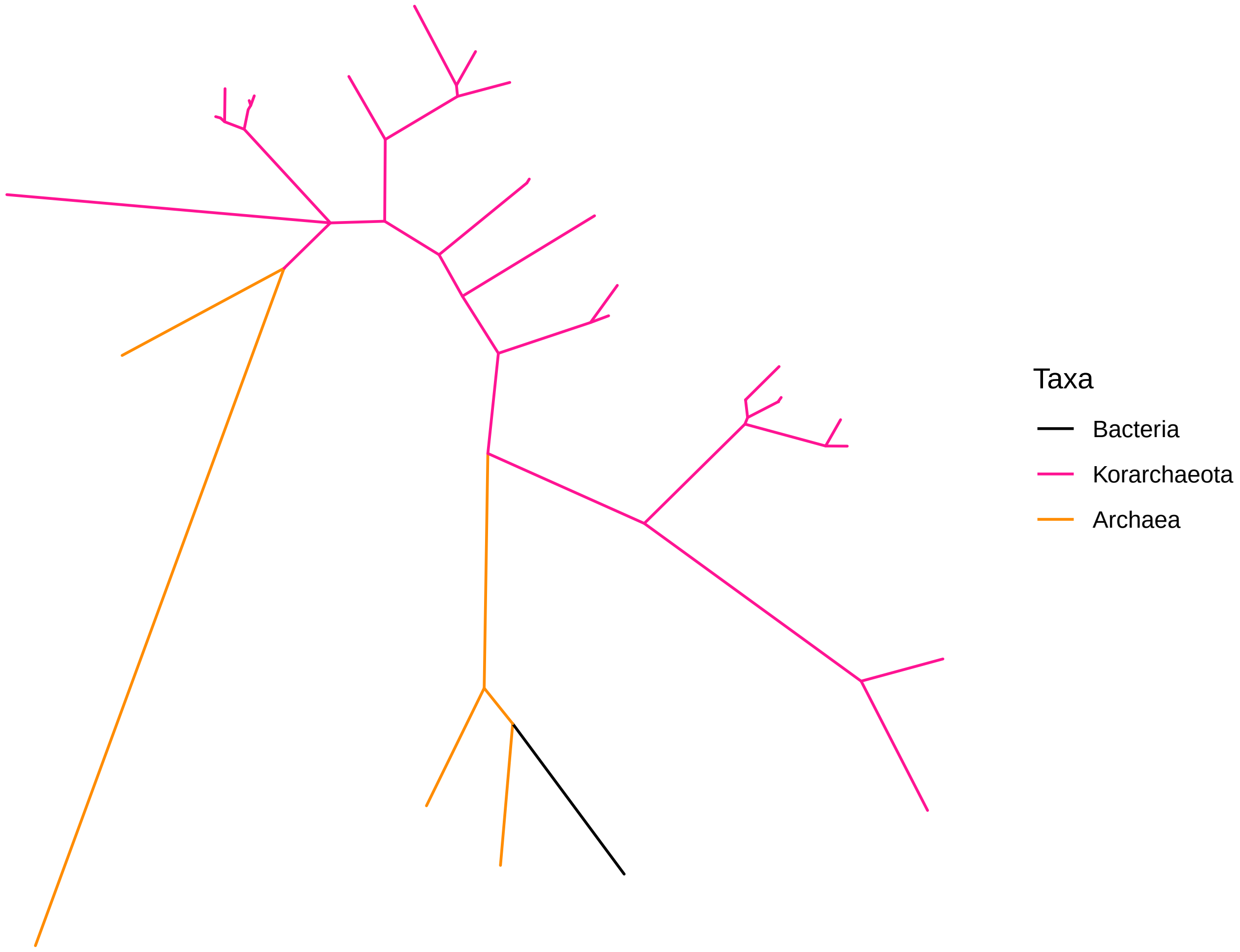

Kor−HOG0001220

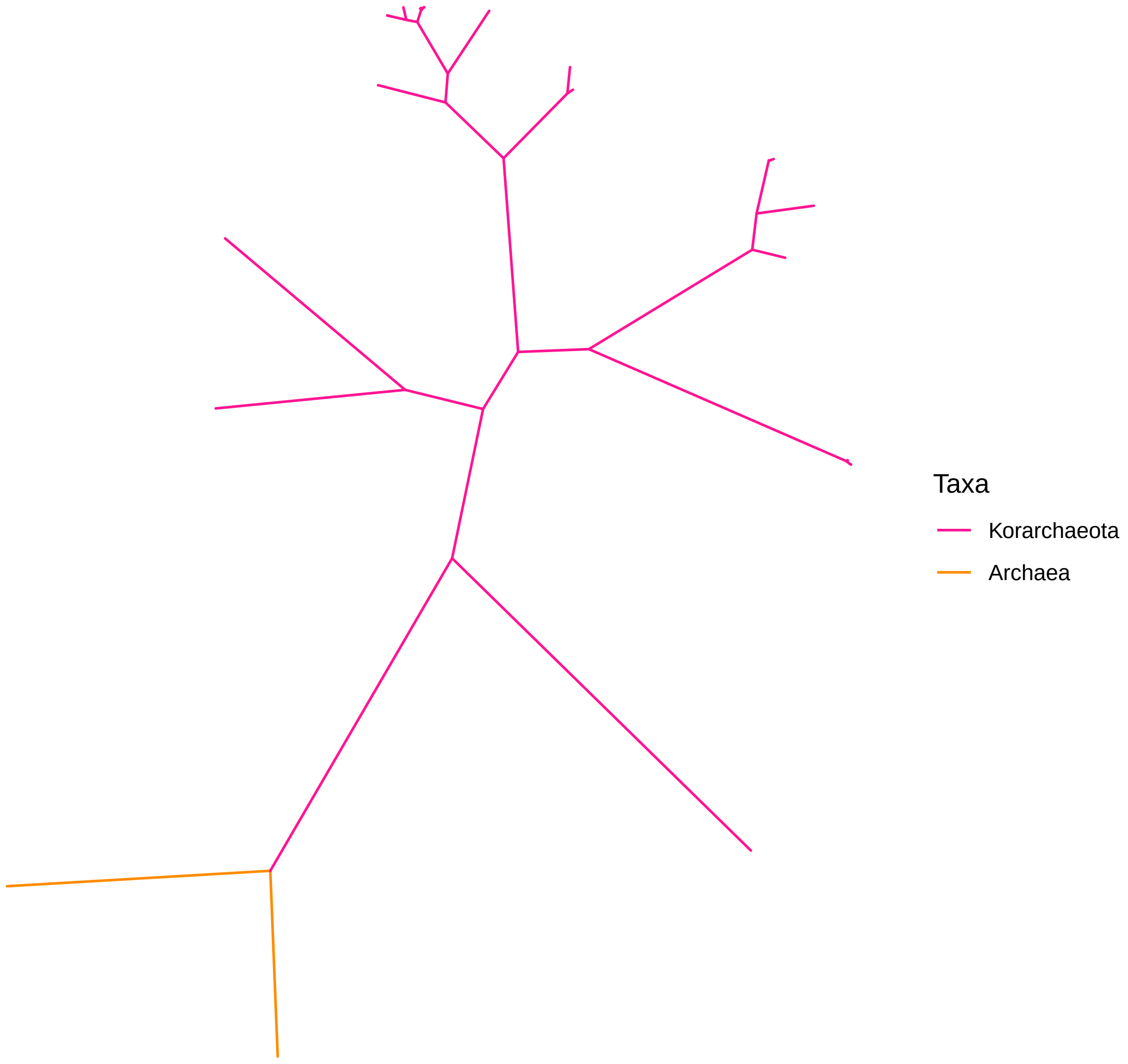

Kor−HOG0001222

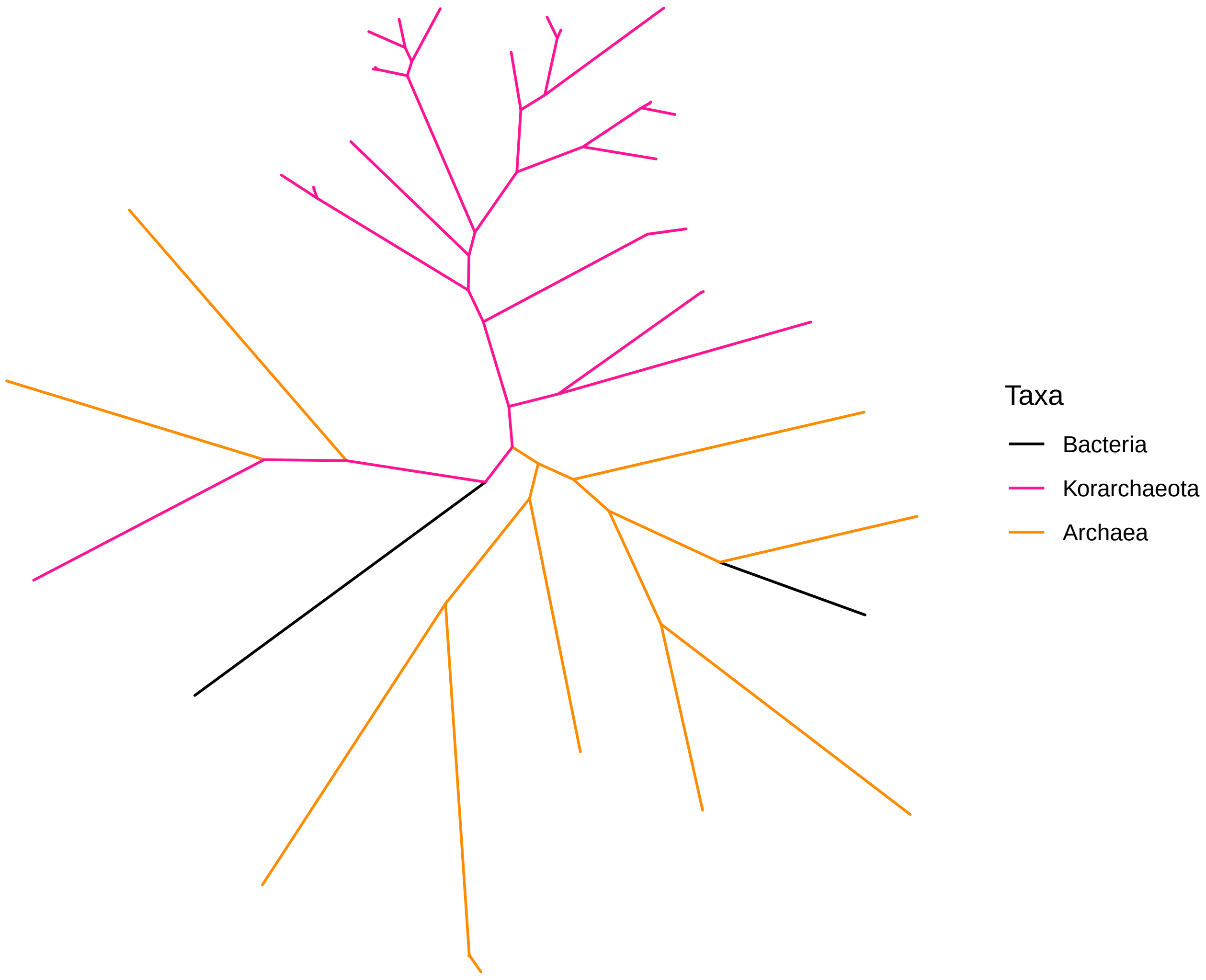

Kor−HOG0001247

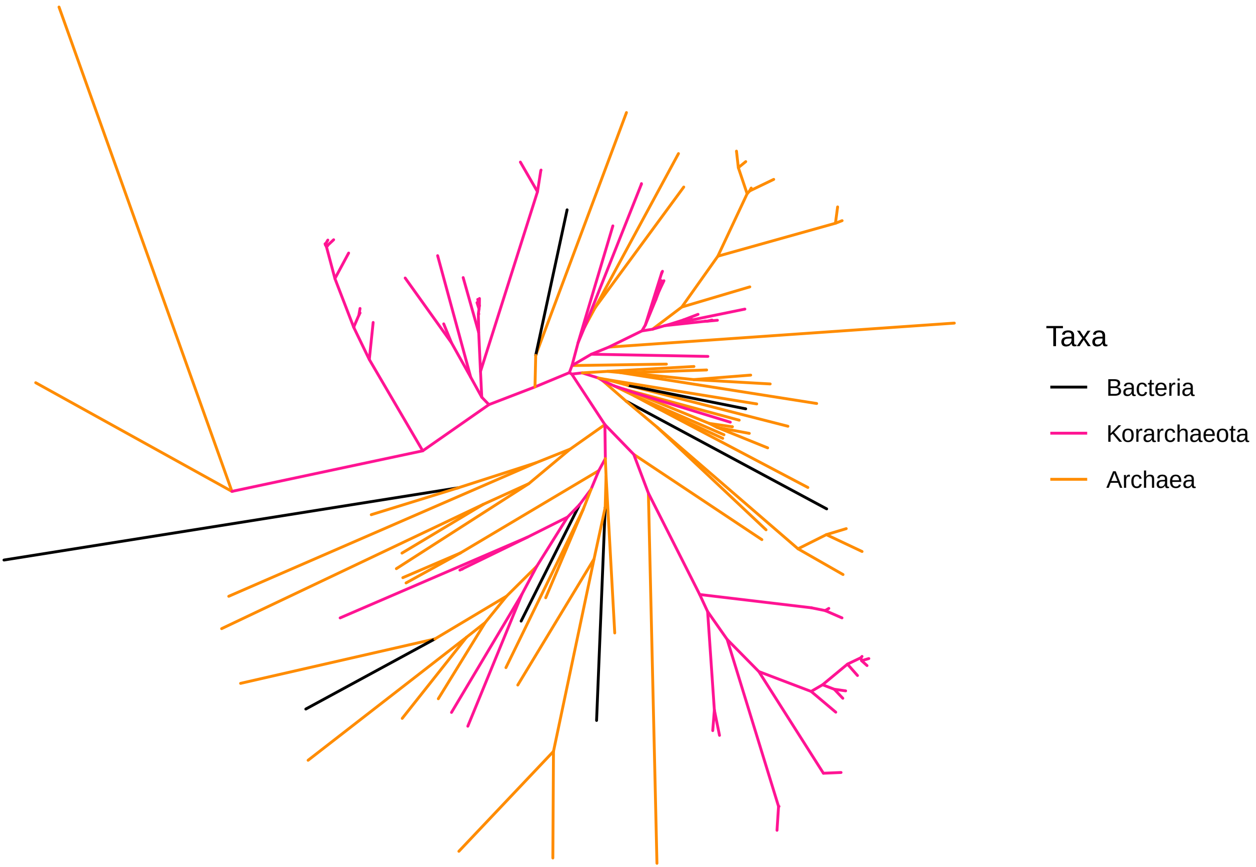

Kor−HOG0001253

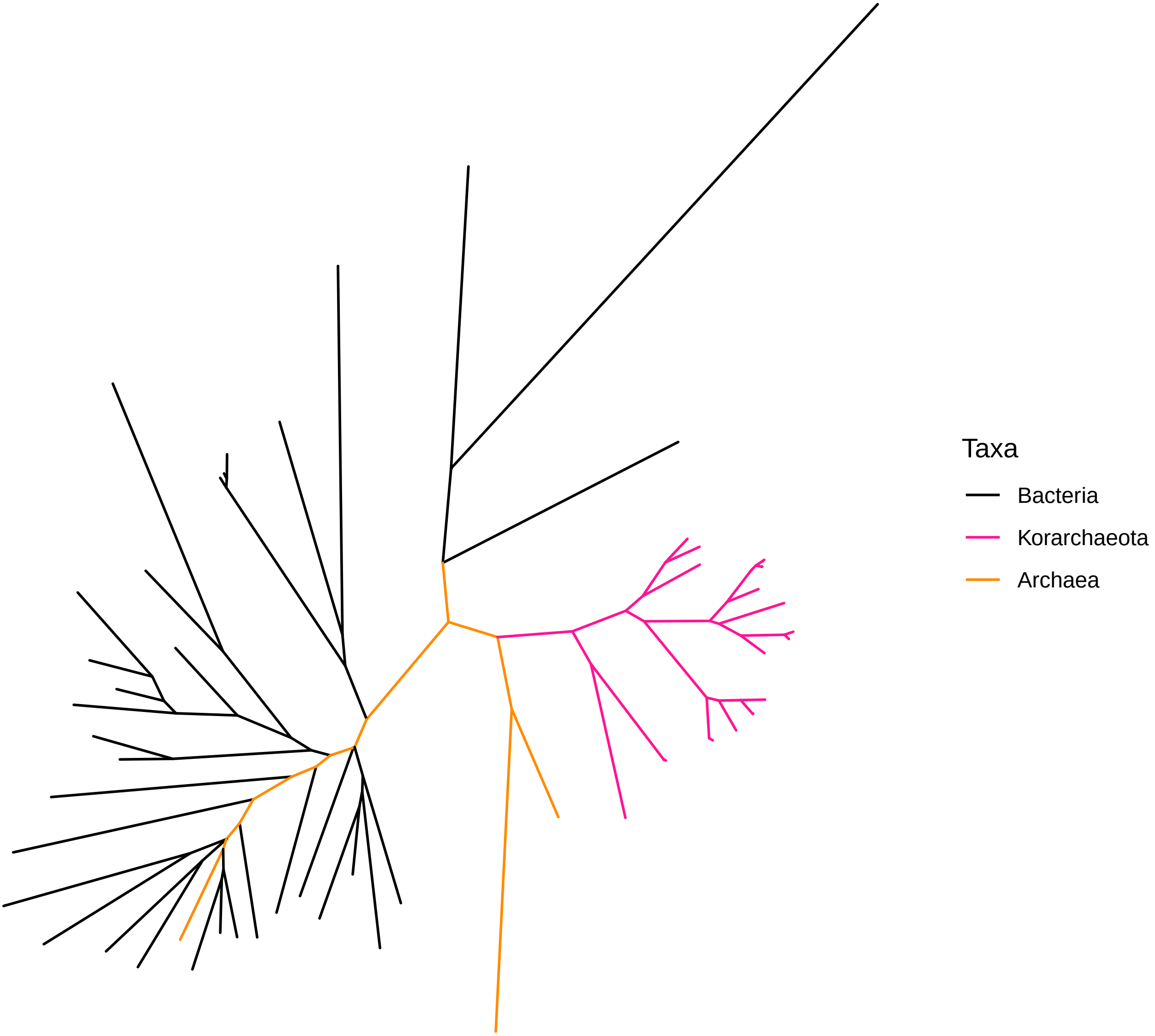

Kor−HOG0001256

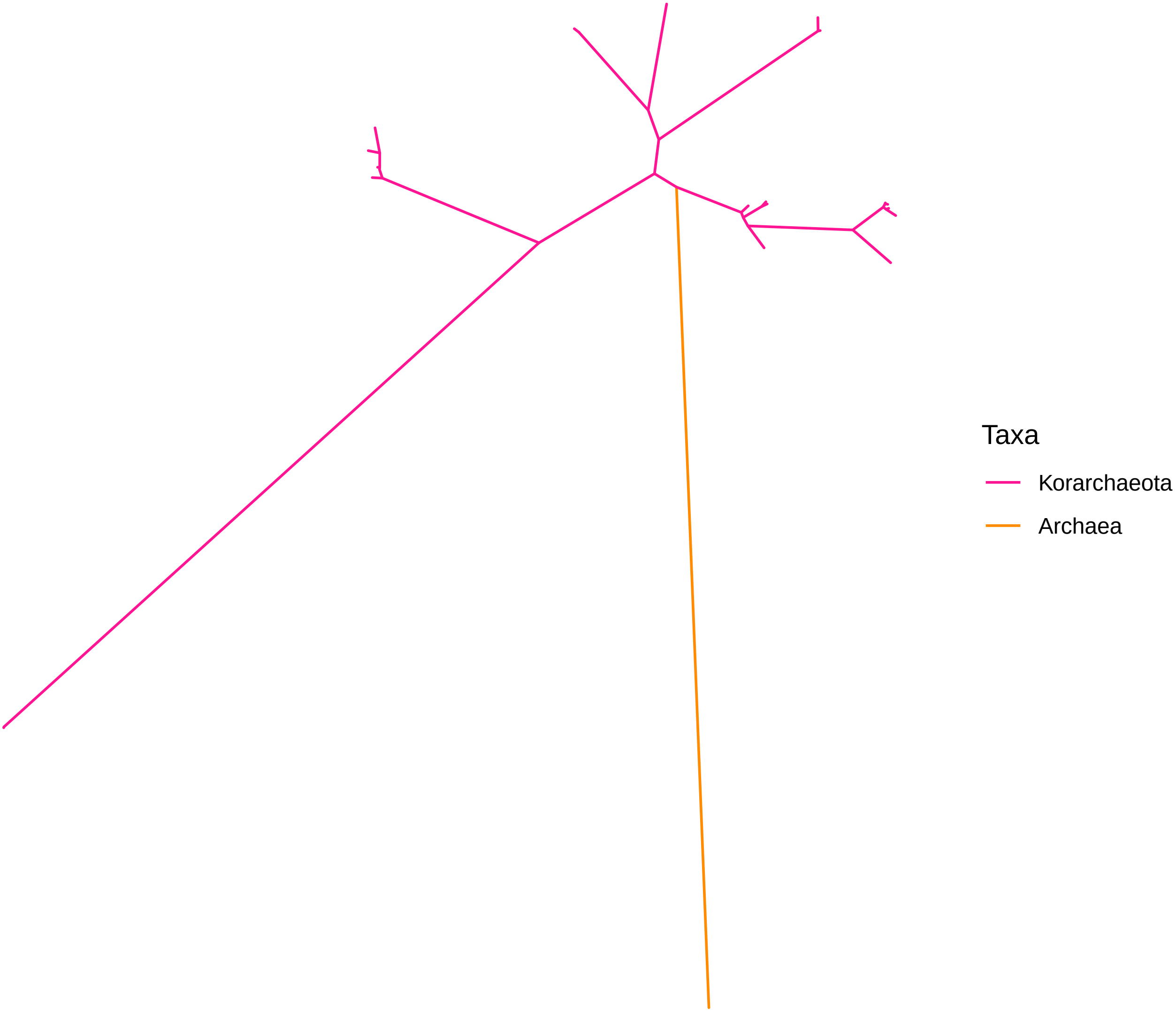

Kor−HOG0001259

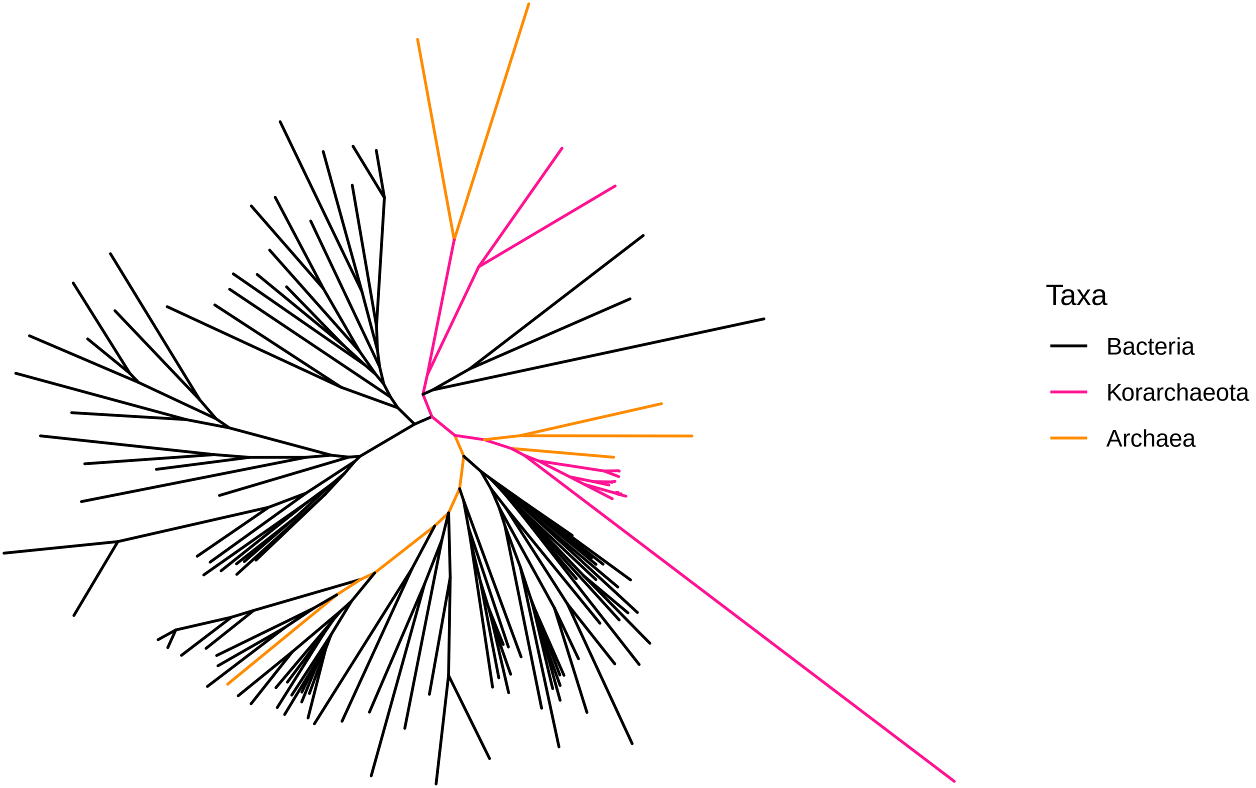

Kor−HOG0001429

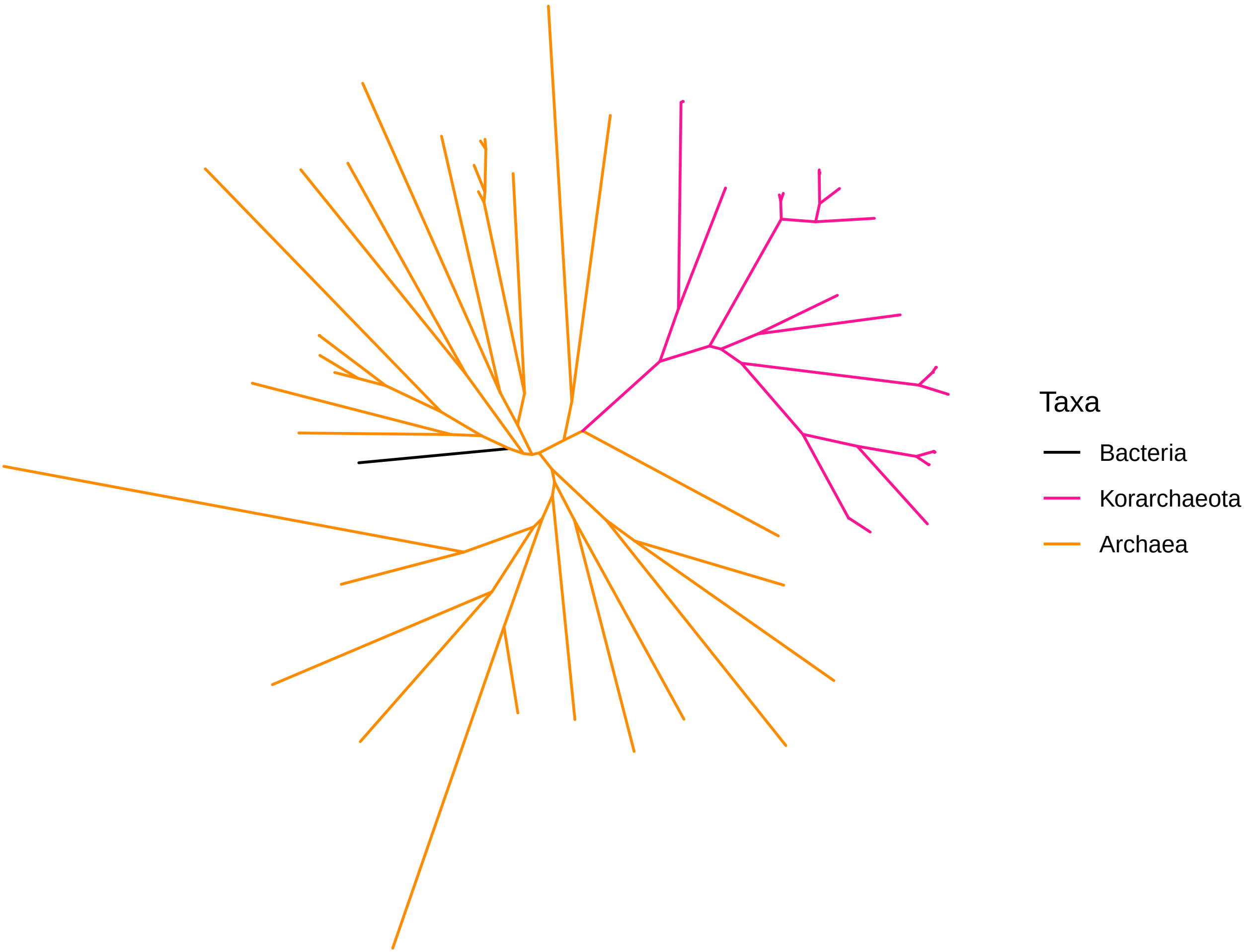

Supplement: Figure S3 — Phylogenetic trees constructed using 115 single-copy genes selected from among homologous genes in korarchaeal MAGs. [file msystems.00305-23-s0002.pdf]
