# Supplementary material for: Wood–Ljungdahl pathway found in novel marine Korarchaeota groups illuminates their evolutionary history
Source: mSystems. 2023 Jul 17;8(4):e00305-23. doi: 10.1128/msystems.00305-23 (PMC10469681; doi:10.1128/msystems.00305-23)
Supplement: Figure S13 — Phylogenetic trees based on 51 archaeal marker genes. [file msystems.00305-23-s0003.pdf]

arCOG01228

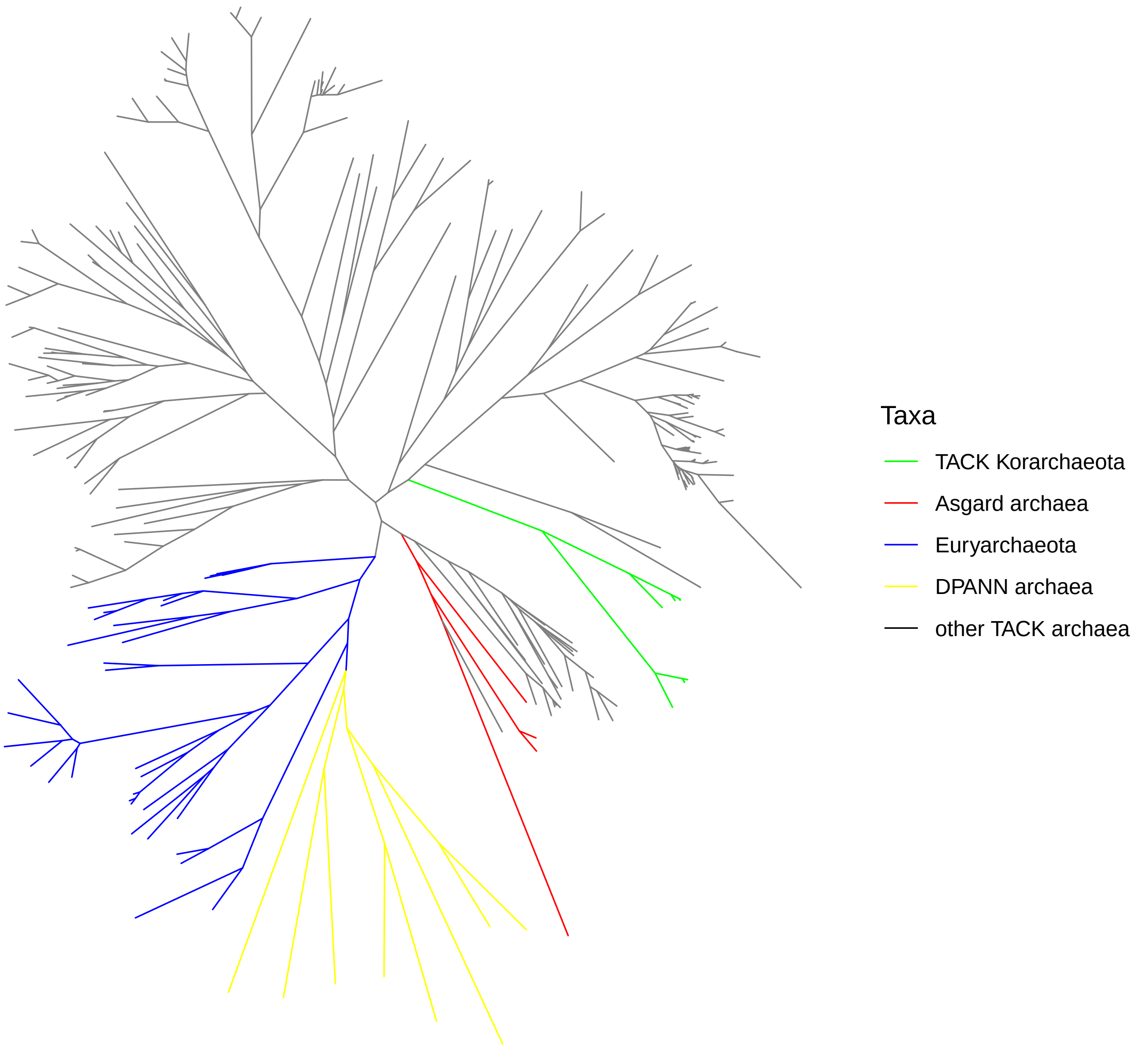
Taxa
TACK Korarchaeota
Asgard archaea
Euryarchaeota
DPANN archaea
other TACK archaea

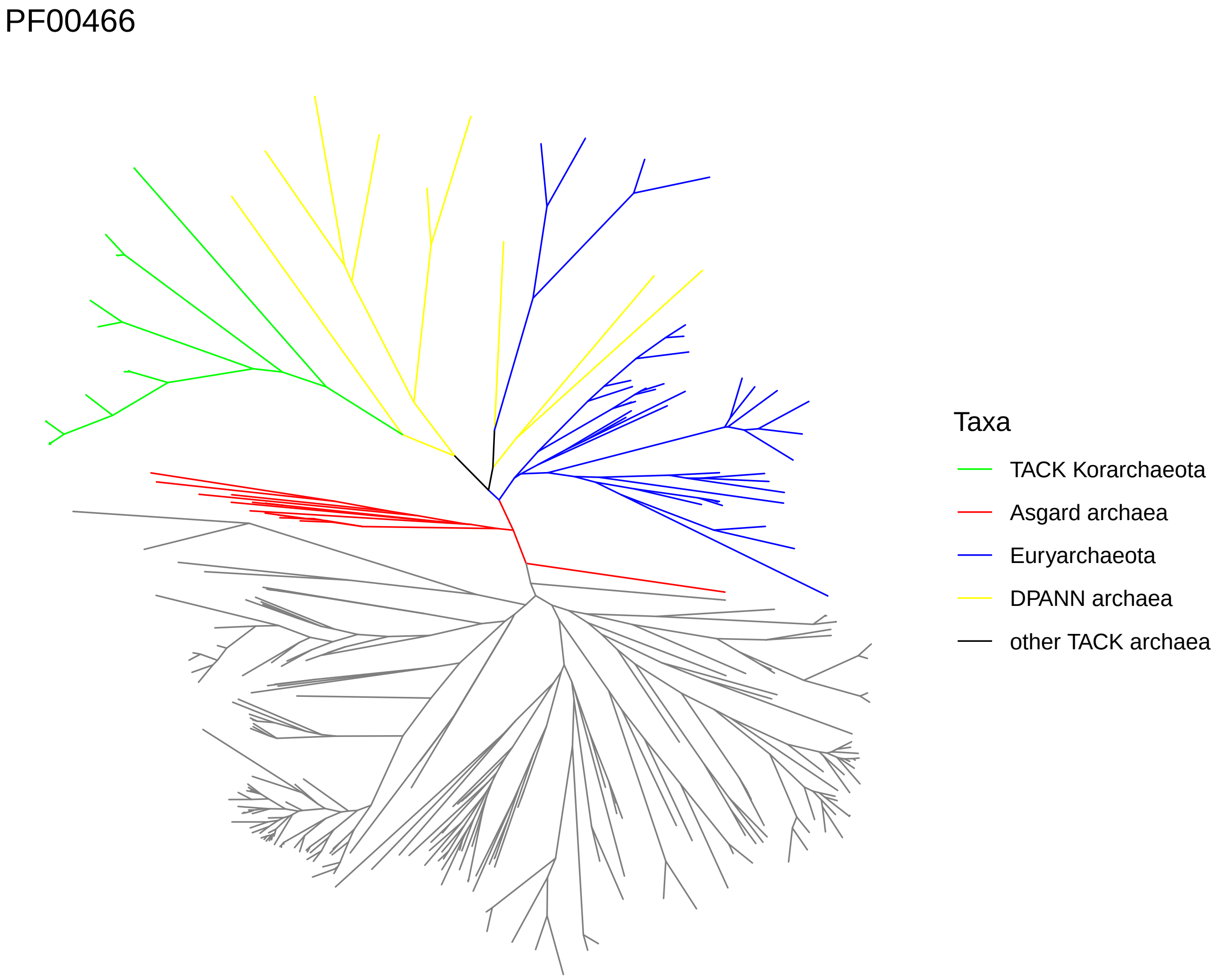

PF00466
Taxa
TACK Korarchaeota
Asgard archaea
Euryarchaeota
DPANN archaea
other TACK archaea

PF00687

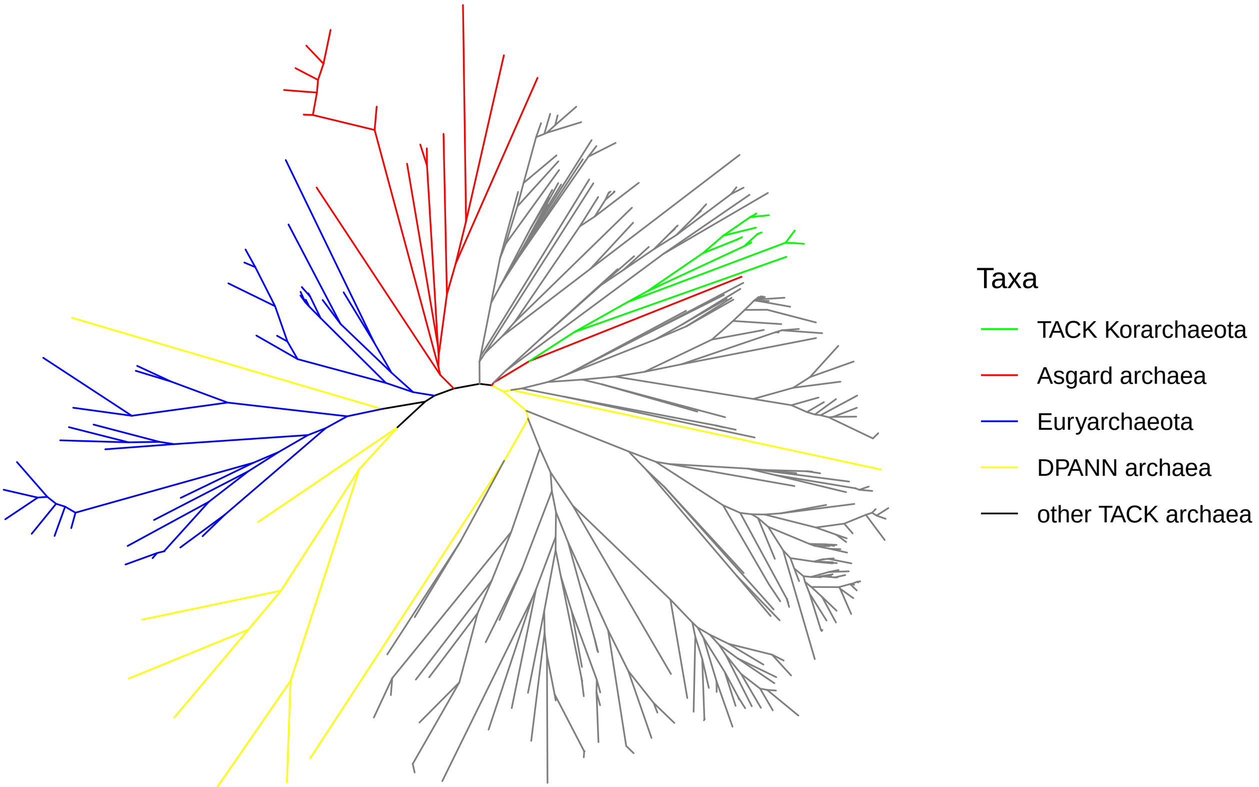

Taxa
TACK Korarchaeota
Asgard archaea
Euryarchaeota
DPANN archaea
other TACK archaea

PF00827

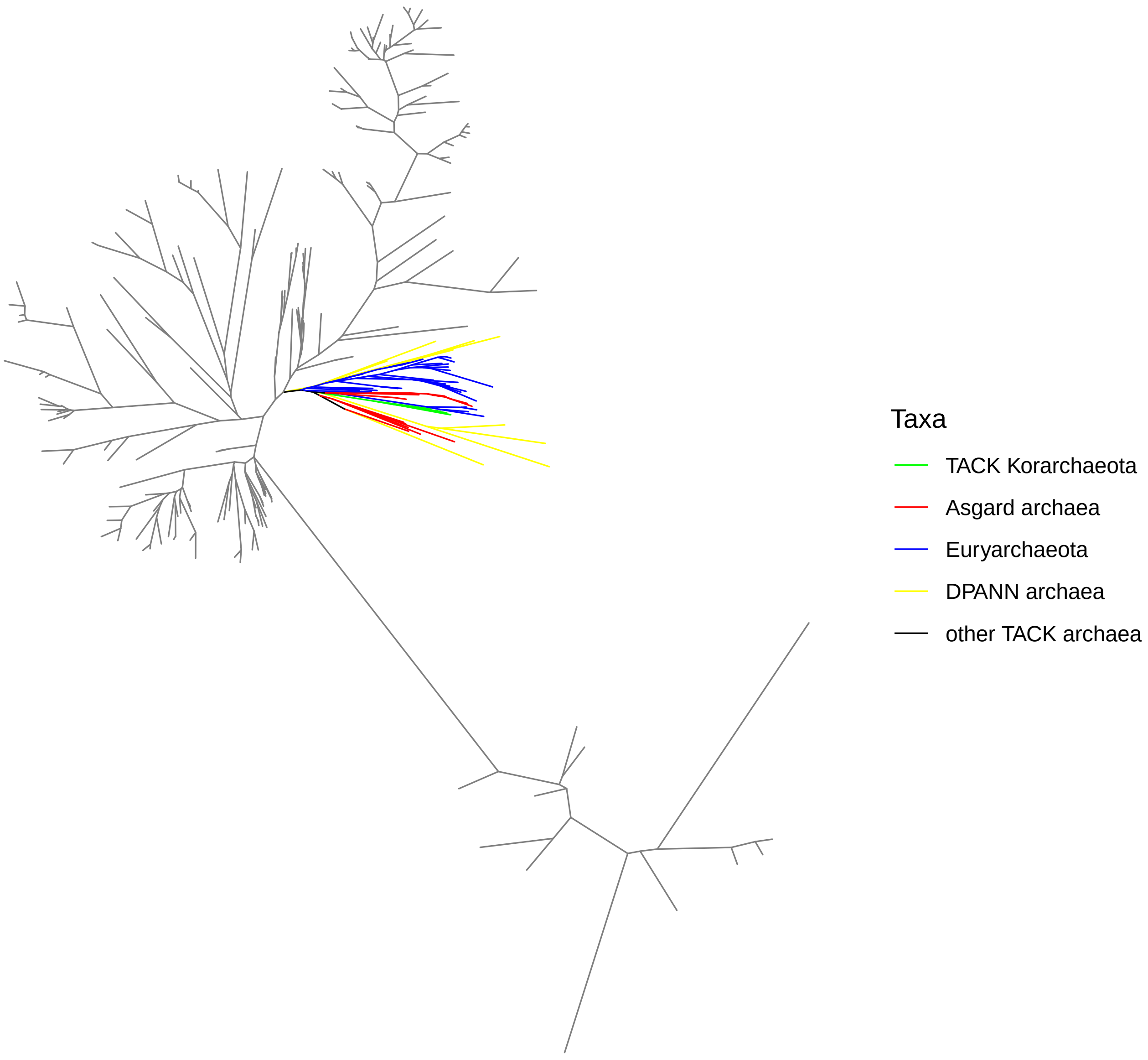
Taxa
TACK Korarchaeota
Asgard archaea
Euryarchaeota
DPANN archaea
other TACK archaea

PF00900

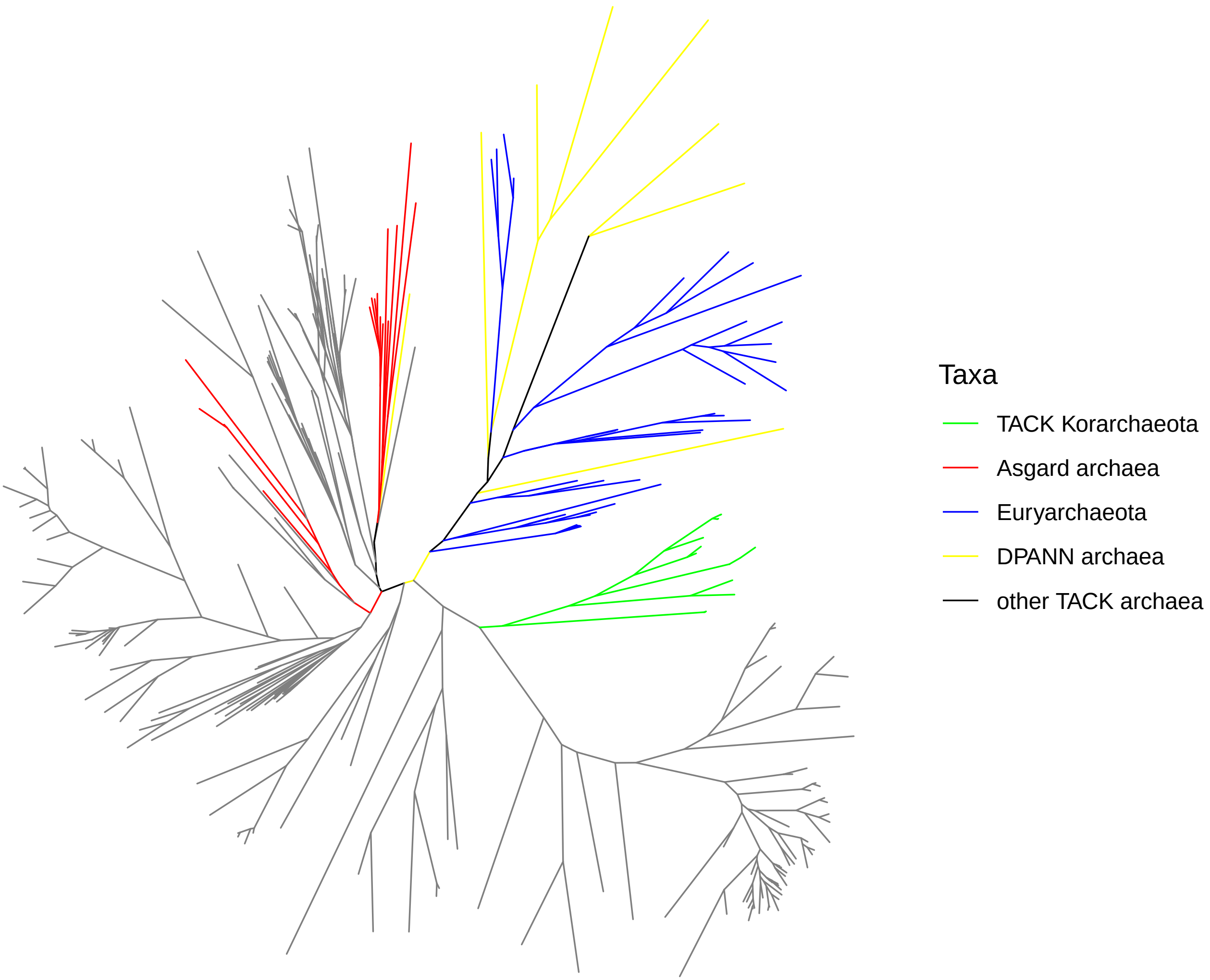
Taxa
TACK Korarchaeota
Asgard archaea
Euryarchaeota
DPANN archaea
other TACK archaea

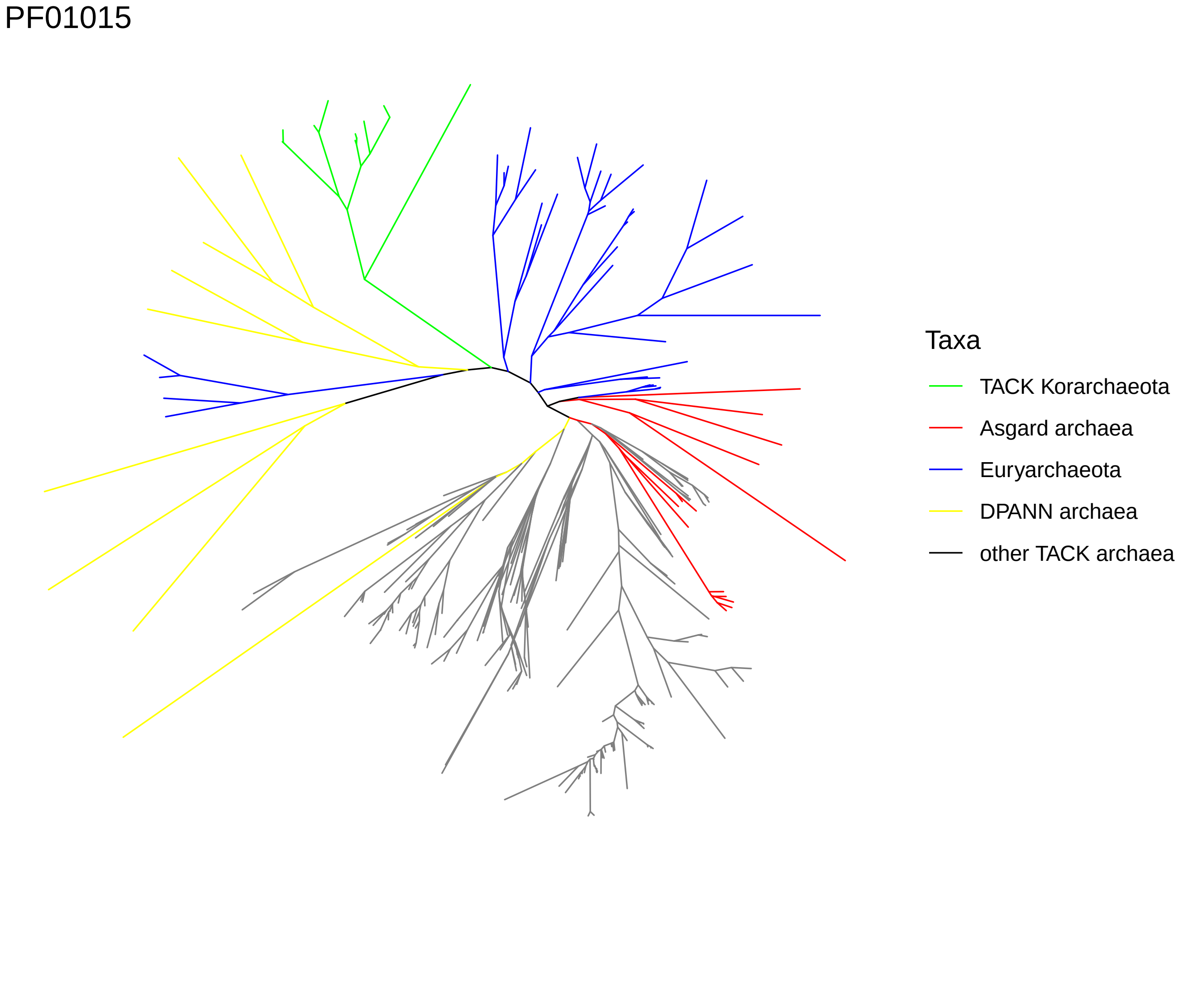
PF01015
Taxa
TACK Korarchaeota
Asgard archaea
Euryarchaeota
DPANN archaea
other TACK archaea

PF01092

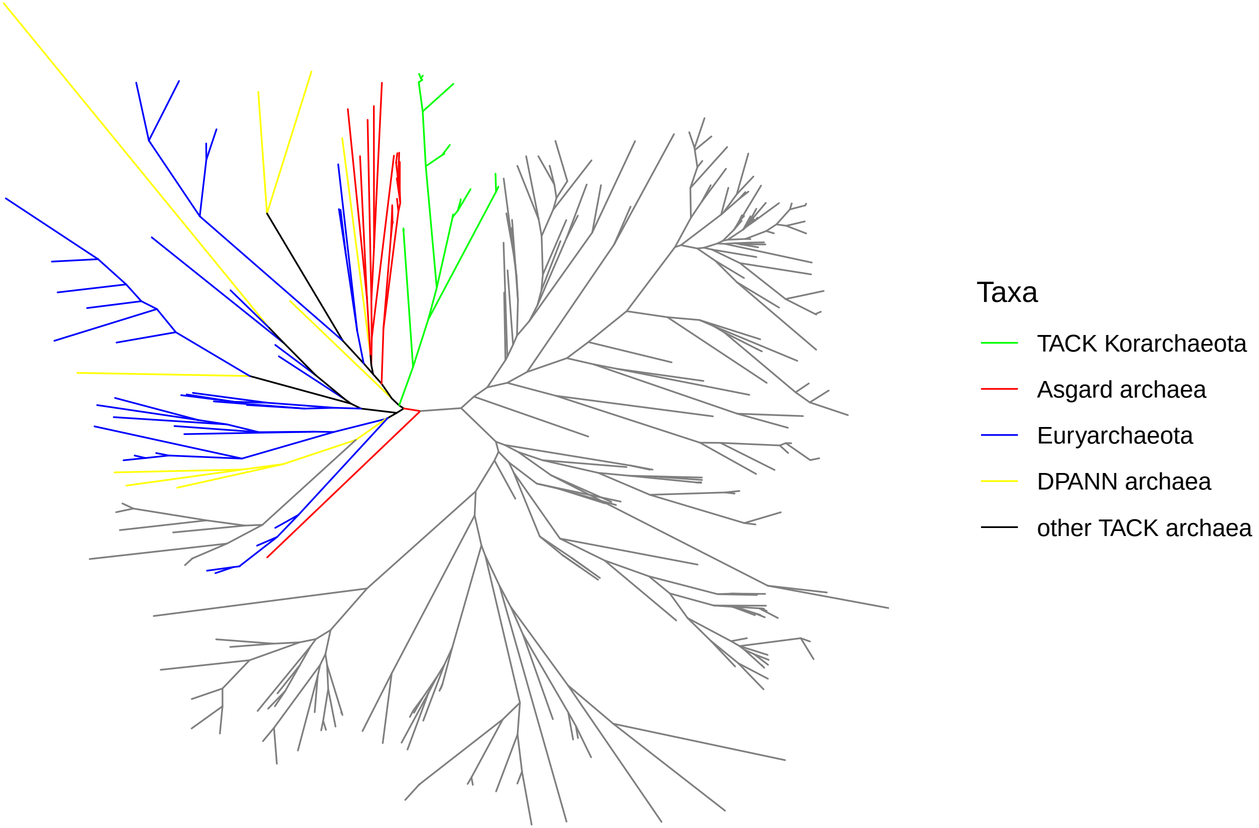
Taxa
TACK Korarchaeota
Asgard archaea
Euryarchaeota
DPANN archaea
other TACK archaea

PF01200

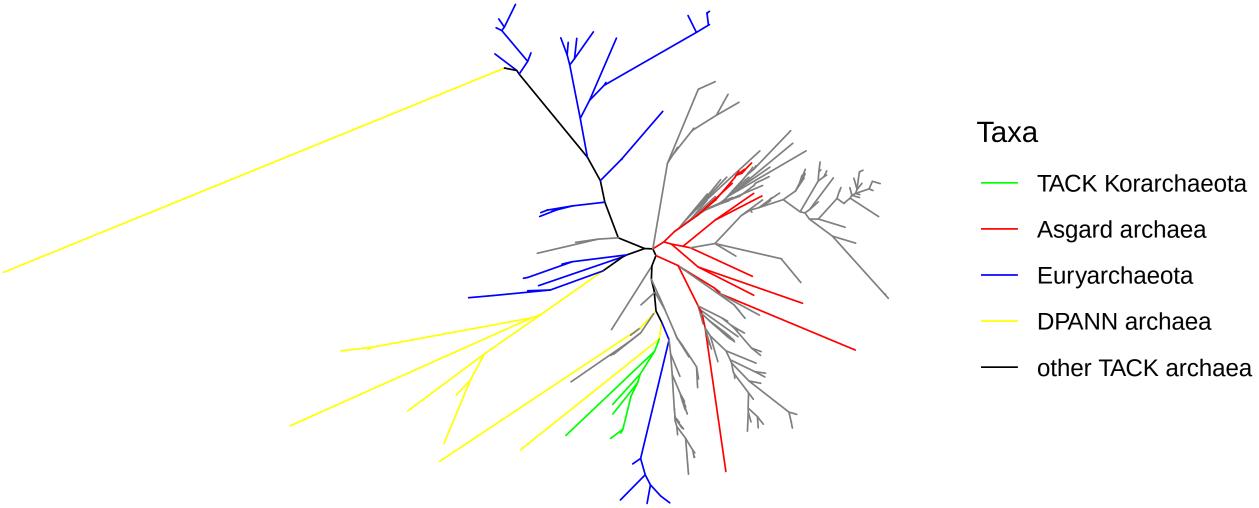

Taxa
TACK Korarchaeota
Asgard archaea
Euryarchaeota
DPANN archaea
other TACK archaea

PF01280

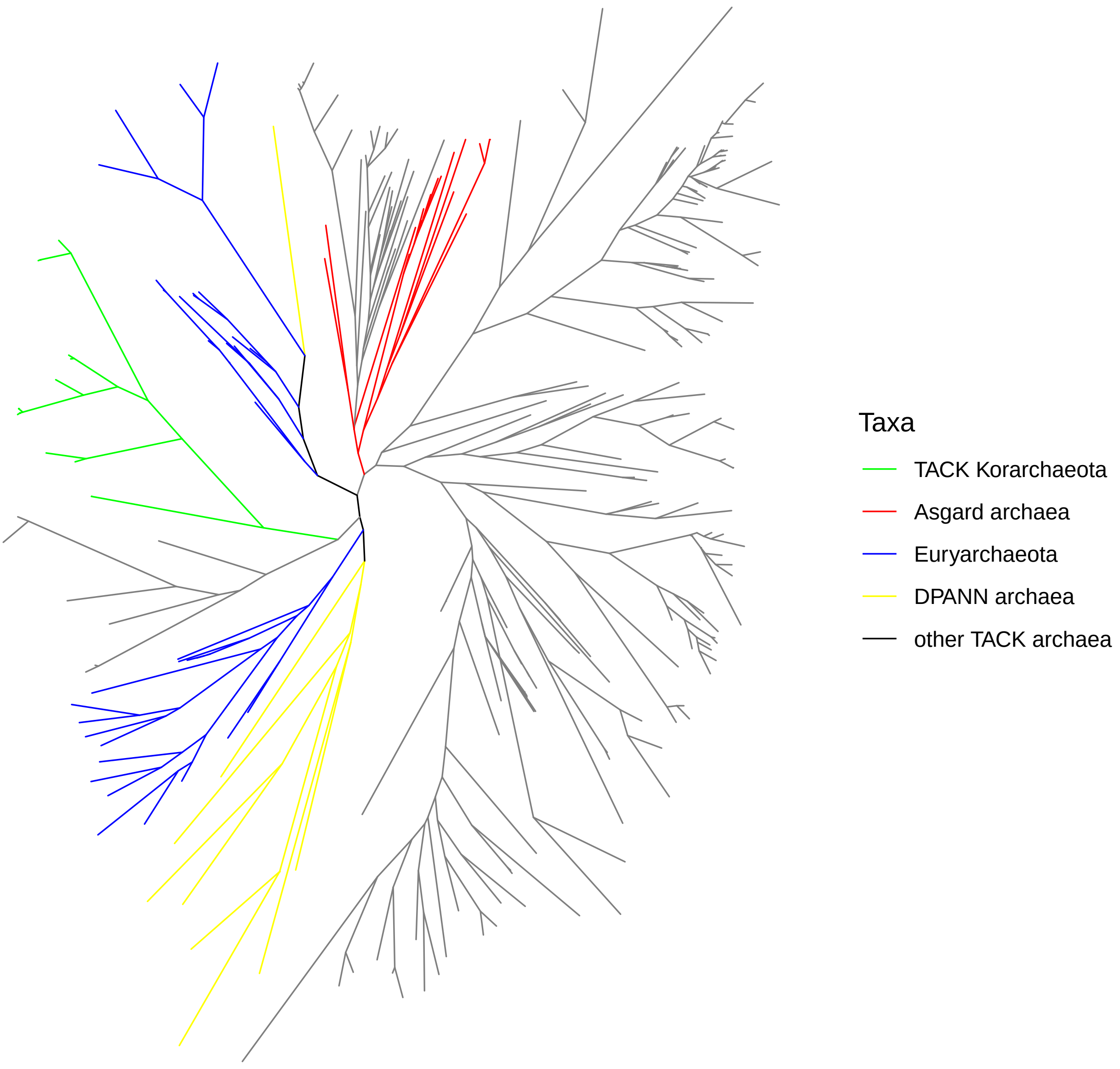
Taxa
TACK Korarchaeota
Asgard archaea
Euryarchaeota
DPANN archaea
other TACK archaea

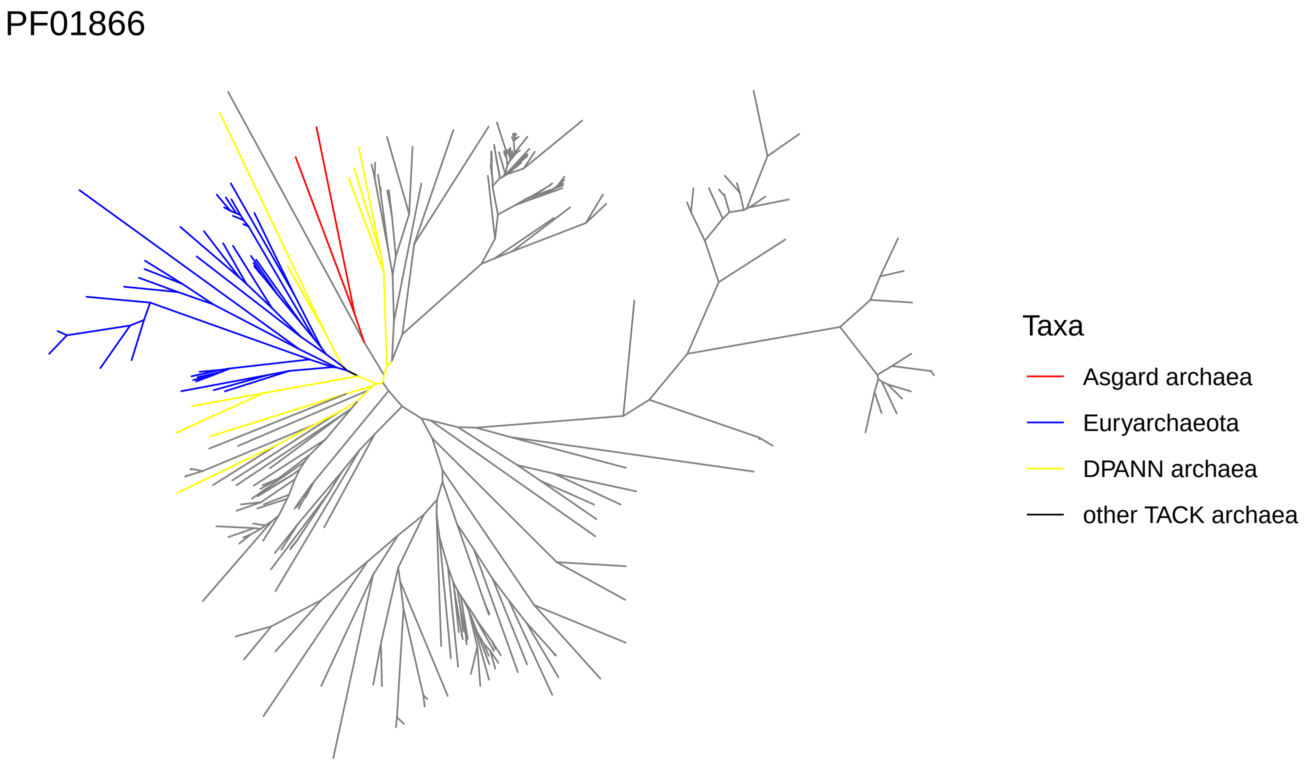
PF01866
Taxa
Asgard archaea
Euryarchaeota
DPANN archaea
other TACK archaea

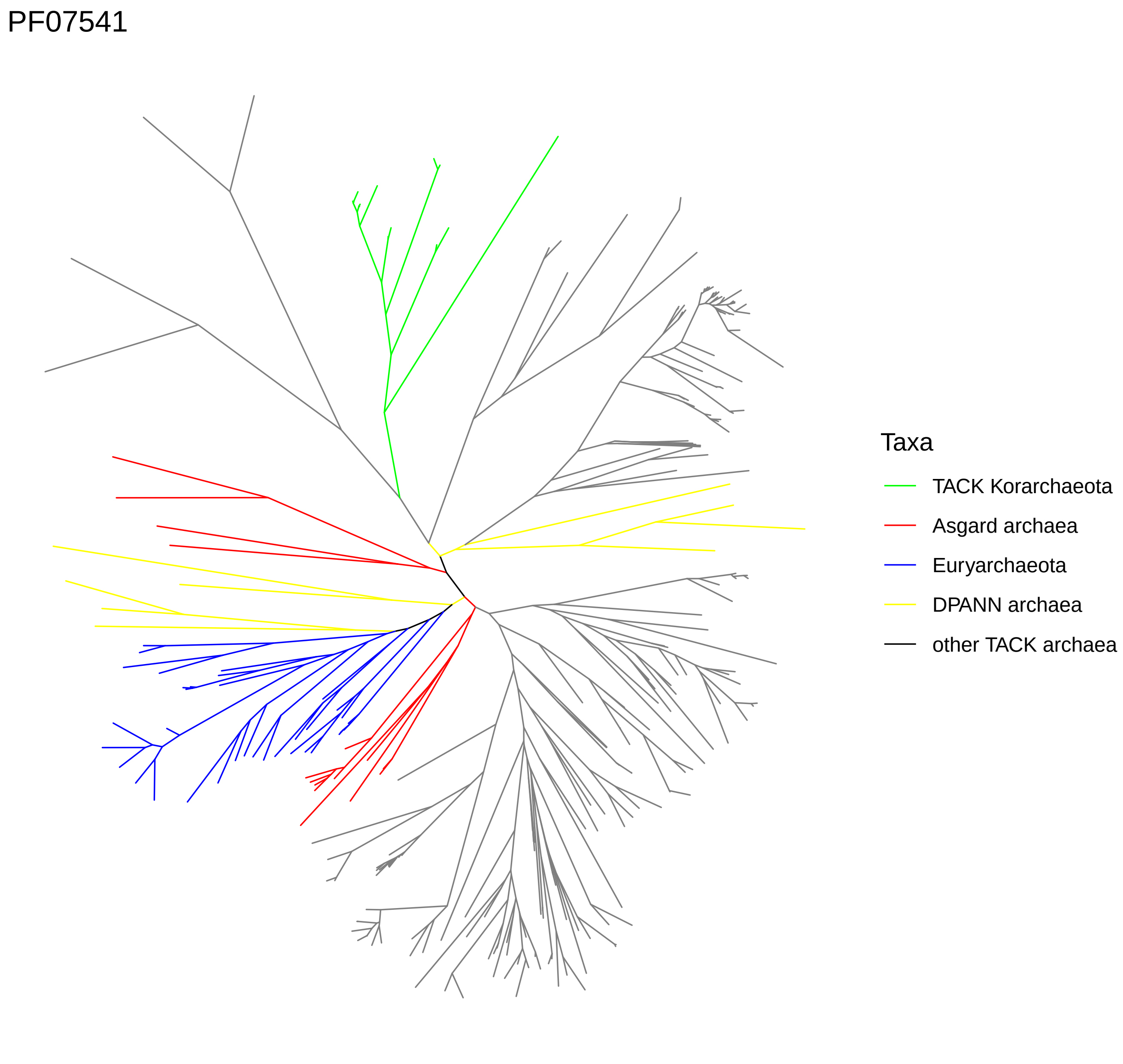
PF07541
Taxa
TACK Korarchaeota
Asgard archaea
Euryarchaeota
DPANN archaea
other TACK archaea

PF13656

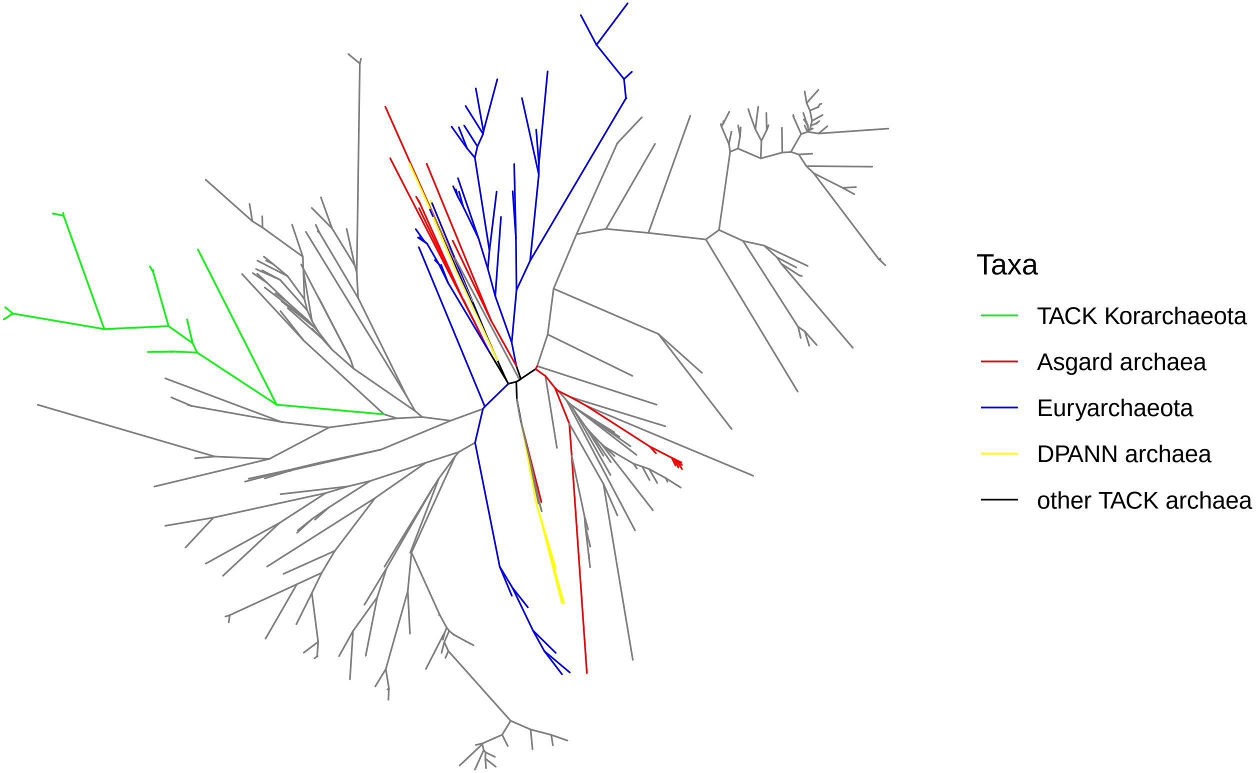
Taxa
TACK Korarchaeota
Asgard archaea
Euryarchaeota
DPANN archaea
other TACK archaea

TIGR00037

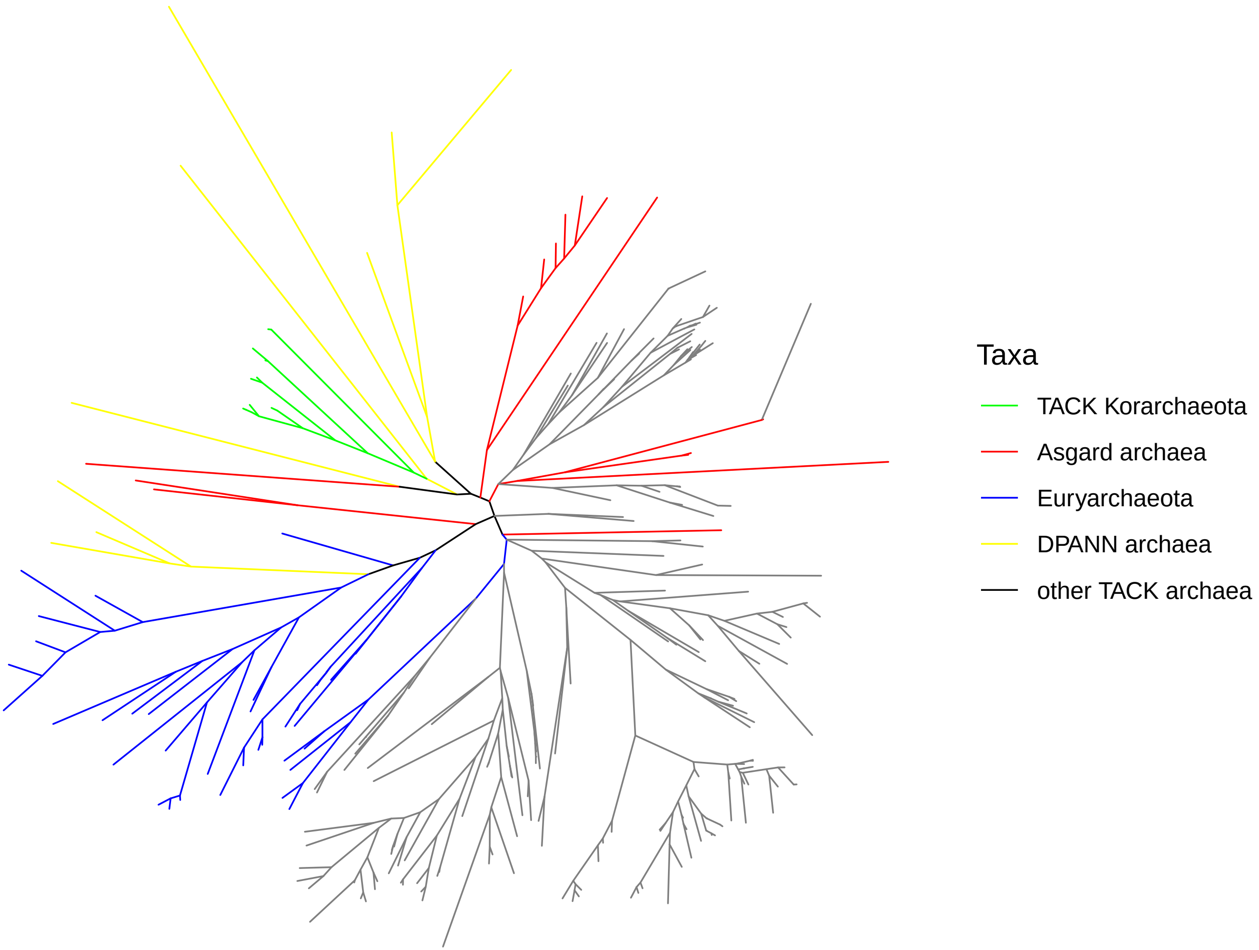

Taxa
TACK Korarchaeota
Asgard archaea
Euryarchaeota
DPANN archaea
other TACK archaea

TIGR00064

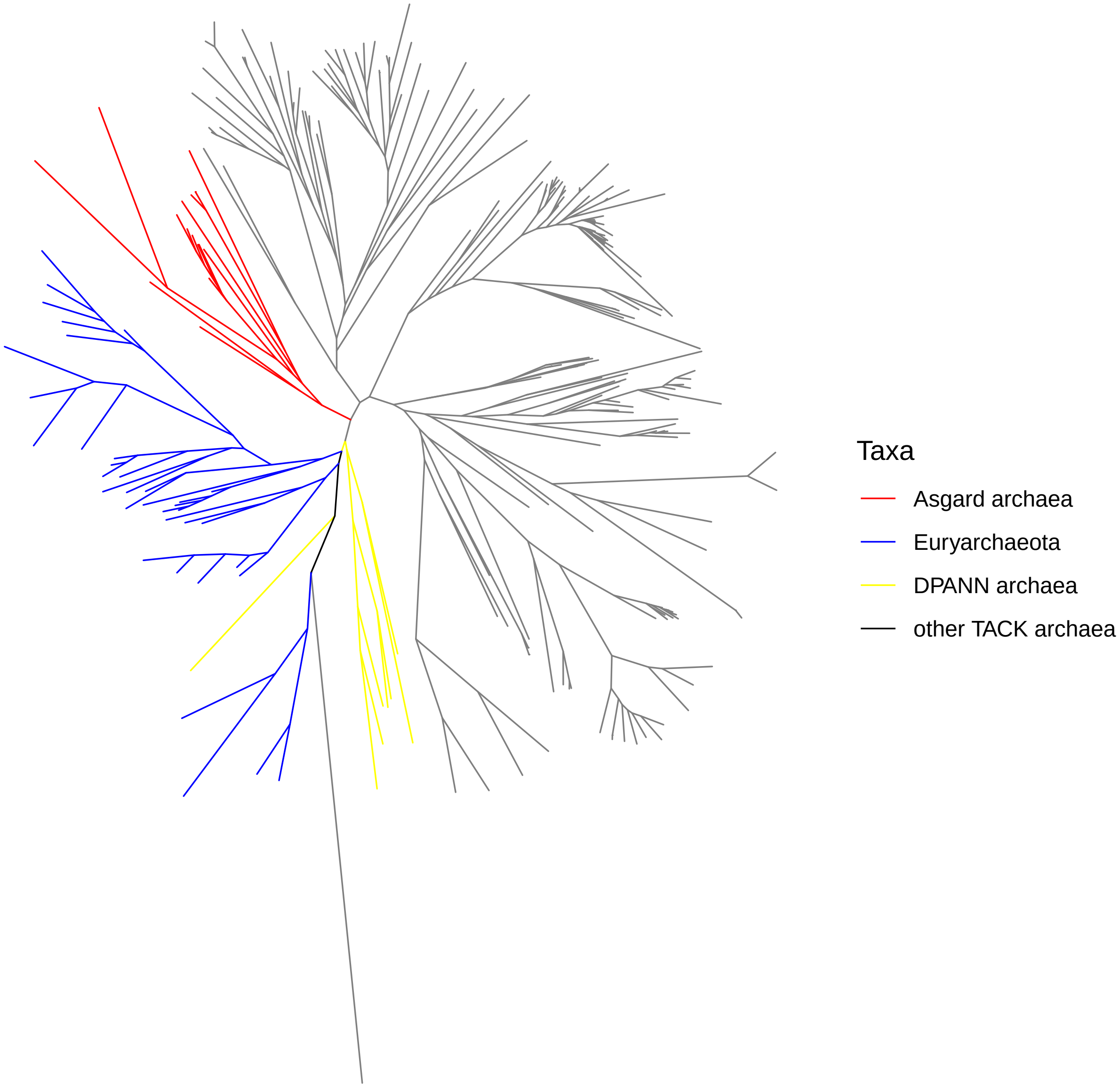

Taxa
Asgard archaea
Euryarchaeota
DPANN archaea
other TACK archaea

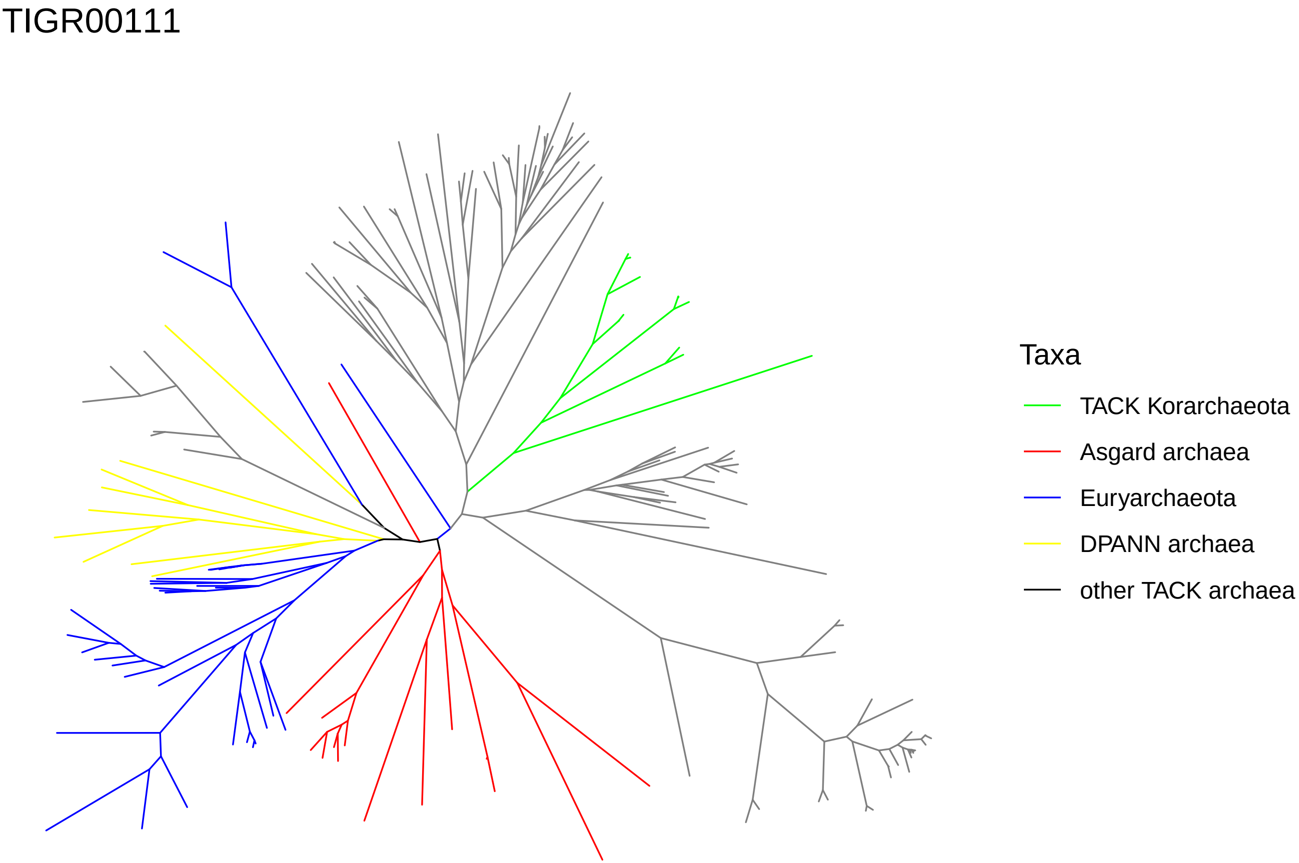

TIGR00111
Taxa
TACK Korarchaeota
Asgard archaea
Euryarchaeota
DPANN archaea
other TACK archaea

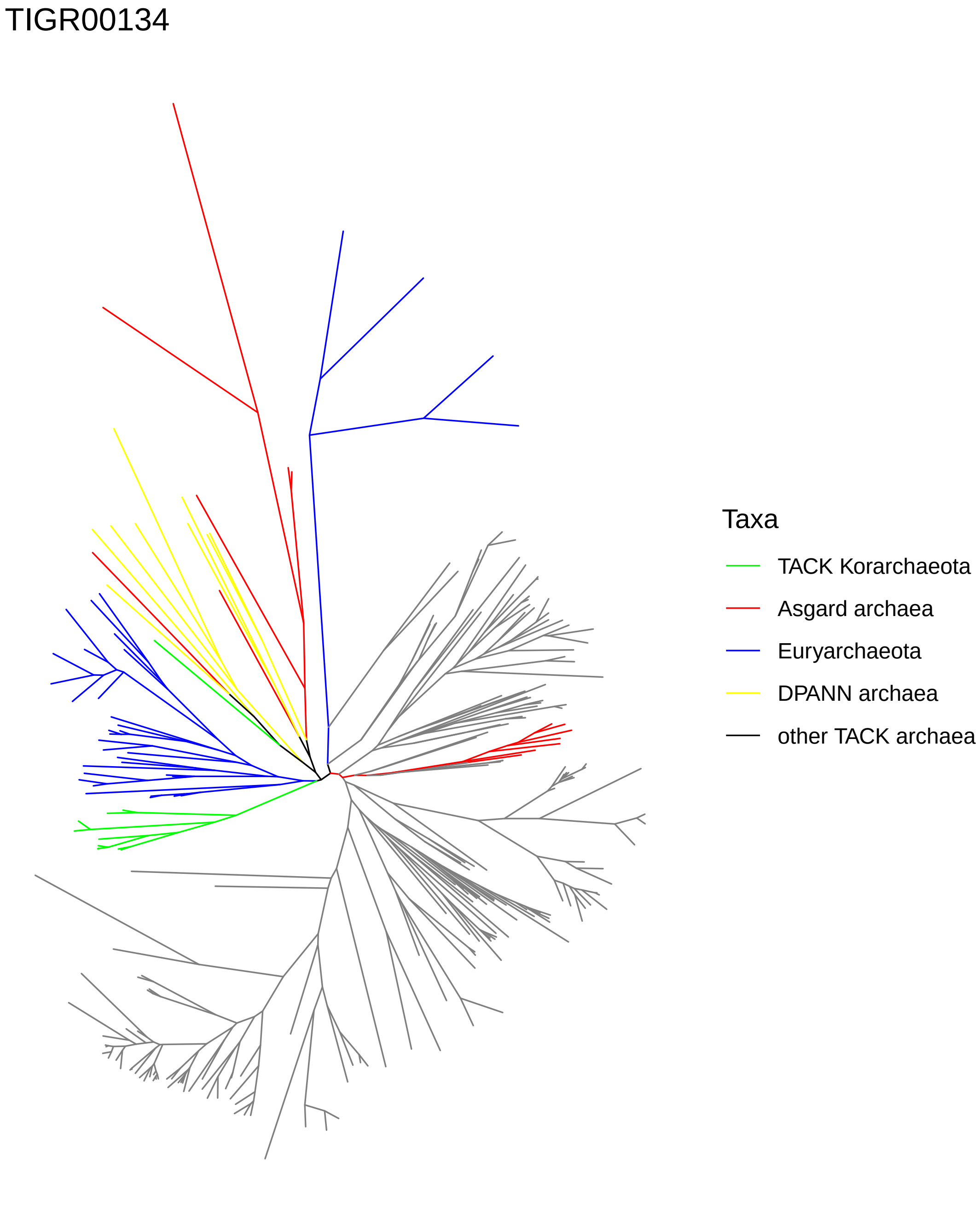
TIGR00134
Taxa
TACK Korarchaeota
Asgard archaea
Euryarchaeota
DPANN archaea
other TACK archaea

TIGR00291

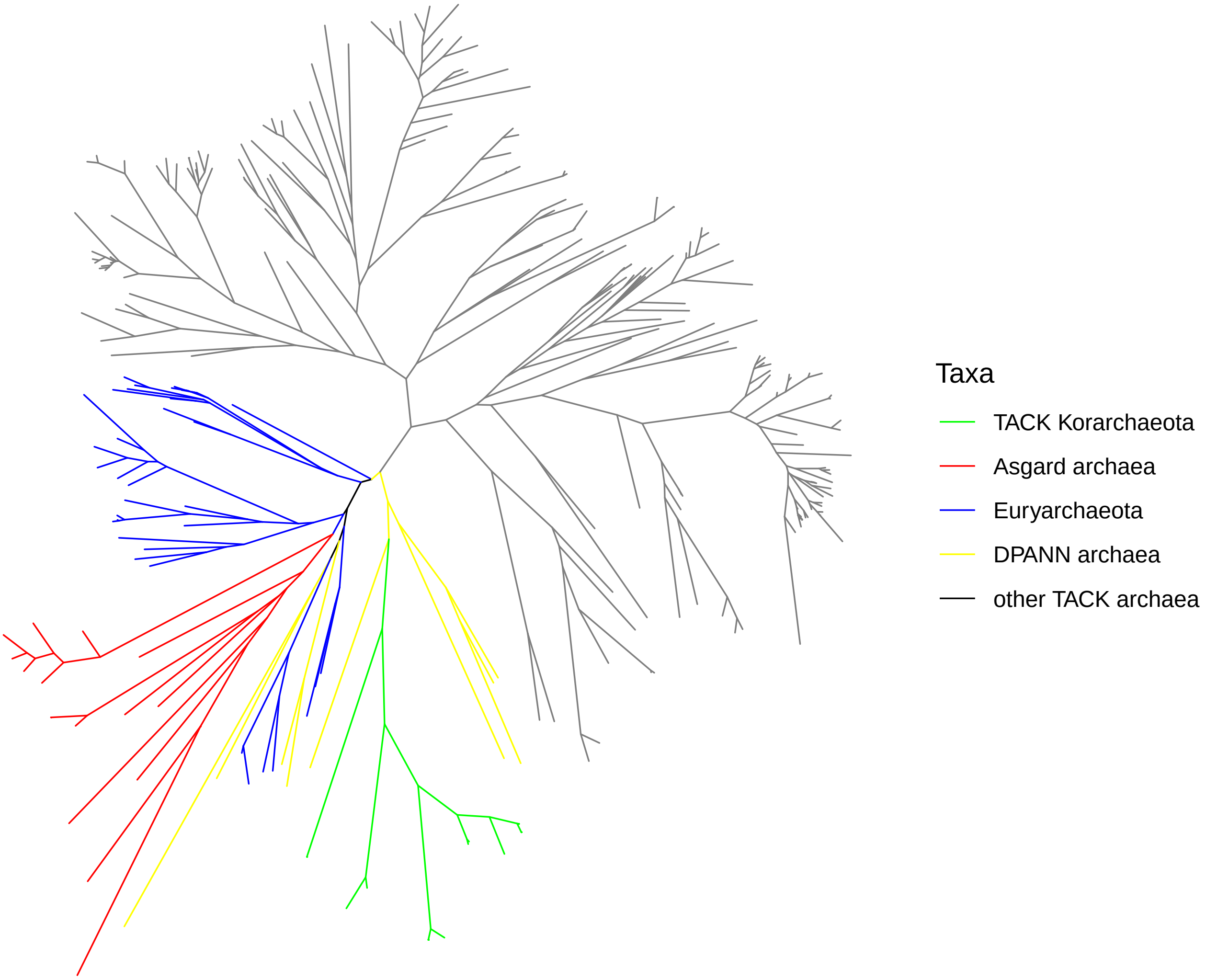

Taxa
TACK Korarchaeota
Asgard archaea
Euryarchaeota
DPANN archaea
other TACK archaea

TIGR00405

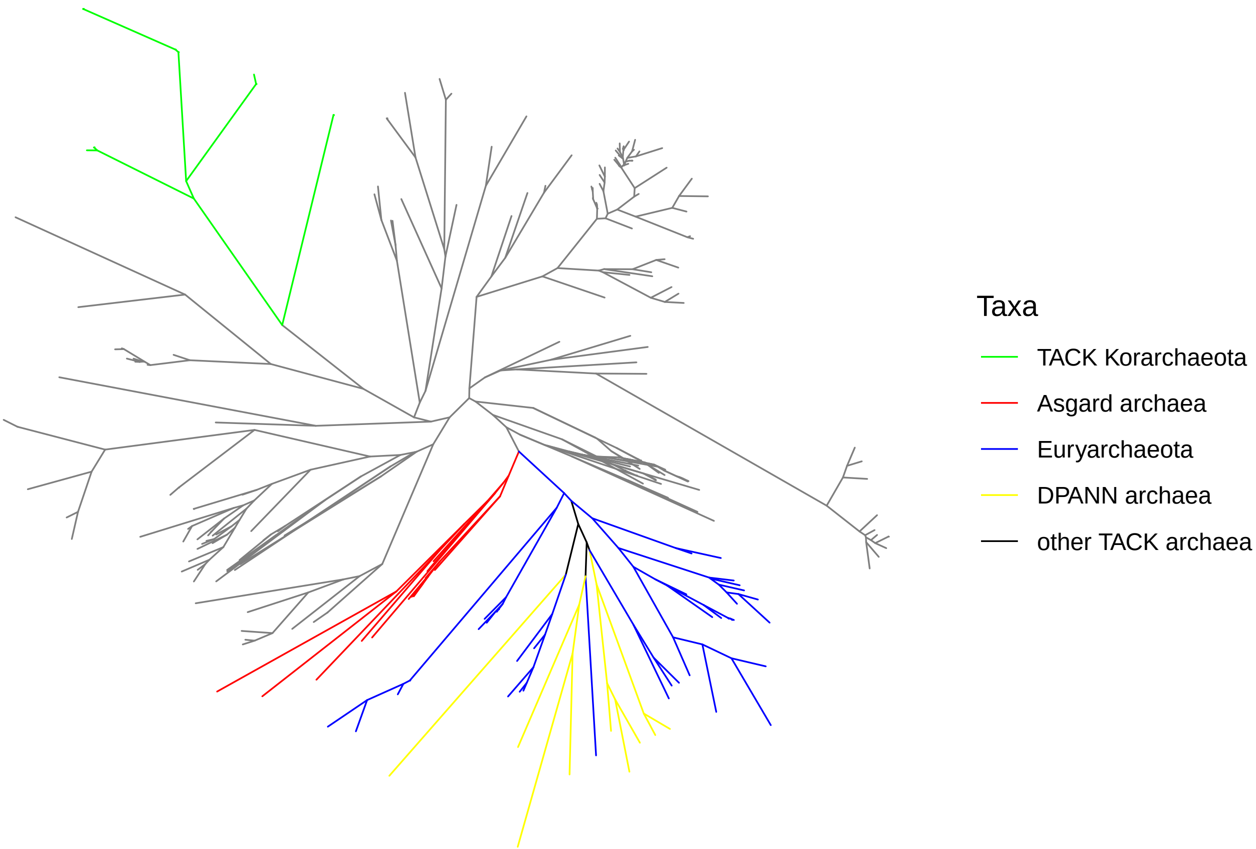

Taxa
TACK Korarchaeota
Asgard archaea
Euryarchaeota
DPANN archaea
other TACK archaea

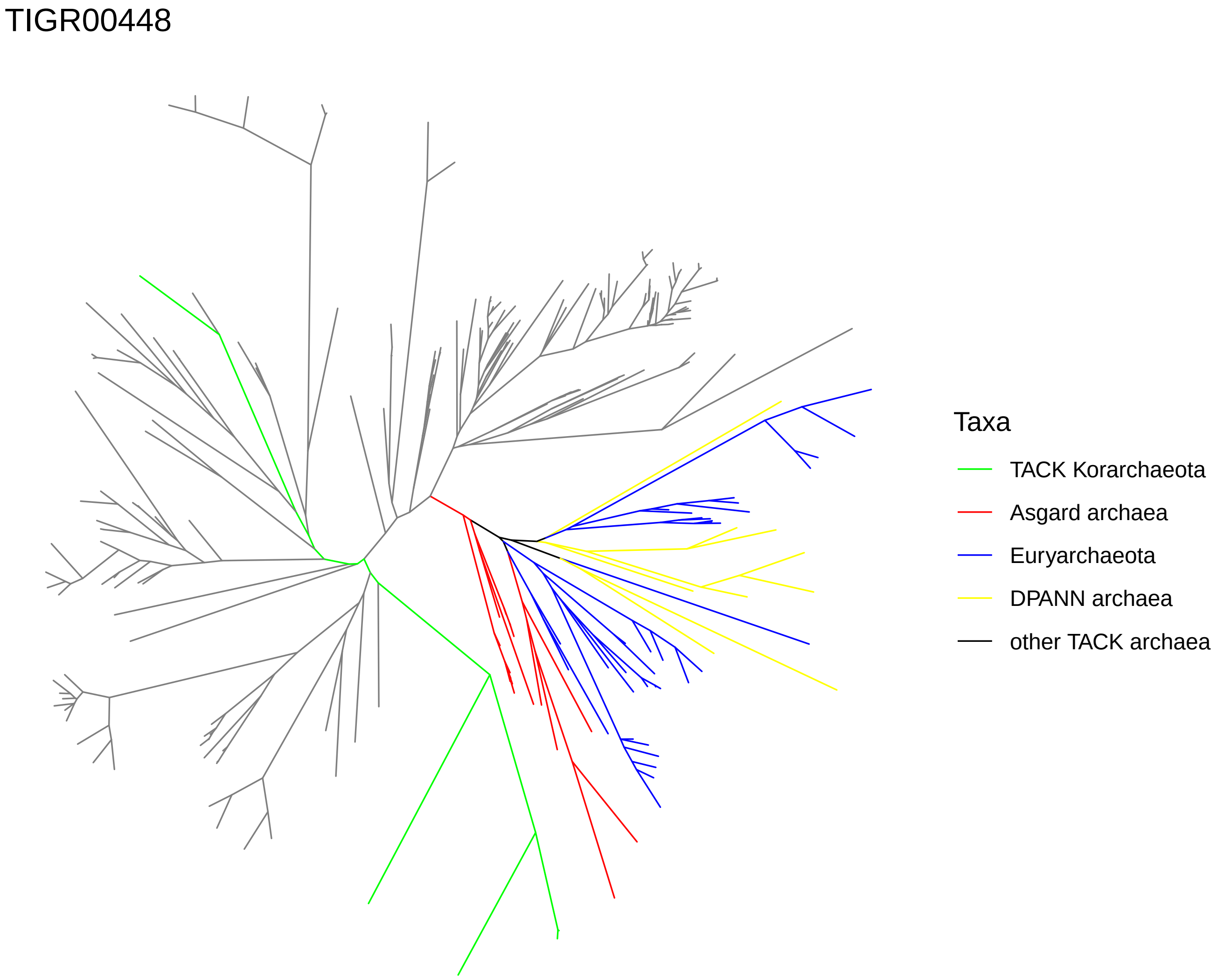
TIGR00448
Taxa
TACK Korarchaeota
Asgard archaea
Euryarchaeota
DPANN archaea
other TACK archaea

TIGR00483

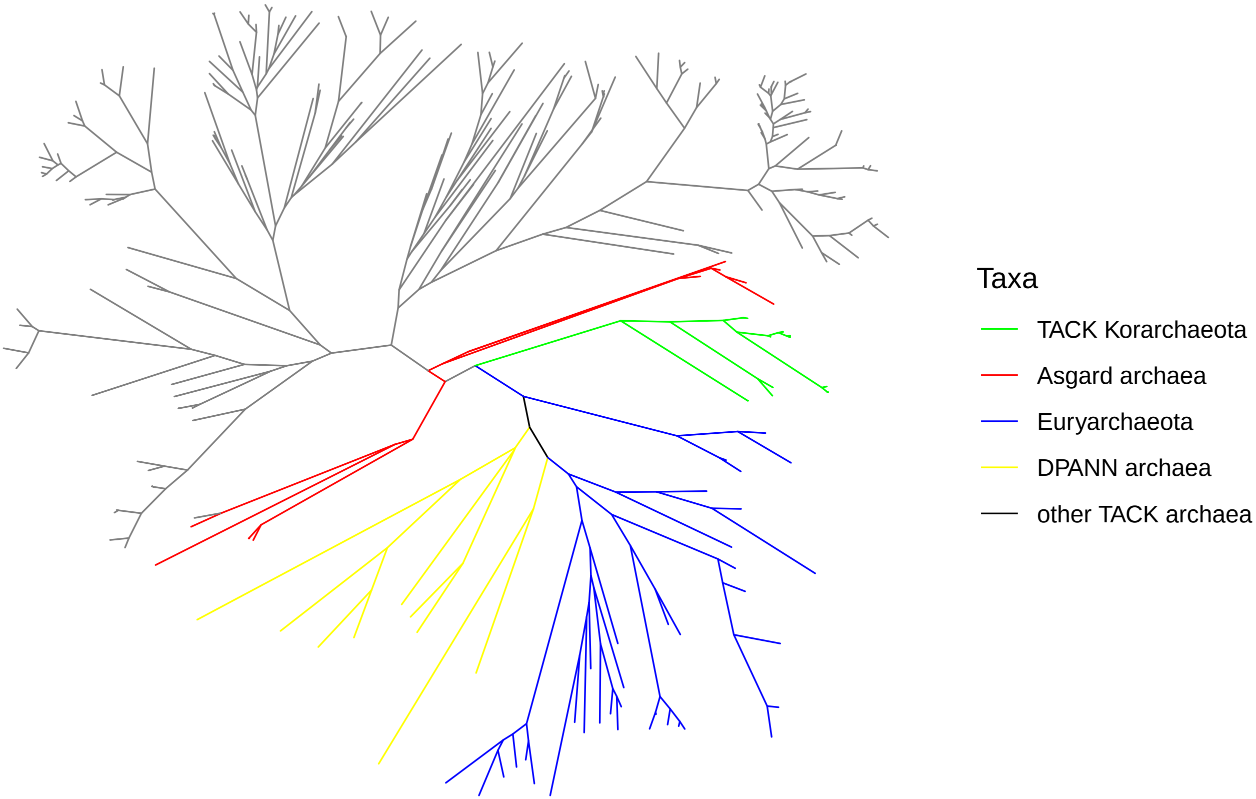

Taxa
TACK Korarchaeota
Asgard archaea
Euryarchaeota
DPANN archaea
other TACK archaea

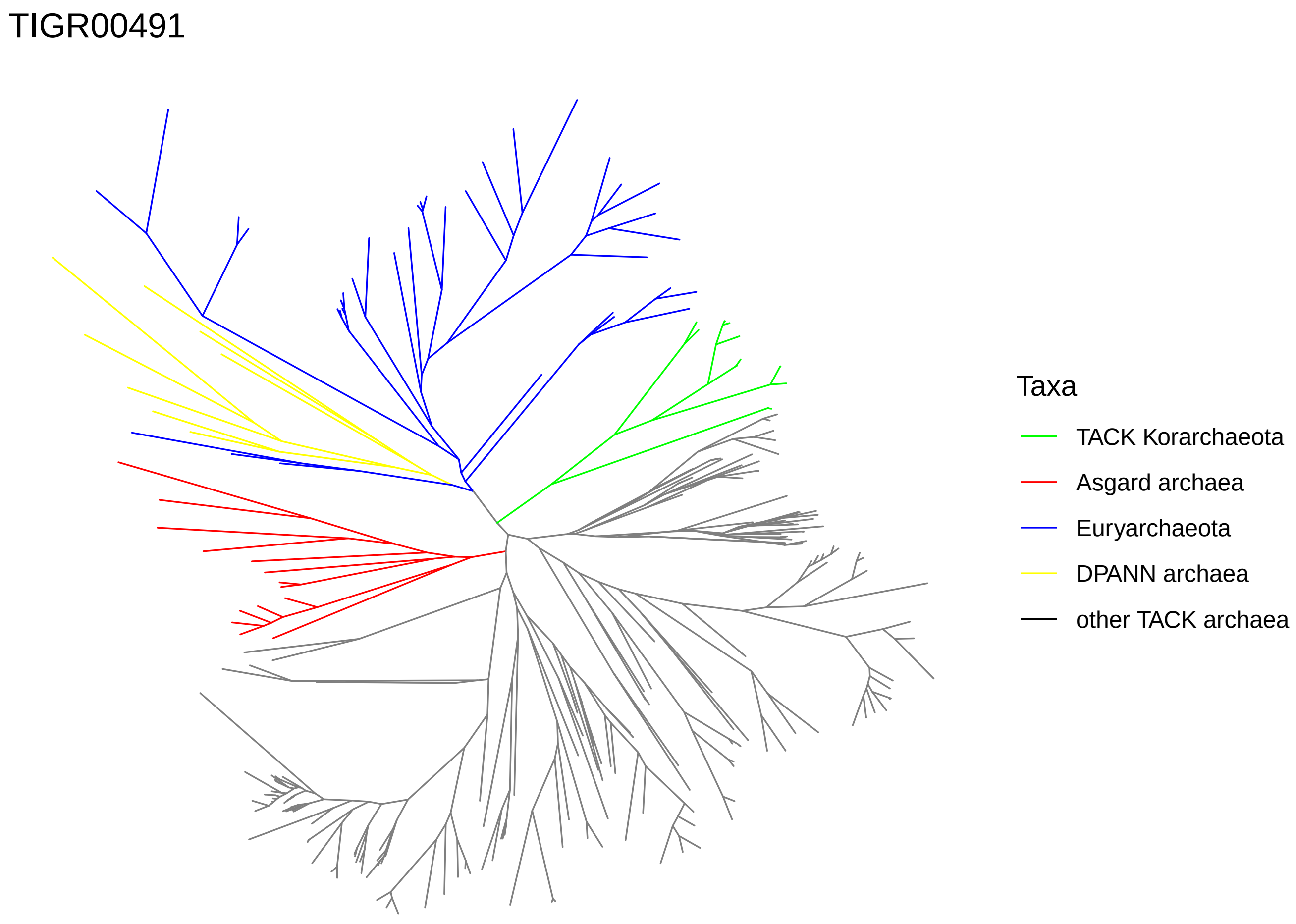

TIGR00491
Taxa
TACK Korarchaeota
Asgard archaea
Euryarchaeota
DPANN archaea
other TACK archaea

TIGR00967

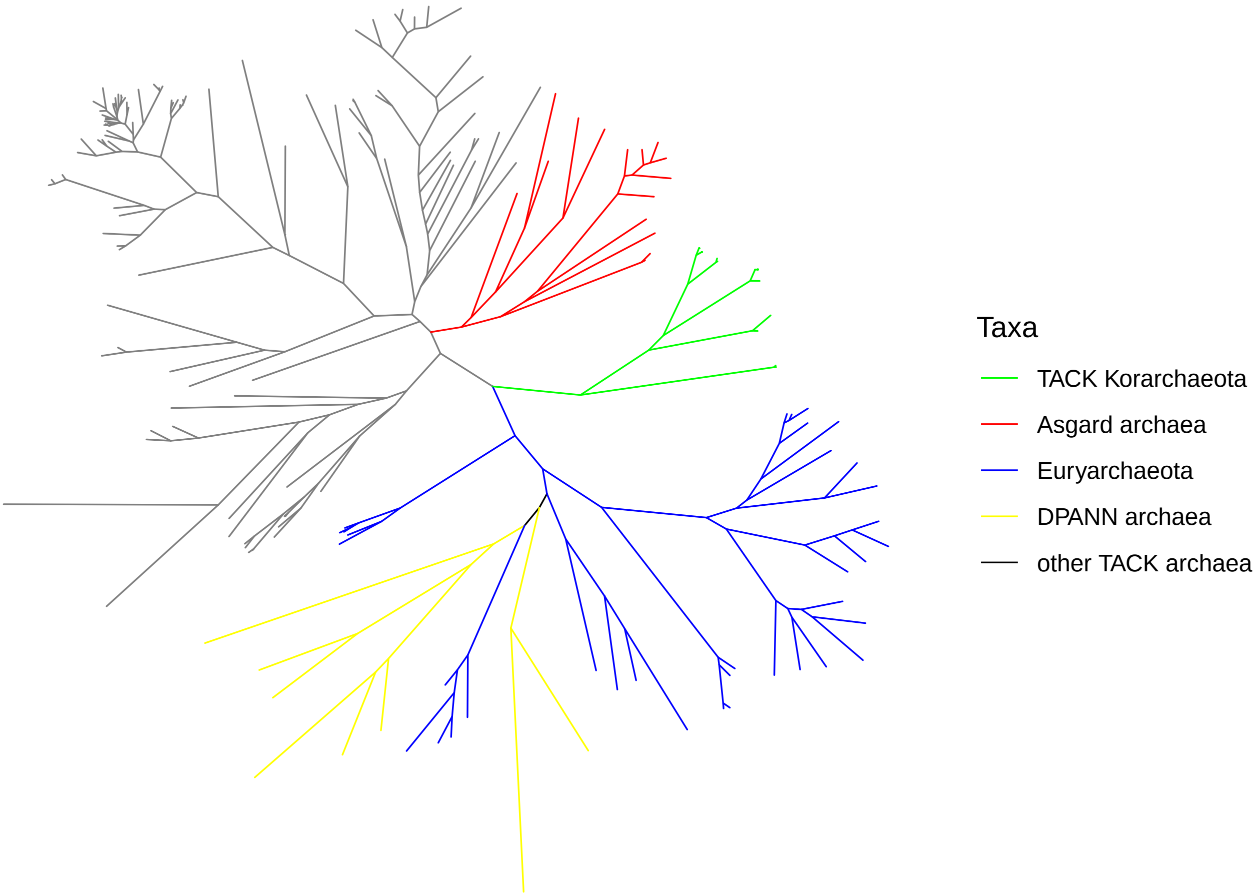

Taxa
TACK Korarchaeota
Asgard archaea
Euryarchaeota
DPANN archaea
other TACK archaea

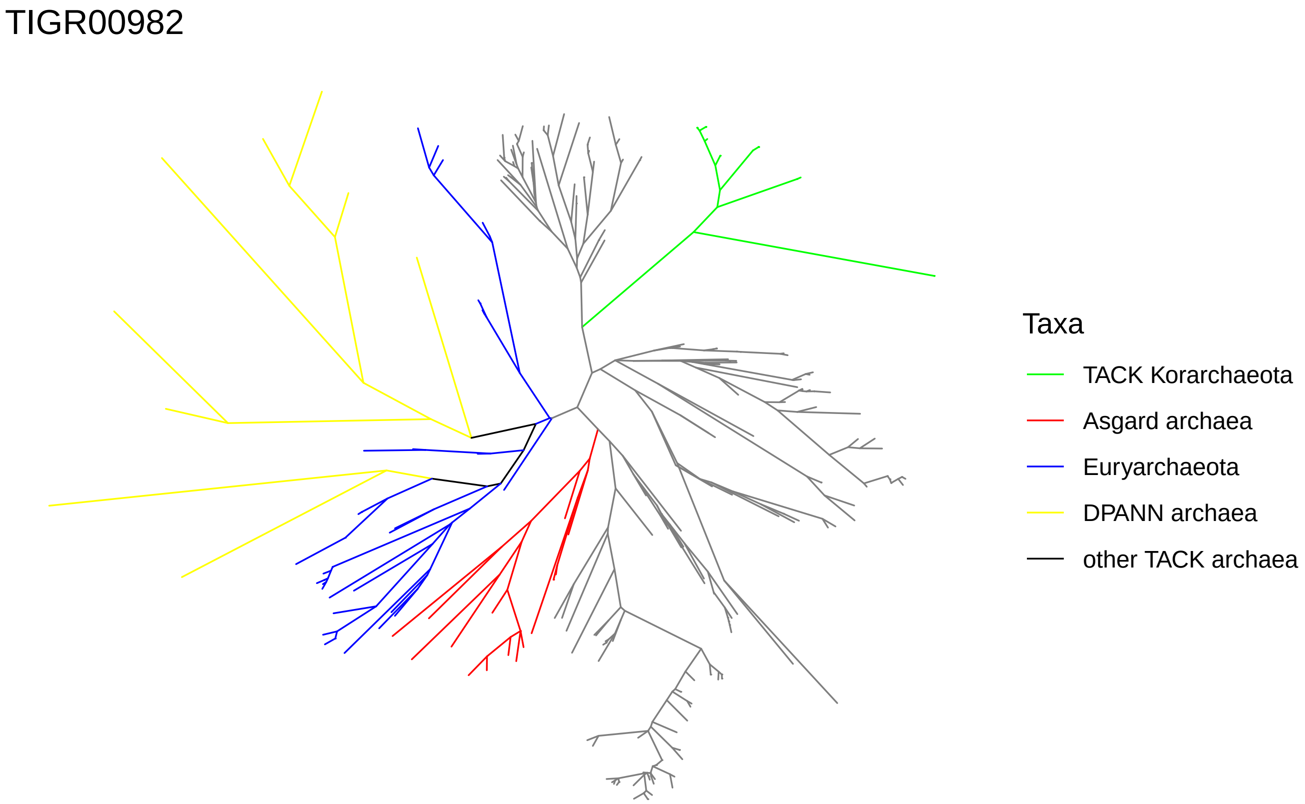
TIGR00982
Taxa
TACK Korarchaeota
Asgard archaea
Euryarchaeota
DPANN archaea
other TACK archaea

TIGR01008

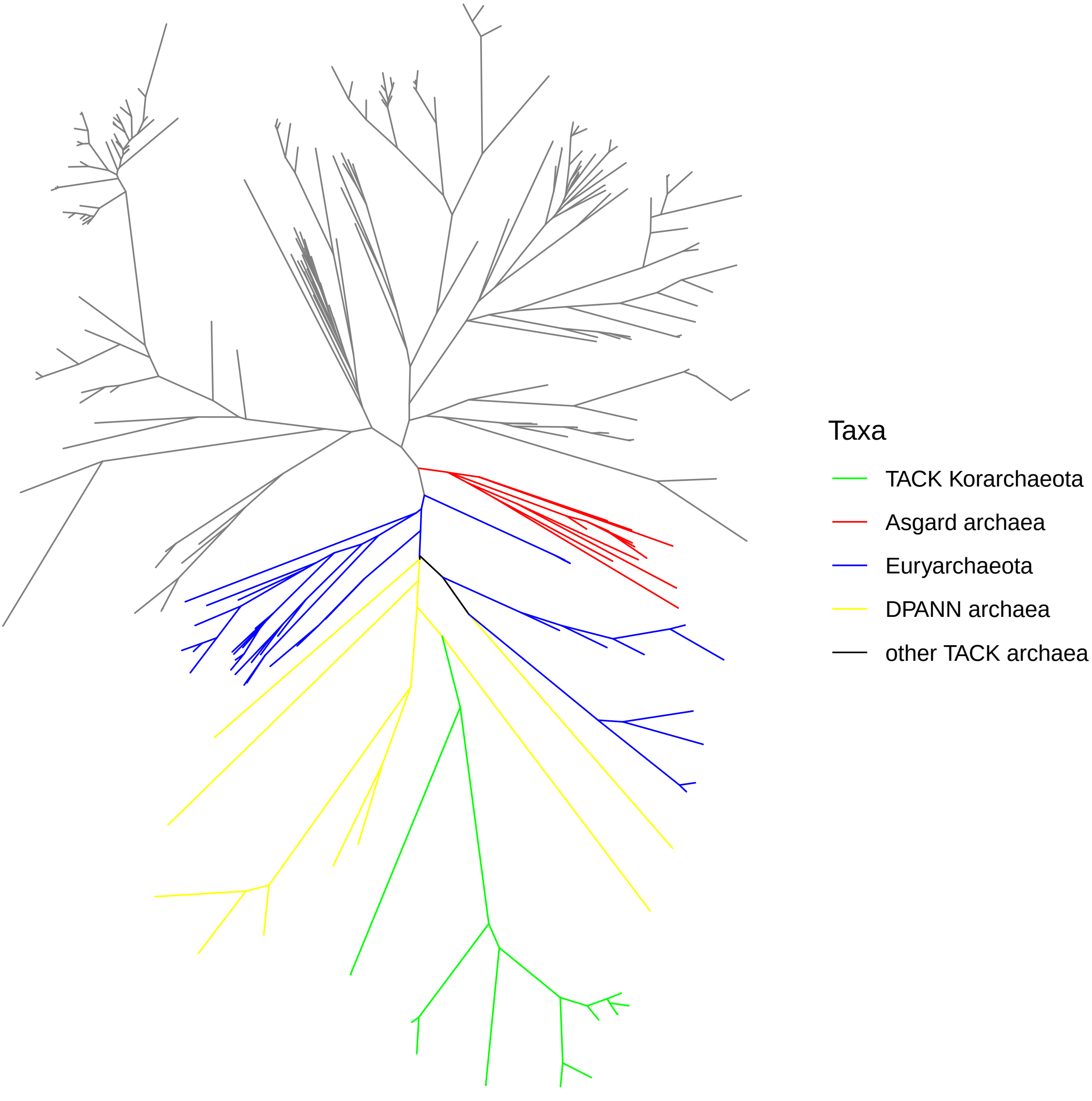

Taxa
TACK Korarchaeota
Asgard archaea
Euryarchaeota
DPANN archaea
other TACK archaea

TIGR01012

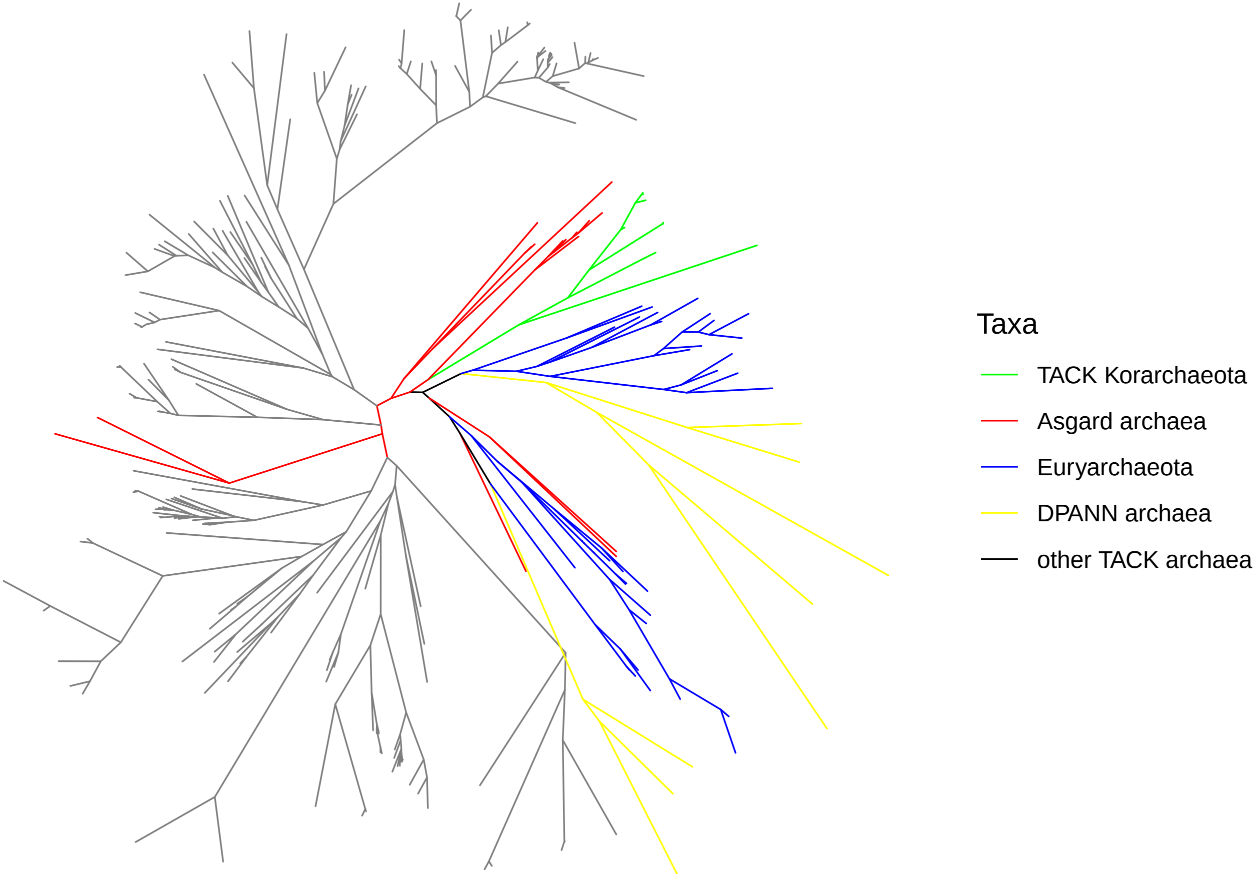
Taxa
TACK Korarchaeota
Asgard archaea
Euryarchaeota
DPANN archaea
other TACK archaea

TIGR01018

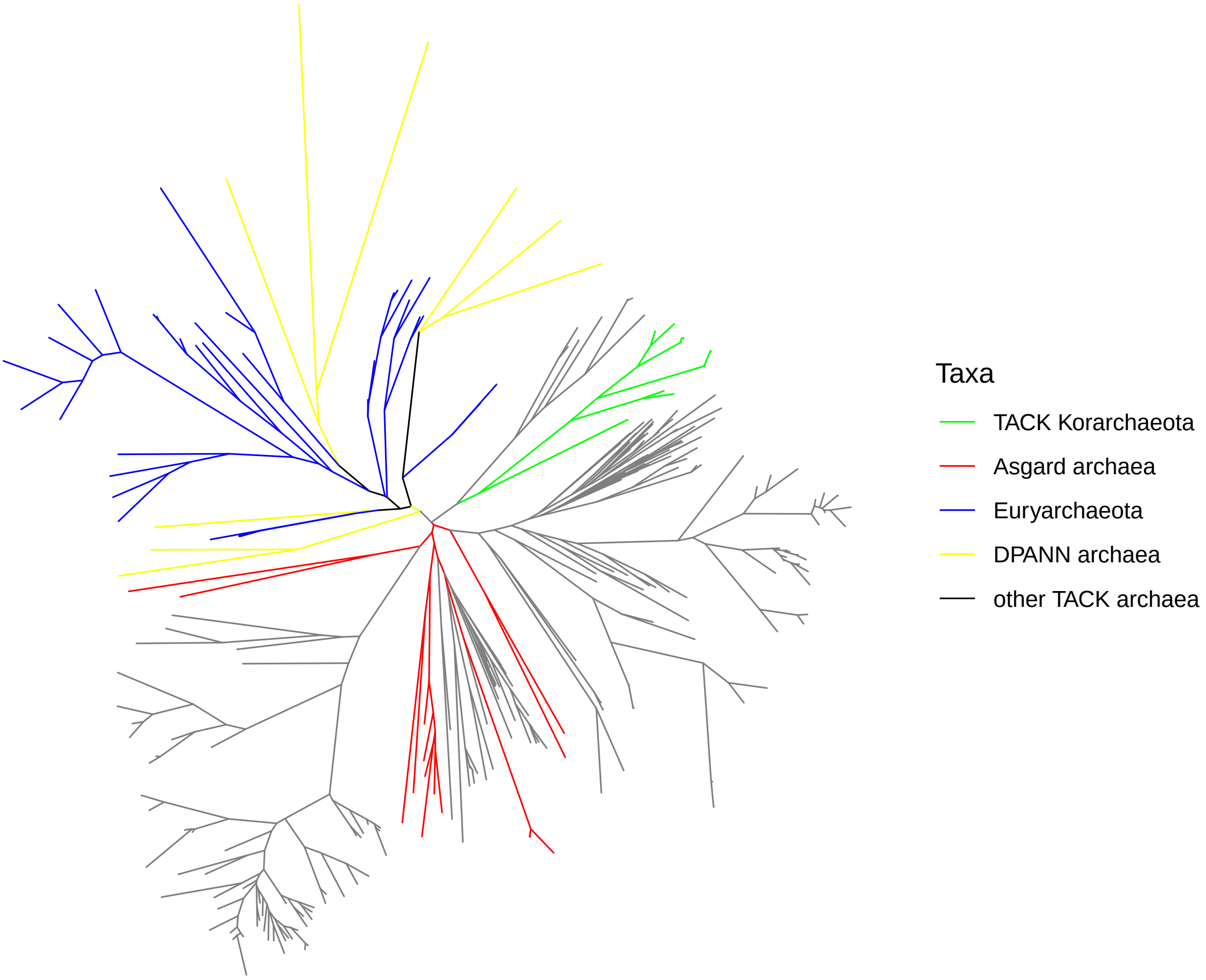

Taxa
TACK Korarchaeota
Asgard archaea
Euryarchaeota
DPANN archaea
other TACK archaea

TIGR01020

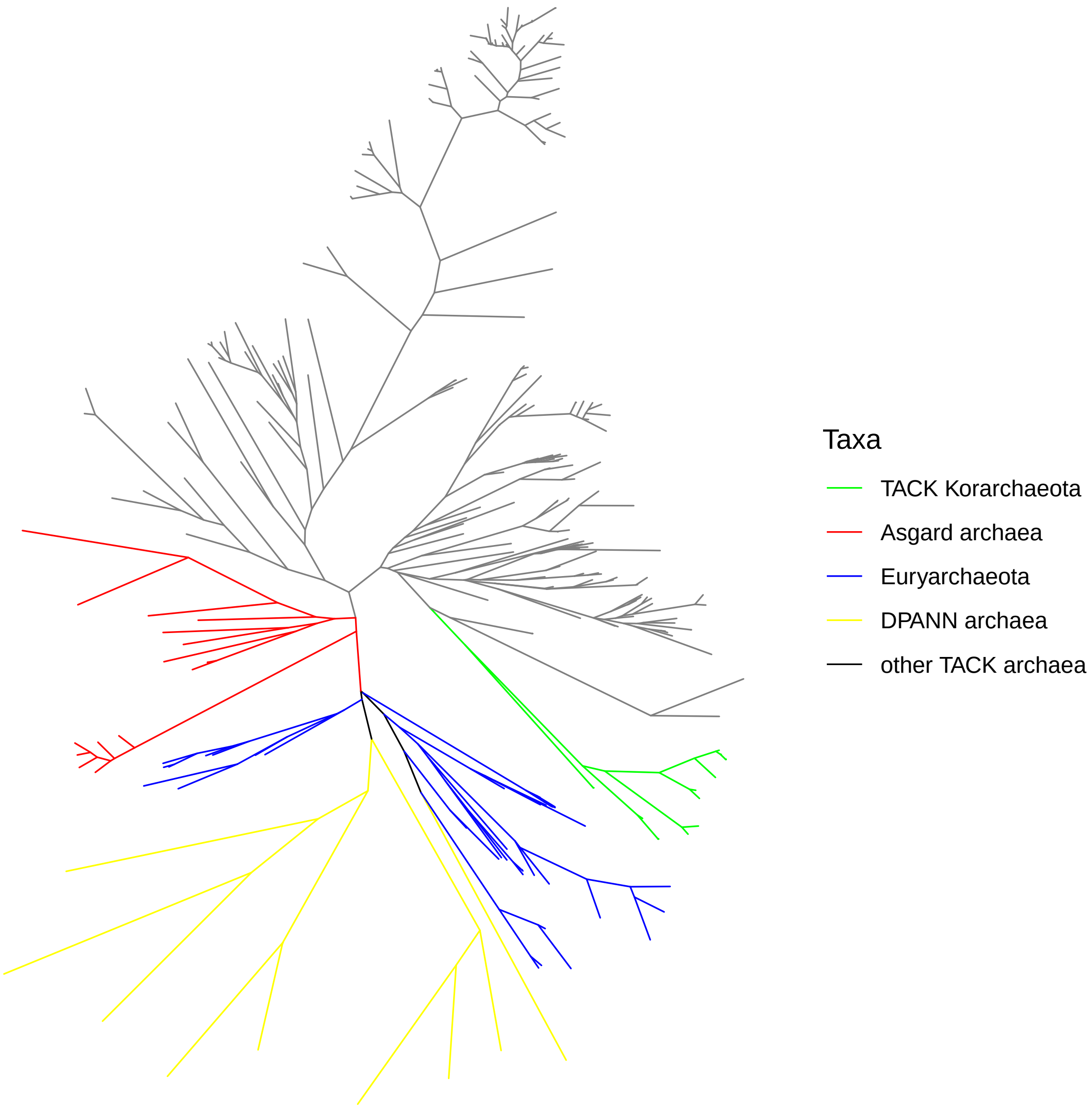
Taxa
TACK Korarchaeota
Asgard archaea
Euryarchaeota
DPANN archaea
other TACK archaea

TIGR01025

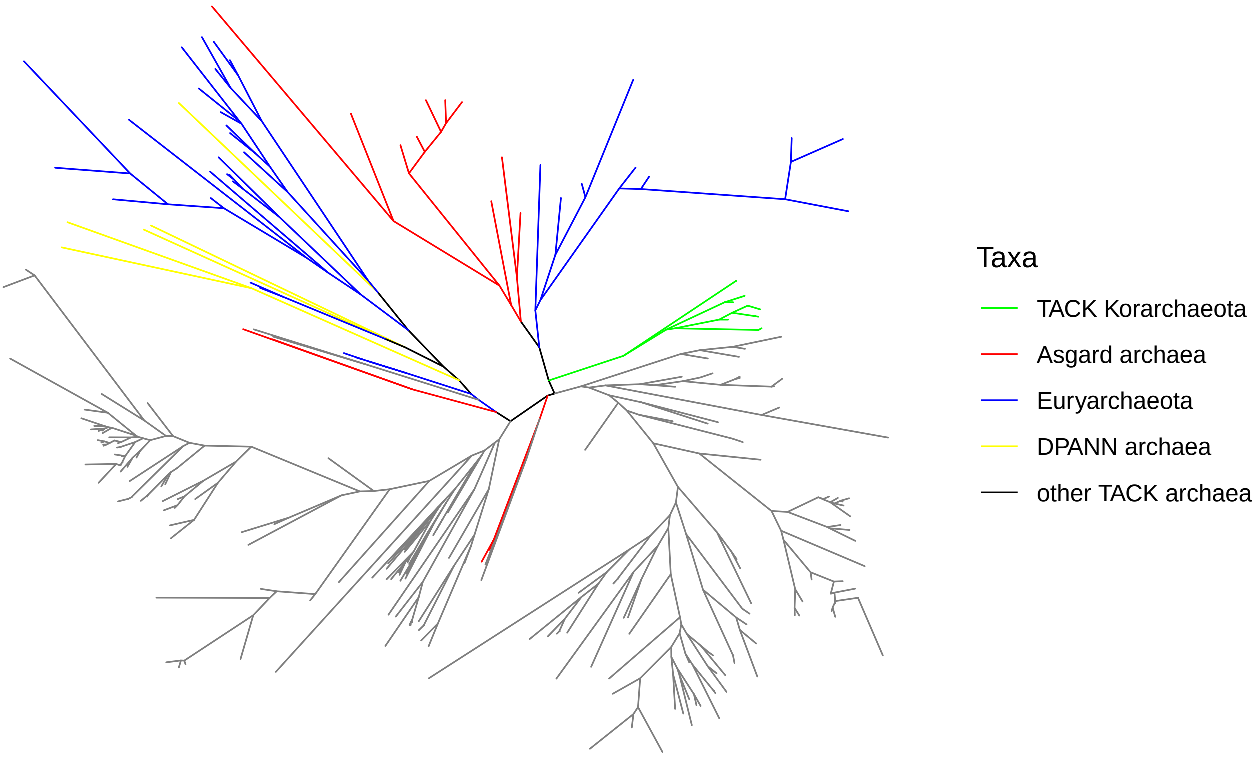
Taxa
TACK Korarchaeota
Asgard archaea
Euryarchaeota
DPANN archaea
other TACK archaea

TIGR01028

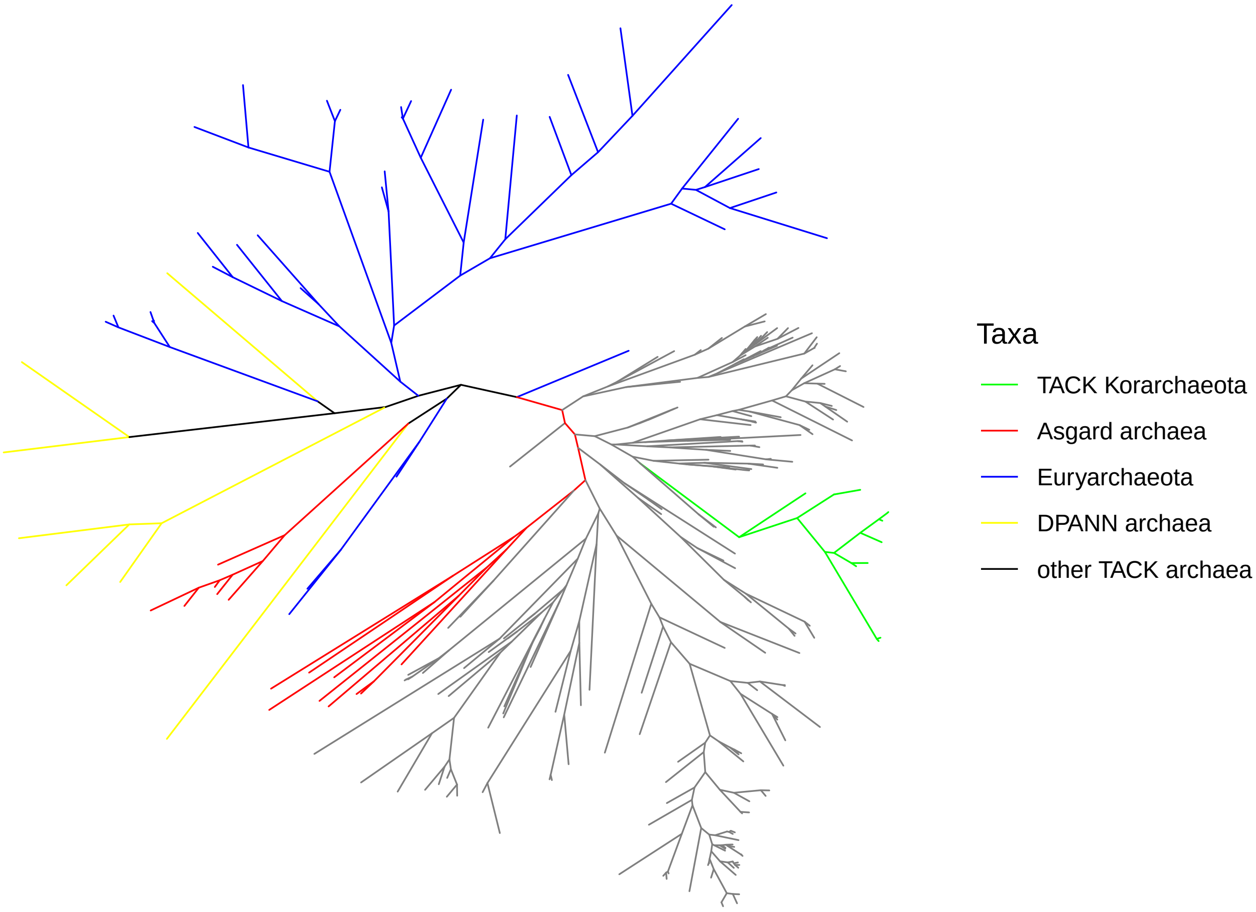
Taxa
TACK Korarchaeota
Asgard archaea
Euryarchaeota
DPANN archaea
other TACK archaea

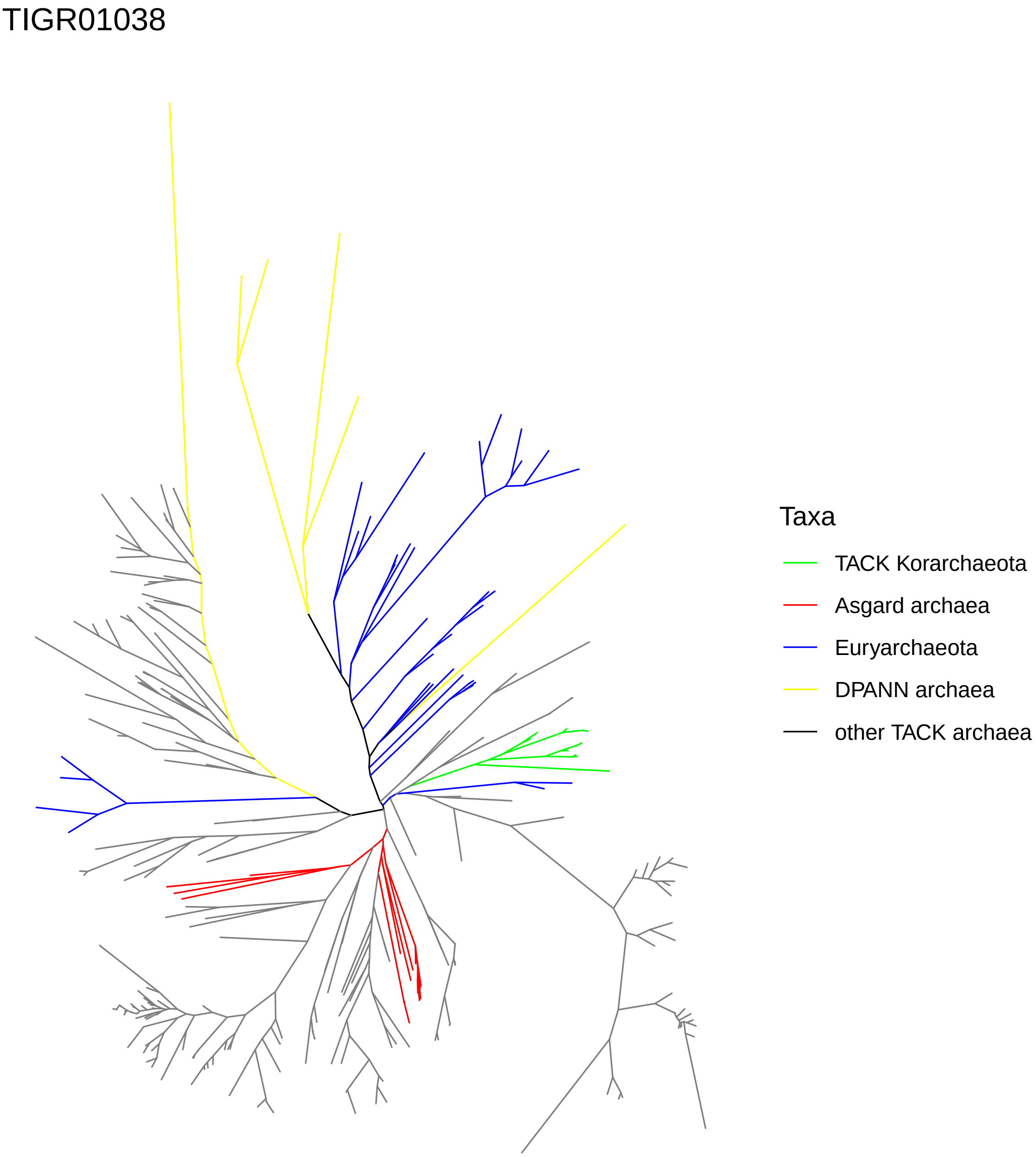
TIGR01038
Taxa
TACK Korarchaeota
Asgard archaea
Euryarchaeota
DPANN archaea
other TACK archaea

TIGR01046

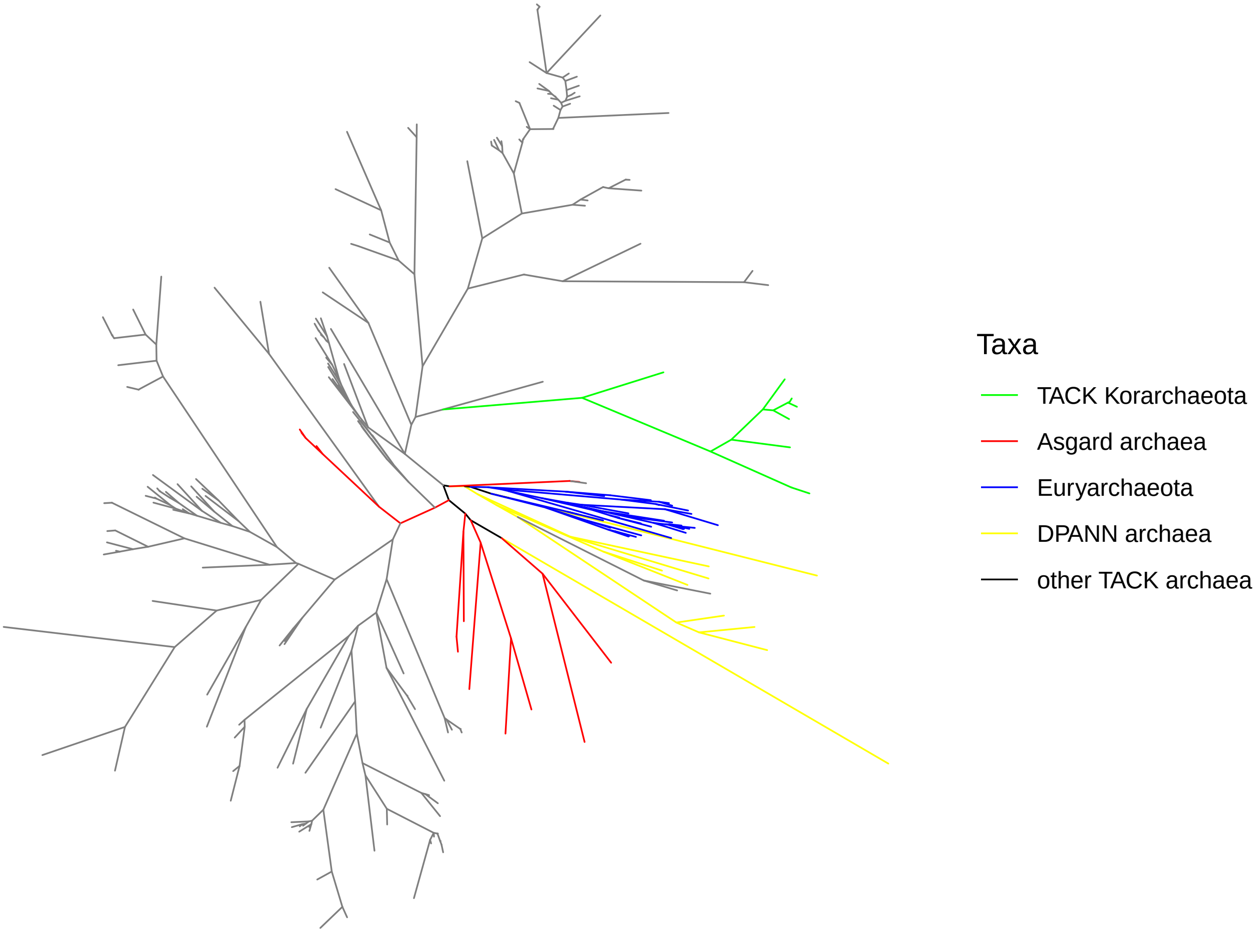

Taxa
TACK Korarchaeota
Asgard archaea
Euryarchaeota
DPANN archaea
other TACK archaea

TIGR01052

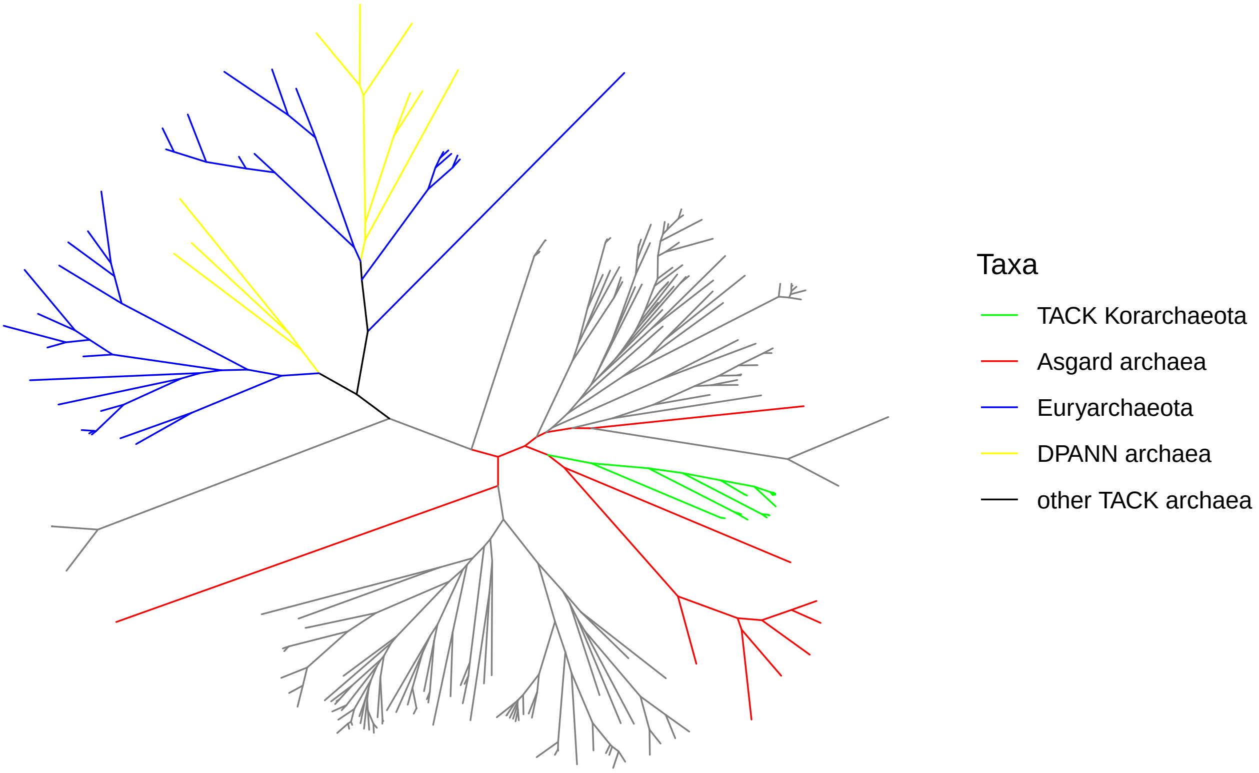

Taxa
TACK Korarchaeota
Asgard archaea
Euryarchaeota
DPANN archaea
other TACK archaea

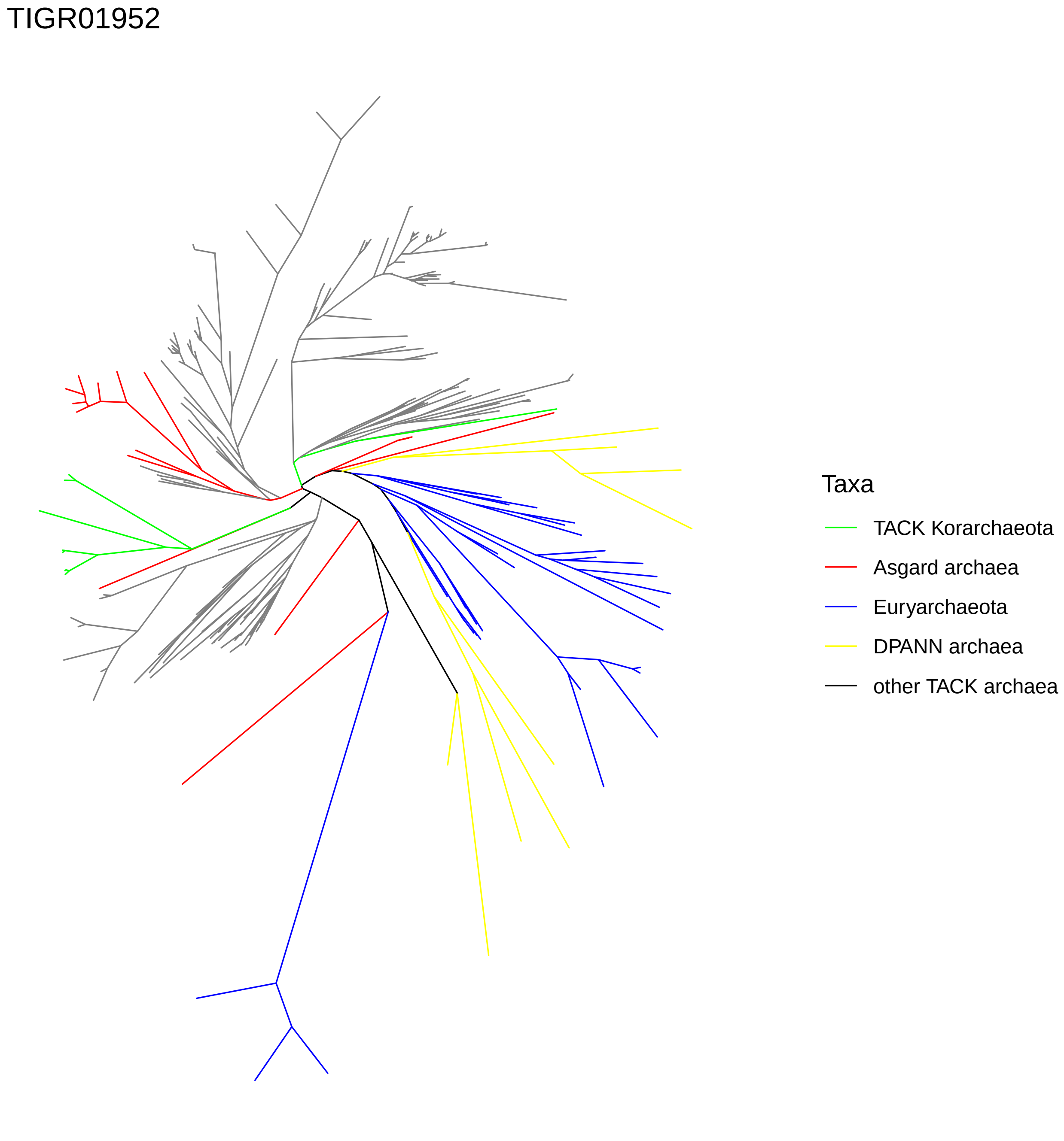
TIGR01952
Taxa
TACK Korarchaeota
Asgard archaea
Euryarchaeota
DPANN archaea
other TACK archaea

TIGR02065

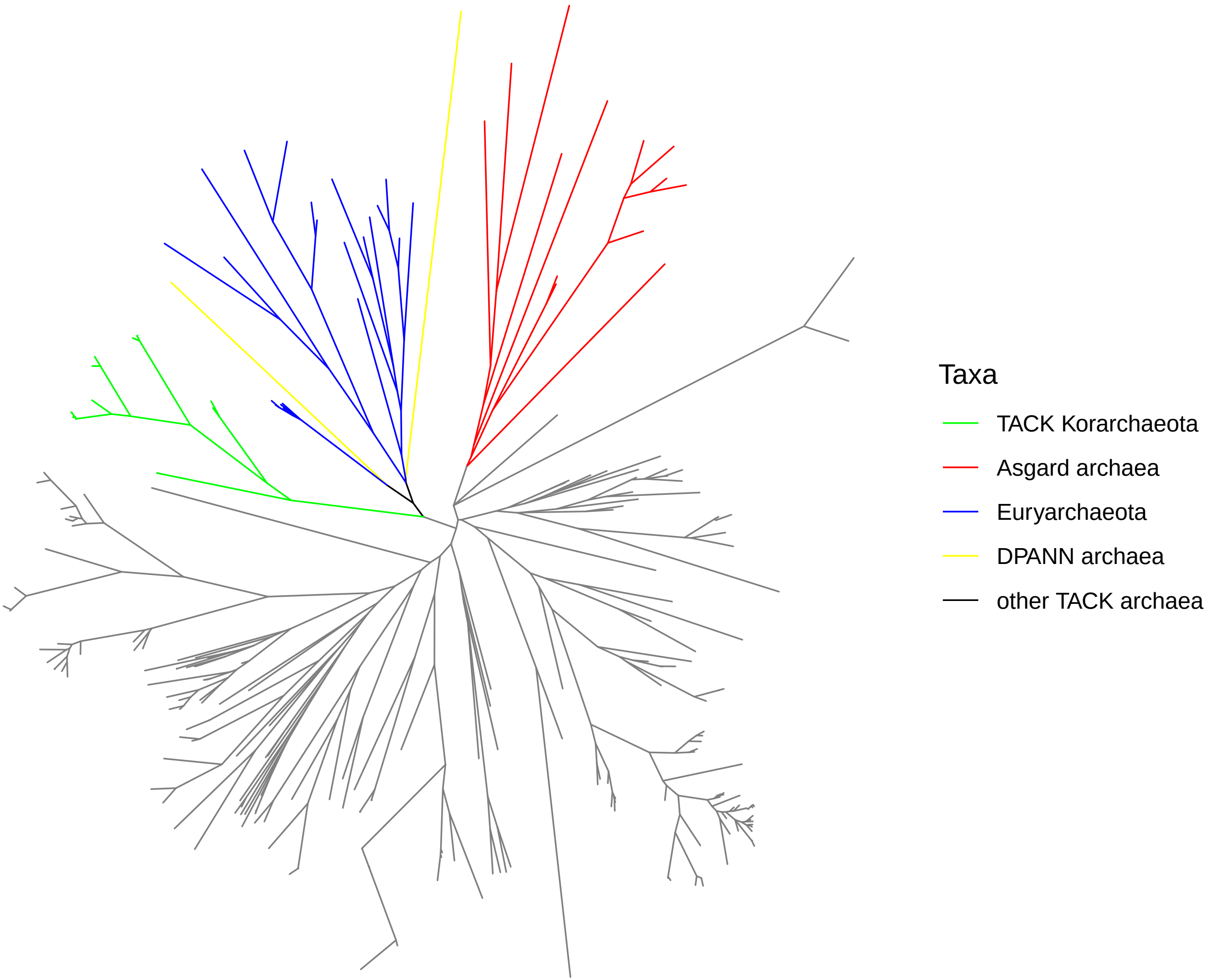

Taxa
TACK Korarchaeota
Asgard archaea
Euryarchaeota
DPANN archaea
other TACK archaea

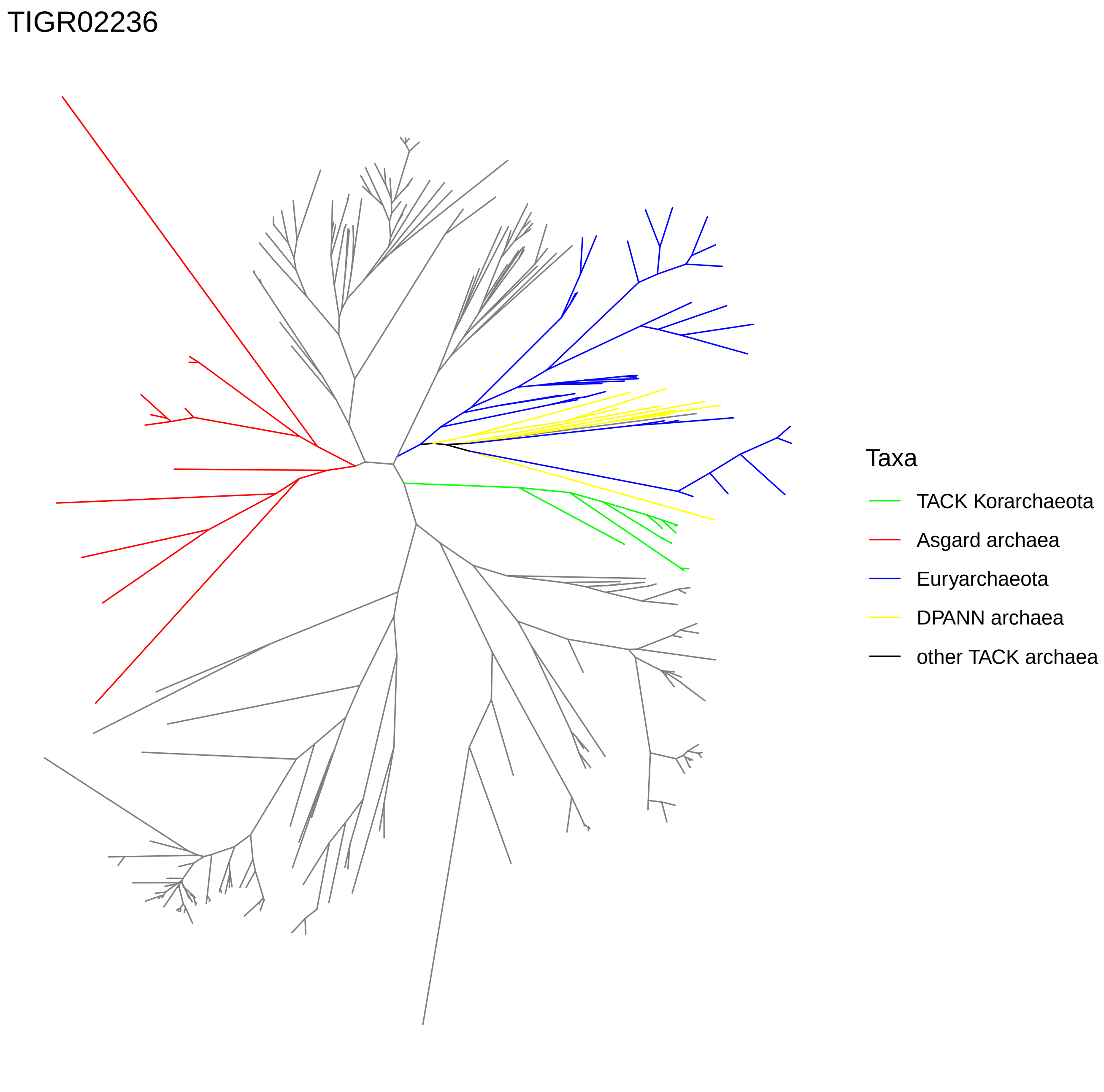
TIGR02236
Taxa
TACK Korarchaeota
Asgard archaea
Euryarchaeota
DPANN archaea
other TACK archaea

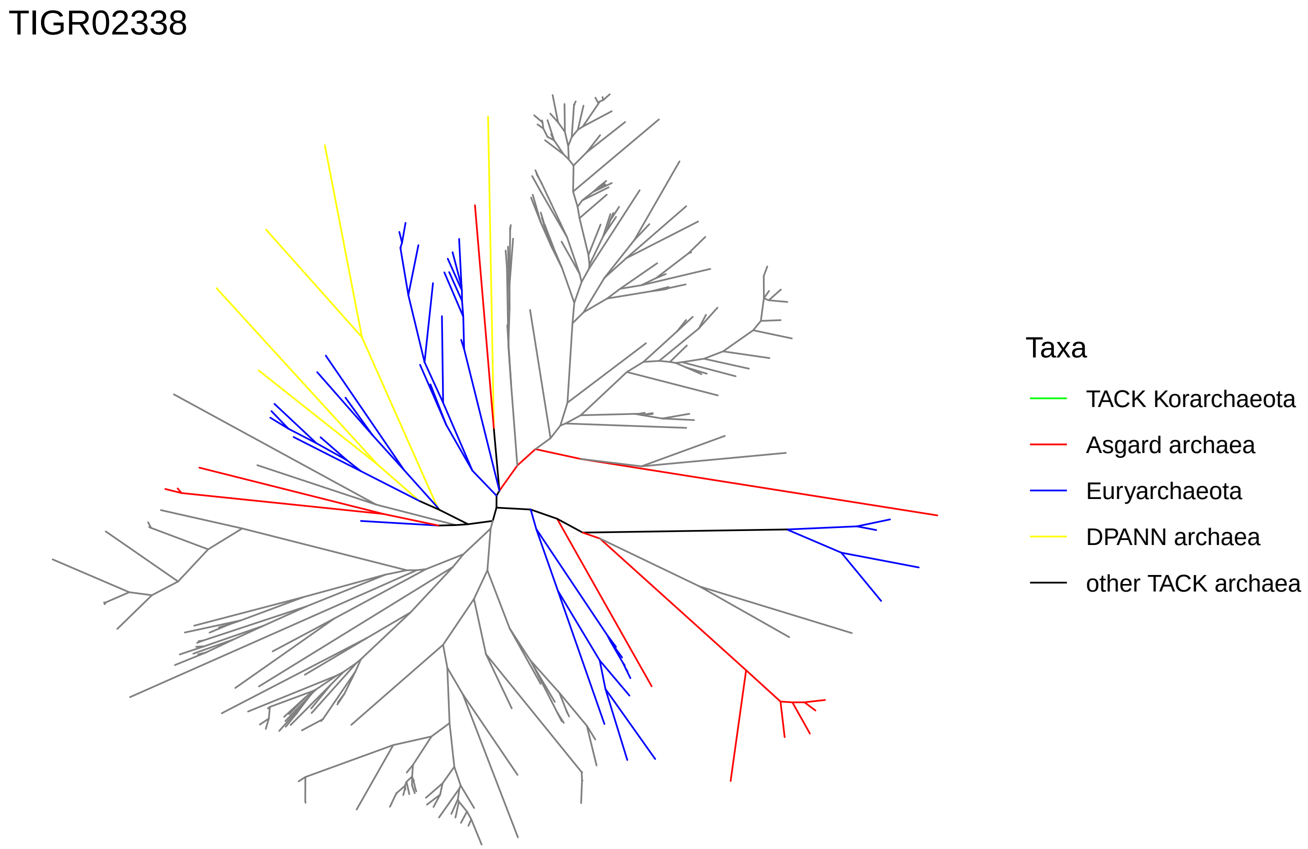
TIGR02338
Taxa
TACK Korarchaeota
Asgard archaea
Euryarchaeota
DPANN archaea
other TACK archaea

TIGR02389

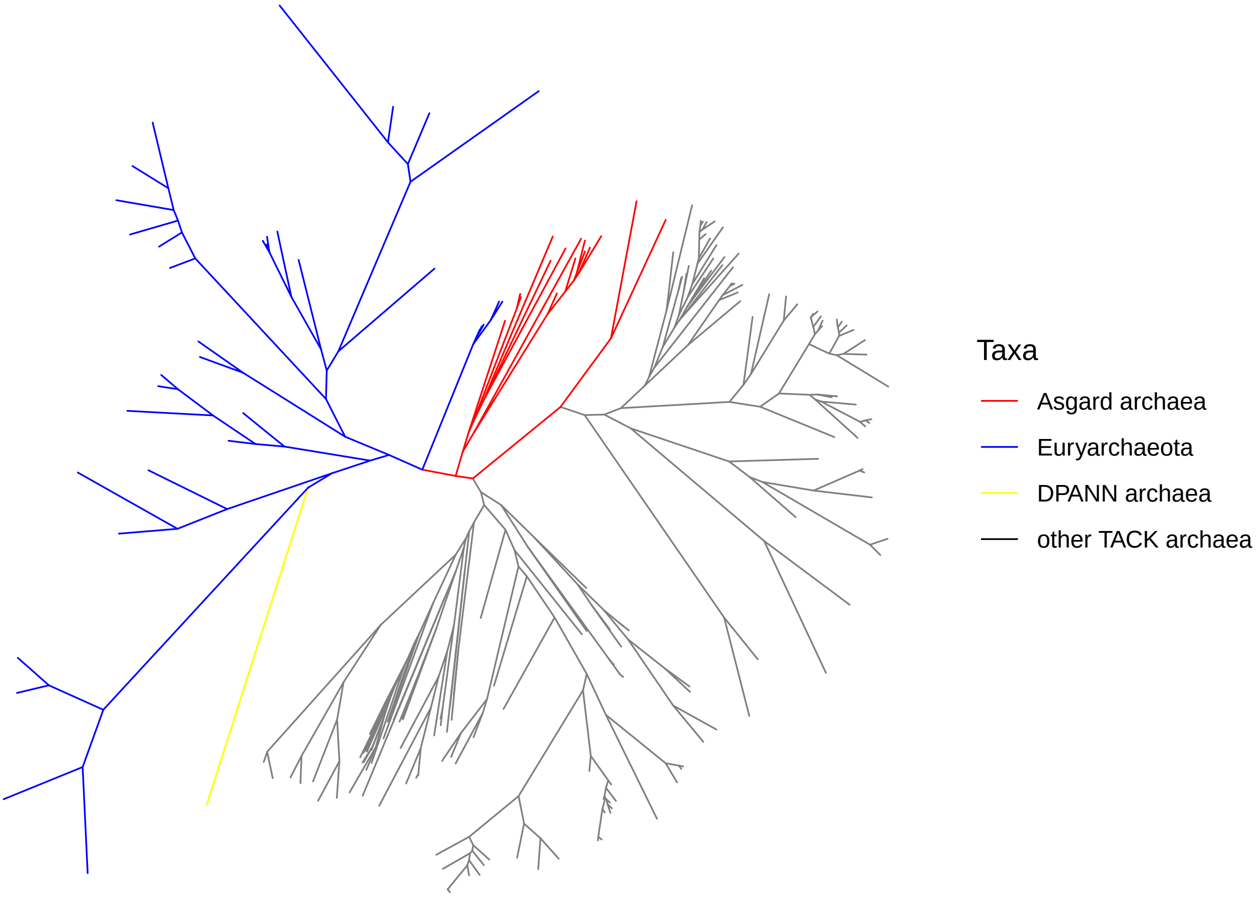
Taxa
Asgard archaea
Euryarchaeota
DPANN archaea
other TACK archaea

TIGR02390

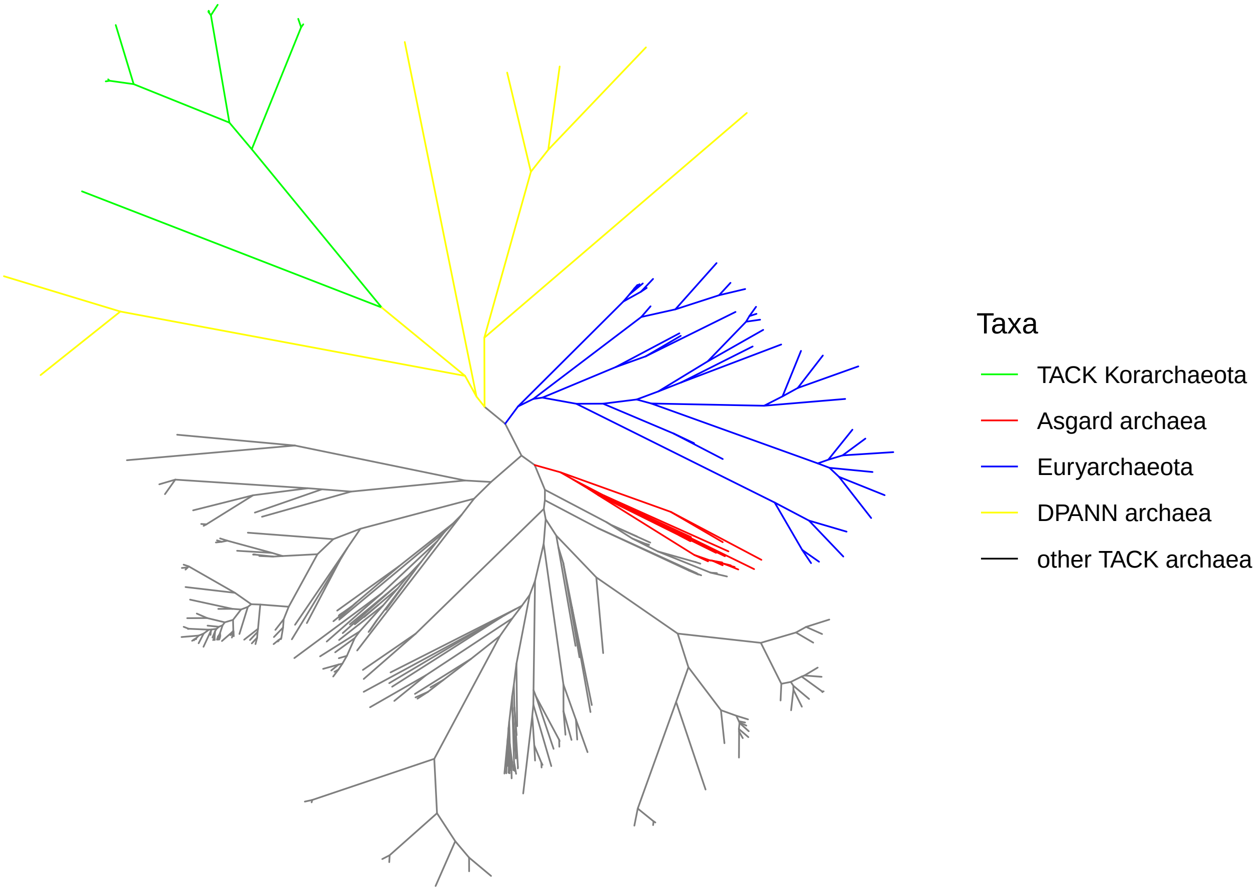

Taxa
TACK Korarchaeota
Asgard archaea
Euryarchaeota
DPANN archaea
other TACK archaea

TIGR02928

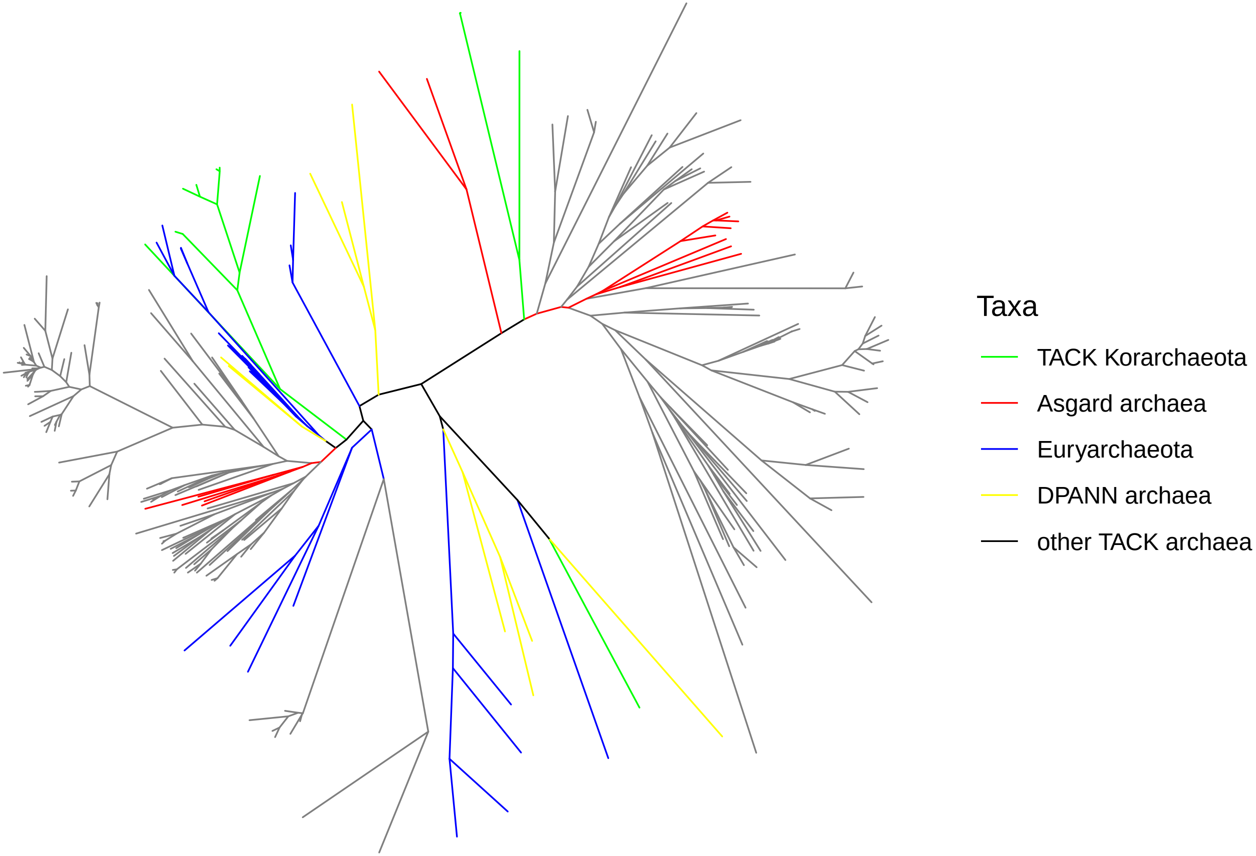

Taxa
TACK Korarchaeota
Asgard archaea
Euryarchaeota
DPANN archaea
other TACK archaea

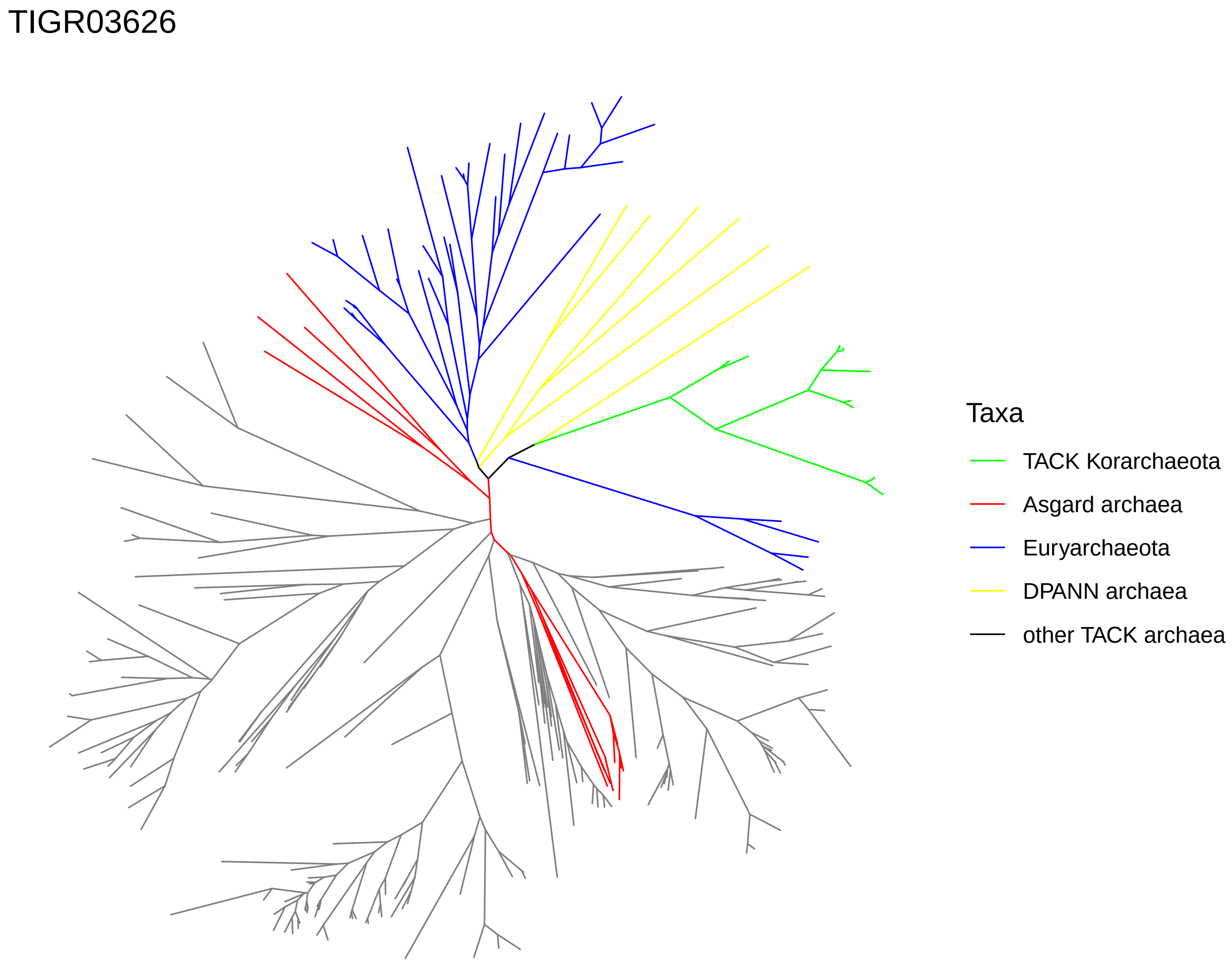
TIGR03626
Taxa
TACK Korarchaeota
Asgard archaea
Euryarchaeota
DPANN archaea
other TACK archaea

TIGR03627

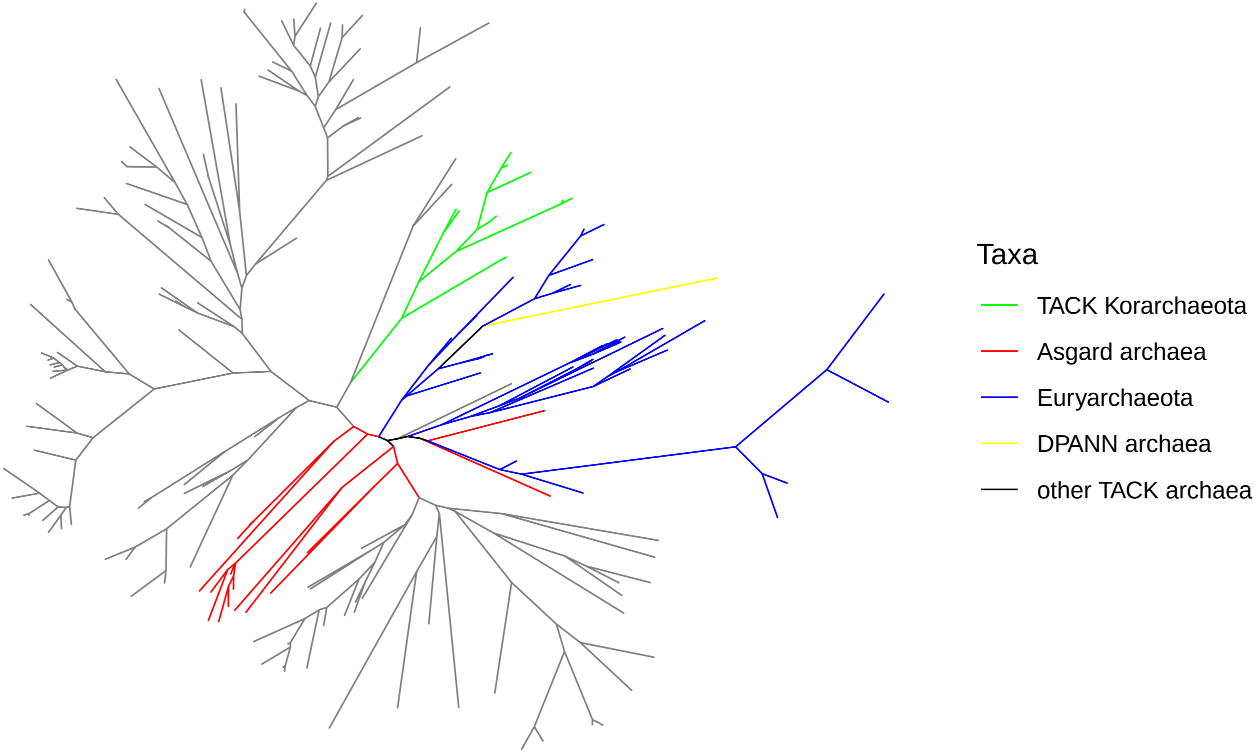
Taxa
TACK Korarchaeota
Asgard archaea
Euryarchaeota
DPANN archaea
other TACK archaea

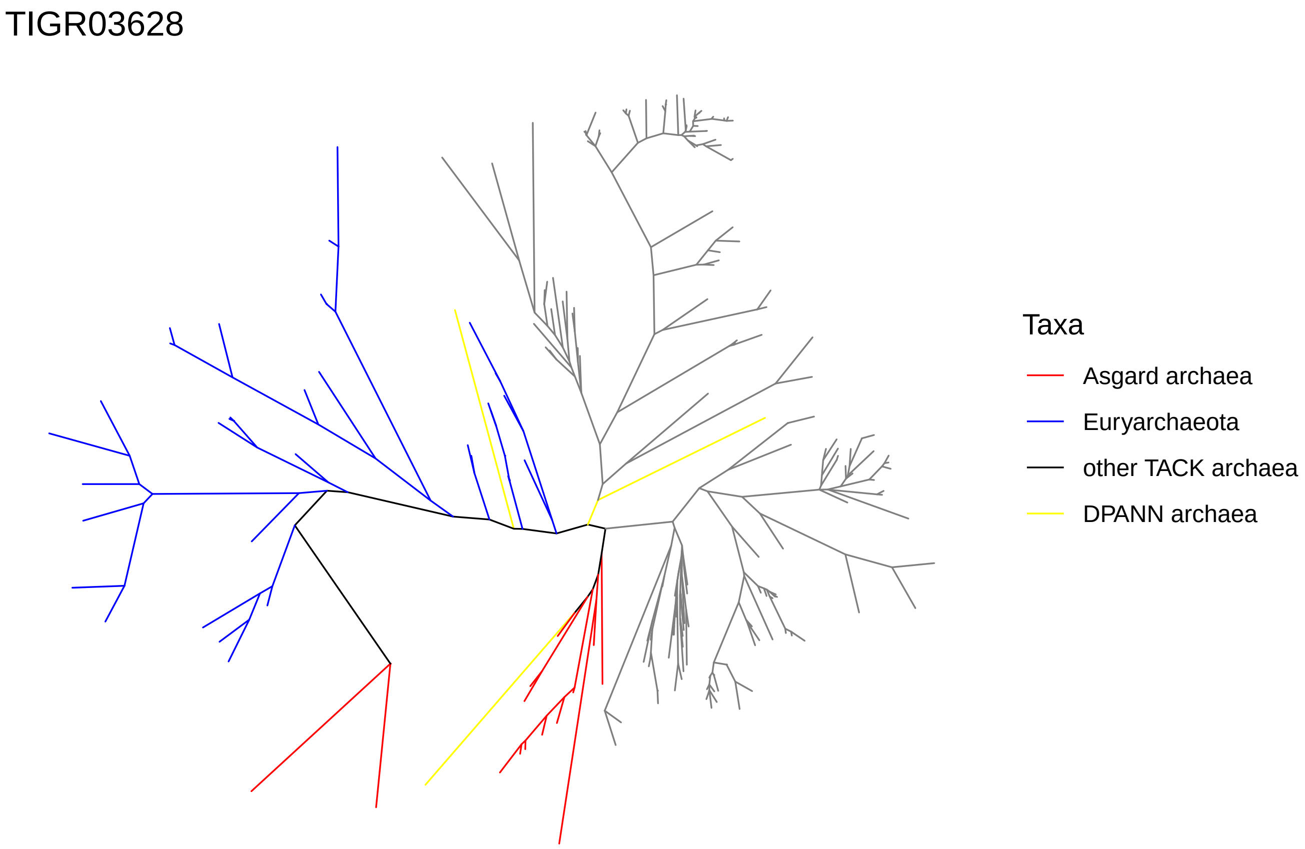
TIGR03628
Taxa
Asgard archaea
Euryarchaeota
other TACK archaea
DPANN archaea

TIGR03629

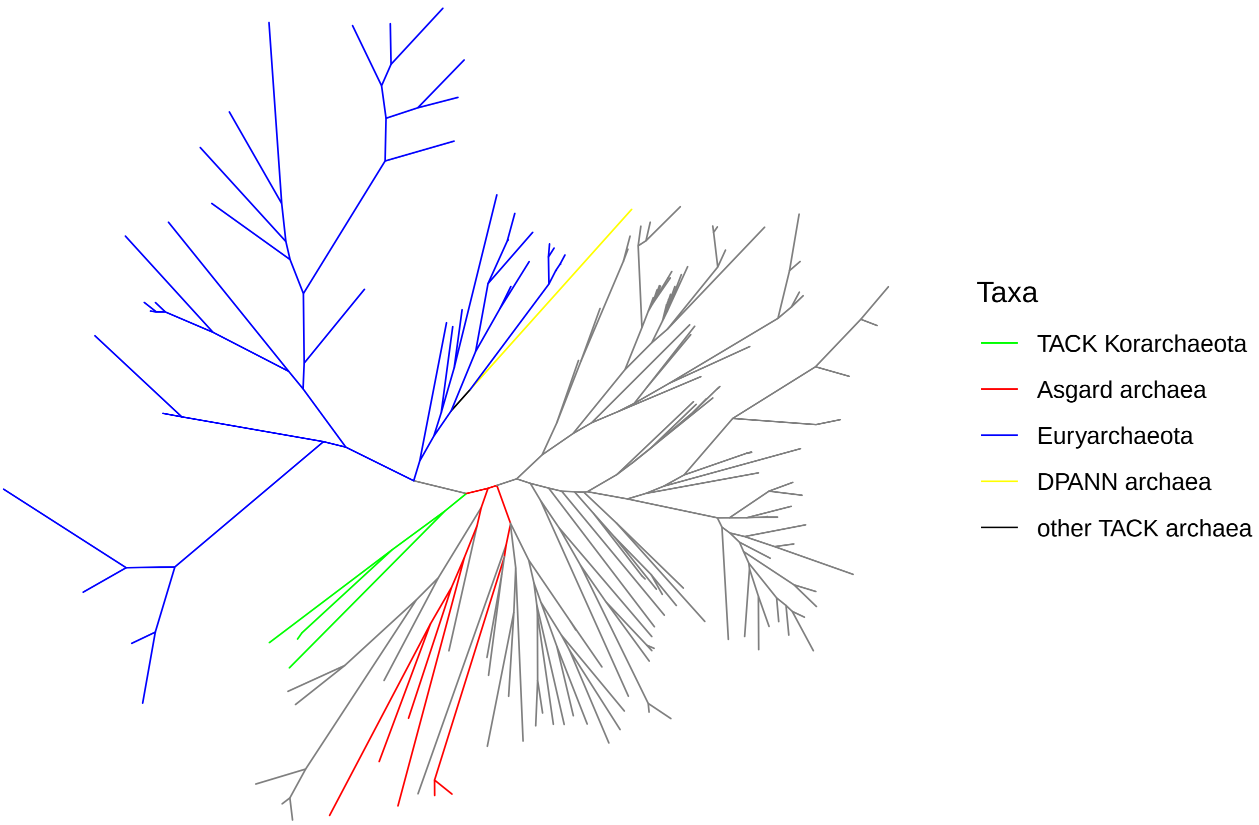
Taxa
TACK Korarchaeota
Asgard archaea
Euryarchaeota
DPANN archaea
other TACK archaea

TIGR03653

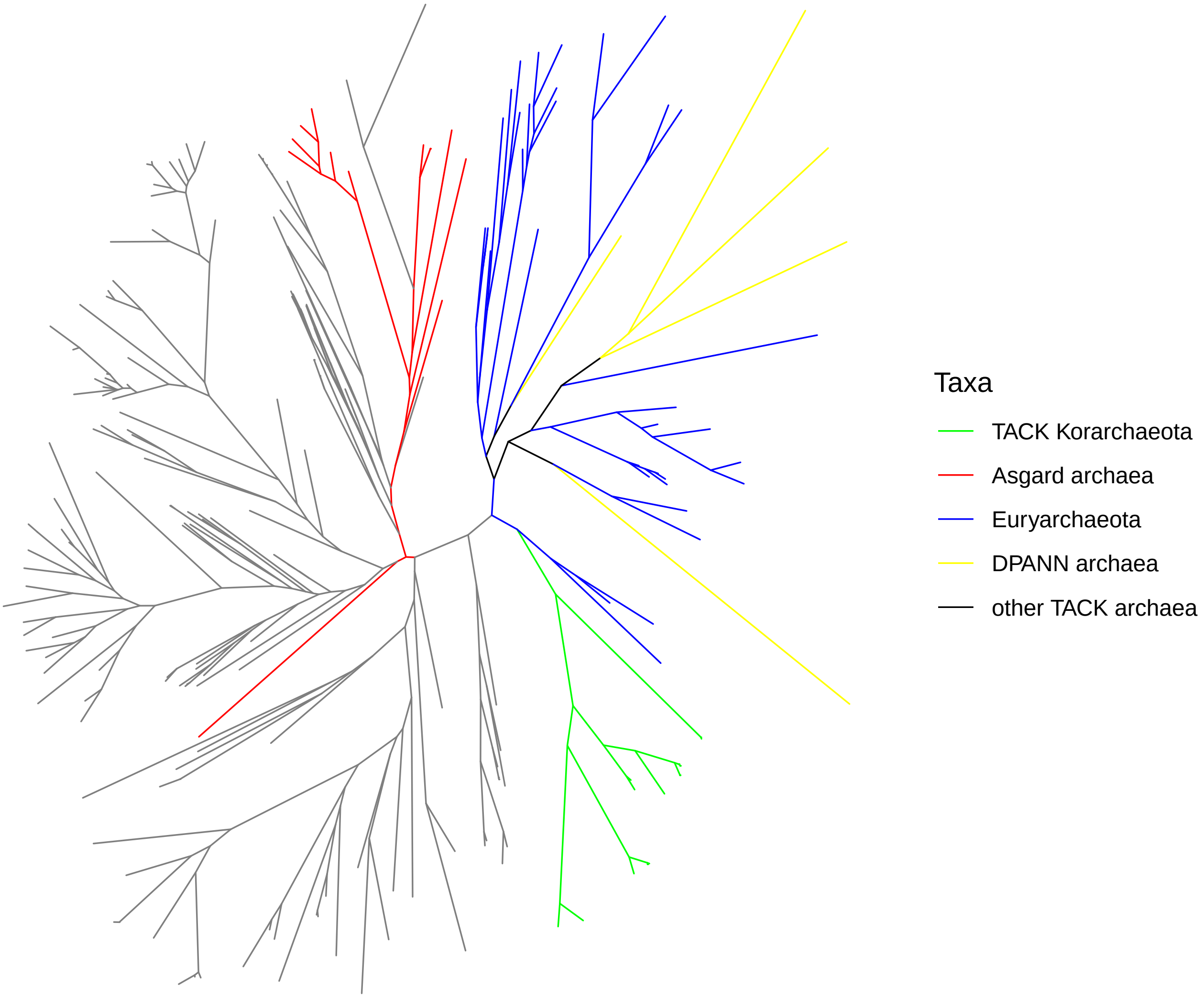
Taxa
TACK Korarchaeota
Asgard archaea
Euryarchaeota
DPANN archaea
other TACK archaea

TIGR03670

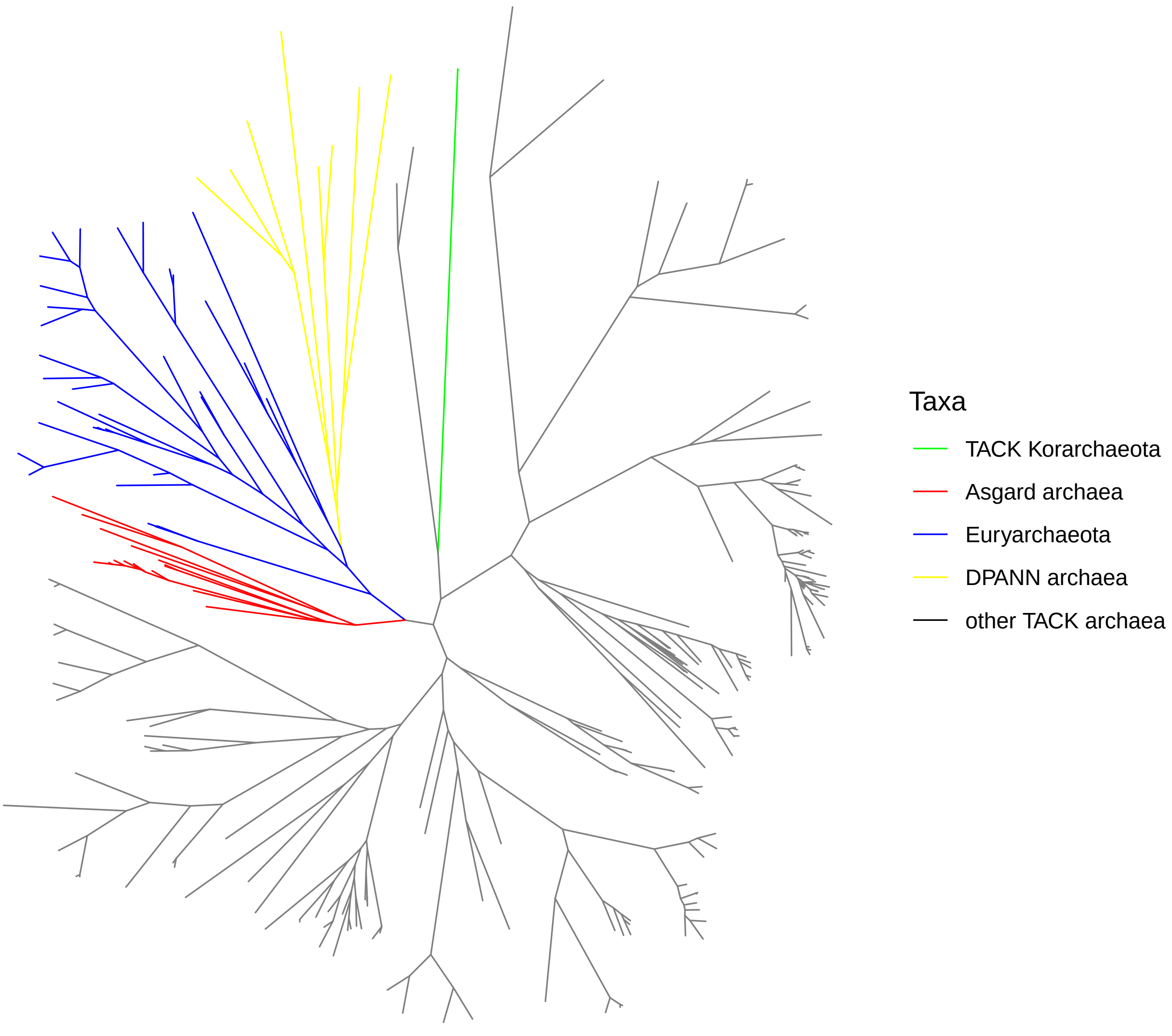
Taxa
TACK Korarchaeota
Asgard archaea
Euryarchaeota
DPANN archaea
other TACK archaea

TIGR03672

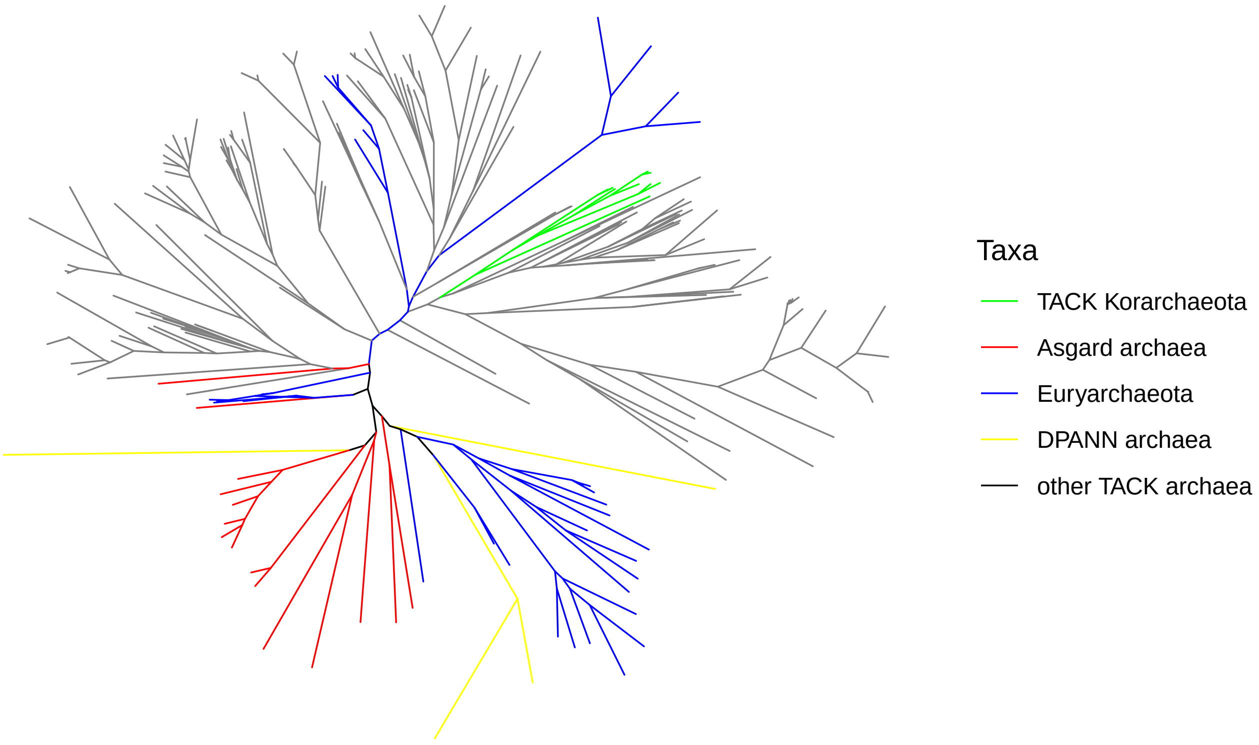

Taxa
TACK Korarchaeota
Asgard archaea
Euryarchaeota
DPANN archaea
other TACK archaea

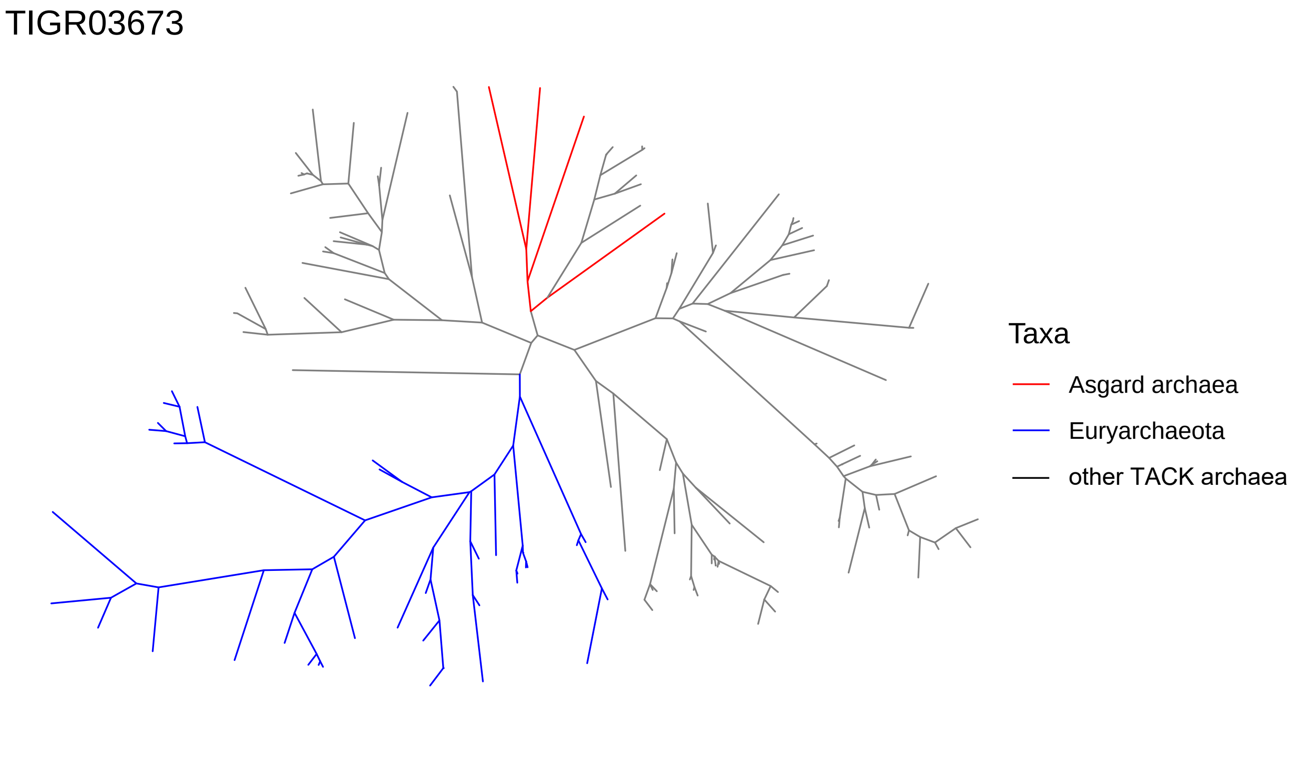

TIGR03673
Taxa
Asgard archaea
Euryarchaeota
other TACK archaea

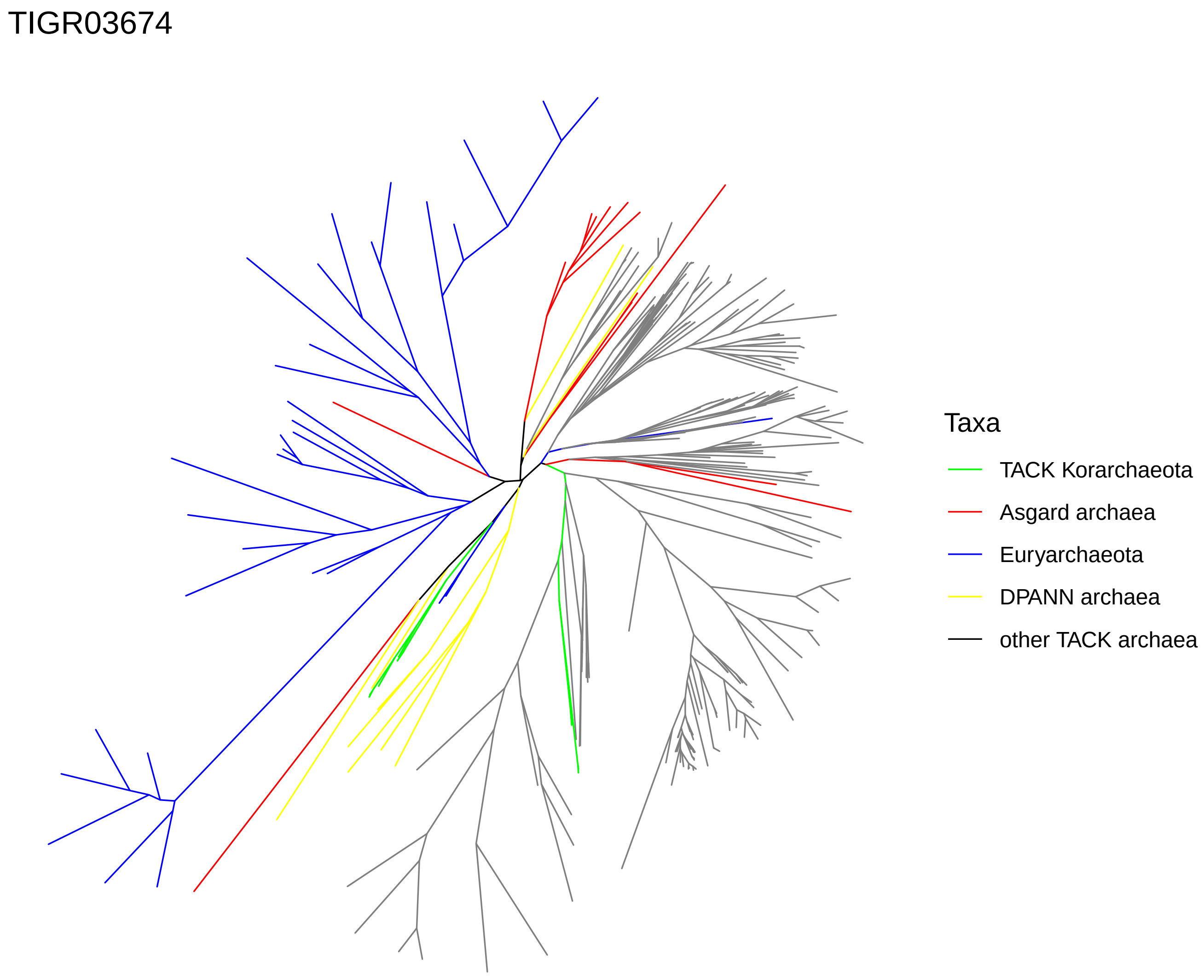

TIGR03674
Taxa
TACK Korarchaeota
Asgard archaea
Euryarchaeota
DPANN archaea
other TACK archaea

TIGR03676

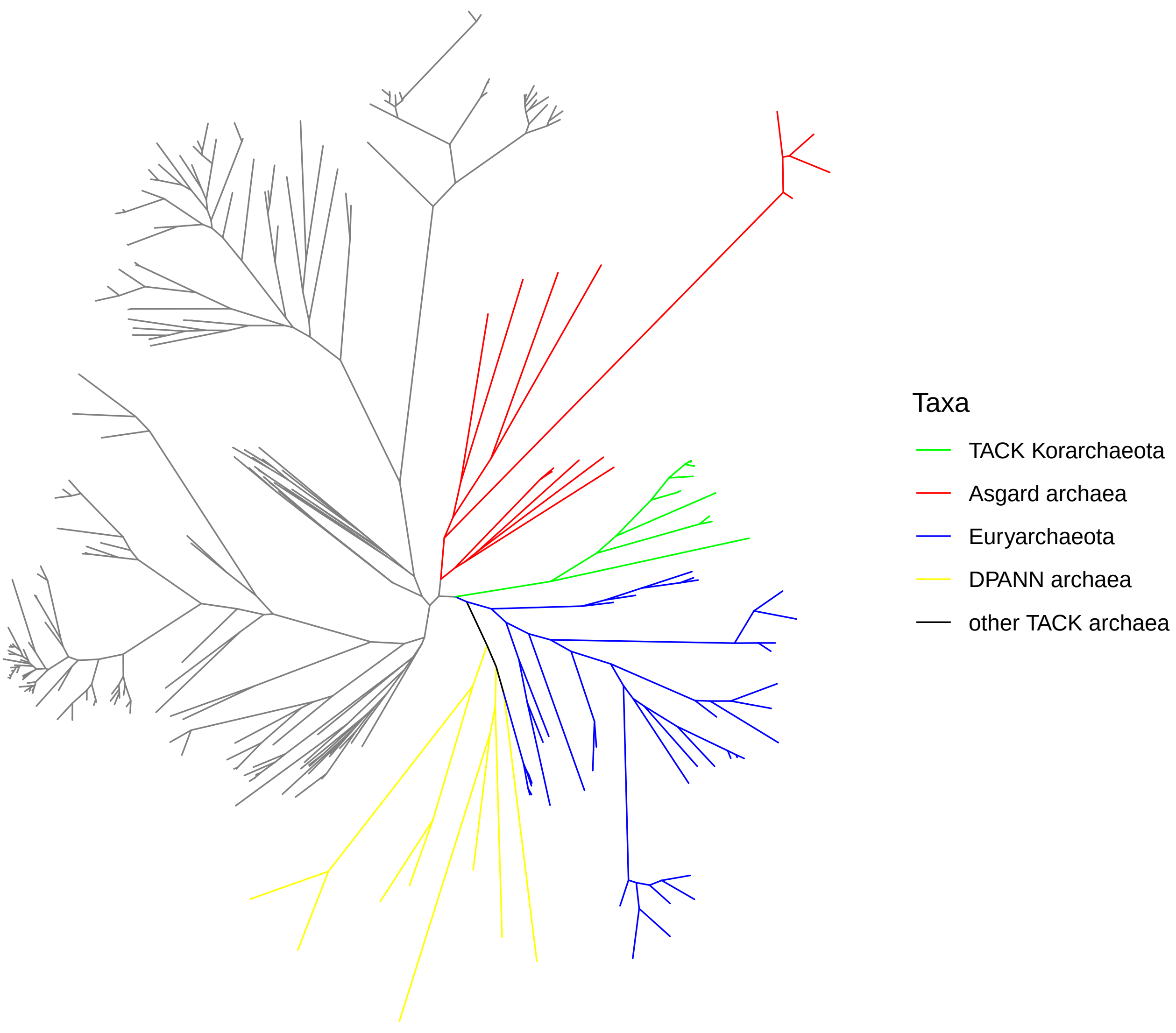

Taxa
TACK Korarchaeota
Asgard archaea
Euryarchaeota
DPANN archaea
other TACK archaea

TIGR03680

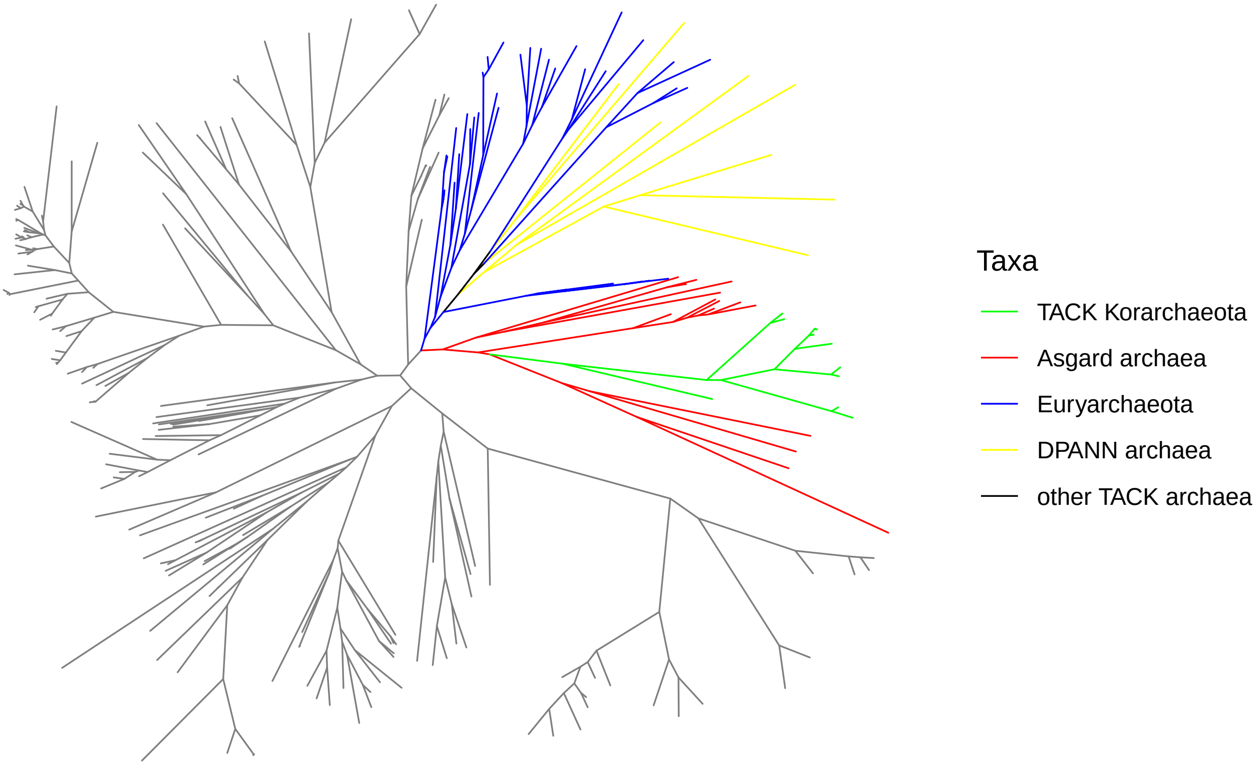
Taxa
TACK Korarchaeota
Asgard archaea
Euryarchaeota
DPANN archaea
other TACK archaea

TIGR03722

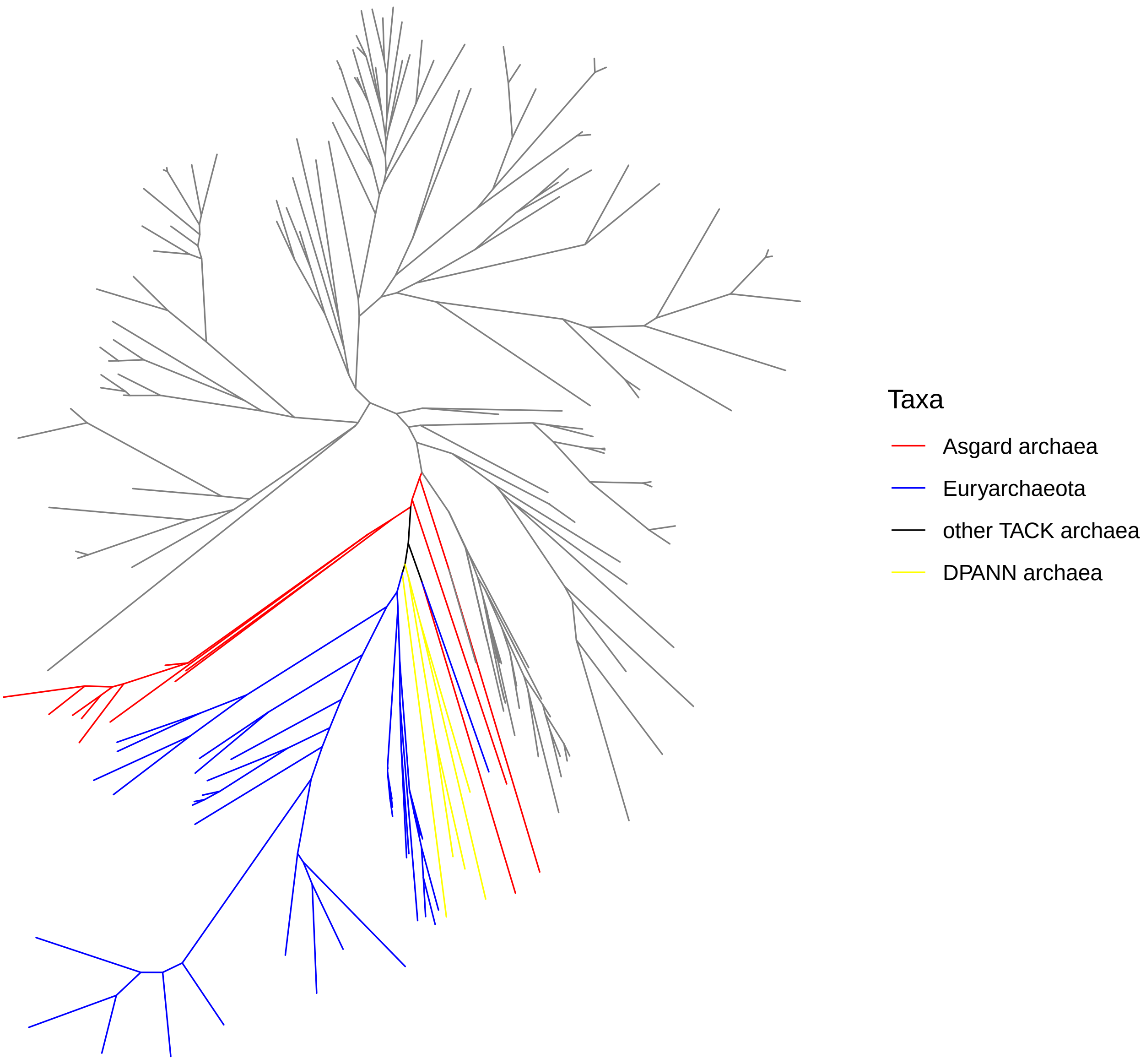
Taxa
Asgard archaea
Euryarchaeota
other TACK archaea
DPANN archaea
