# Supplementary material for: Wood–Ljungdahl pathway found in novel marine Korarchaeota groups illuminates their evolutionary history
Source: mSystems. 2023 Jul 17;8(4):e00305-23. doi: 10.1128/msystems.00305-23 (PMC10469681; doi:10.1128/msystems.00305-23)
Supplement: Figure S15 — Phylogenetic trees constructed using concatenated sequences of the cdhABCDE genes and those based on single genes. [file msystems.00305-23-s0004.pdf]

Figure S15

a. concatenate sequences of *cdhABCDE*

iqtree: LG+F+R6

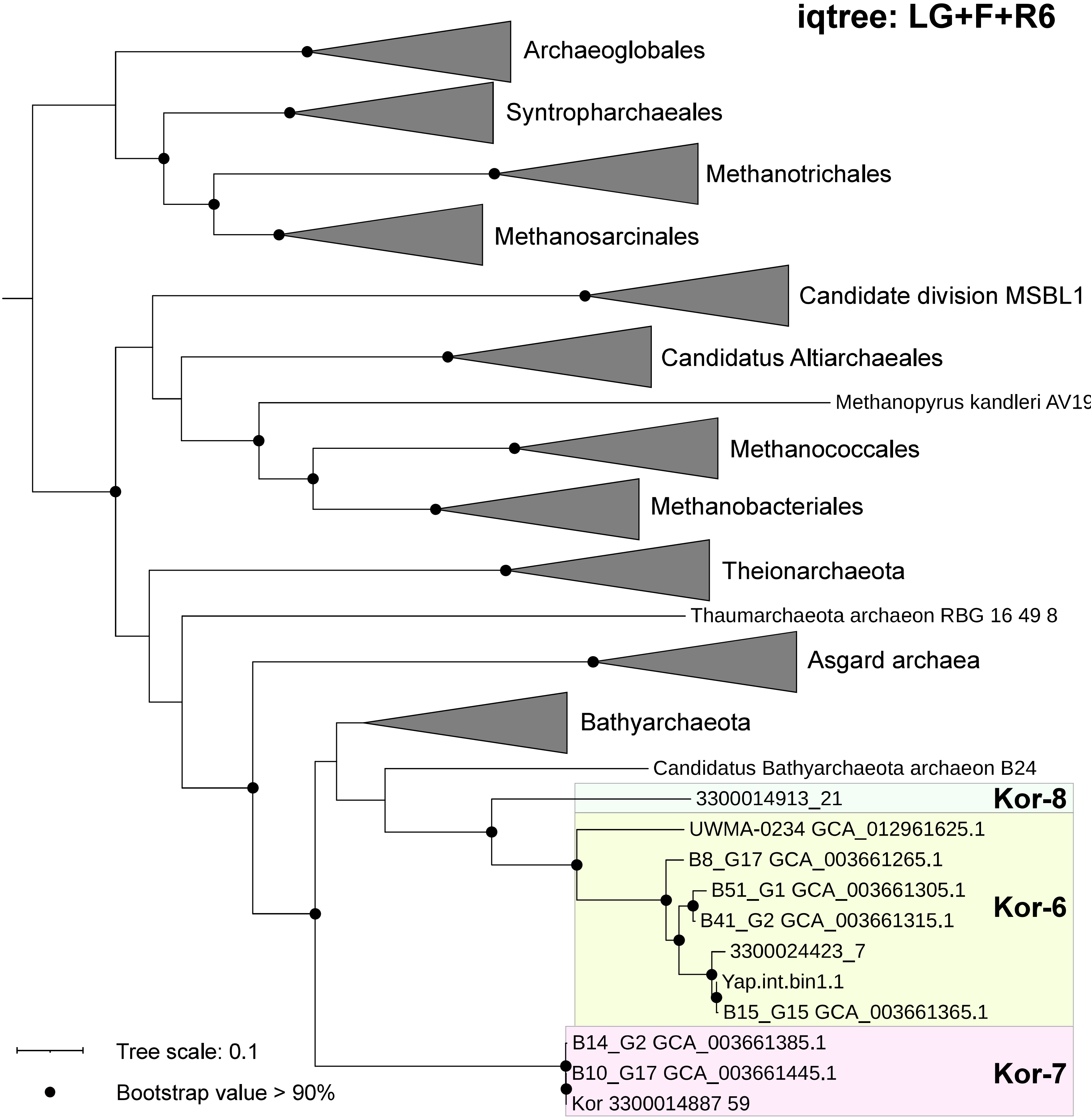

Figure S15 b. *cdhA*
iqtree: LG+R5

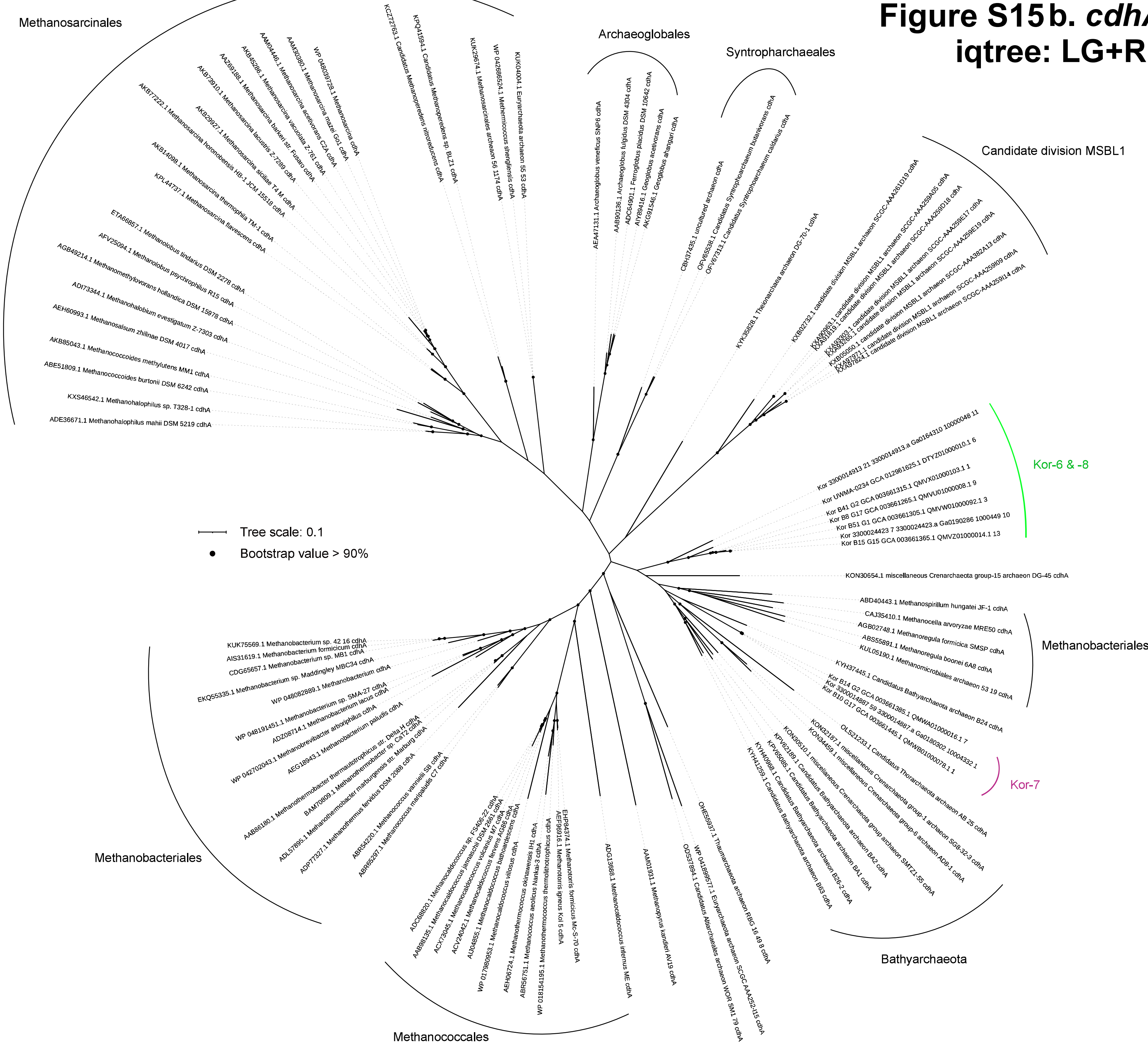

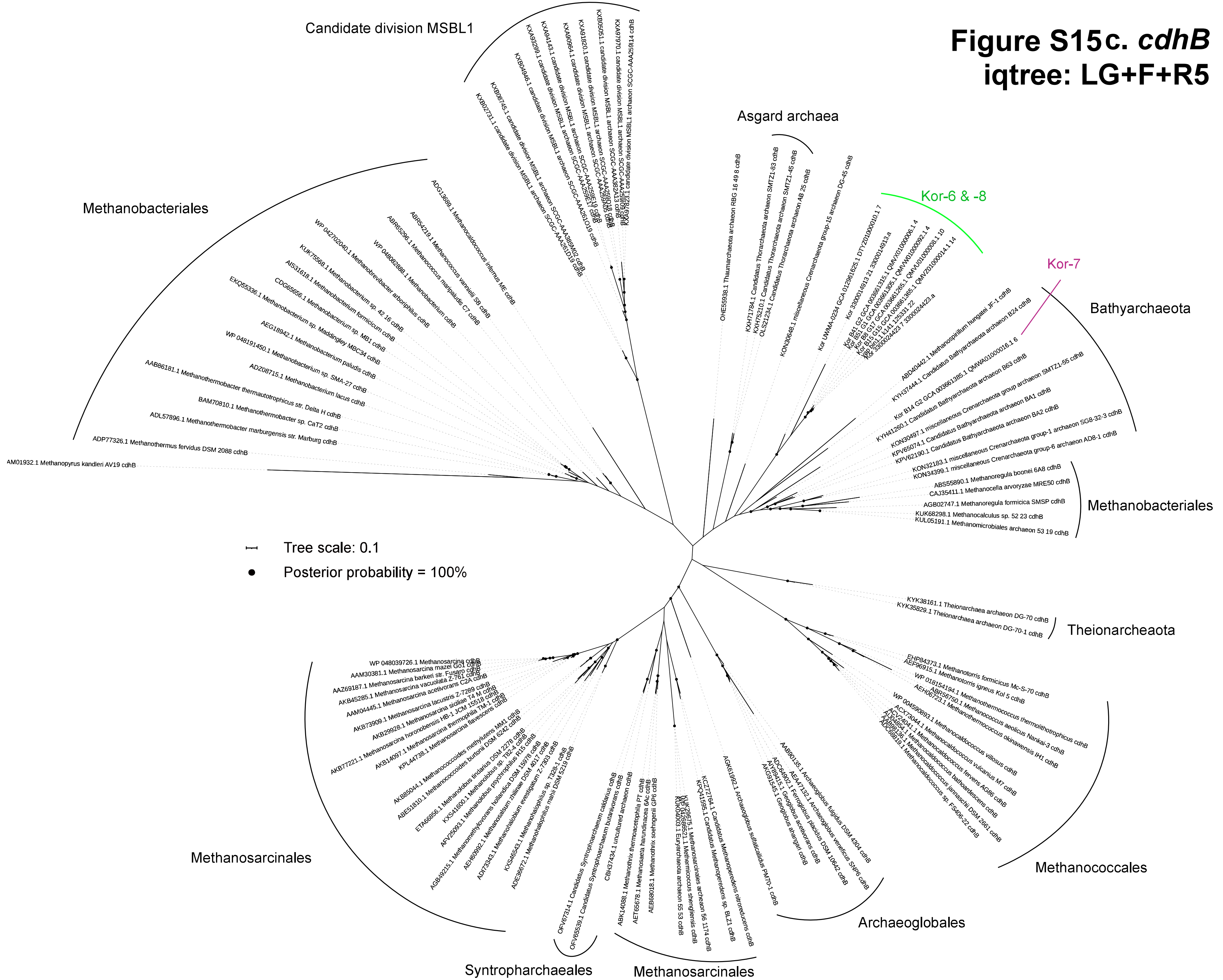

Figure S15c. *cdhB*
iqtree: LG+F+R5

Figure S15 d. *cdhC*
iqtree: Q.pfam+F+R7

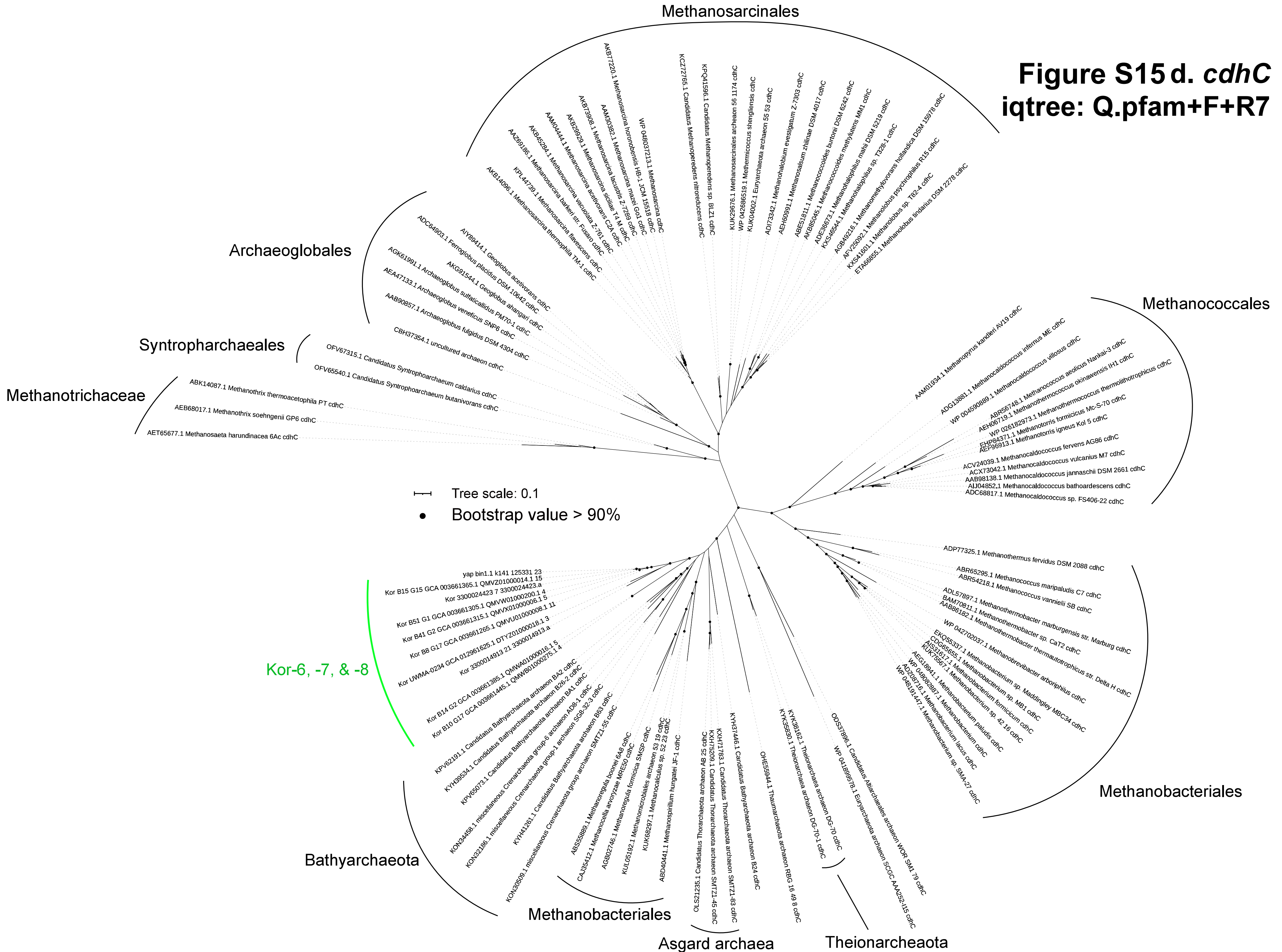

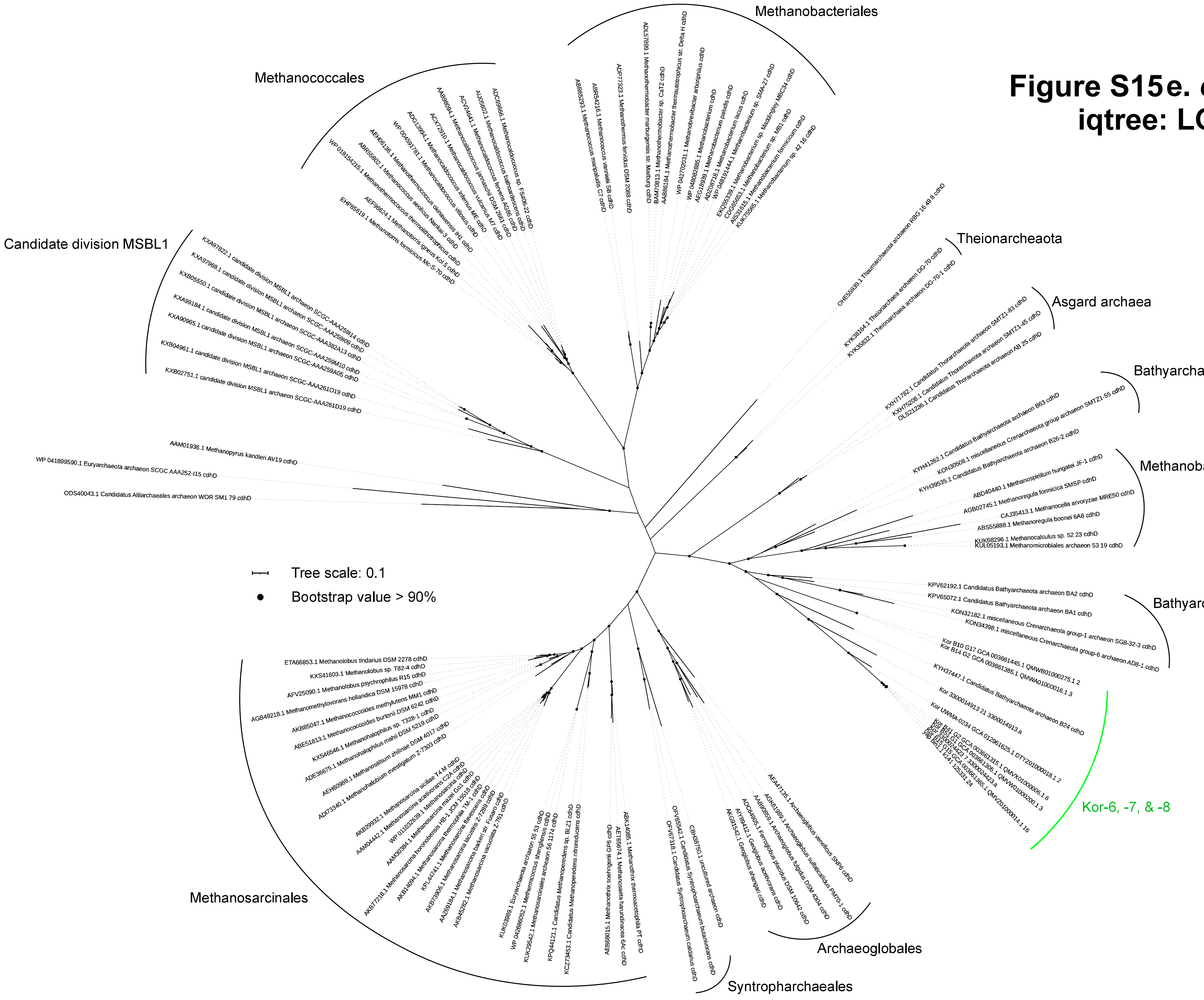

Figure S15e. *cdhD*
iqtree: LG+R6

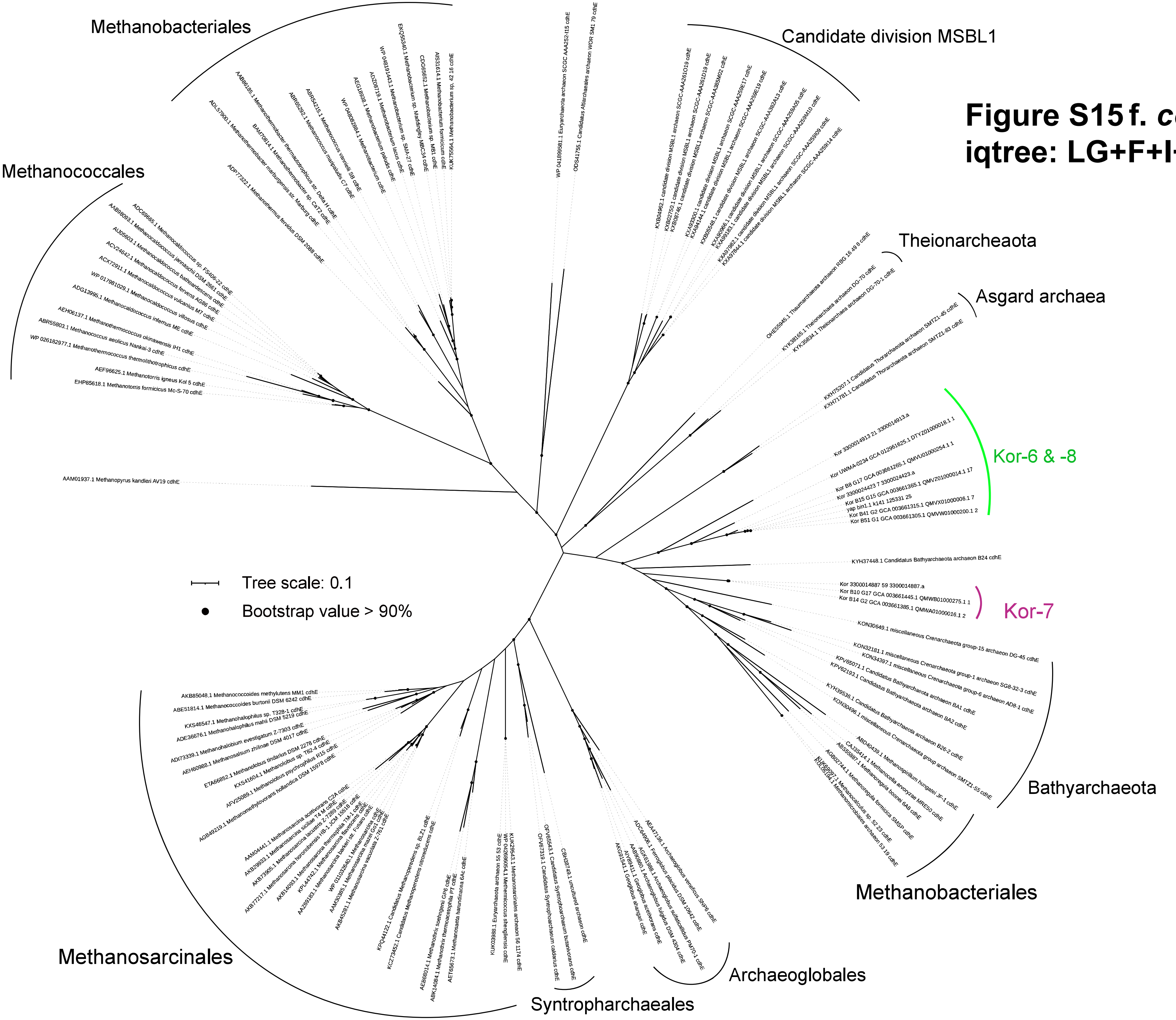

Figure S15 f. *cdhE*
iqtree: LG+F+I+G4
