# Supplementary material for: Wood–Ljungdahl pathway found in novel marine Korarchaeota groups illuminates their evolutionary history
Source: mSystems. 2023 Jul 17;8(4):e00305-23. doi: 10.1128/msystems.00305-23 (PMC10469681; doi:10.1128/msystems.00305-23)
Supplement: Legends — to the supplemental material. [file msystems.00305-23-s0005.docx]

## Supplementary Table legends

**Supplementary Table 1 Chemical parameters, metagenome size, and Korarchaeota abundance in each sample**

**Supplementary Table 2 Overview of 43 korarchaeal MAGs analyzed in the current study**

**Supplementary Table 3 Korarchaeota 16S rRNA gene sequences (n = 266) used in the current study**

**Supplementary Table 4 Overview of the evaluation and selection of marker genes for korarchaeal phylogeny construction**

**Supplementary Table 5 Twenty-one marker genes used for phylogenomic analysis and molecular dating analysis of Korarchaeota (Fig. 2).** The marker genes were selected from among homologous genes in MAGs (see “Determination of marker genes for phylogeny construction” in Methods).

**Supplementary Table 6 Taxonomic profiles of Yap trench samples in the current study and marine samples from Earth Microbiome Project (EMP) used in Supplementary Fig. 7**

**Supplementary Table 7 Counts of functional genes identified in the korarchaeal MAGs**

**Supplementary Table 8 Overview of the evaluation and selection of marker genes for archaeal phylogeny construction**

**Supplementary Table 9 Thirty-seven marker genes used for phylogenomic analysis and molecular dating analysis of Archaea (Fig. 5 and Supplementary Fig. 17).** The marker genes were refined from the “top 50% gene set” used by N. Dombrowski et al. (21). Each gene was selected by phylogenetic analysis (see “Determination of marker genes for phylogeny construction” in Methods).

**Supplementary Table 10 Overview of 283 archaeal genomes used for archaeal phylogeny construction**

**Supplementary Table 11 Estimated gene duplication, transfer, and loss event values calculated by using amalgamated likelihood estimation (ALE) algorithm**

**Supplementary Table 12 Details of the functional genes identified in the korarchaeal MAGs**

**Supplementary Table 13 Sixteen ribosomal proteins used for the phylogenomic analysis of Korarchaeota (Supplementary Figs. 2a, b) and Archaea (Supplementary Figs. 1a, b)**

**Supplementary Table 14 One-hundred twenty-two single-copy genes used for the phylogenomic analysis of Korarchaeota (Supplementary Figs. 2c, d) and Archaea (Supplementary Figs. 1c, d)**

**Supplementary Table 15 Fifty-one marker genes used for the phylogenomic analysis of Korarchaeota (Supplementary Figs. 2e, f) and Archaea (Supplementary Figs. 1e, f).** The marker genes were the “top 50% gene set” used by N. Dombrowski et al. (21). Six genes harbored by less than half of the analyzed genomes were discarded from analysis

## Supplementary Figure legends

**Supplementary Fig. 1 Phylogenetic trees constructed for archaeal MAGs using different sets of marker genes and methods.** The following trees are shown: (**a**) based on 16 ribosomal proteins, constructed using Phylobayes; (**b**) based on 16 ribosomal proteins, constructed using iqtree; (**c**) based on 122 archaeal marker genes, constructed using Phylobayes; (**d**) based on 122 archaeal marker genes, constructed using iqtree; (**e**) based on 51 marker genes, constructed using Phylobayes; (**f**) based on 51 marker genes, constructed using iqtree; (**g**) based on 37 marker genes, constructed using Phylobayes; and (**h**) based on 37 marker genes, constructed using iqtree. The 51 marker genes were from the “top 50% gene set” used by N. Dombrowski et al. (21). Six genes harbored by less than half of the analyzed genomes were discarded. The 37 marker genes were proposed in the current study (see Supplementary Table 8 and Methods for more information). The following program versions were used: Phylobayes-mpi v1.8 and iqtree v2.1.3.

**Supplementary Fig. 2 Phylogenetic trees constructed for korarchaeal MAGs using different sets of marker genes and methods.** The following trees are shown: (**a**) based on 16 ribosomal proteins, constructed using Phylobayes; (**b**) based on 16 ribosomal proteins, constructed using iqtree; (**c**) based on 122 archaeal marker genes, constructed using Phylobayes; (**d**) based on 122 archaeal marker genes, constructed using iqtree; (**e**) based on 51 marker genes, constructed using Phylobayes; and (**f**) based on 51 marker genes, constructed using iqtree. The 51 marker genes were from the “top 50% gene set” used by N. Dombrowski et al. (21). Six genes harbored by less than half of the analyzed genomes were discarded. The following program versions were used: Phylobayes-mpi v1.8 and iqtree v2.1.3.

**Supplementary Fig. 3 Phylogenetic trees constructed using 115 single-copy genes selected from among homologous genes in korarchaeal MAGs.** All trees were constructed by using iqtree. The individual tree models are presented in Supplementary Table 4.

**Supplementary Fig. 4 Bayesian trees constructed using korarchaeal 16S rRNA genes (a) and MAGs (b) using Phylobayes.** The Bayesian inference was calculated until the average standard deviation of split frequencies was <0.01.

**Supplementary Fig. 5 Chemical parameters and percentages of Korarchaeota reads in water sampled at different depths.**

**Supplementary Fig. 6 Geologic distribution of the hydrothermal sites in the western Pacific Ocean.** Data are from the InterRidge Vents Database (https://vents-data.interridge.org/)

**Supplementary Fig. 7 Multidimensional scaling analysis of taxonomic profiles in Yap trench samples from the current study and marine samples from Earth Microbiome Project (EMP).** The detail taxonomic compositions were in Supplementary Table 6.

**Supplementary Fig. 8 Maximum-likelihood trees constructed with korarchaeal 16S rRNA used in Fig. 1 and moderate-temperature Korarchaeota sequences from the EMP and the IMNGS database (Table 1).**

**Supplementary Fig. 9 Spearman correlation analysis of the chemical parameters and relative abundance of Korarchaeota.**

**Supplementary Fig. 10 Phylogenetic tree and the inhabited locations of the 16S rRNA gene sequences from Kor-1.**

**Supplementary Fig. 11 Multidimensional scaling analysis of homologous gene matrix based on Korarchaeota MAGs.** The homologous genes were analyzed by using OrthorFinder v2.5.2.

**Supplementary Fig. 12 Phylogenetic tree based on the *mtrH* gene.** Sequences predicted for methanogen and non-methanogen MAGs were analyzed.

**Supplementary Fig. 13 Phylogenetic trees based on 51 archaeal marker genes.** The 51 marker genes were from the “top 50% gene set” used by N. Dombrowski et al. (21). Six genes harbored by less than half of the analyzed genomes were discarded. All trees were constructed by using iqtree. All tree models are listed in Supplementary Table 8.

**Supplementary Fig. 14 Phylogenetic tree used in the amalgamated likelihood estimation (ALE) analysis.** The nodes correspond to those in the ALE output (Supplementary Table 11).

**Supplementary Fig. 15 Phylogenetic trees constructed using concatenated sequences of the *cdhABCDE* genes (a), and those based on single genes, *cdhA* (b), *cdhB* (c), *cdhC* (d), *cdhD* (e), and *chdE* (f).**

**Supplementary Fig. 16 Phylogenetic tree constructed using concatenated sequences of the *mcrABCDG* genes (a) and that based on the *mcrA* gene (b).**

**Supplementary Fig. 17 Evolutionary timeline of Korarchaeota groups estimated by using MCMCTree and a phylogenetic MAGs tree across Archaea.** The Bayesian tree was constructed using concatenated sequences of 37 marker genes (see “Determination of marker genes for phylogeny construction” in Methods). The three vertical gray bars represent the timing of the great oxygenation event (2,330 Mya), the predicted breakup of the supercontinent at the beginning of the Mesoproterozoic Era, and the predicted breakup of the Rodinia supercontinent, accordingly.

**Supplementary Fig. 18 Convergence plot of posterior mean times from two independent runs of molecular time estimation shown in Fig. 5 and Supplementary Fig. 17.**
